# Patterns of Genome Evolution among the Microsporidian Parasites *Encephalitozoon cuniculi*, *Antonospora locustae* and *Enterocytozoon bieneusi*

**DOI:** 10.1371/journal.pone.0001277

**Published:** 2007-12-05

**Authors:** Nicolas Corradi, Donna E. Akiyoshi, Hilary G. Morrison, Xiaochuan Feng, Louis M. Weiss, Saul Tzipori, Patrick J. Keeling

**Affiliations:** 1 Department of Botany, Canadian Institute for Advanced Research, University of British Columbia, Vancouver, British Columbia, Canada; 2 Division of Infectious Diseases, Tufts Cummings School of Veterinary Medicine, North Grafton, Massachusetts, United States of America; 3 Marine Biological Laboratory, Josephine Bay Paul Center for Comparative Molecular Biology and Evolution, Woods Hole, Massachusetts, United States of America; 4 Department of Medicine, Albert Einstein College of Medicine, Bronx, New York, United States of America; University College Dublin, Ireland

## Abstract

**Background:**

Microsporidia are intracellular parasites that are highly-derived relatives of fungi. They have compacted genomes and, despite a high rate of sequence evolution, distantly related species can share high levels of gene order conservation. To date, only two species have been analysed in detail, and data from one of these largely consists of short genomic fragments. It is therefore difficult to determine how conservation has been maintained through microsporidian evolution, and impossible to identify whether certain regions are more prone to genomic stasis.

**Principal Findings:**

Here, we analyse three large fragments of the *Enterocytozoon bieneusi* genome (in total 429 kbp), a species of medical significance. A total of 296 ORFs were identified, annotated and their context compared with *Encephalitozoon cuniculi* and *Antonospora locustae*. Overall, a high degree of conservation was found between all three species, and interestingly the level of conservation was similar in all three pairwise comparisons, despite the fact that *A. locustae* is more distantly related to *E. cuniculi* and *E. bieneusi* than either are to each other.

**Conclusions/Significance:**

Any two genes that are found together in any pair of genomes are more likely to be conserved in the third genome as well, suggesting that a core of genes tends to be conserved across the entire group. The mechanisms of rearrangments identified among microsporidian genomes were consistent with a very slow evolution of their architecture, as opposed to the very rapid sequence evolution reported for these parasites.

## Introduction

Microsporidia are a diverse group of intracellular eukaryotic parasites including 1,300 described species [1], over a dozen of which are known to infect humans [2]. These organisms were once regarded as primitively simple eukaryotes, however it is now widely acknowledged that they are extremely specialized, highly derived relatives of fungi [3]–[6]. Microsporidia are known to harbour very small genomes, the most extreme being the 2.3 Mbp genome of *Encephalitozoon intestinalis* [7]. Microsporidian genomes have shrunk in two different ways. First, their obligate intracellular parasitic lifestyle has permitted the loss of many genes whose functions can be provided by the host cell, so that that the number of genes is severely reduced, in the case of the complete genome of *E. cuniculi* to about 2,000 genes [8], [9]. Second, those genes that remain have been packed into a smaller space by a reduction in the overall size of genes (due to a paucity of introns and slightly smaller proteins) and by a significant reduction in intergenic spaces [8], [10].

A survey of genomic sequences from *Antonospora locustae*, a microsporidian that is distantly related to *E. cuniculi*, suggested that this compaction has further effects on the evolution of microsporidian genomes. Specifically, this survey showed that the order of genes in the *A. locustae* and *E. cuniculi* genomes share a high degree of conservation, and that this conservation was correlated with short intergenic spaces [10]. Conservation of gene order usually degrades rapidly in eukaryotic genomes [11], [12], leading to the suggestion that genome compaction could slow the rate of genome rearrangements [10]. This comparison also challenged the hypothesis that the rate of genome evolution may be correlated with the rate of sequence evolution [13], since the rate of sequence evolution between the two species is relatively high [10].

The previous study raised a number of questions about genome evolution in microsporidia and other compacted nuclear genomes that cannot be addressed using a two-way comparison. For example, if gene order conservation is conserved in other microsporidia, are the same gene clusters preserved among many genomes, or merely some level of conservation across the genome? When genes do move, do they move individually or in blocks? Similarly, does the conservation between two genomes correlate with their evolutionary relatedness, or is conservation of gene order driven by other evolutionary mechanisms such as gene co-expression [14] and recombination rate [15]? Surveying a third microsporidian genome has the potential to address some of these questions and reveal important aspects of other processes that may be related to genome compaction in microsporidia [10], [16], [17], as well as revealing whether conserved gene clusters are special, or only part of a more random but global process.

*Enterocytozoon bieneusi* is a frequent and problematic pathogen of HIV-infected patients [18], [19] and its phylogenetic position is slightly closer to *E. cuniculi* than to *A. locustae* [20], making this an ideal species from which to compare the conservation of gene order within the microsporidia. In this study, which is part of an ongoing genome sequence survey of *E. bieneusi*, three large supercontigs encompassing 429 kbp were annotated and compared to both *E. cuniculi* and *A. locustae*. *E. bieneusi* genes were mapped to homologues in the *E. cuniculi* genome to determine the overall level of conservation between these two species. These gene order maps were also used to identify conserved blocks of genes and how genes that are not conserved cluster elsewhere in the genome. Finally, a three-way comparison including *A. locustae* was used to assess how genome rearrangements evolved along the phylogenetic tree of microsporidia.

## Results and Discussions

### Evolution of genome structure between *E. bieneusi* and *E. cuniculi*

Open reading frames were identified on three contiguous fragments of the *E. bieneusi* genome, which encode 116, 127, and 186 Kbp. A total of 296 open reading frames were identified and, for each of these putative genes, the position of orthologous genes in the *E. cuniculi* genome was determined. In the *E. bieneusi* genome, 83% of all the genes found in these blocks have a clearly recognisable orthologue in *E. cuniculi* and these orthologues are scattered across the genome. The remaining 17% of the ORFs identified showed little similarity to *E. cuniculi* ORFs, a consequence of either high sequence divergence among microsporidian genes [21] or independent insertions/deletions of genes along their evolutionary history. A careful analysis of these latter ORFs showed that some were indeed true orthologues, that they shared synteny with other genes in *E. cuniculi* and that the low similarity identified between the orthologues was a consequence of very high sequence divergence.

**Figure 1:**
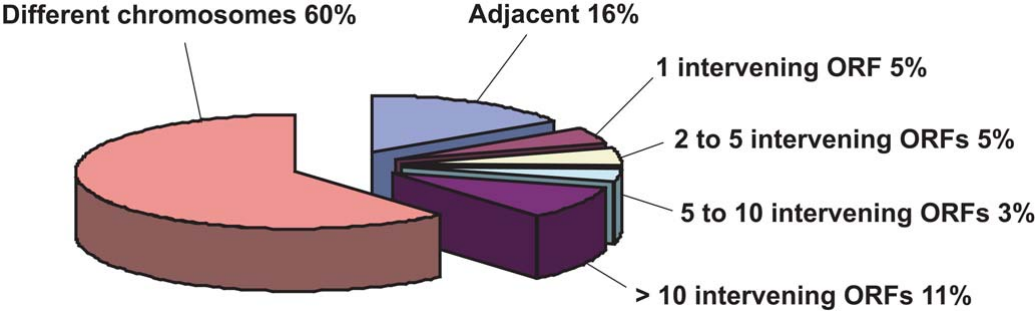
Percentage of gene order conservation between *E. bieneusi* and *E. cuniculi.* Each category displays the percentage of gene pairs in *E. bieneusi* that are adjacent, close neighbours, or nonsyntenic in *E. cuniculi*.

Overall, the *E. bieneusi* genome is compact, with around 0.7 genes per kilobase, but not as compact as *E. cuniculi* (∼0.97 genes per kilobase, [8]. Genetic distance between these two species is comparable with what has been observed between *A. locustae* and *E. cuniculi* [10] (p-distance of 0.52 averaged over 14 protein-coding genes). We found no evidence for repetitive and mobile DNA in the genomic regions we analysed, although some examples are known in *A. locustae* [22], *Vairimorpha corneae* [23], *Spraguea lophii* [24] and *Nosema bombyicis* [25]. Based on the identified orthologues, we first measured the degree of conservation of gene order as the percentage of adjacent gene pairs in *E. bieneusi* that were also adjacent or close neighbors in *E. cuniculi*. From 293 comparable *E. bieneusi* adjacent gene pairs, 16% were also adjacent in *E. cuniculi*, an additional 14% were close neighbors in *E. cuniculi*. Overall, 40% of the *E. bieneusi* paired genes were located on the same chromosome in *E. cuniculi* (Fig. 1). This shows that the degree of conservation is comparable to that previously reported for the *E. cuniculi*–*A. locustae* comparison [10] change to numerical citation format and that a relatively high level of genome order conservation therefore extends to *E. bieneusi*.

**Figure 2:**
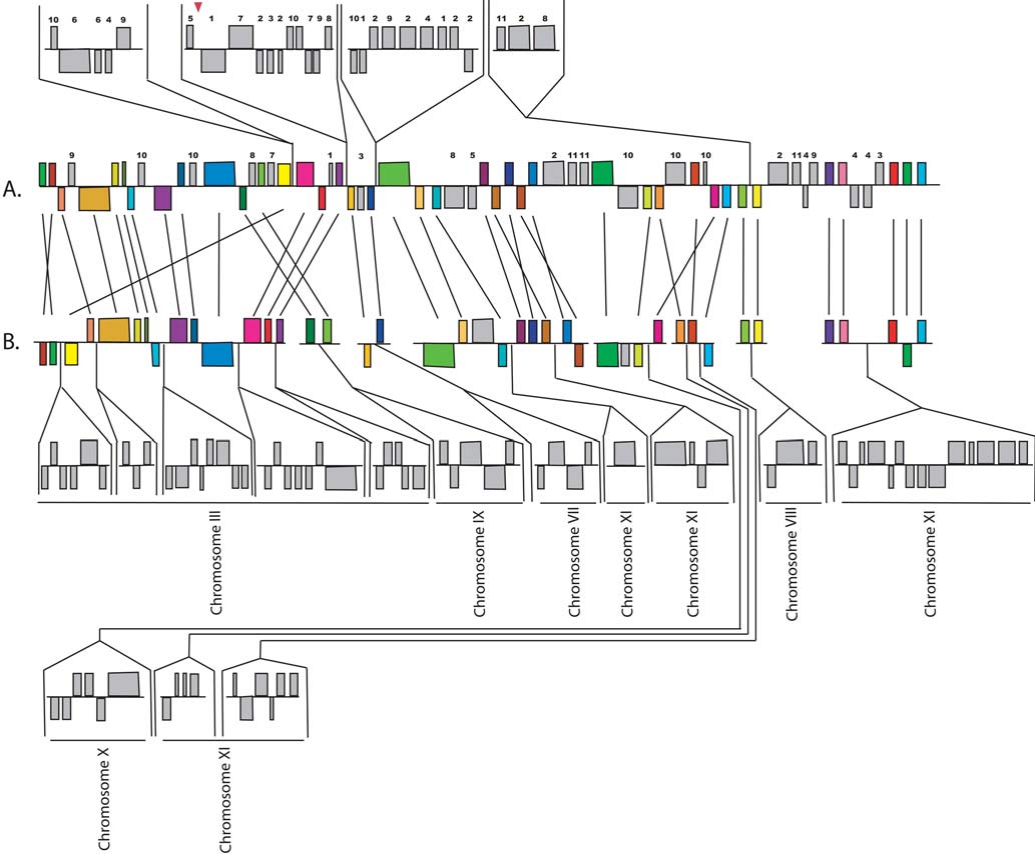
Analysis of contigs included in the scaffold SC_2384 and gene order comparisons between the *E. bieneusi* (A) and *E. cuniculi* (B) orthologues. Coloured boxes represent orthologues in the same context in both genomes, and the chromosome on which each *E. cuniculi* segment is located is indicated by Roman numerals. Grey boxes represent genes that are not identifiably orthologous or are not conserved in context. Regions of both genomes transposed above or below the aligned regions represent blocks of genes in different contexts. The chromosomal location of the *E. cuniculi* orthologue of such genes from *E. bieneusi* is indicated by Arabic numerals above the boxes. The chromosomal location of syntenic ORFs in the genome of *E. cuniculi* is shown in the lower part of the figure and annotated with Roman numbers. Straight lines join the homologous ORFs. Red triangles represent boundaries between the contigs used to generate the scaffolds presented in these figures.

**Figure 3 pone-0001277-g003:**
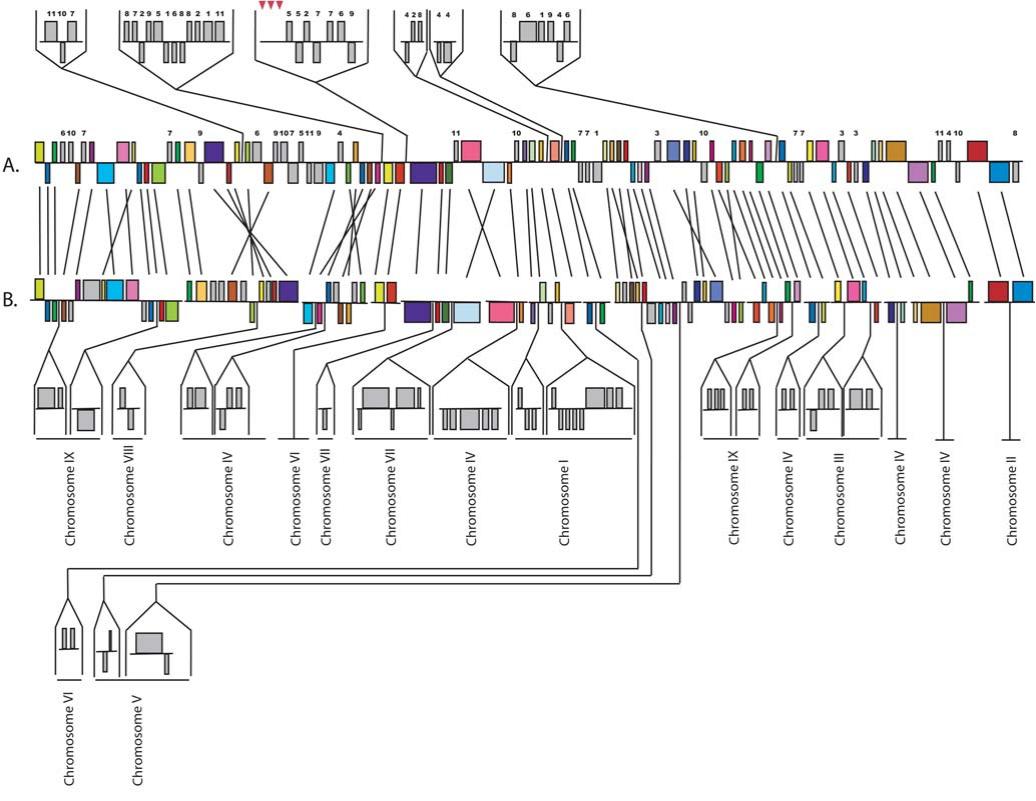
Analysis of contigs included in the scaffold SC_2496 and gene order comparisons between the *E. bieneusi* (A) and *E. cuniculi* (B) orthologues.

**Figure 4:**
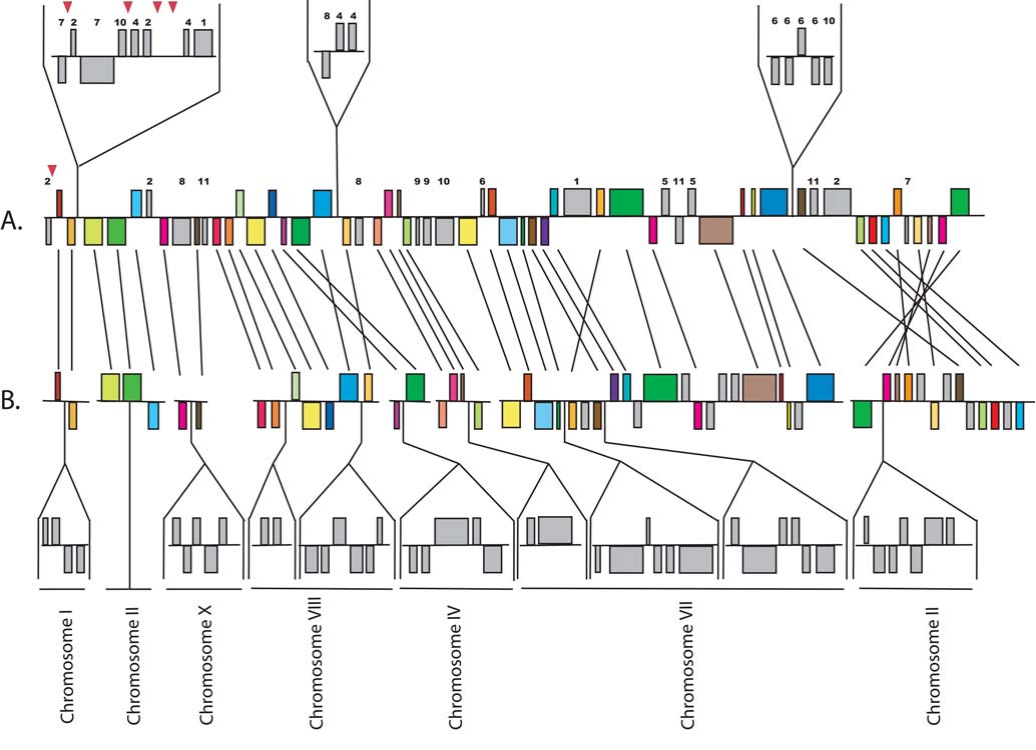
Analysis of contigs included in the scaffold SC_1888 and gene order comparisons between the *E. bieneusi* (A) and *E. cuniculi* (B) orthologues.

The comparison between *E. cuniculi* and *A. locustae* was primarily based on small regions of the *A. locustae* genome. The large *E. bieneusi* fragments allow insights not possible with these small fragments, so we compared in detail the order and orientation of the 296 *E. bieneusi* ORFs with homologous regions of the *E. cuniculi* genome (Figs. 2–4) to see what kinds of rearrangements are most common. As previously determined for other species [26], but at a much larger evolutionary scale, these comparisons reveal very little conservation at the highest level (i.e., for each *E. bieneusi* fragment the genes are found on many *E. cuniculi* chromosomes), but there were many blocks of genes where local gene order is highly conserved. Numerous small rearrangement events have also occurred within regions of conserved gene order, many including small inversions of one or more genes between *E. bieneusi* and *E. cuniculi*. This has also been observed in closely related eukaryotic genomes and is thought to be a widespread evolutionary mechanism in eukaryotic nuclear genomes [11], [27]–[29]. Larger inversions are also seen, including the complete inversions of syntenic regions of as many as seven ORFs (e.g., Fig. 3). Single-ORF transpositions are also evident in some cases (e.g., Ecu03_240 in Figure 2 and Ecu07_0270 in Figure 4).

The blocks of conservation are separated by regions of variable sizes where genes show no conservation of order. Many such regions are composed of a single gene or small number of genes, but in several, blocks of multiple genes exist where no conservation of order is found (i.e., grey genes above the aligned regions of the genomes in Figs. 2–4). The ORFs in which order is reciprocally conserved in *E. cuniculi,* but not in *E. bieneusi,* cannot be determined until the complete genome is annotated. However, both genomes appear to have been rearranged in blocks of genes where there are a few instances of reorganisation, and blocks of genes where there are few instances of conservation.

### Genome conservation across the tree of microsporidia

**Figure 5:**
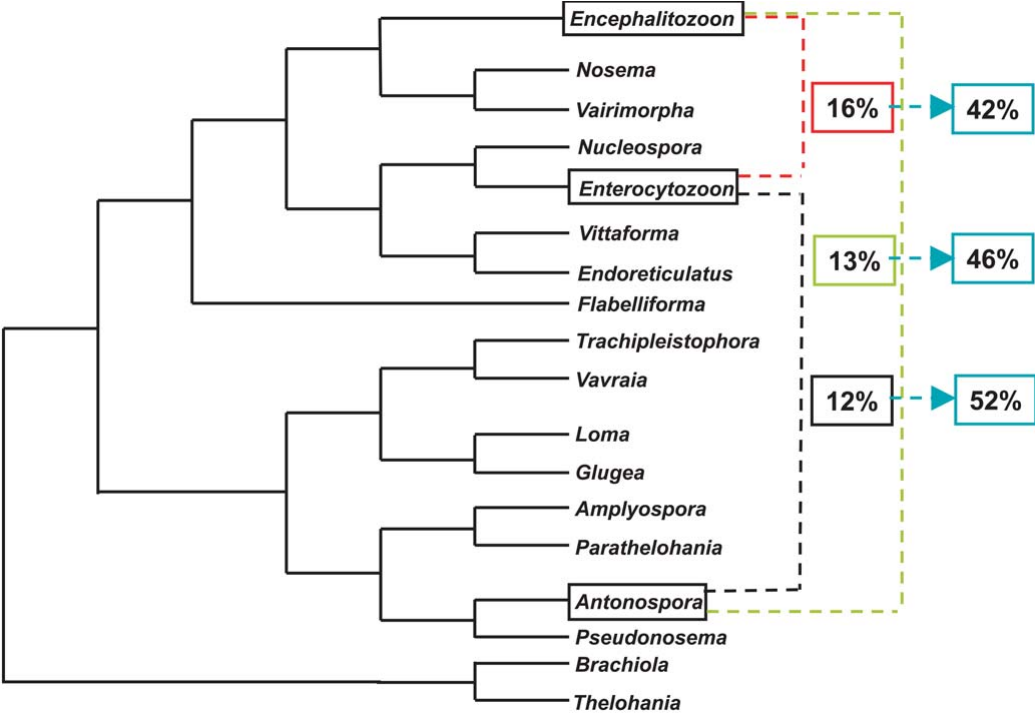
Schematic representation of phylogenetic relationships among microsporidian genera based on SSU rDNA sequences using Maximum Likelihood and genetic distances (Neighbor-Joining) [20], [30.] Colored boxes linking pairs of taxa indicate the percentage of conservation of adjacent pairs of genes. Blue boxes indicate the percentage of adjacent loci that are also adjacent in the third microsporidian species analysed in this study.

A schematic representation of microsporidia phylogeny based on SSU phylogenies is shown in Figure 5 [20], [30]. Molecular phylogeny consistently places the *Encephalitozoon* clade as sister to the *Nosema* clade, and the *Enterocytozoon* clade as sister to both. *Antonospora* falls elsewhere in the tree as sister to *Paranosema*, and is therefore more distantly related to either *E. cuniculi* or *E. bieneusi* than they are to one another. Generally, conservation of gene order correlates with the evolutionary distance between the species [11], [31], so one might predict a significantly higher level of conservation between *E. cuniculi* and *E. bieneusi* than between either of these and *A. locustae*. Indeed, *E. bieneusi* and *E. cuniculi* do share a slightly higher proportion of adjacent gene pairs (16%, Fig. 1) compared to that found between *E. cuniculi* and *A. locustae* (13%, [10]), but the difference is small and the level of conservation of more distant pairs is actually lower (40% and 44%, respectively, Fig. 1). Overall, there is no strong evidence for a correlation between the level of gene order conservation and the evolutionary distances between the organisms, suggesting as an alternative that the level of conservation might be static.

### Evidence for a core of gene pairs with conserved order among microsporidia

To more fully analyse the nature of genome conservation between all three species, we conducted a parallel analysis of genes in complete adjacency between *E. bieneusi* and *A. locustae* using a dataset including 94 gene pairs previously analysed in *A. locustae* [10]. The conservation of adjacent pairs was calculated for each of three possible pairwise species comparisons, resulting in values similar to those reported above and previously (Fig. 5). Specifically, the percent conservation of *E. cuniculi* and *E. bieneusi* to *A. locustae* were 13% and 12%, respectively, while the conservation between *E. cuniculi* and *E. bieneusi* to one another was 16%. The important question arising from this is whether the same genes tend to be conserved in order in all three genomes. To test this, we took all conserved adjacent gene pairs from each of the three pairwise species comparisons, and determined the frequency with which they were also conserved in the third species (e.g. for the 16% of gene pairs conserved between *E. bieneusi* and *E. cuniculi*, homologues for all genes were identified in *A. locustae* and the frequency with which the same pairs were conserved was determined). Interestingly, this frequency was significantly higher than the baseline frequency for all three of the pairwise species comparisons (i.e., 42% to 52%: Fig. 5). This result suggests that there is a core of gene pairs that were most likely linked in the ancestor of all three species, and are much more likely to remain linked than other genes in the genome (for examples, see Supporting Information File S1). This is also consistent with the lack of any pronounced correlation between the level of genome conservation and the evolutionary distance beween any two species. This correlation is likely more obvious in closely related species, but between such distant relatives, it could be overwhelmed by a relatively larger pool of genes that tend to be conserved throughout the group. Interestingly, when the regions conserved in all three species are compared, it is difficult to determine which species are most similar in genomic structure. In some cases, genomic architecture seems to be more similar between *E. cuniculi* and *E. bieneusi* (Supporting Information File S1, regions B, E, F), while in others, *E. cuniculi* and *A. locustae* share a more similar genomic context (Supporting Information File S1, regions C, I, J).

### Possible causes and mechanisms leading to genomic stasis in microsporidia

Examples of gene order conservation at deep levels of divergence are known in a few cases where order is maintained either by chance [32], because the proteins are part of a complex regulatory pathway [33] or because of coregulation of functionally associated genes [12], [34]. However, with the exception of two loci encoding ribosomal proteins, neither of the latter predictions seem to account for the conserved gene order across the three microsporidian species. Using bioinformatics tools such as InterproScan [32] we failed to identify any conserved gene pairs that appear to be part of a same gene network, or which are known to be functionally associated. Conservation was shown to be correlated with short intergenic regions in *E. cuniculi* and *A. locustae* and it was proposed that the compaction of the genome made it difficult to separate genes without introducing deleterious breakpoints [10], a factor noted to contribute to conservation of gene order in other genomes as well [14]. The recently discovered phenomenon of multigene transcription in microsporidia [16], [35] could also contribute to the preservation of gene order, since control elements of one gene may be situated within an adjacent gene, selecting against their separation. Indeed, three gene pairs that we have identified as being conserved across all three species (Supporting Information File S1, regions I, J, K), have also been found to show overlapping transcription in *A. locustae* [16] and in *E. cuniculi* (unpublished). This correlation would extend the idea that co-expression of genes in yeast leads to greater conservation in order [14], however, large scale analyses of transcriptional patterns among syntenic genes in the microsporidia are necessary to determine if a correlation between multigene transcription and conservation of gene order is a significant factor or a minor one.

One correlation that clearly does not hold is the proposed relationship between the rates of nucleotide substitution and genomic rearrangements, which are thought to correlate in other organisms [28], [36], [37]. From that point of view, microsporidia can be considered as exceptional for harbouring very fast evolving genes [21] within slowly evolving genomes. According to these results, it is tempting to speculate that two independent evolutionary forces are acting on microsporidia genes and their genome structure.

## Materials and Methods

### *E. bieneusi* genomic DNA isolation, genomic library construction and sequencing

The genomic data used in the study come from a genome survey project funded by NIH grants to the Tufts University group (manuscript in preparation). *E. bieneusi* spores were purified from fresh stools of infected adult humans using the method described by Zhang and colleagues [38]. Two independent genomic DNA extractions were carried out with using a modified proteinase K-phenol extraction protocol [39]. Using the two genomic DNA preparations, two random 2–3 kb genomic libraries were constructed by Agencourt Biosciences, Inc. (Beverly, MA) in a proprietary high copy number vector. In the construction of the library, genomic DNA was hydrodynamically sheared in the Hydroshear (Genomic Solutions, Ann Arbor, MI) and then separated on agarose gel. A fraction corresponding to ∼3,500 bp in length was excised from the gel and purified by the GeneClean procedure (Qbiogene, Morgan Irvine, CA). The purified DNA fragments were blunt-ended using T4 DNA polymerase. The 3.5 kb DNA was ligated to unique *Bst*XI-linker adapters and the linker-adapted inserts were ligated to *Bst*XI-cut vector to construct a “shotgun” library. Clones were sequenced using ABI 3.1 BigDye terminator chemistry (Applied Biosystems, Foster City, CA). In total 37,383 reads were assembled into 2,821 contiguously assembled segments. consisting of two or more reads, using the Paracel GenomeAssembler (PGA) (http://www.paracel.com/products/pga.html) with default program parameters and quality scores. 1,078 contigs were discarded as contaminating bacterial genomic sequence, leaving 1,743 *E. bieneusi* contigs. This Whole Genome Shotgun project has been deposited at DDBJ/EMBL/GenBank under the project accession ABGB00000000. The version described in this paper is the first version, ABGB01000000. Three large scaffolds (SC_2384, SC_2496 and SC_1888) were obtained based on contig mate pair information and used in this study. SC_2384 was assembled from contigs ctg01_153 (43326 bp) and ctg01_154 (83950 bp). SC_2496 was assembled from contigs ctg01_1952 (65945 bp), ctg01_2519 (1260 bp), ctg01_2570 (1227 bp), ctg01_2003 (1338 bp) and ctg01_1951 (118303 bp). SC_1888 was assembled from contigs ctg01_110 (2152 bp), 111 (3690 bp), 112 (6021 bp), 113 (3057 bp), 114 (2484 bp) and 115 (98412 bp). All the contigs used in this study are included in accession ABGB00000000.

### Annotation of *E. bieneusi* supercontigs and gene order surveys

Putative open reading frames (ORFs) were characterized in this study from the three *E. bieneusi* scaffolds (Supporting Information File S2). The genomic DNA sequence of the contigs was compared to the complete genome of *E. cuniculi* [8] using tBLASTX [40], [41] to identify all potential protein-coding genes. The list of putative ORFs identified along the three contigs, their description, their position and the accession number of their relative ORF in the *E. cuniculi* genome are listed in the supporting information file, Table S1. In parallel, to compare the level of conservation between *E. bieneusi* and *A. locustae*, the gene pairs used in the study by Slamovits et al. (2004) change to numerical citation were compared to the *E. bieneusi* genome database using tBLASTX. As this comparison was performed before the *E. bieneusi* genome was completely assembled, we limited our analysis to whether gene pairs were adjacent (no intervening ORFs) in each species, to avoid any biases due to contigs with different lengths or with incomplete ORF annotations. Finally, the set of adjacent gene pairs found in common between *E. bieneusi* and *E. cuniculi* were compared to the *A. locustae* genome database (http://gmod.mbl.edu/perl/site/antonospora), to determine the number of gene pairs conserved in order among all the three microsporidian species we analysed in this study (Fig. 5, percentages in blue squares). Function predictions were generated for the sets of syntenic proteins using Interproscan [32].

## Supporting Information

Figure S1 Examples of gene order conservation between *E. cuniculi*, *E. bieneusi* and *A. locustae*. This figure represent genomic regions of *A. locustae* previously identified by Slamovits et al. (2004) and Williams et al. (2004) numerical citations. Loci in the same order are shown in coloured arrows and are linked with straight lines. Transcriptional direction of genes is indicated by arrow direction. The accession numbers of *A. locustae* fragments shown in this figure are as follows. A. AY548887, B. AY548895, C. AY548905, D. AY548901, F. AY548898, G. AY548891, H. AY548889, I. DQ057555, J. DQ057548, K. DQ057569
(0.09 MB DOC)

Figure S2 Genomic sequence of the of the scaffolds used in this study.
(0.46 MB DOC)

Table S1 Description of the ORFs, their position along the scaffolds, and the accession number of their best match against the *E. cuniculi* genome.
(0.37 MB DOC)
