# Supplementary material for: Patterns of Genome Evolution among the Microsporidian Parasites Encephalitozoon cuniculi, Antonospora locustae and Enterocytozoon bieneusi
Source: PLoS One. 2007 Dec 5;2(12):e1277. doi: 10.1371/journal.pone.0001277 (PMC2099475; doi:10.1371/journal.pone.0001277)
Supplement: Figure S1 — Examples of gene order conservation between E. cuniculi, E. bieneusi and A. locustae. This figure represent genomic regions of A. locustae previously identified by Slamovits et al. (2004) and Williams et al. (2004) numerical citations. Loci in the same order are shown in coloured arrows and are linked with straight lines. Transcriptional direction of genes is indicated by arrow direction. The accession numbers of A. locustae fragments shown in this figure are as follows. A. AY548887, B. AY548895, C. AY548905, D. AY548901, F. AY548898, G. AY548891, H. AY548889, I. DQ057555, J. DQ057548, K. DQ057569 (0.09 MB DOC) [file pone.0001277.s001.doc]

A.

B.

C.

D.

E.

F.

G.

H.

I.

J.

K.
