# Supplementary material for: Patterns of Genome Evolution among the Microsporidian Parasites Encephalitozoon cuniculi, Antonospora locustae and Enterocytozoon bieneusi
Source: PLoS One. 2007 Dec 5;2(12):e1277. doi: 10.1371/journal.pone.0001277 (PMC2099475; doi:10.1371/journal.pone.0001277)
Supplement: Figure S2 — Genomic sequence of the of the scaffolds used in this study. (0.46 MB DOC) [file pone.0001277.s002.doc]

>Scaffold_2384

AAATTCTTCACTTGCAGAAGACATAAGGGTTAAAATTTTTTCAATAGATTAAAATACCAATTTACCATATTTTAAATAAATGCATTTTTTATAAATTTTATTTATGTATGATTTCTTCTTCGGGTATTACAATTTTTTTAATTTTTATCATTTTTTTATTTGTTATTCGCATAGCTAAAAAAAAAGATCCTATAAAAATTATAATTACTGAAAAAATAAAGTTTTGTATTTGTTTAACTGAAAAATGTTCAAAATCTTGAAAATATATACCGGCAGTTCCAATAGAAAGTAAAGTCCACATTCCATGAAATAAAGGACAAACTATTAATGCATCATAATGTTTTAACGCTCTATTTAACCAATATATTTGCAAAAATGTACACAAAATAACTCCAAAAATAAATAAAAAACCACTCTTATTATATAACATTCCTTTATCACCATTAATTGCTTTTAAAAGAATTTCACCTAAACTCTTAACACATAATGTTGTAAATGATGCTATAAATGAACTTAGACCAATATACGCAAACAGCATAATATATTTGCAAATAAATGAATCTTCATCAATAAATAATCCTTCAATTTTTAATATATTATTAAATGGATCATTATAAAAATTCCAATCTGAATTAACTTCAACGAATTTAACAAATAAAAAAAAACCTATTATTAATATAATAAGAAACATGAACCATACTAATGTTTCTGTTTTATAATACATTTTAATTAATTCACATAGTGTATATATATTATGACTTGTCGCTGAATTTAATACTAAAATTGTTGACCCTATAATAACTAATATAATGGCCACAATATCTTTCCATGTAATAATTTCATTGTTAATTAATGGTGCACAAAATGAATTGGCTATTAATCCAATTGCAGATAATCCTGCCATTAAACTTTGATTACCGAAAATATATGCACTAAATGAAATAATTTTACCCAAAATGTATATTATAACTCCAAATAACATTGTACTAATAAAAATTTTATTAGGCGAAATATTTAAATAGTTTTGTTTATAACTTAATTTTTGATAATTGATTCCAACTGTAATGACTATATTTCCAGTAATAGAACATATCATAGCTAAAATCCACAGCTTTTCTTTTTTATTAACACATTTATTCGCACCATCTATAAAGGTACATACTTGATTTTGATTACAACCACTTAGATATCCAGGTCTATTTGAATTACATTCACCATATAGTGGAATACCTTTACAACAAAATCTAGTATTTTTTATATTATTTTGATTCATTTTGCATGATAAACTATCATTTTTACATTGCATAGAACATGCTAATGGACCATAATAATCATAAGAGGCACATTCTTCAGGGAAATTACATGGGAATCCACTTCCTTTCAATTTAATGCATAGTCCATGAACACAATATAAAGAATTAAAGTCTGGTATAGAACTCAATCGACAATCACTGTCTAATATACATGTATTAATAGTGTTTTTTTTAATATCCATTTCATCAAGATTTATAAGAAAAGAATTTTTTAATAGGTTTATCATGCTTTGTTTATTTCTATTTTTTATCTATTTATATTTTGTTTCAATTTTATGCTTAGTGGAATATTTTTAAAATAAATATATGTATATATTAGTATTAATCGAGGATCATTTATTTTAATACACCATTCTTTTAATATTGTAAAATTTTTTATCTCAGCTTCATTATAATCTGTAATGTCAATATCAATTAATGTTTTATTACAAATTGTTAAATTAAGTTCAATCTTGTAAAATATTGTAATTTTATATTTATTATCTATTATTTTATATTTAATTTCTCCAAATATTGTTTTATAAATAGTAGATATCATTTTTTGCCAACAAAAAATATCTTCAATATCATTTGTAGTATTAACAAGAGTGAGACGTCTAATTTTTTTACTTAATGATATTTTTTCATTTTCTATAGATTGTTGTTTAATCATAAGTTCATTAAGTTCATTAAATTGCATTTTTTTTTTTATTTGTAAAATATTATTGTATAATAAATTATTTTTTAATTTTATATCATTATATTGTTTCAATATTGTATTTTGATTACTTAAATTAAATTTTATTGTTTCCATATTTTGGTTTTTTACTGTAATCTTTTCAAGTGTTTTAGTTAATTCCATATATTCTTTAGATAGATTTTTTATTTCTATATTTTGAAAATAAATTTCATTACTTAATGATTTAATACTATCATTTAATTCTTTAAAGGTATTTTTAGTATTTTTTAAATCTTCTTTTGCTGTTTTTATAGTATTAAATGCAATAAAATCAATATTTTTATTAATTAAATTTTGGTAGAAATATAATTTACTTTTACGTTCTTTATAAGCAAATTTAACATTTCGAAAATATTCTTTAATATAACTAAATTCATTTTCGATGTTATTTAATTCACACGTTTCAATTATTTCATCCATTAAAAATCTTAAGGGTTATTTATAATATCTACAGTAATAAAAGTTTAAAATGTTTATAAAAACAAAACAAAACTTTCCCTTGCCATTTATGACTAAAGAGTTTGCACAACATATCTGTAGCCAAATAAATGCTCCTATATGTAATTCAGTAAACTTAGAAACAACATCATATGTAAGTAGATCAGGAAATTTATTTGGAATAATTATTGATTCTCCATATACTTTTATGCTAAATATATGTGGAATATTATTAGGATTAAGAATGTACTATAATACTTCAAATATGCATGCATGTATTGGAAAAAGTGAAATTAAATGGATTTTGTTTCTTTTTATAGTTAACAATATATTATTGATATTAACTATCAATTTTCAAAATTTTATAAATGGCTTTTCAACAATAATTTATGATATAATAGCTGCTTTTCAATTAACAATTTTTACAACTTATTATTTTACTGTTTTTGCTTCTGGAATAACAATAGATAAAATTCATGGAATTATGAAAATGAACTCAGGTGCATTTTTAAGCACTTTAAGTAGTATATTTATTTGTATTTTAAGCAGTTTTAATATTATTAGTATTGTAATTAATAATTATTTTTTATTACTTGGTTCAATAATAATGAATACAATTTCTATATTAATGTATATCGGAACTCAAACTAGAAAATTAAAAAAAATTAAATCAGATATTTGGGCATATGGAATATTATCAATATTATTTTCAATATATATCATATCTTTAATTCCAATATTTGTTGGAGCAGATATTATTTCTATTTTAACAGAAAAAAATTTAGACAATTTTTTTATTTTTCATTCATTAAACACAATTTTTATGTTAATGCTACATAAATATTGGTTAAGTACTTGTGATTTTGAAATAGAATGTTTAGAATTGGAAGTATAAAATTATTTGTTAATTATTTATTTGAAAATAAACTAAAAATCTGTGATTTATATTTTTATTATAATTAAATTTTTCTTTTGAAAATATTCCATTAAATCGTTTATGTTTTATTATTACATTTTTTATTCGTCCATGAACTAATGCCATAGCACATGAGTAACACGGTTCTATATCTAAAATACAATCCATTCCTGTACAAAGGTAACCATTTTGATTATTTTTAGATATTATACTAATTGCTTCACGAATAATATGTACTTTATTTAAAAATCTATGAATTTGCCCCATAGTGTCAATAATAATACAATTATGTGCACAAGCTATTGCATTATCTATATTTAATTCCATTAAAATATTAAATATACTATCTTTATTAATAAAATTTTCTTTAATAATATTTTTTGTTTTATAAATAGATGGCCAAACAGTATTTGCCATTTGAATTTGATTCGGTGTTAATGGTTGAAATCCAGGAATATTTACCTTAATAATTTTTTTATTTATTAATGTGTTAGACTGACCTATAAGCAAATAAAAATTATTATTATCAATTTTTTTTACTTTTTTTAACCATGATTCAATGTTACTTTCTTTTAAATATATTTTTATTTCATCTTTATTAACAATTATTGCATTACATTCTTTAAAATACATGATTTTTTCTTCATAATCTGTTTTATTCAACTCTAATTGTATATTTTTCATAAATTCATATATTTTTAAGGGCAACATAACCCATGAAATAAAACAAAATTTTAATAATATATTTCTTGAAATACTATTAAAAATTCTATGTTAATTATATTATGTTATTATTTGGTTTTATTTTTCAATCTAGTAGAAAATTTTACCCATTATTATGGCTTTTAAACTGGATAAAAAAATTATAGGAATTAATTTCGGTTTATTTTCACCAGAGGAAATAAAAAAAACAAGTGTAGTTCATGTGATTCATCCAGAAACAATAGAAAATGGTGTTCCAAAAGAAAATGGTCTTATAGATCTCCGAATGGGTACTACAGAAAAAAATTTTTTATGTCAAACATGCAATGAAAATATGTTTGATTGTGTAGGTCATTTTGGTCATATAGAATTATGTAAGCCAATGTTTCATATAGGATACATCACAAAAATAAAAAAATTATTAGAATGTATTTGCTTTTATTGCTCAAAAATAAAAGATTTTAAATTATTAAAAGAAAAAACAAAAAATTATACTTTAGAAGAAGCTTGGAATATTTTAAAAACGAAAACATTTTGTGATAGTGAAAATGAAAAAGGTGAAAAAATTGGATGTTGTAATAAACAGCCAATAATAAAAAAAGAAGGTATGAATTTAATAGCATTTATGAAAGGTAATGATGATGAAGGAAAAGTTATACTTAATGGAGAAAAAGTTTGCAATATATTTAAAAAAATTGATGCGTCTGAATTAAAGTTTATAGGCTTTAATGTTAATAGTCGACCTGAAAATATGCTAATAACAGTATTATTAGTAACACCACCATCTGTTAGACCATCTATATTAACAGATGGTTTAAGATCAGAAGATGATTTAACTCATAAATATGCAGATATAGTCAAAAGTAATGCATGTTTAAAAAAATATGAGCAAGATGGAGCTCCAAATCATATAATAAGAGATTATGAACAATTGTTACAATTCCATTTATCAACTCTTATTAATAATGAAATTTCAGGTCAACCTATTGCAATGCAAAAAAGTGGTAGACCTATTAAAAGTATTTCAGCACGATTAAAAGGAAAAGAGGGGAGAATACGAGGAAATTTAATGGGTAAAAGAGTTGATTTTTCTGCTAGATCTGTTATAACTCCGGATCCTGCAATTGAACTAGATGAAGTTGGAGTTCCATTACATATTGCTATGATTCAAACATTTCCAGAAAAAGTTAATTCAATCAATATAAATACTTTACAAAAAATGGTAAATAATGGACCGACTGAATATCCAGGAGCTAATTATGTAATACGTAATGATGGTCAAAGAATTGATTTAAAATATAGTCGTTTTGATTTAAAGTTAGAAAAAGGATTTATTGTAGAACGGCATATGCTTGATAATGATATAGTATTATTTAATCGACAACCATCATTGCATAAAATGAGTATGATGGGACATAGAGCCAAAATTATGAATAATAAAACATTTAGATTAAATCTTTCAGTAACATCACCTTATAATGCAGATTTTGATGGAGATGAAATGAATTTACATATGCCACAATCATACAATACAAAATCTGAATTAATAAACTTGGCTTCAGTATCACGAAATATTATTAGTCCTCAATCTAATAAACCAGTTATAGGAATAGTTCAAGATACTTTAGTAGGAGTAAGATTGTTTACATTAAAAAATACATTTTTTAATAAAAGAGAAACAATGAATTTATTGTTTACTCTTGATACTTTAGAAGAAAAAGTTATGGAAATTATTAATAATCCCATTCTTATTATTCATAATAAAATCAGTTTATGGAGTGGCTTACAAATTTTTTCTGCTGTTTTTCCAAATAAATTTAATTTTAATTATAAAGGTGTAGTTATTAAAAATTCAAATTTAATTACAGGAACTTTAAGTAAAATAACATGCGGTGCTACTCATGGTGGTTTAATTCATGTTATTTGCAATGATTACGGATGCTTAGAAGCAACAAAATTCATTAACAATGTTCAAAGATTGATAAATCATTTTATGTTTCATATAAATTCATTTACTATTGGAATAGGCGATACTATAATTAATCCTGAAATTATTGAGGATTGTAAAAGTAATATAGCAAATGCAATTCATAATGTCGATGAACTTATAAATATGGCTAAACTTGGTAAATTAGAAAAACTTCCCGGATTAACTATTGAAAATACATTCGAATCTAAAGTTAATTTAATATTAAATAAGGCTAGAGATTATAAAGGTGAAATAACATTATGTAATAATATGAATGCAATGATTCTTTCTGGTTCAAAAGGATCTAAAATCAACATAGCACAAATGACATCTTGCGTAGGCCAGCAAAATGTTGAAGGAAAAAGAATACCTTTTGGCTTTGAAAAGCGAACTCTTCCTCATTTTCAAAAATTTGATTATTCAGCCATATCCAGGGGATTTATTAGTAATTCATATTTAACTGGAATGACTCCTCAAGAATTTTATTTCCATGCTATGGGTGGAAGAGAAGGATTGATTGATACTGCTATTAAAACAGCTGAGACAGGATACATACAACGAAGATTGGTTAAAGCAATGGAAGACGCTGTTGTTAGAAGTGATAGTTCAGTTAGAAATGGATTTGGAAAAATTTATCAATTTGCATATGGAGAAGATGGATTTAATGGTAAAATACTCGAAAAAATAATTTTAAATAAGCCTACAAAAGATGAGTTTTATATTGATATTAATGATATTACAAATACATTAAGAAATAATAAATCATTTATTCCACGAGAAGTTATAGAACTTCTTAAGACAGATGTTGAAATACAAAAGTTAAACAATGAATATTATAATTATTTAGAACAAAATTATTCATTGTTAGATAATGGCATGTATGTTACACCTGTAAATATACAAAGAATACTATTGAACAAAAAACTTAATAATCGTATTAAATGCAATTTATCACCTTATATTATTTATGATGAAATAGAAAAATTAAAAACAAATAATTTATTATGGAATTACTATGTATCATATAGTTTAAATCCTAAAAAATTATTACAAACACAAACAAAATCAAACTTTATGGAAATTGTTAATACTATTAAAATCAAATTAAAAGAAGCACAAGTAGAACCAAATGAAATGGTTGGAACTTTAGCTGCACAATCTGTTGGGGAACCTGCTACACAAATGACTTTAAATACATTTCATTTAGCTGGGGTTGCATCTACAATTACTATGGGAGTACCTCGATTAAATGAAATTATTAATACTACAAAAAATATAAAGACGCCCTTAATGATTGTACAACTACAGAAAAACGATTTACAATCATGTAAAAACTTGAGAAACAAAATTGAATATTCATCGTTAAAAGATTTATATGAATCTTTTCTTCTTTTATATGATCCTATAATAACCGATACAATTTTGGATGAAGATAAAGAGTTAGTAGAAAATTATTTTGAAATGCCAGATGAAATTGATTTAGATAAATGTTCATCATTCTGTATTCGATTTGAATTAAATAAATATCATGTTATTGGACGTGGACTTGATTTAGAATATATAATTGAAAAAATTAAATTAAATATAAAAAGTATGTTAGATATAGGTATACATATTATTTATAGCCTACCAACAGATGAAAAATTAATTTTAAGAATAAGATTAATTTTGAATGATAAAATTAAAGAAAATTTAAAATTTTATAATGAGCTAGTAGATAAATTATTAAATATACATATTCAAGGATTAGAGGGTATTAAAAAAGCATATTTAATTCATAATAAAGATGATACATGGACATTACAAACAGATGGTATTAATTATCAAACTATTTTATCATTTCCAAATGTTGATGGTAGTCATACTACTTCAAATGATTTATTTGAGATATATGAAACTCTTGGAGTTGAAGCATTAAGAGAAACGATTATGACTGAATTAACCATGGTAATAGAAAATAATGGATCGTATGTTAATTCTCGTCATTTAAGTTTATTATCAGATGTAATGACTGTTTGCGGTTATCCATCAGGTATAACAAGACATGGAGTAAATAGAGAAATAAAAGCTAGTGCTCTAAAAAGAGCATCATTTGAAGAAACAGTTGATATTTTACTTGAAGCAGCAGCTACCGCAGAAGAAAATCCAGCACATGGTATAACTGAAAATATTATGATGGGACAATTAGCACCATTAGGAACTGGAAATATTCAGCTTATATTAGATATAAAAAAATTAGATCAAATTGTAAATAAAGCCTTTATTAATACAGTCGATAAGAATTTAATTATGTTAAATTCACCAGCTTTTACAGAAGCATTATTAATAGAGCAGGAAAATTGTATTCTCTCAGAAGATATTGGTTTATTTTCTCCTGAACATGAAGAATTGTCCGATTTTAACAATATAAAAATACCAGAATATAATGATTCACCTAATTCTGAACCAAATACATATAATTCTTTAAATACTCCAATATCATCTGAAAAAGTAAACAATAGCGTTATGTATACTCCAAATTCTTTTTATTTCAATCAACAACCTGAATCTATCTATTTTCCAGGAACAAATGGATCATATTTTGCAGCTTCAGGAGAGTATTTTCAATCAAATAATTTTCATTCCAATACTATTTTAAATAGTGGTGGATATCAAGAGTATATGGCGAATAGTAATGATTATGATAATTTATCCCAACAATATAAACCAATTGATTCACCAAAATATAATTATGATAATATAACTATTTATAATTATTCTAGTGAAGAAGAAAAAAAAGAAGAAAAAGAATAATATATATCACACTAATAATTAAGTATTACTTATTTTTTTTGCTATTAATAAATTAATATAACATATTAATCCAATACTCCAATACATTAATGATTTTCGTCGTACGGATGTTTTAAGTATTCCAGACATTATATTTATTAAACTAGATATAAATCCAATAGTAAATAAATTTGTACACGCAAAAACTTGCTTGTAATTTTTAAAATTTCCATTTATATAATCAGGCTTTATAAATGTATCTGTTATAGATAATAAATAATTAAGTAAAAAATATAATACATTACCTATTTTTGTTGTTGGCGCATATATAAAACAAATGAATGTAATTAAAATTAATGGAAAGATACAAAATCCTAAGTTATAAAAATCAAATCCCTGAGATTTACACCATTTAGTTGTTACAATTCCTAATATAGAGCTAAATATTTTAACAATTCCAATCACATATCCAACTCCTAAAATATCTTTTTTTTTATTTTGTGGTATAGTACATGTAATAAATAATAAGAACTTAGTTAATAAATTAATTATTTTATATATAACTAAATTAATTAATTTTATAATATTAATTATTTTAATTATATATGATGGATAATATTCATTATTTACTATTAGTGTTTTTTTAGAATTTTCACTTATTTCAAATAAAATTCCATTGGCAAATAATCCAAGAAATAATCTAAACATACTTACTAATATTTGAAATAAAAATGATATCTTTTCTCTTGATTGTAGCTGGTTAAATCCTTTAATTAATTTTATATACCATCCTATTAGATTGAAATGTTCTGTAATTTTATTATCACATGTAGAATTATTTTGTGTTAATGCCATAACTATAGCAATAATATATATAATTGATGAATAGAAAAAAATTAATTGTGACCCACCTGTTTCAAAATATAAATCCTGAGCTATCCACGCTGCTAATGAACGAATAGCATCAGATTGTGCTTTCAATATTTGCAAAAATATATTTTGTGATTCTGATCTAGAAGGCTTAGTAATTAATCGAATAGTTGATTTCATAATTTCGGAAATTGATGATGTAATATAAGCAGCAATTATTAATATAATACTTAGAATATTATTCAAACTTTTATCATTTAATGTTTTATTATATGAAGGAGCAATATAAACAAGTAATAATGTACAAAAAGGTAATAAAACAGTTATTAAATGATACCAATATAATGGAATTATCATTAATAAATTATATACAAAAGGTAGTGCTATACAACAACTATGGAAATACCATGGAATTAATTCATTAACTATTTTTTTAGTAGTATAATTTTTTTCTAATTTTATAATACTAAAATATTGATTGATTATAGGTGCAAAATCTTCAATAAGAATTATATTAAATATGCTTACCATTCTACAAGGGTAAAAAAAGTCTTAAACTATTTCCATATTTAGTATCTCCTTTATGAAATTTATTACTATTATAACAAGTGACACAAAAAAATTTTATTTAAATAAAACTCAATGTACAAAAATTAAATTATTTAAAAATTTGTTGGAAATATCTCATGAAAAAACTATAACAATTAATTTAACAAGTAAAATATTTAAAAAAATTTATGAATTCCTTATAACATATGATAAAGTTAATTTAAAAGTTGATTTAAATGACGAGTATAATCCTCTTGAATTATATTTTTCTAAAGATGATCTTGCATATTTAGAAAAATTTTCTATTGATAATATTATTGAATTAATAAATGCATGTAATTATTTAGAATATTCATATTTATTAGAGTTAAGTTGTAAAAAATTAGCAATATTACTTAATAATAAATTGTAAAATTTTATAGAATATACCCTTTTATGTCACTTAATCATATTGATATTTTAAATAAATTGCTTGATCAAAAAGTAATAGTTAAATATATAGACAATATAGAATTTGAAGGAATATTAAGAAGTATAGATGGATATCTTAACGTAGTTTTAGAAAATGTATCAGTTAAATTTATGAAAAATCATATAGATACATTAAATCATTGTTTTATTCGTGGCATACAAATTAAACATATTTATATATCGTAATAAAAATTTATTTATATGATAAAAAAATTTATCGGTATGATTTCTGATATCTTGCTCTTGCACCAGATCCACCATATTTTTTTTGTTCTTTAACCCTTGTATCTGTAACTATAGAAAATCTATCAAATTGCATTAAATTTTTTTGAATTTCTTGCTTTTTATATTCGTCAGAGTGTGTTCCAAAATATTGTAAAACCGCTCTGCAAAATGCTTGACGAACAGAATAAATTCGCTCCGAAAATCCAGACTCTTTATTAAATGTGATATCAAAATCTAATCCTGTTAAATTATTTCGTCCAATGATTTCTATTACTTCATTTAATTTAGCTATCATCACATAATCTTGGACTAAATTTAACGGAACTTTATTAATACGAATTTCAAATTCACCTGATTTAAAACATTCGCAATGTGCTATTGAAGCTTTTTTAATTCCTTTTGTTAAAATTTTTGCGCTCATTATGAAGAAGGATTAAGGATTTATTTTTTTAAAAATTTTACAAATTTTTCTTTATAGCTTTACCCCATTATAAATGAAGAGAAATAATGCATTACCAAATAATCATTTTAAAAAAACATCTAAAAGAATAAAAACATGGTTTGATCAACCAGCTAATAAAGAACGACGAAGAAGTATTCGTTTAATAAAAGCTAAAAAAATATTTCCAATGCCGTTAGAAAAATTACGACCTATTGTACGCTGTCCTACAATTAGATATAATAAAAAGGAAAGGTTAGGTAGAGGATTTACTCCAGAAGAATGTAAAGCGGCAGGATTAAATCATATATATGCCAGAACCATTGGAATATCCATTGATTTAAAACGAAGAAATATGAATCAAGAAACATTTGATCAAAATGTTCAACGTTTAATAGAATATCAAAGTAAAATTAAAATATTTAAAACTAAATCAGAAGCTAAAGGTGCTATACAACATATGAAACATATTATGCCAATTGTTAAAAATGAACCAATTGTAGAAGTAATGAATTCTAATGACATTATTAATTATAATTAATAAGATATTGTTAATCAATTAAATAATATTTTAAATTTAGTTGTGTATAAAATAATGTTTGTTAAGTAATTTTTAATAACGTTATTTTAATTAAAATTAACTAGTTTAGTTTAAGATAAGTTGAAATTAATCATAAAATAATATACTAATATACATTTTATTCATTTAAGAATTAAATTTGCATATATTTTTAAGATAATAAAATTCTGCTTCCCATTTTATAAATTGATGTTTGTTTTTCTATTTTTGTCATTATATCTGCATAAAGTAATTGTACATTATCGAATAGTTTCTTAACCTGATTGATAGGCATTAATGTTACAGCATTTCTTGTGTCACTGAAATGAAATACGTACGGTTGAGTTTTCCATTTACCATTTTTAGTTTTAATAATAATAAAAAAAACAAATGTAGGTGTTGATTGAATTTGCATTAGTGATATTTTATCCGGTATAAAAATATAATGTTCGTTTAAATATTTTTTTAAATATTTTTTAACATGTTTATATTTTTTAACAGATAATTTATTAGTTTTGATTTTATTAAACATAATAAGTAAAGATGAAATCATTCTTTTAGGTAATCCAGTTATTCGTTGTAAATTTGTTTTTAATTTATCAACATTTAATTTTTCATTTGAATTTGTGAAGTTAACTTGTTGATAAACTTCATTTTCTGTTAATAAGTTCCATATACTTCTTGAGTTGCTATAAACTAGATTTGTAATATTAACTTTTTGTGGTTGATCATTGTAAGACTTAGCTACGTATAATGTAACTTTTTTTATTTTACTAAAGAGTGGAATATCAAATTCTAAAAAATCATATCGATTAGAATTAATAATTGAGCATGGAGTTGTATTTTTAATATTTTCCCAATATTCTTCAAAATTACATATTATACCAATTATATAACATAATTTAAATTTCATTAACAATAATGTTATGTTTTGAAATTAATTTAAATATTTGATAACAATTAAAATTATTACAAAAACATATTCATTAAAACAGTTTTAATTATTGTCAAATATTTGTTGTATTTTCATTTTGATGTGTTATTTTAATTTTATCATTTAAATAATCCTCATTGATATATTTAATTTGATATATAAGTGCAGTAATAATAAATGATAACCAAAAAAAAGTAAATATATTAAAATTAAGGATTGATGGAAATCCTAGCATTAGTGCAGTTTCGATAAACATTTTAAATTGATCTGTTAATCCTAATATTTCACTAATTGTTAAATTCATTAAAAGAGAACCATAAATTACAAATATAAAGCAGAATAATATTTCATATAAACCTTTTAGCCATTTATATGTTATAAAAATTAAGTAAACTGTTCCTATAAATAAAATAATACTAACTAGTCTAGTTATATATTTAAAAATAAATTTTTCAAATAAATCTTCAATAATATCGTCTATGAAATCATAAATTAAATATGGTACAAACCATATTACTATTTGCTTAAATATAGGGTAAAATACTAAAAACATACTTCCAATAAATATAGATAATATTTTAAATAATATTAAAAAAATATTTACACTAATAAGGTTTTGTAATTGAATTAAATATATAAATCGACTTAAAAAAGAATAAAATAATACAATAACAAAAAAAGTAATAGGAATTCTTTCAAAATGAGTACCATGAAATGTTAACATCCAAGCTAAAAATCCATATATAAGAATTAAAATAAAATCTTTTGTAAAAAAACTTGAAAATAACCCAAGTTTTTTTACTGTATTATTATAATCATTTATCAATTCCGCATAATTTAAAGCTATTCGTAATAAATTGTTTTCAAATTCCATCCATGTACTAATACAATAAATTGATAGTACAAATTGTATCATTGTATTATAAAAGTATTGTATAATTTAAAACGTGTTTTAAGTTTATTTAGTCATAATAAATTATTAAAACTAGATTTAATATTTCTTTTTATGAAAAGTCTTTTCACAAAATTTTAATACCCATAAATGAATCAGGAAGTTATTTTAAACAAAGATTTATTTTTTAAAAGAATTAATAAAATTTTAACAATGGTTTCAAATCCACTTATAATTATGCTTGGAAAAAGAGCGGATGTTGAAGAATTTGCACTCAATTCTGCTTTGTTTAATTATTTACTTGGATTTGAATTTTCAGAAACAATAGTGATAATAAAAGAACAACCTATAATTTTTACTTCTCAAAAAAAGGCAGCTATAATAGAACAGCTTGGATCAGGGGTAAAAATTATTATAAACAATTCCAAAGAAGATCCTAATTCTCTTAATAAATTTCAAAATATGCTTAAAGAAACTTATTCAGTTGTTGATAGAAACAATATTAAAGGCCAATTTTGTAATATATTTTTAAATGGAATTAAATTTATAGAAATTACTGAAAAAATATTACAATTATTTATTATTAAGGAAGAAGATGAAATAAATATTATACACAAAGCTGGAATTATTGGAAATTATTTACTTAAAAAGGGAATTGAATTATGTAGAGATGATGAATTCACTCAAGAGCATTTAGAAAAATTTATGAATGATCCAATTAAAGATATCGATCCATCTTTAATTGAATTTTCTACAATACCACAATATTCTAATACTAGTCTTATTCTTGGTATTCGTTATAAAGGATATTGTATAGAAATAGGACGTCCATTTTTATGCGATTTAACTGAAGAATATGAAATCCAAAAATATGTGTTAGAATGTATGAAACCAGGCAAAATGTCAAATGAAATTTTACAATATGTTAATGAATTTATAGATGAAAAAGATATAGATAAAACTGTTTCTTTATTTACGATAGGATTATTACCATATGAATTAGATTTTAGGTCAAACTTTAAATTAGAAAAAAATATGTGCTTTGTGTTACGCATAGGAAATTGTTTTGTTAATACATTTATCTTATCTGATTCACCAATATTTATAACACTTAAAGATACTGCGGAAGATTATTCAATATCTCGAATGAAGTTTAGAAATAAAACTAATGAGCATGAAATTCAATTACGATTAAAAGAACATCAAAAAGAATTATTAGATAAATTAATAAATGATATGATAATATATTATAAAGAAAATGAAATAAATCCTGTAGAACAAAAAAAAGAATCTAAAATTACACCATATATATCAGATGCATCAATTCCTAGATCAAAAATAATTAACTGGGATTTAGAAAATTTTTATGTTATAATTCCTATTTTAAGTTATTCAATACCAATTCATATTTCTAATATAAAAAATGTTGCAATATCAGCTAATAATAAACTTAGAATTAATTTAAAAGAAAGTAAAGAAATAAAAGAAATTACATCACATATGCTTTATGATACAAATATTAAATCTTTTAGCATTATTACTAATAATGCTGAGGATGCACTTATTGCTATAAATGAAATGAAAAAATTATACAATAAACCTAAAATAGAAATTAAAACACAAGGAATGCTTAAAGAAAAATATAATCCATCAATATTAAGTGATCTTTTAATGAAAACAGATCAAAAGCTAATTAGTCGTAAAATAACTGGAAATTTAGAACTTCATGATAATGGATTTAAATATTTAGAAATACATTTCTTATTTAATAATATTAAAAGTATATTTTATCAATTTGGAGATTTTGAAGAAATATCTTTAATTCATTTTAATTTTAAAAAACCTATTTTAATTAATGATAAACCAACTAAAAATCTACAGTTTCATAAAAAGCAAAATAATAATTATCATGATACTACTCGAAGAGAAAGTGAAGCGATTAGTATTTTAAAACAAGAAGAAGAAGAAGAGGAAATTATAAGAATAAATAAAGAATTAAGTGACTTTATTGAAAAAATTGAAAATGAAACAATATTTAGACCACAACTTTTACAAAAGGGATTTATTGGAGTTTATCATAAAGAATCATCACCAATTTCAATTACAAGTAACTGCTTAGTATGTGTCAGTGAAACTCCATTTTTTATTTTATACCTTGATGAGGTTGAAATTATAAATTTAGAACGAGTTACATATGCTACAAAAACTTTTGATTGTGTTTTTATTTTTAAAGATAAAACAAAACATCCATTTACCATTAGTGCTATTGAAACAACAAAATTGCCATTTATTAAAACAACATTTGATTCTCTTAATTTAGTATTTATGGAAACAAAATTTAATATTAATTGGAATAATTTAATGGCAACAATTATGAAAAATCCTCTTGAATTTTATGAAACTGGTGGATGGTCTGAACTATTAATTGAAGAGCCTACTGTTGAAAGTAGTAGTATTGATTCTGATTCAGAAAGTGTTATTTCAAGTACAATTGATAGTACTGATGATGATGATGATAATTCAACAGCAATGTCAAGTGATGCATCTAGTGAAACAATAAAAACTTATGATAGTGATGATAATACAAGTTCATTTGTAGAAAGTGATTCGTCAGAATCTAATGATAATAAAAAAAAGAAAAAAACATTATAAAAATTATTAAAATTATTTTTATTAAGTATTTTTATATATTATAGAATATTTATTTATGTAGAAGAATAAAAATATACATTTGAATCTTTAGATACTGCAATTATGTTTTTGTTATCACAAACAAAATCATAAATCTCATCTTTAGTTACAAAACTTCCTACTATTTTATTAATTGATGGTTTATAAATTTTGATTAATTTATCTTTTCCAGCGGATATAACTAAATCATTTAGATATTTTATTTTATAAACATAATTAACATGATAAATAGTAAATAAGCATTTACCTGTTTGTAAACTCCATCCTTTAATAGTTTTATCGAAAGATCCTGAAATAAAATTAAATCCATTAGGATCAATATCTATAGATATAACTTCTCTTTTGTGGCTCATTCTAAATAATACTTTAGTATCTTTATAAATAGTTATATGTCCTAAGTCATCACCAATTATAATAATATCTTGAAATATTCCAATACAATTAATACGAACATTATTTGTATCTAAATCTTTAATATAATCCATAGTATTTATTGTAAACAACTTGACTATACCATTAGTATAACTTGAAACAATCATATGGTTATTTATTTTAATATCAGAAACTGAACTACTATATCTATAAATATTTATTATTTCTACATTTTTATTATTAACTATTTTAAAATAACATAATAAACCGTCTCTTCCTCCTGCTACAAATTCATTTTGTTTAATTTTTACTAATTTTATTACTCCTTTTGTAAATTTATGGATTGATTGAACAAAATTGCCACTTTTATCATATACACATATTATTGAATCCATACCTCCTGAAATAATATAATCTTCTAATTCTTCTAATACTAATACCCAGTGATTATGATTTATATTTACTTTAAATTGTGTTTTTGTTATTAAATCCCAAAATCGTACTGTTTTATCAGAACCCGCAGTAACAAAATACTTTATTTTATTATATGTAAATTGAAGACATGCTAATACTGGACCAGTATGTCCTGAAATAATACTAGAACAAAACTCAGCTGGTTGACTACCTTTTTCTTTTACAATATTTATAATTTTAACATTCTCTGTATTAATATCATCTTTAAGTGATTTTTCAAGTGATTCAATTATCATATTTCCATTAACATATAAAAAAACATTATCATCTAAATTAAATAAAATTTTCAATTGAGAACTAGTTATATTTGCAGGAACTTGAATGATTTGATCTGATTGATCACATCGAACTAAAAATTGATTCATTAAAAAAAACTTTCTATGGGATTAAAAATTTTATTTTATAATAGATTTTAAAATTTCAATTCCTTTAATTCTGCAATTATTAGATATATAATAAAAACCTCTAAACATTTCCATTTTATTATCTTCTGAAATTAATAAGAACGGATTTTTCTCAAATGATTTAAATTCATTTATAAAATTTTTAAATAGTTCATTTTCAGTATTATGTAATAAAATACATAAAATTTGATTTTCTAATATGAATTCATCATTAAAAATATTATATATTTCAAATGCTTTTTGAAATTCATTAATTTCAAAAAATTTTTTAGCACAATATATATATATGTATTTAATACACCAATTATCTTTATTTTGATTTAAAAACGGGATATCAATTATTTCTATAGCTTTATGAACTTCATTCATTTTTATATACCACACAGGAAGAAATAATACACTATCAACCTTACTATCAATTTGATTCATCTTGATTAATTTGACATATAGTTCTTCATTTTTTAAATTATACATATTTAAAATAGGAAGTAACTCATTATAGTTTATTTGATTATCATTACATTCCATTAATATTTTATCAATATATTTATCTATATTTTTTTTAAAATATATTGCTTTACTATCAGTATATGAATACAATTTTTTAAGAATATTAAGCATCTTATTATAATCATTTTTTTTATGTAATAAAAAATATTGTGGAATTAATGTATCGCTTGTTTCTGGTAGAGCTTCTAATTGAATCAATCTTTCTTCATCATTTTCTATTTCTAATATTTCATCAAGGGATTTAGTCTTTTTATTATTTTTTAGACAATGTTCGATAGTATTAATGCTAGTTTCAAATGTATTATTTTGTATTTTTTTAAATTCATTTATTAAATTTAGTCCTTGAGTACTTTTAACAATACTAAACAAATCATCAACTACAGTTGGTATAGAATTATTTTTTGTAGAAAATTTCTTTAAATCTTGAATAAATTTTTTAAAGTTTTTATCAAATATTTTAGGATTTTTTTCTGACATTAATTCAGTAGCTTTATTTTCAATTTCTTTATATTTTTTATCTTTTTTAGAAAGTATCTCAGTTGAATTAATTTCATTAAAGTATTTTTCAGCTTTTTTTTCATCAATTCGATTATTTTCATTATTTAAATTTGCAAAAAATCTTGCCATATAAACCAGGGTAACTAATATTTTAAATGTAAAAATAAATTATTTAATTTAATAATGATGACGATCGGGGTTATAGCTCAGTGGTAGAGCATTCGCTTTGCATGCGAGGGGTCATGGGTTCGATCCCTGTTAACTCCATTTTTTCTTAAACATTATTATAAAATAAAAAATTTATTTATCAAATTAACTAATATCAAATTCTAATTTTATATTCATTCCCATTAATTCAGCTAATAAATATTTAAAAACATATGGCATTTTAATATTTTTTGATTTTATTCCACCACATTTACATTTATCATTTGGAGAACTAAATAAAATATTTTTACATTTATAACAATATTTAAAAGTTGTTTTATCTGAACAATCTAAAAGTCTATCTTTTATTAACGCACTAGATCCATGTGCTATTAAAGTATCTTTTTCCATCTCTCCAAATCTAATTCCGCCTTTATTTTTTCTACCTTTAACTGGCTGTTGAGTAGTACTAGTTATTGCTCCCCCAACTCTAACCTGAAATTTATCAGACACCATATGTTTAAGACGCTGATAATATACTAGTCCTATAAAAATATCAGTTTTCAACTCTGTACCAAATACCCCTGAATACATTGGTTCATTACCATAATAATTAAATCCACATTCCTTTAATTTTCTATTAACATTAAATTTATTTACATTAATATCATTATTTTTATTATCAATAAATGGATCGCCATTCACAAACTTTCCAAATTTTACAGCTGCTTTTCCACACATGGATTCAATTAGCATACCAATTGTCATTCTACTAGGAAATGCATGTGGATTAATAATAATGTCAGGTTTTAAGCCATTTTCAGTAAATGGCATGTCAATTTCGTTCCATAAAAATGCACAAATACCTTTTTGACCATGTCTACTACAAAATTTATCTCCTACAACTGGATTTCTTAATATTCTAAAAGTAAATATTACAGTTAAATTACTTTTTTCAGTTAAACTATTTTGAAATACTCGAATTTTTTCAACAGATCCCATTTCAATATTTTCATATCTTACTGTTTTTATTATTCCATCTGATTTATACGTTAACATTATATCATTTGTTTTTAATTTAGATTCTATGGGTGGAGTATATAAAATTTTATCATTTTTTTCTAAAATAATTTTTTCATTCATTGTAATAAATCCATTGAACATTCCTCTATTTACAGATGCCTTATTTAAAATAATAGCATCTTCCATATCATATCCTGTATAACTTAATACAGCTACTATACAATTAAATCCTAATGGAAATTTTTCGAAACATTGATAATTACTAGTTTGAATTAAAGGATGTTGCAAATAATTAATATTATATAATTTATTATCAACTCTATACTTTATATTGTAAGCAGCAATTCCCATTGCTTGCTTTGCCATTTGACATTGATATATATTACGAGGAGAGGGATTGTGATCAGCAAACGGAATAGTATTTGCTATAATACTAAACATGTTATTTATATCTATTTCTTTATAATTAAAAGTATTATGTACTGTTCCTAATTCTATATCTAAAAAAACTTGTTCTTTAATTCCTATATATTCTTCCATATTATTTTTTTTATTCCATACTTTTCTAATTAATAAACTTATATCATCACAGATAGAAATTATTTCATCAATTTTATCACCAAACTCATAAACAATTTCTATTTTCAATTTATTTTTACATCTATAATTTCTTAAATCTTCAATCAATTTTATAGGATAACTAGTATAACCCATAATTTTTCCATTATAAAAAACAGGTATCATATTATTATTTAAATTGATAAATGAAACACCAAGTTGATATAAAATATTTGTATCAAATATATATTTAGAACATGTTATTTCACATTGTTGCGTTAAATAAGTTAATAATCCACATAAACTTCCATCAGGAGTATTAATTGGACAAAAAAATCCAAAACTTTCTGGTTGTAATTTTCGAACACTAGTAATTTTTAAATTAGAAAAAAATGCACCTCGAGACACTGAGTAAAAATGAGATGCAAATTCCCAATAATTAATCCTTCCACTTAAAAATGTAAATCCAGATGTTTGCAATAAATCCGAACAATTATATGTTGTAATGTTACCTGTTGATAAAAATTTATGAATTTTCGTTCCGATATCTAAATCTGTTTTTTTAAATAGTTCTACAATTTTTTTTATCATAAATTCATCGGAAAATAATTTATCAAATTCAACAGTATCATTAATATTTGTTGATCTATTAGTTGACATAATATCATCTATTTGTGTTGTTATTTCTGTATCAGTTTCATTGTCTCTATATTGATTTTTTACTTCTCTTTTAAATAATTTTATTATTCTAGTTTTAAATAATTTTTTAAATTCCTCAAGATGTTCTTTTATACACAATGGTATTAATTGAGCTTCTGTATATAATTCATGATTAGATGATAAATCTATATCTTCAGATTTTATTTTTCCATCAATCAACTTAAATAACTTATTAATACTGTATATTAATAAATTAAATTTATCAATATGTGATGATAAATGAATTAATATTCTATTAAGTAATTCTTCCCCTGCCTCTTTATAATTATCTATTCTAAAAATATGTTTAAAACGACTTCCTAAATAATTTAAACATTCATCTTTATTGTAATACGGTTGATTAGATTTCGTTAAAAATTCAATAGCACGTGGATTTTGATTTAATAAAGTATAAATTTCTTTGTCTGTTGTATTTTGTAAAGCTCTAAGTATATAAATAATTGGAATCAAATATCCTTTATGATAAAAAAAACATTTTAAAAAAATATTTCCATCAACTGTATGTACTATTTCATTTTTTTGACCAATTTCATCATCACATACACTTCTTATAGAACAGGAATATCCACTAAATATAGAATTTTTAGGTTTACTATAAATTCCAAATACCCAATTTCGTTTATGAGCAATATGAAATCGTATGATTCTATCATATCCATTAATTATAAAAAATCCTCCATGCTCATTTGGATCTTCTTTAACAGCTAGTAACTCTTGATCTGTCATCATAGATAAATGACACATATTACTTTTAACCATAATTGGAAGTTGTCCAGCCCCCCTATAATCATGAAATAATATTTCATTTTTGTACATCAATTTTATTTTAACAAATATATTTCCAGAATATGTTCTTCCTGTTTCTCGACATTCATGTGGCAATAATCGATTATCTACATCTTGTTTTAATACATACGGTTTATGTATTTCTATTTTTTCAAAGTTCATAACAAATCCTTTAAATATTAATGGTTTAGTTTTATATACAACATTTTTAATTATATTTTTTTCAAATAATGCATTAAATTGTTGAATATGAAGATTTCCAATATTTTTACTTTCCATAATAGTACAATCGGCTTAAAAATTTTATTATAAAAAAATAAATTATAATTAATTTTATTATAATGTGTAAAAATAAAATAACAAAAATAAATGTAAAGATAACAATACCCTTAATGTTTGATATACAAGAATTTCAGGCCATTTTAAATGATACAAAAAAATTATTTAATGAAGATTTAACTGATGAAGAAATTATTTTTAAAATTAGAAATAATTTAAAAATTTTAACAATTTTATGTAATAAAATTGAAGATAAAAATATAAAACATGATGCTTTAAAAACATTAAATGAAATAAAAAATAATGATAAAATAAAATTGTATAACTTAATTGATATTAATCCAATTTCTAAATCTAATTCTATTCAAAATGTAAATTATAAAGAACATAAATTAAGTATTAATAATTTAATTGAACAAGAAATGTTAGAAAATAGTATAAAATTACATAATATGACAAAAAAATTCAATGAAACTTTAGATAAAGATAAAAAAATAATTGACAAAACAAAACAATCATATATGAGAAATTTTAAAGAATCATCAAAAAATACATTAAATATTTCAACATCTGAATCTTTTAATACATTATATTATTTTTTTCAAACACTAGTTATTGTAATATTAATGTTTTTAATAATAAAAATAATCTAAATAAATACTATTTAATATAAGAATATTATTTACTTAATTATAGATATTATTATATATTTATAAATATTATAAAATTAACATTGTATTTAATAAAACATTTTTTACATTTTTAAAAGCTTATTCAAATGGAACTTTATATTCTTCATACTCTGGTGAATCGTTCATATTGATATTAGAATTTGAAGATAAAACTGGAGAATATTGTTGAAAAGTAACATTATTGTTTAGATTATTATATTTAGAATCTTTATTATATTTGTTATTATAATTATTATGCTTATATAAATAATTATTATTTTCATATCGTTGATAATACTGATTTTTGTTATTATATTTATTATAATTACCATTATAGTTATTATAGTTATATCGAGTATTTCTAAATCCATCCCCTGATCTTTTATTAATATTATAATTATTTATAGACCGTGCATTATTTTGAAATCCTTCAATTTCTTTGTATACTTTTCGTGGATTGAAAGGAATACAAAATGCTTTATAACTTGGAAATTCAAATCCTATTACATCTTTATTTTCTTTACATAATTGAATATAATTTTCAACCCATATGTGATCAGCTTCCTGTTCTAATGATGGATTTTGAATAACTCCTAATCGTAATTCGTCTGGATGAATATCAAAATTACTAGATGCATAAATATAATATGGATGAGTTTGTGGATGAATAAACTTACAATTTACATGAGAACAATAACCTTCCATCCAATCTCTGCAAATAGGCAACTTATTTGAATATCTATTATCATACGATTCATTTTTATAATTAATTCGATTATCATAATTTCTATCGTTATATATATTCCTTCTTTGCATTTAATAAAATTTTAAGGGTAAAAATAAAAATTTAAACTTTTATAATTACCCTGATTTTATGAGAGAATAATTTTATTTTATAATAAAATTATAGTTATAAAATCAATAATATAATTAAAATTTTTTATTTAATTAAAAATTATTTAAAAATTTTCTTTAATTTTCATCCTAGCATAATATTGTCAGGAATTAATTTTATAATTAATTCTGTTACATTATGTTATAATAATTCATTTACATACTTAGTAGTTTATTAATATAAACTGCTTATTACGGTATGAAATATTTTAATTAAAAAGTAAATAGTTTTTAATTAGAACATATTTATTCAAGATAATTTTGTTTTAACTTTTTCCAATTAATTTCAAAGTTTTTATCCCAATATTCTACTAATATTTTAGATATATCATCTATATCATTATAAATATCTAACACAGTTTTATTTTTATTTTTAAACATTTATAATTAGGAAAAAAAATTCTTTATTTATTTTTATTATTTTTTTGGTTATTATTTTTATTTTTATATAACAAATTTTCTTTTATAGTAAATTTATAAAATGGTGATATACTAAGTAATAATCCAAGATGCCATAAAAAATGGGAATCTATAAAATATAAAATAGGTGGAATATCAGACAATTCAAATAATCCGCTTAATATAGATAAATTTGTTAGCATTATAACTTTTTTCCTCAACGTTTTATTTTTATAATACATAGTTGTTAAAATATAATTAATTTGAAGTAATAATACAAGTATAACACAAAAAATTTTATTATGATGATAATTAAAATCAATAATCAACATCTTATAAAGGTATAAAATAAAGATTCCAACAAATAATGTGAATACAATATAATTAAGAATATAATAAAGCTCAGGAAAATATATTAATATATTTCGTTGTGTTGATACCAATGTATTTGCTAATATGGACCCTACCGCTGTAAAATAATCCATATTTCTGGTTACTATATTTTCATGAAGATGAAATAAAAAAGATGACATAAATGCTAAGTTCATGATAATATAATGCAACCAATATTCTCTTTTTAACGGACTATTTTTTGAGTACTTTTTAACTTTATAAATATAACTTAAATTAATTATAAGTTTAATTAAAGCACATAAAGAACTCATAAATTCTGTACATCCTAAAAAATATTTAAATACCCATCTTCCTTTAAATTTACAATTATAATATGAAAGTTTTCTCAAACAATCTAAATTACAATATAAATGAGCTTTATCAAGTTTAGTTCTGCCTAATAATTTATCAAGAGGTGTGAGTTTTTGATAATTAATTATATTTTTAACACATAAATCACTACAGTATACAAGATTGTTAAATTGACTATCAGATCCTCGCATAAAGTTTTTATCAAATCCTATATTATTTTCCAAAATATCTTTAGTTATCATAAATGGGGGTTAGTATTTATTTATTTTTTGCAATATAAAAATAATATCGTTTTTATTAAATTTCATCTATTAATTGAATATTATCTCCTGTTCCTATACTTATATTTTTACCTAATATAATACTATCACTCACACCACTAACTATATCTTCTCTTTCTTCTATGGCAGCTTGAAACAAAAAATCAGATGTTTGTTCGAATGAAGCTAACATTAATGTGCTATTAAAGTATTTCATACCAAAACGTGTAATTCCCAACACATGTCCTTTAATTGTCATTGTGTCAGCTAATAACATAAGATGCCTACAATTTATTTGTATTCCATGTGTTCCTATAGTATATTCAATTTCATTAATAATTGATTCTCTAGCTGCTTCTATTCCTAATGTAGATTCAATTTCAAGAATATTATTAGTTATAGTAGTATATGGATTTATAAATTCTGTTCCCAAGATATATGGTAAATCTCCAAATGAAGATGATATCAATAATTCATAATCTTTATTTGTTTCTTTAATAAATACTTTATTTGTATTAGTTAAACCACTAATTTGTGTATTTAAAAGAGTGTTTTTTATTTTAAATATATTGAATCCATTATATTTAAATTTATATTGATAATAATTGTAATTATTATCATTAAATAAATCCATGGTATTATATTTTGATGATAAAGGTTTAATACATAATTTTTCTAAAATATTATTATCTAAGGGGGAAATACATGTAAATATCATGTTAAAAATATTTTTTTCATGTGTATACATTATATCAATTCTTTTAATAATATCTTTAAGTACATTTTTTTCTAATTTTTGTTTAATTATATATGCATCTTGCAAATTTACATTTTTAACAGAAATTATTGGAGTTGAAATTGTAGATGTTGCATTAATAATTTCTTTAATACGTGGAACACCAAGAGTAATATTCATTGATGCTACTCCTGCAAAATGGAATGTTTTAAGTGTCATTTGTGTCCCTGGTTCACCAATAGATTGTGCAGCAATAGCACCAACAGCGGTTCCAGGTTCAATAAATTTTTTAATAGTAGCGTCAATCGTTTCATCATATAATGGGTCTTTACCATCTTCACCATATAAAAATTGTATTATTTCATTATTATTGTTTCTTACAGTATTATCATATTTAATACTTAAATTCTCTAATGCCTTCATCAATCTTCTTTGCATATATCCTGTATCAGCCGTTTTAACAGCTGTATCTACTAATCCTTCTCGACCACTAATTGCATGAAAAAAAAATTCATATGCTTTTAATCCTGTAAAAAAAGAATTATATACAAATCCTCTATAAATAACATCATCTAATATATTTGGTATATTTTTCATATGAGGAATTGTTTTATCATACATTCCATTTTGTATACGCATTCCGGAAACAATTTGTTGTCCTACACATGTTATCATTTGACTAATATTGATTTTACTTCCTTTACTTCCACATTCAGCCATAACAACAGCTGCATTATTTTTATTAAGTGTATTTAAACACATTGTTCCACAATCTTCTCTAATTTTATTTAATAAGCTTGTTTTTTTTAATTCTGACCTATTAGTTCCATCATTACATTCTTTATTAATAGTATTAATAATATTTAACACTAATTTATTTTTAGATCTATTTAAATCATTTGATAAGTATATATCATCTAACCCAATACTAAAACCATATTCCATTATGTATCTAATACTAATTTTACTAATATTATTAATAAATTTAATACATTCTACCTTGCTTTGTTTAAAAAGTTGATATATTAAACTACTATTTTTATTTTCAGCACCAATAATAGATTTATCAATAGTTCCTTGTATAAGTTTACTGTTTTTAATTATAACATCTTCTTTATTTCGATTTTTAGTTTTTAAATTAATTGTAACACTATGTAGCATGTAATCAACAATTTGCTTTCCAGTAAATAATTTAATTACAGTTGTAGATTTATATATAATTATAGAAGGTTCAATTATTATTCGTTTACCATGATGAATATGACCAATAAGTTTATAAAATTCAGTTTTTGTTAAAATTTTATTTTTATTTGTTAATAAATACATTCCTGTAATAAAATCTTGAGTCGGTGAAATAAGTGGTTCATTATTTTTTATAGTACAAATATTTTTTTTTATACTCATTAATTCAATAGCTTCGGCTTGAGCAGCTTTAGTTTGTGGAAGATGAATATTCATTTCATCACCATCAAAATCAGCATTATATGGATTACATACACTTTCATTAAATTGAAATGTTCTATTATCATGTAATTTAACTCGATGAGCCATTATACTCATACGATGTAATGATGGTTGTCTATTAAATAATACTATATCACCATCTTTTATATGACGTTCCACTATATCTCCCACTTTTAACTCTCTTACTTTTGGATTTTTAGCAAATTTCAAAAAAATTTTAAATGTTCTAGTTTCATTATTTCGTACTTCATTTCTAATAATATAATTTGCACCATCAATACAATTTGGACCATTATTAACTAATATTTGTAATTCTTCCTTATTAAAAACAGTAACTTTCTCTGGAATGGTCATTTCAAGAGCAATTTCTTTAGGAACACCAATTTGTTCAATACTTAAATTTGGATTAGGAGATATAACAGTTCTTCCTGAAAAATCAACACGTTTTCCGCTTAGATTCATTCTAAATCGACCTTGTTTTCCTTTTAATCGGTGTATAATAGATTTAATATTATCATTATTAGTGATATGAATTGATGGAAGATCAGAATTAATATATAATGCTATTTGCAATTGAAGATAATCCCAATCTTCATTTAAAGTAATTAAACTAGCTCCTTTTTTAATATTTTCTTCTATAAGATTATTACAATTAATTATTTCTGAAAGTTTGACTGTTAAATCATCTTCATTAAATCCATCTTGATCCATATCTACTGATGGTCTTATACAGCATGGTGGAACTAATAAAGTTTTTATTAATAAATTACATGGATTTTTACAACTTAAAAATAATAAATCAGTTTCTTTTATATTTTTAAATAAATTATATACAGTTAACGGATTTAATTCATCACAAAATAATTTATTATCTTCCATGTCTTTTGATTTACTATTAGATTTACGAAACTCATGAAAAATCCTAAATCCATTTGATTTTTTAACAATACCATTAAAACTATTACAATTATAACATTGTTTTATTTTTTTACATTGATCTACTATATTTACTTTAGATTTAATATCATGTTTATTTTTAATTAATAATGAACTGCATTTTTTGCAAATAGAATTTAAAATATTTATAATTTCTTTAAATAAAGCAATATGAAAAACAGGTAATATCAAGCTGATTTTTCCCCAATGGCCAGGACAATCTTGTAATCCTTGATTACAAGTTTCACATTTAGTTTCCGTTGTACTAACTCCTAATTTAAGGTCTAATGGCCCTCCTGAAAATGGTTTTCGTGTTTCAATATCATGAATTGTTTTGTGTTTAATTTCAGTTAAGGCTAAATTATTTATGTTACTATCTGATAGTAACATAAATTTTATTCTATCTATTTTTTTAGTAGTTTTCATGTAGATGGATAACAGTACAAAAAATTTTAAAATAACTTATTAACTAAATAGTAAAATTATATAATTATTTATTTAAATTTCTATACTTTGACATCAAATAAATATTATCAAATTTGCCAATTGCAGATTTATTATAATGTCCTGCTATAAAAATACATTTATCTTGATTATTAGAAAATATTTCATCTAAATATATTTCATTAGGACTATCAAATAAAAGAATATCTCGAGATTTTGCAAGTACTAAAGCTCTAAATAAGCATATTCCTTCTTTAATTTTAAATGGACAATTAATATTATAAAATATTTTTTTATTAATTAAATCATCTTTTAATAAAGCATTTAGCATAGGATAGTTATTTAATATATTTAATATCATATTTATATCACACTCTTTCATAAATAAATTGTCCAATATAGTTCCATCTATAATCTTTAAATCTTGACATAAATAACTAACAACTGAATTAAATTCATCATAAGTTAAAAAAAGTGTTTCACTAATGTTATCTGATAAATTTTCTAAAAATACTTGTGATTTATAATCATTTAATAGATATAAAAATAATTTAGATTTTCCTATTCCATTTGTACCACATATTAATGTTTTTTCTCCTTTAAAAGCAGATATTTTGTGAATTTTTTTATCTATTTCAACCTCGATTATAACTCGATATTTTTCTGGTAAAATATAAATAGTTGATGAATTACTCGATGTTTCTTTAATAAGAAATGAATTTCGTTGAAGATTATTAAGTATCATTTAAGGGTAATGTTTAATAAAACTTATTTATTTCATATATAATGATAATTCTTTATCAAAAATCATTCCTCGTTCTTTTTCAAGTTGAATAATAGAATCAATTATACCCTTAAATAAATATAGAATTGGAAATGTTTTAACATATGTATTAAAATGTTGAAATTTAAATAATTGTATGAAATTAAAATACATTAATGCTATAATAAATGTTGTAAATAAATTAAAAACAGTATCTACTATAATTTGGTATAATTCTGAAATATTTTTATATTTGATATACAAAATATCATTGTTTAGATTTTCTTGGATAATTTTTTTCATATCTAATATTTTATTAACTTCTTTAACAAATAATCGTTCAATTTTACAATATTTTTTAGTAACTTTAATCCTAATTTTAAAAATTAGCCATTTACTGATTAATATGATTAAAAATCCAATTATTATCATAAAAATATAGTATATATTATATGTAAATATAGCAGTTTTTGTATAAATTAATATTTTTATTTCTCTAATTAAACTAGATATATTTAGTATTAAATCAAAAAATAATATACATGATGCATCCTCTATTACTTTTTTATGTTTATTAACAATATAATATAGCTCAGCTGAATTGTAAGATTTAATGGGGTCTTTAGTAGTTCTTTTTGTTATAAGAGATGTATTTTGTAAAATATAAAATTGTAAAATATTTCCTATATGATCACTATAAAGTGGACGGATTATACGACCAATCCCATTAATAAGTAACATTATTAATGTAAATCCTATTATTGTATTTATAGATTCATTTGAAGAATAATTATCAATAAATTGATAAAAATAATATGTAAGAGACTGGTCTATTAATAATAACATTATAAATCCGATAACTATACATTGATTAGATGTAGATTTAAATATAAATACAAATAAAAAATACATAAATTTAGATATATTAATAGGTTTGATGGGTTTAGTATTTCCAGGAATTGGAATTGCTTTTTTTTTTAGTATTTTTTTAAATTTAAAACTTTTTACCGAATTTGACGTGACTTTTTTATCAGCTGATAATAAATTGTGTGTTTTTTTAATTTTTGATTCTTTAGATTTTTTTTTAATGTATTGAGTTGATATTGATATATTTTCATCAATATTATTATTTATTGACGGTGGATCGTCTTCTTCATTAGAATTAATTATATTTTCATCCATAAAAAATTAAAAGGGTTAATAAAAAAAAATAAATAATCTATCTTAATATTTACCAAATTTATTTAATTCTTTTAATTTAGGTATACGCTTTAATTGGAACATGGTTTTAATATCATCTAAATTCAGTTGTTTGTAGTCAAGAATATAATTTAAAATATGTTCTTTATAAAATCTAATATAACTAACAAATGCTTTAGTTCCTAATTTAATAAGTTTATCATTATTTTCAAAAATGATATTATCAATAGTATAATTTCTATATTCTTGAATTGATAAAGTTTTATATTTCAGTGTAATATATTTTAAATAATGTAACTCATTATTTAATAAAAAAATAATACTTTCTCCAGTTTTACCATTTCTTCCTGTTCTGCCACTACGATGTAGAATATTTGATGGATCTTTAGGAATATCAAAATGAATTACTAAATCTATATTTTGAAAATCAAATCCTCTAGCACTTAAATCTGTTGTAAGTAACAAATATTTGGTAGATTGCTTAATTTGTTCAAATATAATATTTCTATTGTATTGCTTTATTTTTCCATGCAATAACATTAAATTGTTAGTATCATAATTAGGATGTATATCTTTGTTGTTAGTAAAATATATTACTAACAAATTATAAAAATAGTCTACTTCTGCACATGTTCCAAAAAAAACAATTACTTTTGAATGATTTTTAAATAATTCTAAGCATGTATCAATTTTTTTATCAGCATGTAAAATCATATAAAAAATATTTAATGTAACTGGTTTAATATTATTCATAATAATTTCATTATAATTTCCAATTTTTTGAGCTTCTAATTGAACTTCTTCACTAATAGTCGCTGAATAAAATTGAACTATTTCTGTTTTTTTTGCTATTTGATCTAAAATTATTCTAGCATAATTATCAAATCCTTGAAATAATAATTTATCAGCTTCATCTAATATTAAATATTTTAATTTATGAAATCGATTTGGATTTTTTTTTATTAAAAATAACATTCTACCAGGTGTTCCAATAATTAAATGTGAATTAATACTGGAATCATCTAGCTCATTATCTCCACCTACAAATATTTTATTATCTATTTGAAACATATTTGCTAATTCATGAATTTGTAAGCACAACTCTCTTGTTGGAACTAAAATTATACCATAAATTTTCTTTACATTATTATACCGTACTGTTAAATTATTTAAAATATTAGCTAATGGAATTAAATAGCATAACGTTTTTCCAGACCCAGTTGGAGCTTGAACTATTAAATTTTTATTATTATAATCTAATACTTTTTGCTGGATTTCTGTCATAATTATAAAATTATGTTCTTTTAAAATATTTTTAACTTTTGATGTAATATATTTATTATTTTCAAATTTCATTAAAAATTATTGAAGTGTGGTACCCTATATGAATGAAAAAAAAATAGAAAAAGTATCAATAACATATGTATTAAATGAATTATTAAACAAAGGTAAAATTAAAATAAATGAGCATATATTTAATATAAAAACAAGTTATTTAAATACATTTTTAAATCCTCTTAATTACAAAGCTATTAAATGGAATGACACAAATGAAATATATCTTGTAAATTGTCCTGATAATATAGAAGAATGTGAAGAAAAATGGTTAGTAATTCCACCATGGTCAGAATCAAAAACAAGAAATGCAAAAATTAATTTAGAAAAATTGGCTAAGGGGGAAATTCATAACAATACTGATTTAGACATGTTATTCAAATATGGATATATTGAAAAGAAAAATAATAAGATCATATTATCTCCTAGATCATTATTAAATTATGGATCTTATTTTAAAGAATTAAATGAACAAAGATTTCATAACTGTAAAGTATGTAATAATATTGTTGATGGAAGTAATATTCATAAATCTTGTAAAAAAATATTATTAAATAAATAAAACTTTTTTAAATATATTATTCTAGCCCCTTTCTAATAATGAAAATAAGACGTACTATTTTATTTTTATTGATATGCATTGCAATTATTATGATTTATTTATTATTAAAAAATATTTTTGAAATGATATCTATAAAAACATATAAAGATACAGTTTTTAAAGAAATTCCACAGCCTTCTAAACCCTCTCCATCACAAGATAATATATGGAATATAATAAAAACAATTGATATTGATGAAATTTTTATTACAAATAAAGATGAATTTTTAAAAGAAATTGATAATATTATTAAAACAGATAATATATATAAAAAATACAGAGATGAAATTATATTATTTAATGTTAATTTATTGTATTTTTGGAAAAATAACAAGAAGCATCCACAATATAATTTATCTATGAAAGAATATCAATCGCAAAATAATCTTAATAAGAAAATTTGGGAAGAACATTTATTTATACAATATCTATTTCCAACAGATACACCATCACGAGCACAGCCAACCCAATGTGCAACTCTGTTTACATTAAAAAAAATAGTTGAACATTGTACTATAAAATATGTTCAGGACAATTTAACCTTAAATGCATATATGTTTTTAAAATATTTAGATGATTTTTTCGTTAAATCAAAATTAATACATTCAAATTGGCAATTTATTCATAGTAATGATCATAATATATTAAGAATTTCTCGTGTAATCTTATCATTAAGATTATTCAACGTTAAATCTACAGTAACTACAGTATGCGATTATTGTATAAAGTGTATTAAACAATCTGGTAATTCTACGTGTTTTGCATATGACAATTATTGGAAAAAATATAGTAAAATACCCCCTTTATCCATTATACCCATTGATGCACATAGCTCAAATGAAAGTGATAAAGAATAAATGAAAATAAATTTAACAACAATATTATCACAAAAAGCCTCAGGCGGGAATTGAACCCGCGACCTTTCGTTTACTAAACGAATGTTCTAACCACTGAACTACAAAGGCATTATTTTTATAACTCATTATAAAAATAATTTTTATTTATATCATTGTTTTATATACAAGTAAATAATGCAAAATATTTTAATACTTACAATAATAGTATCAATATTAATATTAATGTTTTCACCCATAAATATCTTGGGGTATGTAGGTATTTTTTTGTGTTTTTCACTAAGTGCTCTTGGAACTGCACAAGGAATATTTACTATTGCTACATATGTTAGTGGTAGCTCAATAAAAGCCCCAAATGTAGGAACTAAATCTATTTTAGGTACGATTGTGTGCGAAGCTAATTTTCTTACTGGTATTATTACATGTGTTATGCTTAACAACACTATGGTAAATACATTACCGATAAAAGCACATTATATATATTTTTGTTCTGGATTGTTTGTAGGAATATGTAATTATTTTTCATCAGTAGCCACTGGTTTATTATGTGGAATTATTTCTATGATTGATGCCAAGAATAGTTTATTATTTTATAAAATTGTAGTATTAGAAATCATTCCGGCAAGTATTGGATTGATTGGTTTTATAATTGGAATAATATTGAATTCTAAGGCACCATCATTTAAAGATTTAAATCAATAATAAGTTAAAAATATATATCAACTATTATTTTTTCGAAAAATTTATCCCTAATGTCATCTGATGAAACTCGAGTATTTATAATAAATTTTGCAGAACAGCATAAAGATGGAATAATACAATCTCAAGATCTAATTTCATATTTACAATCAATTATGAAGGTAAGAAATAGTAAAATAATTGCTAGTAGAGAATTAAAATTCAATGATAAAACAACTTCTATAGAAGTTACATCAAAAATAACAGATTTAAAGAAGAAAGATATGAAACAATATGTTAGACGATTTTTGAGAAATAAATCTCTTAAAAATTATATACGAGTAACTGGAAACAAAGTTGATGGGATCTCTATGGAATATATTAATCCAGTTGACAACGATATTGAAGAATAAAACAAATTCTTATAAATTAAATAAATTTTATTATTAAATGATTATGGAGTACATCGGGATGGTTTTCTACAATATAAAGTAGACTGATCTACACTTTTATATTTCATTAAACACATTAGTAAACGCTCAAATCCCAATCCATATCCACCATGTGGACTCGGACCAAACTTTGCCATATCTAAATACCAACTATATGGTTCATGATCAATATTTTCACGTTTAAACCCATCTAATAAATTATTATAATTTGTTTCTCTCATTGATCCGCCTACTATTTCACCTATTCCTGGAAATAATAAATCACAAGTTTGTGTACCATATTGATCTTTAGCTACATAAAATGGCTTATGATCAGTTGGAAATCGAATTAAAAAAATTGGTTGATTTTCACCATATGTTTCTAATAAAAACTTTTCAGATGCATCAGAAATATCATCTCCTAGTTGATAATCTGTATTATCTGTTTTTTTATGATTTTGAGCTATTAAAAAATGAATAGCATCTTCATATGTGATTTTTTTAAATTGTTTATCAGATAATACAACAGGTTGAAAATCATTATCTATTTTTTTAATTTCCGGTAAAATGTTTTTATAAAATTCATTTATTGTAGATCGAATTAATTGTTCAATTTGGTCCATAAGTTCATCAAATTGTATATCTACTAGTTCAGCTTCTACGTGCGTAAATTCACTCAAGTGTCTACTTGTTTTACTTTTTTCAGCACGGTAAGATGGCATAATACAAAATGCTTTACCAACTACAGGAGCTACCGTTTCTAAATAAAGTTGCGAAGATTGGGTTAAATATGCAGGCTGATCATAATAATTAAGTTTAAATAGTGTAGCACCACCTTCAACTTGTGTTTGTACTAACGTAGGTGGTGTTATTTCTGTATAGTTATTATTCCAGTAAAATTTACGCAAAGCCTTCAGTAATTCATTTTGTGCTTGTAAAAAAAGTATCCTATGCGGCATTCTTAATCCTAAGTGAGCCAAATCTAATAAAGTATCTTTATCAGAATGAATATTTAATGGAAAAGATGGTGCTTGGTATCCGCCGTAAACTTCATATGCTTGAACATCAATTTCAAATTGATGTTCATCATTTCCATTATTTGGTTTCATAATTCCCCAAACTTTTAATGTAGTATGATCAGTTAATTTCACTTTTGTTGGAATAACACATTTAATAATTTTAAATTGACTTATTAATTCAATAAATGATATTTTACTACTACTACGGCTACTTTTTACCCACCCAAAAAAACATATTTTTTGGTTCATTAAAGAATCATCTATGTCAAATAATCGAATATATCTGTAGGTGTTTTTATCTATATTTTCTTTTGACCAATTTTCAACTTTAATTCTTTCTAATTCTTTTTTAATTGATTTTAAATTATCTTGTAATTTCATTATAAGAGGGATAAAAAAATTAAATTTATTTTAAATACTAAAAAAATTATTAAATATTATTTAATTATTATCCTTTGATCTTATTGAAAATTTTATACTTCCTGAACATCAGGATTACCACCGGCTGGGTTTTCATTTGATCCAGGATTTGCAGAACCACCATTAGTAGCTATTATTTTTTGTATTTCTTGTGTTGTTGTATCTATTCTATTATTTATTTCTTCTATAGGCAATCCCATAAATGCATCTTCACCAATCCACTTTAACTCCTGATCAATTTTATTTTTAACTTGTTTTTTGGTATCTTCTGATGCAGTTGACTTTTCCAAAGCATCTTCCATAGCAAATAATGTTTGTTCATATTTGTTTTTAGCGTCAGTTCTAAGTCTGAATAACTTATCGGCTTCTTCTTGTTCTTCTGCAACTTTCTTCATCTTTTCAATATCTTCATCACTTAAATTACTCGTCATATTTTGAACGAATATCTTTTCAGAATTTGTTTTTTTATCAACTGCTTTAACAGTAAGAATGTTATTAGCATCAACCTCAAAGGTAACTTCAATTTGTGGTTCTCCCTTCCTGGCTGGTGCTATACCTTCCAAATTAAATTGTCCAACGTGATAATTATCGGCTGCCCTTGCTCTGTTTCCTTGTGTTATTCTTATAGTTACAGCTGTTTGATTATCTGCAGCTGTAGAAAAGATTTGAGAAGCTTTTGCAGGAATAGGTGTTCGAGCATAAATAAGAGGTGTTGCTTGTCCTCCTAATGTTTCTATGCTTAAATTTAATGGAACTTTATCAACCAATAAAATATGGTTTCCAGAATCACCTTCAACAGGATTGCATGCATTAGCATAGTATCCAGCTCCATATGCTATAGACTCATCAGGATTTAAAACTGCGACAACTTTTCTCTTAGATTGTGGTGTTGCCTCTGTTTCACCAAACATTTTCATTAACACCTCACCAACTTTTGGTATTCTAGAACTACCACCAACCATAATAACTCTATCAATTTCTTTTTTAGCATTATTTATTACTTCATTCAAATTAGTATTACATCCTGGATTGGCTTTAAGTGTTCCAGCTTCATCAAGTTTACCATTGTCAAAGTATACTGGATTAATATGACCATAAGATAACATAACACCTTTAACTTTTTCTTCAAGTCTATTGAAAAGTTCTCTAGCTGCCCTTTCAAATCGAGATCTAGAAACTTCAAATGTAATTCCATTAGCTCCTCGGAACCATGGAAGATTAAAGGTTACGCTAGTGTTGGAACTAAGTTGAGTTTTAACTTTAATAGCCTCTTGTCTGAGTCGAATCTCATTCTTTTTAGCTTCTAATGGATCACAATTATCATAAGCTTGCTGTCCATATTGTTTAATAAATTCATCTTTTGCCATTTGAATTATAATGTTATCAAAATCTCCTCCACCTAAATAATTATCTCCATCAGTAGCTTTAACTTCACCAACTGGATTTTGTTCATCATATGTGAATTCTAGAATAGAAACATCATAAGTTCCACCACCCAAATCAAAAACAAGAATTCGTTCTTCTTGATTAGGTCTGGTTTGTTTATTCTTTATCATCATTGATCCATATGCCATAGCAGCCGCTGTTGGTTCAGCTAAAAGAACAGTCTCAACTTTAATTGCATTTCCAGTTTCATCTTTTTTATTAGCAAATGCTATTTTAGATGCAAGAAGTGTTTTTTGTTTTGCTCCATCATGGAAATATGCTGGTACAGTGATAACTATAAATGTAGGAAATGCATTCAATCGAGTTCGTGCTGATTCTGCTAAATAATTTAACACAGAAGCAGAAATTTGAATTGGTTCATAAACTTCTTCTTTTCCATCAATTTTAACACAAATTCCAATATTATCCTCAACTTTAGCCTTATCAATACCCTTATCTGTATCTTTTATTGGCTTAATTTTAAAAGGCCAGTATTTCATAGACTTTTGGATATCTGGATGATAGTATCCTTTACCAAACATTCGTTTAGCTTCATACACAAAATTATCTGGATTAGATTGAGCTGCTAATATTGCATTATTTCCAACTGTTATTTTTCCTGCTTTACTATCTTTAGGGAAACATACAACGGAAGGCGTTATTCTCTCACCATCAGCATTATCAATAATTTGTAATTCACCGTGGACCATACCCGCCATACATGACATAGTAGTTCCTAAATCGATTCCTAATGTAACTTTTCTCTCTGACATCGGTTGGGGTGGAAATTTTTTTTACTTAAATATTTTAAAATTTTCTAGAAGAAGAATATTAATATTAAAAATTCTAGAAATATAATTTCTAATTATTTAAATATTGTTATAAATGTATAAAAACAGGTATTGATTTTAATTACTATATCTTTATATGTGAAAAAATACCCCTTTTATTGAACATATGCCTGTATATGCATTATTTGCTGTTCGAAATGAGGATTATAAAATAATAAAAGAAGCATTTTATTTAGATAAATTTGGATTTTTTGTAAAAATTCAAATCAAAAACATTTGTAGAGCTATAGCTACAGATTTTAGTTCACAAATCAAAAATGGAAATGATAATTATATAGAAATAGAAGAATCACTTAAAACAGATAAAATAATAATAGCAACAAAAAAAGATAATTATAAACGAATTATTGCTATAACAGATTTAGAATATAATTCATCTATTCGTTATAAAGTTATAATGACTGCAATGGATATAACCCAAAATTATGATTTATTAGTTCAAGAATATAAAGATTGGACAACTAAAGATCTCACATCTCAAATTGAAAAAGAAATAAAAGCAGCAAATGATAATGTAATTAAGGGTCTTTCTGCTATCTTAGATAGAGGACAATCCCTTAATGAACTTGTAGAAAAATCAGAAAATCTTTCATTACAAACTAAAAAACTTTTTAAGACAGCAAAAAAACAAAATTCATGTTGTTCTACATAATTATGTTTAATGTTAAAATATTTACTTATATTATATATTAAATGCTTATTTTTAATTCAAATTTAATTCACTTTGATCTAAGTGTTTTTCTTCATGCCATATTTGAGTATCTAAGGGACAAACATTTTTTGTATTAAGCCATTTATTAATACAATGTTTATGAAATGCATGGCCACATTTTCCCCATGATACAGAACACACAGATGATACTACATCATTTTGGCATTCTATACACATATCCATAATATGATTTCTGCAAATAGTACATGTTTCAACTTGTAAATCTAATGACCAAGTTGCAACTAGATCCCAATGTAAAATTTCTATTTTATTTTTTTCTTCAACCATAATTTTATGGTTTTAATTTATTTTTATTTATAAAACATCATATGTAAATACAAAATAACCATAGACATATTATTATTTTAAATTTATTTTATATTGATCTTTTGATTTTTTTATTATAATTCCTTTATAATTTAATATTTCGATAATATCTACTGGATTTTTAATATGATTTGTCTGTTTTATAAGATTAATTAATTCAAATTTACTTATATATGTACTGTTATTTGTAACAATAAATTCTTTGATTTTATCTTCTAAATTATTTATTTTATTTGATGATACATTAGAATCTATATCTTTAAAGTTACCTAAAATTAAATTATAAAACTGAATTATAATCTCTGCATCTTGCTGAGTTACAATAGATCTAAGATGTAATTTTGAATAAGCTTCGCCTAATCTAATTAATGATTCAAAATTACGTATCGTGATATTTTTATTGGTTCTTCGTATTTTTAAATAAAATTCTTTAAATATTCGTTGAGCATCTAAACTTAAAATTGGTTCATAATTCTTTTTTGCATAATTAATATATTGTTTAATAAAATCTGATGATATTTTTATAGATGTTTCTTCATAAAATTTAGTTTTATTTTGAATTTTTTGTATTAAATTTTCACTTTTTGATAACGAATCATTGGTAGAAATTTTATATTTTTTTTTAAATATATAATTTGAAATTTCATAATTTTCTTTTGATGTTAAATTATCTGTTAAAATAAAAATTAGATCAAACCTACTTAAAAGAATTGAATTAAATTTAAGATTATCTTTTATAGATTTTGTTTTAATAAATTTTCCATTTTTAGGGTTTGATGCAGCTATAATAGTTGGTTTAATTGGTATTGAACATAATACTCCACCTTTTGCTATTGTAATCATTTGATTTTCCATAGTTTCAAATAAAGATGCATGCTCAGTAATTTTATCAAATTCATCTATACAACATATTCCTTTATCACTTAATACTAAAGCACCTGCATCAATAACAAACTCACCAGATATAGAATCATGAGTTACAGATACTGTTAATCCTACTGTAGTTGTAAAATTTCCACTAATATAAATTGATTTAGGTAATACTTTCACTGTATTAAGCAAAAATTTACTTTTTCCCAAGCCAGGATCTCCAATCATTAATACATGGATTTCAGATCGTTTTTCTTCATCATTAATAATTTTTCGAGTTCCACCAAACATGCTTAAAATTAAACCTATTTTAATTATCTCGTTGCCATAAATATCAGGATAAATATTATTAATTAGAATTCCTAGTATATTAGATTGATTACTAAATTCTATAAAATCATTTGATGATAAATTTATTGTTAAAAATGATGTATTTGCATCTTCCATATCAATTAATGGTTCTGACTTATTTATTATTAATTGTTGTTTATTATCAAAAGTATATTCTTCTGTAATATTCTTTTCTTGAATTTTTATAATATTATTAAGTTCTAATTTAATTTGATAATTATTATCTTTTAGTTCAGCTTTAACTATTCCAATAGCTTCAATAATATCTCCTGGTGAATACATTCCAATATTTTTATTATATACTATAACATCAATAATTTTTGGTTTTTCACTTGTATACAATTCTTGAATTTTAAGTTCTTGTTTATCATAAATAATTGAATTTGGATGATCATTAAGAAAAATAAAAGTTTTAGATTTACATTGACAAATCTTAGGTGGTATATATATATTATTAATTAATTTATGTTGTATTATTGAATTACATTTGGTACATTCAAAAAATCCAGATACATTTTCTAGTTTTTTATATCCAACATGACAAATTATACCTTTAATTAAAACAATTTTTCCTATTAAATTAGGCAATAATTGTGAAAACGAATTAATTATAATTCCATTATATTGAGATATTCCAATACGTGTTTCTATTTTAATAAATTCATTATATCGTATATATAAAAATTCTGAAATAGCACAAGATATACATTGAATGTCTGGAAACATTATATAAAATTTATAGGAAATATACTGAATAGATGTTTTTTTAATAGTAAAATTGGATATATAGTCTAAAATATTATTTACCATTAAAATTTTTTCATTAAATACTGTAAATTCATCTTCTATAAAATATAAAAACCATGTCTTTAGACTTATCTGATTTATCATGTAGGATAATTTTTTCCCTAATTTTATCAATAATCAAACAAATTAGACATTTTAAAATTATAGTATAAATAAGAAAATCCAGTAATTTTATATATAAATTTTATCTCAATTATTAAAAAAATATGATAAAATATTAAATTAAATCTAATAATATCAATTTATTATTGAGCATATTGTGAACTATGAAAAATATGCTTTTTTAAATTATTAGAAAATACATTAATCAAGTTTATACAACTTATAATAAAAAAAATTTAATAATTAAATTAATTATTTATAAAATATCTTTATTAAAATACAACAATTAAGATAAAAAAGCAATAACCTAAAAATATAGGATACCCTATTATAAATGTCTTATCATAAATTTGAAATCGATAGCTTAATACCTGTGATGCAACATAAGTCGACCATGATGATATAAATAAACCAAATAATATTAAAGCAGCTTTAGACATCCCAAAAGCAAATATATGAAGCCACGAAAAAATTAAAATAGGTAAAAATGAATATCCTAAAATATTACAACATGCTGAAAGGTTAACATATTTATCACATAATAAATTTGTTAGTCCATAAATTCCAATAACTGTCGAAATGCTTAATAAATATATATGTCCAAAATGAAGTTTGCCTAACAACATTAATGCAATAGTAAATAAAGAAATATATACTATTGGGCCTATAAGATCAAAAGAACTATGTGTAACAGCTTTTTCTCTATCCATAATAACATCAAAAACTAAAGTGCTTTCTTTGTATATATTTTCAAAAGATATTCCTAATTCTTCTAATAAAGGTGGATCATTTAATAAAGATCCTGTAAATAATGTTTTAATATCTAAAGTTGGACGATATTGAACTTTATTTTTATAACTTTCCATAATAAATGTAAGAGAGGGGTAGAATTTTAGTAATATGATTTTATTATATTAAATTTAATGTTTTAAGTATTTCATTGACTTTTTGTATTGTATCATTAGGTCCATTAACATAAATACCATTAATTCGTGGATGATGATATATTTCATAATCATTTCCACCTGGCTGAATTCTATCTCCAAAAAAATAAATAATTTGCTCTTCAACATAATTTAAACAATAAGTTTTGTCCCATCCTTTTGGAAAAATATCAATAGAAATTTGACCTCCAATAGAACAAACTAAATTTAATTCATCAGTAATATTTTTTAAATTAAGAATAATTTCTTTTCTAATATTATGTTTTTTATCAAATTCAAAAAATTCTAATCTTTCATGATAATTACATGATCTTCCTATAGGTGAAATATTCACCATTGAATTTCGCGTTTCAATAAATAAACCTCGTTTTTTAGGACAATCAACCTTAGATAAAATTAATAATATTTTATTAATTAGTTTTTTAAAATTATCTTCTCCTAAAAATTCAATTATAGATTTAGAATTTTGTAATATTCCATCTTTAAAATATTGTAATCCATTTTCAGGAAATCCGTAATGAAAAATATCTAAAATATTTTCTCCAATTTGCTCTTTTTGTTTTTGTAAGTCAGATCCACCAACAAATCCTATATTAACTTTTGTTTTTAGTTGAAGTAACATATCTATTATTTTTTTAGGTGCTTTACACCTAATGTCACATAAAGTTCCTCCATTTTCAATAAAAAAGGAAAGTATTTGGGTTTCTGATCTTATTAAAAATTATTAATAATTATGTATTTATTTTTTAAACAATCTTTAAATAATAATTTTACATATCTCTTATTTAATTTCCTCCATTTTAAAAAACAAAATTTATTTGGAAACTTTTTTCTTTACCTTCACACTACGGTTATATTTATTGAACACTTTTTTGTCCTCCTCACATTAAAAAAATTTTTTGGTTTTTACATTTAAAGAGAGTATAATTTATTGGGGAATATTTTTTTTTTACCCTTCACCATAAGGGGTGTTGTTTATTGAAAATTTTTTTTGGTCTTCCTCCTCCCTAAAAAAAATTTTGTATTTACAGGATTTTTGAGAATAAATTTTCATAAGTTGGTTTTCGAAAATTTAGACTGTTCATAATATAAATAGTCGTTGCCTAATAATGAAATAAAGGTTTTTTTTCATTTTTTACATATTAAATTTAAAACTATTTTATTTATTTTTCGTTTGAATTCTTAAAATTGCTCTTTAACATTTTTAATTTCTTTCAACGAGCTAATGCTTCTTTTTTACTTTCAATTTTTCCTAAATGGTTTCTAAATTTAAATTGAAAAAACTCATTTTTCATATGAGGGCCAAGTTTTTAACTTTTTATTTTTTTACCCTTATTTATGTATAAAATAAACAAAAATTGCATTTCTGATTTTAGTGTTAAGGTACCAATTGTTGCTTTAGCAACAAAATCAAAATTATTTGATACTTCTTTTTCAGTGACAAGTGAATTAATATTATTTAATTATAAAACTAAGCAAGAACAAATATATAAAAGCGAGCATAGGTTTTGTAAAATTAAATGGTGTGAAACAGATAGTAGTTATTTAGCAGCAGGTCATGAAAATGGATTTATTTCTGTATATAAGTTAGATAAAATAGAGGAAAACAATGAATATACATTAAAATTACTTTTTATAAAACAACATCCTAAATCATCAGATGATATTATTGATTTAGATTTTCTATCAAATAAGGGAATGCTAGCAGTGTTATCACAGAATGGAAAAATTTTCTTTATAAATATTAATGATCAAAAAGAATATTTTGTTGATGTTATCATTGAAAATCCAGTATGCATGGCATGGAATTATAAAGTAAATAAAATTTTAGCAGTAGGAACTAATAATGGACTAATTAAATTAATCGATATTAAAAAAAATACTGTAATTATGACAATATCAGATTATAATAAAATTAAGAAAATTTGTTGGGATACTTCTATTCCAACAAAATTAATTATTCAATCAGACAAAAAATATTTAACTGAACATGAATTATCAAATGATTGTAAAAATGAGTTAGAAGAATATTTAGATGATATAGTTACATTTCATGATCAATTTATTGTTACTGCTAAAGAAGTAATTAATACCATAAATAAGACAATTTTTAAATTTGAAACTCCAATTAATTCATGTACATTATATAAAAATATAGTAGTATATAATAATAAAAATGGAACTACTGGTGTTATTTATATTCCTGTTATGAAAGAAAAACGACCACATTTTAGATTTGAAAATAAAATATATACTAATAATAAAATATATGTTATTAAAAATAAAAGAAATATTAGTAATTATACTTTGGATAATGAATCAGTGGATAATTTCTATAATCAATTACGTGAATCTCAGTATCCAAGTACATTTTTGTTAAACAATGCACATTCTATCAATAAAGTTTATGTTAAGCATAGAATAAATCCAATTCATGTAGATTTAAATAATAAAATTCATCAAGAACTTATTAATGGAAATTTTGCTATACTACAAGATAGTCAAGAAACAATACCTTTAAAATATTTATTAAGTTTATTAGAAAAAAATCCTGAAGTTTTAAATACTATATCTAATTTTGAATCTTTTATGTTATTTAGTAAATTACATGATAATTTTTCATTTTTATATAGAATAGATAATTCTAGAATTTTAGCTGCTATTTTAATAAATTTGAACTTGGATTTAAATATATTAACTAATACAGACAAAGAAGGAAAAATTATAAAAGGAATTTTAACAAATGATGATAGTATAATTAGTGATAGATTAATATTAGGAAATAATTATTATAAAAATATGTTCACTATTGAAAAGATGCTAGGTAATTTAGAAAAACCTATTGAATCAGATTATTTAAGTGAATTTTTTTGGTATAAGCAAAGTATAGGAGAATATGAAGCTGTAAAAGATTTAAAAGTACAAGATATTAAAATTATTCAGTATAAAAATGCTATAAGCAGTACTACCTCTATTGATTTAAATAATTTTTCAAGAGATTATAATAAAACTGATTTAAATAATAAATTAAACAAAACAACTTTGAGTTCACATTCAAGTAATATTTTTAAAAGCAATATATCCAATAATACTGATTTAACAGCTAATTCATCACAAACTATGCCTAATTTAAATATGAATTATAATATTAAGAAACCTATGTCTTTAAATAATCCTCCTTCATCAATAGGAATAAAACCTGGGATTAAAAGTCCTTTTGCTCCTAAATCTCCAGGAATAATAACTAAACCAATGGAAAATCTTCATAGTTCGCAAAATATAAATTCAACAACCATTACTAGAAATCAACAAATTCCAATACCTTCATCATTAAAACCAGGACTACGAACGCCTTCATTAGCACATAGACCAATGGGAATAAGAGAAACAAATCCAATAGGAGATAGATTTCAATCACCAAATAGAGTAATGATGAGAAATCCTTTACATCCTAATGTTCCAACTATGGGAATACCAGGAATTAATGATTCTAGAAAAATACCAGATCCAATAAATAATATTAATCCTCAACAAAATAACCAAATAAATAGACAACGTATATTTGAAGAATTTGATATGTTAATTAATCAAGTAAAGCAAAAAGCTGAACAAAAAAATTCAATATTATTAAATCAAAAAAAACAAAGATTTTTAAATGGGTTAACAGCATATAACAACATTGATAGACAAACAACAGATATAGAAATTTTATTGCAAATGGATAAATTAATACAAAGAATGAAACAACCTGATCATAGATTAAAAATAGATTTGGATGGAATGAATTTACACAGTACTGTATGGATGCAATCATTAATTGAATTAATTAAAATAGGGTATTAAGTGAAGTTAATAATTGTAAACTGTTCCAAATATTGTATTTTTATAAGTTACAATAAATATTTATAGAAATGATTTATTGAGAAATTTCAATCATTAAATAATCTTTGTTTTGTAACATAATGTCTTCATTTTCAGAACATTCAACTATTTCCATGTTATTTTTTTTATAAATTTTATTCAATATCATATATTTATTTCTTAATTCTACAATTGTATTTACCTTTTCTATAATTATTGGATAATTAATAATTTCTTTTTTATTATTAATAGTACATATTATCATTGGATTTTTAATTTTATTCATAAAAATAATTAATCCACCAGTACGAATTATATCATTATCATGCTTATAAAATATAACTTGTGGGCTATTTTTAAAAACATGAATTATTTCAAAATCTTTTTTATTATTAGATTTAATACATTCATGATTACACATTATGTAATGCTGTACTAATATTTTTTTAGTAATATATAACTCCATAATAAATGGATGAATATGAGGTATAGATCCAATACTAATTTTATCAAACATTGATCCTATATAATATATAACACCATTAGTATTTAATTCTATTTCATTTTGTTCATTTATAAGACATTCATTGCAATTAAAATTAGAAATATCATGCTTATTACAAATAGATATTGATGGAAAAATAAATTCTAATGTATTAATAGTTAAATCAGCTTTTAATTGATTTGCTAATTCAGTGGATGTTAAGTTTCTTTTTATAAATATAGGAATAGTACCAGTAATATGTGCTAATTTAATAAAAATAAATCTTCCATCAATTAAAAATTGAATATATTCCTCAACGATATGTTTTTTTGAATCTGTTATAATTAGAATTGAACCATTTTTAATAGTTTTAATATCATTAATTTCAGTAATTATATTAGTTTTGTAATTATCTAAATAATTTTCTTCATAAATATTTAAAAATCCTTCTTTTAAATCTTCTATTTTTGTATTATTAGTTATTATTACCATTTTAATTAATTTTAATTTTTCAGGATATACTTTACACTCAATAGTGTTTATGAAAATTTTAATAAATACAAATTTACTGTTATTGAGTGAATATTTACAACTATTAAAGCAAGCTATTAGTTCTGCATCTTGACAATATTTGTTTTGTATTTGATTCCATGCATTATTTTTAAATTGGAATATATATGAATCTTTTAAAACTGTTTTTATATTATCAAATTGACTCAATGTTACTATTGGTAAATTAATTTTTTGATAATCTAAGCTGTTAGCATTATATATTCCTGGACCTATATATTTAATAGGTTTAACATGTGAAAAATAAGTTTTCAATGTATTAGAAATAATTGGAATTTTATAAAATTTTTCTTCTTCCACTAAATTATTAGGAATAGTATTTAAAAAAAACGGTAAATTATCATTATTTGTATCTACTCGTTGATATATTAAATAGTAAGCTGAAAAATGTTTTCTTTTATTTGTAAATAAATTTTTTCCACCATAATTCCAATCAATAGCTTCATCTTTTATTGCTTCTGTTACACATTCATCATTGAATTTGTAATATTTGTTATCAATTTTTATAAAACAATAAAAGTGACCTTGAGATATTGAACCTGCATGAACTATATTACTTAATAATTGATATTTAGTATTTGATTTAGATTTTACTTTTTTACTGTTTGCATAATTTTCTTCATCATATATATACTCTGTTATATCAAGTTCTTCTTCATATTTACAGTATGAATTATCTTTAACCGAATCACCTTTTCTCCAATCAAAAATAAATCTATTTAATAAAATAAATAAATACTTAGGTGTTTTTTCGATCATTACCTTTTTTTTAGCACATGTTTTTCCATGTTGTTCACAAGTAAATTCATCAATCACCTCAGGAGAAGTAAATTCATTAAATGCATCTATAAGTGTATTTTTTATATTTAATGAAATATCTTGAAAAGGTTCACGTTTTTTAAGTTTACAACCACAATTAATATCAAAAATAGTCACTAATTCACCCTCAATAGTATTTTTAATAAGACTTTTATTTTCTAGTTCAAGTTTATCAAAAAGTTTCTTACTAAATTCATGAACGTCTTCATGATCATGAATATTTTTCACAAATTCTAAATTATTAATAAATTTTTTTACCAATGTATATTCAACTCCGGTAGAATTTACTGACATACTATAAAATAATCGTTTTAAATTTAAACAATAAAAAGCATCATTACTATTAAATAAATATTTTTGAAAAGTATACATATATCGTATTGTCTGTAAAAGAGAATTAATATAACATGTTGCACCCAAATTAGCTAATCCACAATATTCATTGATTAATTTTACTTTATTAATATATATAGTCATTGTTAGTGGTACTTCAGAATTAATTTCCTCAATATTTTTCTCAAACCTATTATTTAAATCAAAAACAAATGTGTTGTTAAAAAACGAAAAATCAAATAAAAAATCAAGTTTACAATTCATATTTTGATAAACAAAATTTATCTCAATTTTATATGTAGTATCTTTGTATATTTTTGATAAAAACTCGATACAAACTAAATTTTTATTTAAATTAACCTTAAACTTTTCATTATAACCTTTAACTGAAAATTCAGTGGATTCTTCTTTGATTTTAGTAAAAATATATTTTTCAAACATAATTTCCTTCATCAGGGTAAATTTATTTTCCTATAATATCAATAATAATAAAATTACTTAAATTTTATTTAGAAAAATATTAATATATTTTACTAATATAATAAATTCAATATATAATTAAAATAATTTTATAATAAAAAAACGAAGGCTCAAGAGGGGATTGAACCCCCGACCCACAGTTTACAAAACTGTTGCTCTACCACTGAGCTATTGAGCCTTGTTTTATCATAATTAATATATTATATAATTTCTATGTTTTATAAAAAGAAATTTTGAAAATCCCATATTATGACACATCAAGAGTCTTTAACTTCAGATGATATTTATCATGCAGCAAAAGATTTATTTAATAAAAAAGATAAAATTAATATTGAAAAACTTTTTGGAAAATATCTTAAAGCATCATATGATTATCGTTTATGGAAAATTTATTTATCATATACTAAAACACTGAATTTATCCCATGATAAAATTATGGATGTATATTTTTATATTTTAAATCATTTTGAATATGGTTATGATAATTATGATTTTATTATAGCATGTATAGAAGAACTTGATAAAAGTGAAATAGCAGATTCAATTAAAAATGAAAAAATTAGAAATATATATCAACAATTTTTAAAAATACCAATGAACAATCTTAACAAACTTTGGAATCAGTATGAAACATGGGAAATTAATATTAATAGATTAAGTGCAAAAAACATTATAGAACAACAGCAACTATTATATTTAAATGCTCTTAATGTATATCAAAAAATATCTCCATATTTACAAACAAATGCATATTTTAATATATTTGATATTGAACTGGAAAATCCGTTAAAATTAACAAAAAAAAATTTTGAAACTAGAATATCTTTTGTTTTTCAATATTTTTTATGCTTAATACCTAAAAAAGAAAATATAGAAATTCTTAAAACGTTATATTTACCAAATGTTTATAATTCTAAAAAAGACTTAGAAAAATTGAAAAATTTATCGTCAACATCATTATTGCTTTCTTTTTGGTTTTCTTTTTTTTATAAAATTGATTTATTTAATCTTAACTCAAATACTAACTTTACTCTTATAGCTATAAATTATTTAAATTTTTTTATTCAAAATAAGAGCTTAAAGGAGTTTAGAGACATGTTTAATCATATATCAACATCGTTTACAGATATCAAACCACATTTATATATATTTGTTGCTGAAACAGAATATATGCTTAATAATCCTGATGAGGCTTATAAAATTATGGTTAAAGCATATGAAAAGTTTGGAGATAATAATTTGTTAAATGAAAAGTTTATAGAATTATTAATTAAATATAATGATACTGAAAAAATCAAAATTTTTTTTAAAAAATTAGTTAAAACAGAAAAAATGTATGATATGATGATGAATTATGAATTCAGAAAGGGTTGTTTTAATAATTATCAAACAATTCTTTTACAAAAACAAGATGCTATTAAAAATAATGAATTATTAGATAATGTTTCAATAGCATTATCACCAATTGTTTTTAAAGGAACGCAAGGCGTTATTCAATCAATTATTAAAACATTTGAATATTTAGATTTAACCTTCTCAGGTACGTTTAATACAAAAAATATTTTAAAAAAATTACCTATTTTACCGGATAATGAAAATATTTTTAAAAATATTGATATCAATGCTTTAATTCAATTGCTAACAAAAATAAATTAATAATTATATCATACAAAACTAACTATTTTTATTATTCTACCCTAATAAATTATTTATGATAAATCATAAGCATCGATTATCATCTTTTAAAACAATGGTTGATTTAGATATACAATATTTAAATAATATATTAACTAGAATGAAATTACATCATCCTCTTATAATAAATGATTTTTGCAAATTTTATAGAACTATTTTATCGAATTTAGAGTTTTTAGATATTAATTTATTAAAAATTTATTCTGAATGCTTTGTATATGCATATTTTAATATTGATATATCAAAAGTAGATATAAAATTAGTCAATATTAATATTATGAAAGCAGGAATAAAACAATGCAATAATTTTCTTAAAAGTATTGAAAATCAATCGGATATTATACTGAATGATTCTCAAAAACCAGAAGATACTAATACTACTGTTAATTTATATTCAATTTTAGAATAAAAACTTAATGATAAATTTTTTTATAATTTCGTACCCCATGTTATCAACATGTGTAAAAGGAACTTTTGTGTCTCATAGACGAGGATTAAGACGACTTCATCCAAAGCAAGCCATATTAACTATTGATAATGTCTATGATAGAGATACAGCTTCAAAATATATTAAAAATGCTGTTTTATTCACGTATACACGTCCTACTGGAGAAGTAGTTAATATCTATGGATTTATTAGAGCTGTTCATGGTAATAAAGGTGCTGTTCGGGCAACATTTGAAAGAAATTTAAGCCCAAAATCAATAGGTGATAGAGTATTTATTAAGTTATATAAGATTGAATAAATAATTTATTTTTAAATTAATGTTGTATAATTTGAACTATTTGATTAGTTGAATTATACACTACTAAATAGTTATTTTTAATTATACTATTAATACATTCATCTATTAACTTATTATCTACTAATAATTTAACTATTCCTTTGATAATCATATCATTAACTTCTAATGTTAAATCATTTTTAGTAGTTATATTTATTTTATATTTTAATACAGAATTTTTTAATAAGTCTTCAAAATAATTAAAATGATTATGAAATAAATTATCATTAATTTGAATTATAAATGATTTTTCTCTTTCTTTTTTTTGTTCTAAATCATATGTAATATAAATTATTAAATTTTCCTTACTAACTTTTAGTTCTTTATTTAATTGTATATATTCAAAAGTAATTATTTTTGTATTTAACATTAAAATACTTTATCCCCCAAAATATGACTTTTTTTAACAATATTTATATAACATATAATTTAATTTATAACACATGGTTATTTAAGAAGTTAGAATTTAATAATGTGCATAGTGTGAATACATTTATAATGTGCTTTATAAATATTATATTATTATATATAATGGTTCAATTAATTTTAGCAAAATTATTTATAACTCGATATGTAATTAATGTAAAAATTTATTGTATTAATTGGTCGATTTTTTGTATTTTTATATTAACATATATAATGTTATGTAAAATAATATATTTAATATCTTTTAAAATATCATTATTAAAAAATATTAATGATAAAATAAAAAAAGCTACTAAGGAAAATAATAAAATACAAAATATACCGAATGTAAAATATAATTGTACATCTGAAATTATATCAATACCCTCTCAATCTAATAATGCTTCATTAATTGTTAAATCATTAGAAAATAAACACAAAGATATTTATAAACAAAAAATTAATAGCAATTTTAAATTTATTATTTTATTACCATTCACATTTTATCCAGTATTAAAATTTATTAGTAGTTTCATATTAATTCAAAATAGATATGTTCTAACCTTAGTTATATCACTTTCTTATTTTTTTATTATTCAAAACATTAACTATAATAATATTATAAGCCGTTTAATAATGTTTATAATGCAATTTTGTTCAACTATGGCAATAGAATGGATGATATATTATATTAATAAAAATAATAATATATTTATTATTATTCAAAAGTTATTTATTTCAAGTATAATGAATAATAATAAATAGTACTTACATTCTTAATGTTTGAAATAATAATAATAGTGATATAAATGAAATTGAATTTTGAATTATAGATTCTCTCTGGTTCTGAGGTGTATATATTCCATAAAGAGAAACTTGAACATAATAACTCATAATAAGAACAAATAAAAAGCTAATAATATATTGAATAAATCCAAACGACTTTAACAGTGTTATTAACGGACTTCCAGAACATCCCGATGCAAAAATTAATATTATAAATGGTGAAAAATCTACTGCTGCCCACCATGTAAACAAAAATGATACTATTATTAAGCTTATTAAAAATATACTATAAAATACTCCAAATTTAACAAGAATATCATTAATAGTTAGCTTATTTTCATGAAATGGTAAAAACATATTTATTACTAATGCTAAACAATTAAAACAGCCTAACATAAATAAATAAATAAAAGGAACCATGTACGAATTATCTATATCTTTGGGATTAATTGGATGTACTGTTAATGAATTACACCATACTTTTATAACTGAATTACATATGCTTCCACGTCTTTCATTGTTTGCATAATGATTTCTGATAATTTGAATTCTAGTAGGTTGTTTTACCATTTGTTTATTAGGAACAACAAAAATTTTAAAAGATTTATTATCTTAAAAATTATTACAATTAAATTAATATCGAATTATAAAATATTATATTGAATTTAATTATTATTTATTGTTTTAATATGTTAGTTATATTATATACTTTAATTGAATTATTTACACTACCTATAAACATATATATATTGCTATTAAAATTGACTATTGTGAAAGCTGTAGGAATTGTATCTAGTTCACTACTGTATAAAATATTCAAATTAATATCTAATATTTTATAATACTTATCATTAGATATTGTAATTATTTTATTATTAATAATTTCTATTTGAAATATAACATCATTATGAATTTTTTTCTTTAATTGTATTGTTTGAGTATTTAAATCAATTATATATATTGAACCTATAACATCTGCAACATATAATTTTGTGTTATAAACTTTTGCATTTTCTATTGATTCATCAACAGTTATAATTTCCATAAATGTTTTATTCATTTTGTAAAATATTAATATAGTATCATTATTGATTATTATACAATTTGTATTTGAAGATGAATCACAAAATAATAAAAAATTAGAAATTTCAGTATCAAAATTATACACTATGTTTGGTTTTAACATTTCTAAATTCCATTCAATAATTTCATTTGAATCTGATGTGTATAATTTATCATTAAATACTTGTTGACAAGTTAATTCATTTTTGTGACCTATTAATACACAATTAGGATATGGTGCATATTCAACAAATGGATCAAAAACCATGACATCGTTTTCAAATGTGATAACTGATATAAATTCTTTGTCAAATATTTTAATTAAATTTCCTCGAATACAAGTATTCAGCATAAAAATATCATGATGACAATAAATTTCTTCACCATTACTTACAAATACAGCTACTTGGTTAAGTTCATCAGCAATAACTGTATATAATATTATTTCATCTTTTGTAATTTCTGTTTCAAAAATATTTTCACTAGTTTGTGCTAATTTGACATCCGAATTAGGAATCATGTATTTTTGATTTTCTTTTCCTCCAATGTCTACAAATTTATTGTATTTAGTTATATCTTTACTTGATAATACTCCAATGGAACAAATCATGGGAGAAATGAAAAATAAAAATTTTGTACCCCTTGATGACAGAAAGTTATAGGAATGTTTTTCCATTTGCACAAGAAGTTATTCTAGAAGAAATTGTTGGAAAAACAAGAATAGCATCTTTTCCACCAACAGGTGTTGAGTTAAAAGAAGGAAATGAAGTATATAAAACAATTGTGCAAAACACTTTTACTGAATTACCTGCATGGTTTAATGAAAATAAATTAAGTAAATACGAATTAGGTATTGACATATATATGAGAAATAAAATAATAGAAAAATATCGAAAAGAAAAAAGAAATATAACAATCAAAGAAACATTTGAGGAAATATGTGAAAAAAATTATACTGGTACTGATTTTGAAATCTTTTTAAAAACATTTAATTTTTGCGAAGATAACAAATTAATTAATTATGGAATAGATATATTGACAATTTTTGATATTATAAAGCAATTATACTATCAAGATGAAAAAGATATATCAGATGAAATAAGTAATGAAACTAATGAGAACAATAAATCAATTATAGTAGTTAAAAAACATTTGTATTATAAAGAAAGATATATTAAACCTGAACTACTGAAAACTGTAAAATGTATTAAATGTAATAATTCAGCGCAATACTTTTCATCAAATCTTAATTTTTCATGTGTAAATTGTTATAAAAGTGATGCATTAATCAATAAAAAATTTCATGAAATAACACCTGAACTCCTTAATGCAATATGGAGTAAACATGAAGAATTTTATCTATTACAAGGAATTGAAAAATATGGAGATGAATGGGATAAAGTTATGGAGTATGTAAATGTAAATAATAAAAATGAATTAAATGTTAAAAAAACAAAAGAGATGTGTATATTTCATTTTATAAACATGTGTATACTTGAGACATTAGAAGAATATCATGCTTTACCATTTTTTAAATTTCAAAATCAAATTACTGCTTTTATAGCATTTTTATCAACAATTGATCCTGTACTAAGTAATAAAGTATCTAAAGAATTTTTAAAAATAATGAAAACTAAAGAAACACAATCAGAAGTAATTAAAGAATTAATTGATGTAGCAAAAAATGAAGCTCTTAATCGAATTACTTTAAAACAAGAAAAAATTCATAGATTAAAAAAAGTACGATTAGAAGCTTTAATAAAAAAGTTAGAATTAAAAATTAAAGCAATATATAATATGAATAAAGAACCATCGGAAGTTAGAAAAGAACTAACAGAAAAACGGTATGGCTTATTAGATGAATTATCCAAAATAAATTAATTATATATCATATTTTAATATGTTATTCCTTTAAATTTCTCCTATTCATAAATTATGATTATTATTCCAACTTGGTATTTATTGTTTTTAGAAAAACTTTTAGAACAAACGTGCAAAGAAACAGGAATTATTTTTAGTTTATTGGAAAATTCATGCAACCATTTAACAGATAATATTCTAGTAAATTTAAAATTATATCATTTTTGGAATTTAGTGGGATTTGGAACATTGATTTTTGTATTATTTTTTTTTGGTGTAAAATATAAATTAATTTTAGGGTCATTATTGTTATCTTTAATATTATATAATTTTCAAAATTTATTAAAAAATTATTATTATGAAAATTTATCTTCCCAAAAAATTACAAGCTATGCTGAGATAGAAGAGTGGAATCCATTTTTTATAAAAATATTAAATTATAGAATGAATAAGTTAGCAGAAGAATATCCTGATAATCCAGGTATTATTCTTTCACTTATTAAATTAAATAAAATAAAAAGTATGGCAAAAACTATACGAAAAAAAAATAAAGAAATTAATTCAAATGAATTTATTCAAAAAGAATTAATTGTATTTAGCATTTGCTTTATAATATCACTTTTTTTATTTGGATTTATTGGAAAAATTGGTGTATTGATTATTTTTCTTGGAACAACTATTTTAGGTAGAATTTTTTATTTAATGCATTTTAAAAATGATACCAATATAAGTAGAATAATAATAACAATAATAACAATTGCTACATGTATTGCTTTATGCTATTTTATTAGATTAATAGTAAATGGTATTTTAGCATTTATTTTTGCAACTATTGCATCCTTTTATGGAATATTTTTGTTAGGATTTGTATGTGGAAAAAAGAAAGAGTTATTATTTCTCTGGGTTGATCTTATTGAATACACAGAAATATCAAATCTCAATATTCCATTTTCACCAAGTTATACTAAATGGATTATTTTATGGCCTATATTGCTTTTTATAAGTTTCTGTTTTAATTTAATTCTAAATAATATTAAATAAATTTTAATTGGTTAAACCTCAATATATTGAATTTTATGATTTTTTTCTAAATCTAAAACATCCAATCTCCAATTACTCGTATTTAAAATTAGACGGTCTTTTAATGCTTTAATACATAAATTAATGAAATTAATAGATTCTTCGTGTGACATATTAAAATTATAATAATTATCATAAAGACCATATAAAAAATAAATACCATATTGTGTAGCAACAAACTGTGGTTCACTTAATGCTGAATAATTATCAACATAACCTAAGTAAAATGATTCATTTTGAACACCAATAAATATTCCTTGAGCCTGATAAGGATTTCTTGTTCTTAATTGATTATAAATCTTAGTCTGAAAATCATTTAATAATATCTTAGGCGTTATTTCAATTTTATAAGTATTCTCAAACCATAGAGCAGATTCCTCAATCCATAATTTCAAACGATATGTATCTCCTTGATGTCCAGATAAATGAATTAAATTTCCTTTAATATTAACAACTTCATCTTGACTTTCTTTTAATTTTAATATATTATTTTCTAAAGTTGTTTGAGTTATTAAATAAGCTTTAGTTTTCGTAACAAAAGCAAAACTAATCATATAGGGTAAAAAAATAATATAATAATATATTTGTTATCTATGGTTATTCAATCAATAATATTACTTTATATTTTAAAATGTTTTAATTGTTTTATAAATACGATTATTATACCCTCCAAACTATTTATGGATAAAAATACAGAATTTACAAATTTAAATGTTCGTGATTTAATAAATAGAATTCAAAATGGAGATATTATAAAAGATCAACAAAAATTAACTGATGTAAATAATCAATCTTTAAAAACATTAAATATTATATCATCGAATTCAAAATGTATTACGCCATCTAAATGTACTTATAGTTTCAAATCAACATTAACTAATATTATAAATAATGTTCCAGCATATCAAATTACCTATAATACAGAATATCCACCATTATTGCTTCCTAGTAAATTTTACAAGCATGAATTAAAAATAAGATTGTTAGGTATTTGCACAAATACTAAGATTAAACAAAAATTAGCTGATTTTGAAAATAATCAATATACATGTATTAAATGTAATGCATTATTATTCAATAATCAACATTTAACCAAAGAAACAAATAATTGGGAAGATAATAGTGATACAGATAATGAATCTATATCTGATATTATACCAATAAATGTAGAAAATAATAAAAAAAGCGCAATAATTAATTTTTTTTTCAAATTTTTTGATTATTAATTGATATTGAGTATTTAATATATTTAACAAAATATTGTTAATATTTAATAAATTTACTTTACACCCCATATTTTATATATGCTAAATTTATCAGTAAGAGATTTATGGTTAGCTAGAAATACAATTATTGAAATGTTAACCGATAGAGGGTATATATTTGAACAAAGTATATTTTCTCAATTAGAAGATTTTAAAAATACTTTTAATTTAGAAGATAGTACAAGTTGCAATTTTACAGCCATACGAAATGTAAATGATAAAGTAATACTATGTGCTATTCATTTTACTTCAAATGATAAATTATCAAAAAAACAAATAGAAACAATTATAACAGATTATAGCAATCAATCTATTTCTAAATTAATTTTAATATCATGCAATAAATTAAATCATGCAGCATTAGCCTATTTAGATAAATCAAATTTTTCTATAGAACTATTTTTATATAAGGATATTTTATTTAATCCAACAAAACATATTTTAGTTCCGCATCATAAAATTATGTCAATTGAAGAAAAAACAACTCTTTTAAACAATTTAAAATGTAGTTTAGATGATTTACCAATTATTTTAAAAAAAGATGTTATAGCTAAATATTTAGGAGCAGAACTAAATGATGTAATAGAAATAACTAGAAAAAGTAAAACAACTGGAGAAAGTAAATATTATAGAGTAGTCAAAGAATAGTAAGTAAATAATAATTTTTTAACGTATTTTTTAAATTTATTAATTAAAGTAAATATTTTTTTACCCTATGGTGTTAATACATGACAACATCGAAATTACTCTTGATAGTGACAATTCAATAATTATTAACTTGTTAAATAAGCAATCATATTTTACTGTGGAATTAGGAGACAGGACTTTTAAATCTACTCGATTTACATTCAATAAAAATATAATTGAAGAATATTGGAATGTATATAAAGATGAAAATATTTTTCAATTGTTTTTAACAGAAAAGGATATGAATATACATATATCAATAATATTTAATAAATATAAAAAATATTCAGATGAATTATTGTATTATTTTAGTGAAATAAATTGGTATAATAATGAAAAATTGATAAATATATTTCAAGTTATAGGATTAAATGTAGTCATGTTAAAAATGGATTTATCAATATTTGATACAAATGAAAAACAGTTAACATTTTTTGAAGCACATTCCGAAATTATGAAAAAATTTTTTTTTAAAAAACGACGATATTATGAGTTAAATTTATCTTTGAAAAATAATACGAGTAAAAATATAGAATCTGGAATTATGTTTAGAAATTTTATGATTTATTTTATTGTTTGTTTAATATTGATATTATTTTTATATAAATATATGTTAAAAAAAGGATTGGCTGGAGGTATTACATATAATATTATTAAAAAAAAACGATTACAATAAAATTGTTAATATACTAATAAAATATTTTATTTAATTATGTAGTAGAATTTTTCTTTATAAAAATTTTAATTATTTTTTCCCTTGTTCATGGAATTTATTTTTAATAATTCAATCATTTTTCGTTTGACTTCAGATACATTAGAAATAACTCCGTCTTCAAATTTATCATATCTAAATATCACGATAAATAATAAAAATGAACAGACTGATAGTTTAATATTATCTAAGCAAGATATTAATAATATATTTTTAAGTAATTCAAATAATGAATTTTATAGTTTAATGTTTTATGAAGATGCTACTTCTATATTTTTTAATATACGGTTATTTAGATATAAAGTATTTCTTGAGGATTGTTTCATATATCTCAATAATAATAAATACAAAGAATATATTCCACAATTAAAACAGCTATTTAGATCATTTGGATATAATTCATCTATAATGATAATAGATATGGACATAGCTAATATTATATATAATATTGATTCAATTGATACCCATTCTTCATTAAGACAACGACAAATAATATGTCAAAAATATGGATTGTGCGAAGAATGTTTTAAACACAAATAATATTAAATCAAAAATTTTATTTTTTCCCCTTGTTTAATGATAAGTTTAATTATTGTTATATATTATTATATGCAATCCTGGCTTAGTAAATTAAAAAATTTTCTATTGTCATTTTTTATTTCTACAAAGAACTTTAAAAATGATTTTGACAAAGCCAGATATTTTTTAAGCATTAATAATAAATATGCTACATATTATGAAATTAAACAATCATATTATAAATTGCGAAGAATAGTACAAAACCCATCACAATTAATGAATATTAATTGGGGATACAATTATTTAGTTAATAATATTGAAATTGAATTGTACAATTCAGACAATATTATTAATTATAAAGATGATTTTTATGATAAAATTAGTAGAATATATTTAAATCATAAAAAACCTGATAATAGAAATTTTTATATTTTTTGGAAAACAATAAATATTAAAGATAAATATGTAGAAAAAAAAGTTAAATATTTAACAAATGTATTAAAAACAAAAACAACAATTAATCAAAAAAATACCTTACCAAATGAAAACATAAATTTATTTAATGATATAAATACAAAAATATCATTATCAACAAATATAATACCAAATAAGAAAATTCCTAAATTTAAATGTGAATTATGTAATTAAAATTATAATAGTATAAAAACCTTAACTACACATCAACAGAGCAAAAAACATTTAATAAAACTAAATATAAAATCTTATTCAGCTTCTATTGAACCTACACTAATTCATACCCCATTACAATTGCCGAAAAAATCAATTATATCACCACAGGATGTAAATGATACAATTAAAGTTAAAAAAATAAATTTTAAATCAGAACACGTTTTATTTAGAACATGCCATATGTGTAACAAAGAATTTCAAAACAGAATTGATTTAATAAAACATATTCAATTGCATAATCTTAATAAACATTCATCATATAATTTTATTTATTTTTATTACCCATAGATGCAAATAAATAAGTATTCCGATTTACTTTTTCAAAGTTGTATAATTGTAATAATGACAATTATATTGATATATTCTATCTATACACCATCACTGTTTCATTCCTTAATGCTAATATATTCGAACAAATTTGCATTTATAGTTTATAATATTTTAATTAGTATTATTATATGTTTTATTTCTAATATAATATGCATATGGACTTTAGGTACGTTAAATGAATCTGATCAAAGTCAATATAATGAAATAACCTTAGAATATTTATCTAATATTTCTATGACTGCCGTGCTGTTTCCATATACCCTTATGATTAGAAATTTAGTACCATTTACAATCTTGTATAATATAAGAAGTTTTTCAACTGTATATCATTTATTAACAAATACTAATAATACTATTATGATATTTGTAGTTTTTTTTATTATTTATCAATTGTTTAACTTGGTTAGTTTATTTTCATTAACATATATGTCATTTAATGGATTACTTGCTTTTGAATACTTTTTAATTTTGTTTTATGTATTAAAAGATATGTGCTTAAGATATTTTAAAGATAGGTCTCCAGTTGAAGAATTTACTATTAACATTATTTATTTAATATTAAGAATATTAGTAATTGGAATATATGCAAAAAATTTATCTCAATTCCGTATGCCAATAACATATTTAAAAATGTTAATACAAGATATTCAAGAATTTAAAAAAAAAGTTCAAATATTTTATAACTATATCAAATTGTGTAAAGAATTAGATACTATAGAAGATGTAACTTTAACTGAAACTGAAATTTGCGCTATATGTACAGATGAGATAAAAAATGGAAAAAAATTGGGTTGCAAGCATATTTTTCATACAGAATGTTTAAAAATATGGTGTGAAAGAGAAACAACTTGTCCAATATGCCGAAAACCACTAACTTTAACTAATATGTTAAAATTTGAAACTAATGCTGAAATTATAAATGTAGTACCATTATAATAAATTATTTATTATATCATCTAATAATGTTATTATTTGATTTGCTTTTTCTATATTATTATTTAAAATTACATTAATGTCATTCAAATAAAATTGAAAATTGTCTATTTTATGAATTGTCGTATCTTTTGATTCTTCATGATTCTCACATTGTAATATTGATTTATAATCAATATTTTCATTAAATAAACGATTATAATCAAACTCTGGTAAATTTAAATGATCTTTATTATTCATTATTAAAATAAAGGTATAATAATTTATTAAAAAATATTTCAATTAAATAAATTTAATTATAATTTTTATGTTTTAGTATTTAAACCTCAATTTTATTCTTTTTTAACTTATTTTGATAAAATGTCAATTCTTCTCCTTGTAAAATATGACCATTAGCCTTTCCAGTTTGTCCAGGACGACTTGTTATAATTGCATATAAAAATCCTTTATTTATCATATTTTTTAATACTACATCCTTATCTTCTAATTTAGATATGTCACATCCTTCAGACGAGATTCTAACAACGGCGCTTTTTGTTAAAGTATTAGTTCTCATTAATTCGTTATTGCTTGGATGATAAATTACTTGCTCAATTTTAAATGTATTTTTAACAAATTCTTCTCCATTAAAATATTCAAATTCGCCTTCATTTAACCTTAGGGCTCGTTCTTTAATATTTCCTCCTCTACAGCGTAATTTCTTAATTCTTTCATCACCAATTTTCGTATTTGCAGGTTGGCGTCCCATCATATTCTTTCTTTTTTTTTGCATTTGATTTCGCATTCCACCGGTATGCTTCTTCTTATGGCGTCCACAGCGTGTTATTCCCATATTATCTAGGGATCATCCATAATAAAAATTTTTAAAAAATATTTTTCTTTAAAAATACTTAACAAAAAATTAAATATAATAAATTATTTAATTATATTGGGTTATATTTATTTCATATTTAGTCATAATTTCATTTAATCTTATATTATCTTCAATTCCTTTTACCAAATTTATAGCTATTCCTTCAGTTTCAAATCTACCGGCTCGACCAACTCTATGTAAATATGTTTGTGGATTATCAGGCAAATCATAATTAATTACAAAGGTAACATCATTTATATCAATTCCCCTAGATAAAATATCTGTTGTACACAAAACACTATTATTTTTTTGATTTTTAAAAGCATTTAATATTTTAATACGCTCTTTAAATGATTCATACTGAGTTAAATAATAACTATTAATATTTAAAATATAATGAAGATATTTTGCATTTTGTGTTTTATTACAAAAAATTATAGTTTTTTTACTAGAAATATTTTGTAATAATTCAATAATAACTTTAACTTTTTCTTTTTCAGTTTTAACAGGCAAATAATATTGTTTCAATCCATATAATGTTAAATGGGTATCATCATCTACAAAAATTGTAAATGGATTTTTCAGATATCTCATACATTCTTTTTTTGATTTTTCTATTAAAGTAGCTGTGAACATCATTGTAACTATTGGATTGATTTTATTATACAATTGTGAAAAATTTTTATTAAATATAATTTGATCACATTCATCAACAATTAAAATTGTATTATCTTTTTGTTGTAATGACTGCAATGAATAATTTATATCAATTATTAAAATTTTATTTTGGATATTTATATTAGTACTAAATCTTTTATATTCAGATTGTATTTGATAAACCATTTCAATAGTATTACACAATACCACAATATATTTTTCAGGATATCGAATAATAATTTCAATTGTACTTAATACAAATACAGCTGTTTTTCCAGTTCCAGACTTAGCTTGACATAATATATCTTGTTCTAATAAACACTTAGGGATAGCTAAAGATTGGACAGGAGATGGATTTTCAAATTGAGCATTTTTAATATTTAATAATAATACTTGATTTAATCCAAAGTCATCAAAAGTCATAATTGGTTTAATACAATCAACATTATTTGTCAAAATAATATCATTATAATCTTCTAAACTACTAATATCATTTTCATTTGATCTATTTTTATCATTCATAATAAGGGTAAAACTAAATTATATTATAATAATAAATCATGTCAATATATTTTATAAACGCTTATAATGTGATACTTCATAATATCTTTCTACTTCCGCATAATCTAATGACTTAATAAAATTATAAACACTAGTATCATAAAAATTTAATTGACTTCTTGGATCAGTATATTTTGTAGGTAATCCAGTTATATCACATACTTTAAAATGATTTAAACCAATTTTTACTAAATTAACATAATCTTCTGTGAGAATTTTTTTTAGCTTAAAATTCATTAAGGGTTATCAAGCTTATTATTATTATTATTTAATTAATAATTAGATTTTATTATATGCCATTGTTTGATTATTAAAATTATATCAAATTTTAATATTAAATTAAATCGAATTGTTTTTATATGTATAAATATTGATAAATAGTATTAAATTAAAAATAAAATTCTATATTATTAATATACTTATTTTGCTAGAAATAAAATATAATATTTTAATGATATAATGATGTATTTATATTTATTTTTTGTTTATTAGGGATAATATGTATTATATTCTATTCGATTTAATTTTTTCTGATTCAAGTCAAGTATATGAAGAGTCTAATCATACATCTGCTGAATTAGAAAGTATATTATCATCCTCTCAAATGTCATCAAAAAAAACGCGCTCAAAACGCAATTACAGTGTTTCTGAAAATCCATCACTTAATAAACAAACAAGGCAAGTCACTATAAATGAACCATCTGTAGAAGATTGTAGAGAAGCATTTTTACAAGATATAGAAAAAGAATTATGGAATGATAATACATTAAAAGATATACTTAATAGTAATCAAAAATCAGATAATAAACAAATGACGTCTAATGCATCACATACTCCTATTGTTATTGAAAAAACAGTTGAACAAACAATGAAACATAAGGTTGAAGTATTTGTTGATTCATTACCAAATACACAAACTTTTAAAAATATATTAAATATATTTGATGATATTGATGATGATATTAAAAAATTATTATTGGGTGAAAATACTATTGTTCGTAATACATCATCCCCATCATCAATTCCATTACCTTATAAATCACTTGCATCATTTCAACCACCTGTAACTAATGCATCTAATAATGTAAATAATACATCACCTAATGATAATGTAAATGATGAATATGATGGAGGATATATTTTACAATCCATCGAAGAAGAAGATAAAGAAATACCAAACCCAAATAATCATGAAAGTAGTAATATAGAAGAAATTGATGTAGAACCATTACCATCATAAAAGATAATTTCACCTGTGATTATATTATTTAAAAATTTTATTGATTAATAATATTCTAATTTTTTAGATTTATGTCTATATAATTGTTTTATGATTATCATTAAAATACTAAATATTATTAGTCCTCCTATATAAAATTTTTTGTATGTTTTTCTTGGTGGATATACTGGTAAATTTTTATTAAAATAGTTTTCTACAATATTTAATAGTATTTTTTTAAAATTAGAACTAGTAATAGATGCTTCTTTATAATACCATTGTGATTCTAATGGATTAAAAATTACTATTCCAGGAGCTTTGACATCTTTAATAACTGTATTTTTAAATAATGGATATTCGTCCACATCTAATGTAGCATATACTACATTAGTATAAGGTGTTCCTAAATGTCTATCTGTAGCAATTTGGTTGTATTCCTTTAATAATTGTTCTCCAGATGTTACAAATAAAATTACTGGTTTTATTCCATGAAATATAGAATAAAAGTTTTCATTATTAACATTAACTACATGTGGGAAATGAGTATGGAAAATCCACTGAATTATTTCACTATTTTCTTTTAAAGAATATGGATTAGCATAAAATGATCCACTTTTATACACTATTAATTTAACCATTTTATTATGATCTTCGTTATTTTCTTCAGGTATAAAACCTGCCATTTTAAACATTTTTTCATCATTAGATCTAAAAATATTAGCCTTAAATTTAAATTGCTGTGCTAAAGTATTAAAATAATATGATGCAGTATCATAATCCTTATATAACACAACATAAATTGGTTCTCCTATTTTATAATTTTTTGATTTAAATTTTAATTCTTCATAAGTAATATTCTTAAATGATGGTTCAATCAATTTGTTAATAAATTGTTCTAATTTGAAATTAAAGTTTTTATCGTCGATAGATCCCATAAATGGAACAAATAATCCTACATTAATTCCAGTTATATGCGGATATTTAGTAATACCAATTTTTTGTTCAATATTTTTAATTTCATTTTTATGAATTTCTCCAACTTTGAGATTTGAATAGTTTTTTGCTATTTCAATAAATATTTTTCGTAATGGATCATTTTTGTTTTCATAAAATAGTACTAGCCATTCACCATCAAAGCCTGTTAAAAAATCATTATTTGTAAGTTGTGTTACCTTAGACTCTTTAAAATCAATAATATTATCTAATAATAAACTTTTTTCAATATTTAATGTTCCAGCTACCCAATGAGCTATATCATTATATTTTTTGTATCCAGTTGATTTACCAATTTGTTTTTTATCTTCTGTTATTTCAATAGTTGGAAATGTTTTAATATTATCACAATCACAATTATTACATACTACCTTTCTATATTTTATTCCTAAGTCTGTTTTTTCTAATTTAGAAATAAGTTCTTCTATTATAGGATTTAATCGAGTACAAGCAGTACAATTTTTAATAACATAATGATTAATAACGGTTCCAAGCATTGGAGGAATACATTCTATACTATCATTAGATTTTATAATATGAAATAAAGCAAATATGTTATTCATTTATTATTATTTATAAAATGAAATAATTGTTATATAATAATGTTTCATATTATTTATTTATATTATACAATACCACAACATAATGGTGAAAGAACACCTACAACTTTACAAGTATCAGCACATGTATTACAATTTGCTGTAATTGAATGTGTTCTAGTAGAATATGCATTATTTTTAATCTGCCTTTTAGTTTTACCATCTTCTATTATTTCAAATGTATCTGTATTTTCAAGTTTTTCTTGTTTTGTTGTACATGTACATACATAATTTGCTACTGGACAAAATATATTCTTAAGAGTTGAAAAACATCCTAATATACCACAACAACAACTTTTTTGTTCAACTTCTTTTGAATCATTTATAAATTTATCTGATTCATTATCATCTACAACAATTATATCTGTATTGGGATTTGGTTTAATATTATTTAATGAAGATATTTTATTATTATTAGTATTACTACAATAACAAATAGTATGTTTATTTTTGTCTGATTCAGCCTCAATAAGTGTAATAGGAGTAGTATTAATTCTTTGAGTATCTACAGTATCTGTGGAATCAGTAGATGCTATATCATTAGTATTTATAGTAACTAATGTATAATAATCAGTTACAGCTGCAGAATTCGGATCATTTGTTGTAGATGATTGATTTTGATCATTATTATCTACAACAATAGTCGGTGTAGTCAAATTTGTGAAACTAGTAGCAAGATTTAATGATGGGTTATCTAATATTTTTTCCATTTCATTACTAAATGGATCTATATCATTATCAGATGGTAGATGTGCTATTGGAGATGAAGATGGCCTAGGTTGAGTTATAATTGGATTAATAGAGCTAGGTTGAGTTGTAATTAGATCAATAATTTGTGAATTATTTACTTCATCGGTATTATACGCAAAAATATTCAAAAAATATATATCCAACATGTCAATATTTTATTTGTTTTTTAATTAGAATTTGTAGTTTTAATCAATTTATTAAAATCATAATTATAAAATTTGTAAATTAACACCGATAGTAAATATTAAAAATCATTTAACATATAGTTTATAGTCTTTAATTTATAAATAAATGTAATTTAATTGAAAATAACAAAACAGATGATGAACAATTTTTATTAATTGCTATAATATTATTGAAACAATAGTTTATTAAGAATAGAAATATTAATCATCTACAGTACTATTATTTTTATGTAATTTATGTGGTATTAAACAACACCATACATCTCGTGTCTTTTTAATCCATTCACCTTCAATTACTATAACACGTGTAGTAGATTTTGTTCGTTCAATTTTTGTTATAGTAATATTTTTTGCTTTATTATTATTTTTTTTACCTGCAAAAGTTTTTGCTTTATCTTTATCATTACGCGTGCCTTCTTCAACAATAAGTTGAGTATTTATAACTGAACTGTTATATATAATATTAAAAAATAATAATAATTCGTGAAAACAAAACATTATTGTTTAACATATCAATTTAATAATAAATATTTGATTAAATAATATCAACAATCAATAATTATTATTTAAATATATAGAAATTATTATATTATACTCTATTTATAAAACTTATGGTGTTTTTTTTGAAAATAATCCACCAAATCCACACCACTTTTTTTTTTGTTTTTGTGTCACACAAGTTGATTGCGTCTTTATTATAGTACTATCTTTTTTAATTTCAAATGTTGTTACTTTAACTTTTGTTAGTTCATTATTATCACTAAATCCTATGAGACCTTCTTTATCATCGTCATTATTAGTAGTAGTACTAGGAGCATTATTTATATATGATGTAACATATTCTGTTAATTGTGCAGTATTCATATTTGAAATATTATTTATTAATGATTCTCTATTAAATTTTTTTTGTAACTTTGTAGTTGATACTTTTTCAGAAAACTTTTTTATTTTAATGTGAGTAGAATCATAATTTTGAATAGATGGATTACTTACATAATTAGATGATGTATTTGTAGCTATAAAAAAATGTATATACAAATAAAAACAATTCATTTAAAATGTTAGGTTAAAATAAACAATAATTTACTAATTAACAATATATAATTAAAAATACAATTTATTTATTATATTAAAATATTAATTGTTTTAATTAATAAAGAATATCGTTGTTTATGCTCCTATATCATCTTTCATTACTCCTTACATAAAAAAATTTCCAAAGAGGGGTATGCACAAAGATATGAGACATGCATTTAAATCTATGGAAGATTCAGAAGAAAATATTAGGTCCAATGAGAACAATTTTAACAGTATTAGATTAAAAAATTATGGAGATGATGAATTTTTAACGCCAACACTTAATAATGATGAAAATTTTACTAATGCTCCTAAAAAGAAGAAAGATAAAAAAAAAAGAAAATTAGTTGATGAACCTAGAAAAATTAGTAACAGAAAGGTCGTTAATTTACCAGCATCTCAACCATATATTAAAATTGTGGATGGAGAAGAACGTCTTGCTTTTAAATATAATACCAAAATAAGTGAATCTGCTTTGAATTCTATGCCTAAAGATACAATAGAAGAGAATGAATTTTGTGTCAGATTTGATTTAGATAGTGTTGATATAACCAAATTAAATGAAAAATTTAAAGCAGATAATTGTGTATATCCACGTGCAAATGTTCCATATGAAAGGTATCAAGGAAATAGATGGAATTACGAAACTGAATGCAATAAACTTGCGTGGCAATTTGTTTCATTAAATCCAGTATTACTTTATGGAAAGAAAGGACTGATTCAAAGAGCTGTTGATTCATACAGAAATATTTGTAAAACTTCTAAAGGGTCAAAGTATTTTAAAGATGAAGTTTTTAATGGTGATATCGAAAAAAGAAAATTGCATGCTTCTGTTCCATTGTCAGGTCAAATAGAATATATTATACGTGGAGTAGTTAAGAAATGTAAAATACAATTAGCTATTGATAATATTAATATAGATAAAATTCCAGTAGAATTTATTACAAAATATTCTGTTATGCCAGAAGACTTTGAACCTGAAGATTTCGGAAAAACTATTTATAAATTTAAAAATAAAGATAATGAATTAGCAGTTAAATTAGCATTTTTAAACGTTGAAAATAGTACATTTCAAAATGTTATAAAACAAATTGGAATAACGAGTGCATTAAAACTTGCAGTTAATTTATACAAAACAAAAAAAACTGAATTAACTAATTCTACTGATCAAATGGATGCTGCTGAAATTGTTAATGAAATATTGGATAAAGAATTTAACAATTTAAAGCTTGAAGAAGATAATGATTATTTTTAATATTAACAATTAGTACAATAATTTTTATGAGTTAAATTAATTATTTTTTAATTATCAAATGGAGGAATGTTAAATCTTTGTTTAATTGTTATTCTATATTCACTATTTTTATCTTCAATACTAAATTTTGCTGGAATGGCAACGTATGAAGATTCTTCTTTCATTGTATAAATTTTTTTATTATTTGTGTCTAATGTATAAAAAATCATATAAAAGGATAAAAATTTTTAATTATTTATTAAAGAGTGTCAAAATACGCATCTGGATCCAACTTAGACGTTGTTCCTTTGTATTCTCTAACTTCTTTAACAATTTGAGCCATTATAGAATTTTCTTTTTCCAAACTTCCGGGGCATATTTCATAATGATCAAATACTAATGAAAACGTAGAAAATCCTCTAGAATTTTCACGTAAATCTTTGTTTAATCCAAAGGATGCTTGTACTGGTAAAAATGCCTTTATAGTTTTGCAACCATTTTCAATTTCAAAATCTTCTGTATAACCTCTTCTATTCTTAATCACATTTTCACATGCAGTTCCATATTCTTCAGGTACTGTAATAGTACAAGCAAATATTGGTTCATATAATACTGGAGATGCTGTCAATATTAATCCTCTAACTAATTGTTCAACAGGTCTAACCATTTGATTAGCACCTCTATGTATTGCATCCGAGTGTAATATTAGATCTGTTAGATTAAATCGCCCTCCACGCACTACTTCTCCAATCATAGGTCCTTCTTTTGTTGCTAGTTTTAATCCTTCAAAAATATGATCTTTTACTTCATTAAGATATTGAATACCTTTTGATTCATCAACCATAAGATTCATTGGTTCAATTTCTGGTGCAAAGAACATAATTTTTTTTACCCATTCATCTTCAATCTTTAATATTTCCCTAAATCTTTGATTTCTAACCTTTGGATCCTTAACATTAACTTGTTCTATATTTTCAAGAATTTTATCATCTAATGGTTCACAAGTCATATAGATTCTATTATGCTTGTTTGCTGATTTAGTCATTTTTGGTGATTCGACAGATCCAACAAATCCTTCATAATATTTAACTTGAGGTTTTTCAACTATAAATTCACACTTAGCGTAAAATTCTTTCAAATCCGATAAAATAATTTCTAAATGTAATTCACCAGCACATGCTACAGTAGCTTGTCCTTGATCAGTATATTCAATAAGACATAATGGATCTGATTTAGCTAGTTTCTCAAGGCCTTCTTTAAAATGATTTAGATCTTGAGATCTCTTTGGTGTTACAGCGACTTTAACAACTGGACTTACTGAGAATTTCATGGTTTTAATATTGTATCTATCTGGAACAGATGAAATTGTTCCTGTTTTCTTTAAAAAACCTTCTACTCCTATTAATCCAACTATATTTCCAGCTGGACAATCAGGAATACTATTATACCCTCTACCCATCATGACACAGACCTTAGAAATAGTTTTAATAACAACATTTGGATTTTTTTTATTTTCTGGATCAGGTACATAATTAGGATTTTGAACATAAACTTTAATACCTGGTTTAACATTACCTGAAAAAACTCGTCCAAAAGCAATGAATCCATTGGAACCATCAGGAATCATTTTGGAAATATAAATAGTAATTGGATCTTCATCATTAGCACTACAAAGTTTAATTGCTTTAAATGTTTCGTCTTCCGGACCAGTATATAAATAACTGGCTCGAATATTTTGTGCCTTATGTGGATTTGGTAATTTAAGAATAATTTGTTCTAAAAGACATTCAGCAGCAGGTAACCATGCTTTAAATACTGTTTTAAAGAGTACTTTCCCAGGCTGTTTTCCTTCTAAATCTAATTTTTCACAGTCAACACCGAATTTCAATAAATATTCTTTAATTTCTTTTGTTTTTCCTTCAAAACACCAATTTTTAACTTTATAAATTGGTCCAAGTACAAACATTTCAAATGGAGATTTTTTACCCTTTGGAATAACTCCATCTACAATTTTTTTAAACTTGTATTCTGTTGTAAACTCATCATCAAAATCACAATAATGTTTCATGCTCCATAAAATATCTGTGAATTTTTTTTCTTTTTCAAATGTATGATCTCCTCGATATTTAAGATAAAATCTTGCAAATTTGTTTAAAGTAAATCCCCATCCTTGTAATCCAGAGCAGAATGAAATTTCATTTAATTTTGGTGATAAAGATTTAATATAATCACGTTCTCCTAATATAGTTTCCATTTTAGCATTAAAAGCTTCAATTCTTTTTTTAAGAACTTCATATATTTCTTTTTCAGTAAATCCTAATTCAATAATTGCTCTATCAAGTTTATTAAGAACAAGTGTAGGAATTATTCGTTCATCGATAGCCTGTCTCAATACTGTTTCTGTCTGTACACATATACCATCAACACAATCAACAACAACTAAAGCACCATCTGTTACTCTAAGTGCTGCTGTAACTTCTGATGAAAAATCGACGTGACCTGGTGAATCAATTAAATTAATTAAAAATTCTTGCCCATTATAATCAGAAGATTGTGCATAAGCTTCTAATATTTCTTTCTCAACACTAAAACTCATGGAAACAGCTGTTGACTTAATAGTAATACCTCGTTCTTGTTCATCTTTTCTTGTATCCATATATCGTCCTCCATTGCTTTCTTTAGATGCAATACGAGCCTTAATTAACAAACAATCTGTCAATGTACTTTTTCCATGATCTACGTGTGCTATAACTGAAATATTTCTAATATTTTTTGTATTTCTCATTAATTCATGAATTTTTTGTGTTTGATAATCCACCATTTTGTGGGTAAAATTTTTACAAAATATAATAAATTAATAGTAATGGTTTTTATTGTTTATAGCCTAGTGACTAATATATTAAAATATTATTTAGTTTATTAATTATAAATTGATATTTATTTTTATATTTATTTTTAACCCATAATAATATATAATGCAAAATGATATTATAGCATTAAATAGTTTCGAAGTAAATTTTTCTGGAAAAATTGCAGTTGGAACAACAAATAATGGAATTTTAAAGATATTTAAATTAGAAAAGGAACATATTGATTTATTAACTTCTCTAGAAGGTCATACGGCACCTGTTGTAAATGCAAAATTTATGAATAATAATTATATAGTTAGTATAGATTTTACTGGTGGATTAATTATATGGGATAAAGAAAATAATATGTATTGCAAAAAAATAGTTAAACAAATAATTGATGGTTCTATTTATGACATGTCAATACTTGAACAAATTTCAGGTAATTATTTAGTTTTTTGTGCTTCTAATTGTGGAAAAATTATTATAACTGAATTTTTTGAAATTAATGAAATTAATTCTTGTACTATTCAAGTACATGAAAATGATATTATAAGTTTAGATAATAACACAGAATTTCTTGTTTCAGTTGATTTAGATGGTATTGTTAAATGTACATCTATATTGGGAAGTAAATTACAAGACTCAATAACTATATATAAAGTAAATAATGAGCCAACTGTAATTAAAATTACTCGAAAAAATTGTTTTGATAAAACATTTATTGCTATAGGTACAGGAGACGGAGTAGTAACTATTTTAGAACGTGATATAGATACAGCAGAATTTAAAGTTAATAAAGTTATACAACTTGAAGGACCAATTTATTCTTTAGTATGGTCTAAGGGTGGTTTCTCTTTATCTGTATTATTTAGTAAAAATGAATGTATAAAAGTATTTGAAATAACAGAAGAAGGATCATTTGAAGAAGTAGAAGTAGAAGACTATTAAATCTTTTAATAAATAATTATTATCAAAGAAATAGACTATTTTTAATATATTGTTTTTGAAACATTGTTACAGAAGTGTTTAATAAAAACTTCAATTGTTTAATAGATATGAAACAGCATCTTTCAATTTTACTTGTATCCTTAGGATTAATTTGGACAAATGATGAAGATGTACAAAAGGGTAATTTATCTCATGGTCGATCTATGGATGGAAGAAGTGGAAAAATTTTTGGAGAAGATAAAATGAATACAAATAAAAATAGATATGAGGAAAATGTAAGAGTATTAAAAAATGCAGTTGAAAGAGCTGCTCAAAACATTACATTGAATGAAAATCAAAAATCAAAAATTGAAGCGCTAATTAATAAGGCAGCACAAGGAAATCATTCAGCAATTGATAAGTTTTTTGAAAAAGGAATTATTAATAAGAATGAATTAGAACAACTTGAAAAATCAATGTTTTCAAAAGATAAAGCACATATTACTGAACATTTTTTAACTCCTGATGAAACAAAAACAGTTAAAAAAACAATAGAAGAAGAGACAATAATGTCCCCTAAGAAAGAAGATGTTATTAAAAATAATTCGTCTTCTCAATCTAAAAATATTAAACCTACTAATACAGCACTTAATGATAAAAAGTCACATACAATGCCTCAAGAATCATTTACACTAAATACAAAACCTGGTACAGTAAATTTAAATTTAGATCAAAAAAAAGCAGATTTAGCTCAAAAATTATATGAAAGAGAATTACATGATAATGTCAAAAAATATGCTTATGAAAAAAGTTTAGAAAATGGTTTACTTCCGGAAGAAGCAGAAGCTAATGCAGAAGCTGCAGATGCATACATAACATCATATCAAGAACATTTAAAAACAGGAATGACACCAGAAGATGCCGATAGATTAGCAAGAAAAACATACAAAAATAAAAGCATTATTGCACCAAACGGAGAAGAGATGTTTGAAGATATAATTATTGAAAAAATTAAACAAGAATTTTATGATGAAACATTACCACTTGGTACACTAGCAGATCAATATAGTGCGCATCAATTAAAAAAAGAAGCAGAAATTGCAGAAATTCAAGAACAAATTAATTTAAATAATAAAAAGGAAGCAGCACAAACTGTAAAAAAAGCATTAGAGGAAGATAAAGAAATTAAGCAAAAAATTCAAAAAAACATAGACGATGTTAAGGAAAAACTTAATGAAATTAAAGAATTGGAAAAATTGACTATTGAGGAAAGAGAAGCAGCTAATAATAATAAATTACCTGATTTACAAGATTCACATATTAAAACTGAAGTTGAAAAGAGACAAGCAATATATCAAGAATTAGTTAATAATGTTAATGAAACTCCAAAAGAACAAACATTCCAGGAATTAAATTTAAAATCTCAAGAAGATAAAAAACAAGAAGATACATTAAAAAGAACTTATGAAGAATTTATTAAGACACAAACTGAAGAAATAGAAGAAGAAAATAAAAAGAAAGAAGAACGTAAAAAAGAACTTGAAAAAGAAGAATCAACTTATTCACCTAAAACTAAAACATCTGATGAAGTTCATCAAATAATTAATGAAGTATTGACTGAAACTACTGAACAAAGAGCACAAGAAGAAGCTGTATTTGAAGAACAAGTACATGCAGTTGTTCAAACAGCAGGAGAAAAAGCATATCAAAATGTTATTAATTTACATGGAACAGAAGAAGAAGCTAAGGCAGCTCAACAAGATGCTATAAGACTAAAAGAAGAACAAATTAAACAAGAAGTTGAGACAGTTGTATCAGAAATTATTGAAATAATTGAAGAACAAATGGACCCTGAAACTGCAAAAGATGTAGCACACCATATTGCAAAAGTTCATACAACAGAAAAATTATCACATTTAGAATCACCAAAATCAGAAGAAACTGCAACAACTTCACAGTCTAATTCTAATACTACTACAACTACTGAAATTTTAACTGTACTAACAGACGATGCTAAGAAAGGAACTATAAGTAATGGAGTATTAAAATTACCACTTCCTAAAGATATTGAACCTACACCTAAAACATCTGAAGATCAACAACATTTAGAAACATTAGAAGAACAAAGAGAAAAATATAGAAATAAAACATATAGAATTCTTGATTCATCTAAACCATTACATACTGGATTACCAACAATACAAAATAAAGATGGAACAGTTACAACTTATAGCGAAGGATTAGAATCAATGACTGTTGAAGAAGGTATTATTCAAATGACTGAAGAAACAATAATGGAAATGAAGGAAAAAGGTTTAGATACATCAGCTTTCAAGCCAAAAATTACTGATAATGGAACAATTTATGAATTACCACTTGAAAATGCGATAATTGAATTAGGAAATGATGGAAATCGTTCAGGAGTCAAAATAAAAGTTGAAGATGAGGTTATTGATATAGGACGATTCCCAGCAGAAGTAGAAATTAAAAAAGGACAAGCAAATATGCAACCATTTGAGGAATATAGAAAATTATTAAGACCTGAAATTCATGGAAATGAAAATCCTACATTAGATGATGATAATGATCCTGTTGAAACAGAACAAATTGAAAAATTTGCAGAAATTGAGCATGAAATTGCTAAAGAAGAAAGAACTAAATTAGAAACATATCCAACAGAAGAAGGATCTCCAGCAGTAACTATTATTCCAGGATATGGAACCTCAACATCAGCAACCCTAGATGAAGCAAAAAATAAAGCTGATAGAGCCAGTAAAATTGATTATGATCATATATTAGAAGAAAAAACAGAAGAACATGTTATTTTTGATATGGCTGAAATAAAACCAGAGCCAGCTGATGGTGTTGAATCATTCTTTGATAAAATTAGTAATAAAATGCAAGAACCACAAAATGTATCACCTATACCATTTGTTGTAAAAATGGAAGATCCGATCCCATCAAGTAATAAAATCATAAATGAAGATTCAGTTCAACCATTTGGTTCTCAAACAGTTACTACAACAATTCAGCATGAAATTATGCAATCTGATCCACATAATAGTAAATTAGTTCCTAAAAACACAACCAAAGGACAATCATCAACTGGTCTTAATTCAAATAATCCAATTACTAATCAAGTAAATCCAGAAGTAGAACAAATAACGCAGCATATAGAAACTATAGAAGAATTTATTACAACGCATTTATTACCTGAAACAAATCTAGGCAATAAAGTCTTTAATAAGTTATTGAAAGACGTTCCAAATATAGTATCTAAATTAGGATCATCAATCTCTCCAGAAAACCTCATCAAACAACTAACTACAATTACTCCTGAAAAATTCGAACATGTAATACACAATATTACTACAGGAGCACCTGCTGAAACTATAAAAAAAACTATTCAAACAATAGTTGAAACATCATTTTCTCCAGAAATAGCACCAATAATTAATGAATTAGTAACTGAAACCATATATGATACTATGCAAGCAAGAAATCCAAATTCAACAATTATTAGTACAGTACCAATAAATGGAAATAGTGTAATTAAGCAAAATCCAAATGGATCTTCATCATTAATTTATGGAAAGCCAGGATCATCTACAGTATTAAATACAACTTATTATGATAATGTTCCTACAATTATTCCTAATCCACAATTAAATCCAAAACCATTTATTGTTCAAGGAATTGAAAATAATAATCGCCAATCACCATTATCTAGGCCAGAATCACAACTGCCAAACTTAAATTCTAAACCTACTAATTTTATTTCAACAAATAAAACTACCACTCCAACTTTACTAAATAATAATCAAGGTGTAACACCTAATAATAATAACTTTGTTCAACAAAAATTGAATCAAAATCTTTCATAAATATAATAAATGAATATAATATTTATAATTTTTTTATTTAAAAATTTTACTAATATTAATATATATATATGTTATACATAATTGGTACTGGACTGAATGATTTTCAGGACATTTCGTTAAAATCTATAGAAATATTAAAGACTTGTGATAAAATTTATAGAGAAAATTATACATGTACACAAGCTGATAGTTTTAAACAATTAGAAAAAATAATAGATAAAAAAATTATATTGGCTAATAGACTTTTGATAGAAGAAGAAACAATGGAGATAATTAATTTTGCACAAAAATATAATGTAGCTATTTTAGTTTCAGGAACACCGTTATTTGCCACTACACATACAGATTTATTAATTCAAGCTAAAAAATATAATATTCAAGTTAAAATTATTCATAATGTATCTATAGCATTAGTAAAAGGATGTTATGGATTATATTCATATAATTTTGGAAAAACAGTTTCTATATGTTGTTTTACAGAAACATGGAAACCAATTTCATTTTATGATAGTATTTACAAGAATTATATAAATAATTTACATACTTTATGTTTACTAGATATTAAAGTTGATGAAAATAAATTTATGTCTGCAACAGAAGCATTACGCCAATTACTTTATGCTGAAGAACAAACAAAATATGGTTTAATAACACCTGAAACAAAAATTTTTGTTGTATGTAGATTTGCTACTGATACAGAAAAAGTATATTATAATATAATAGATAAATTATTACAAGAAGATTTTGGAGAACCATTACACTCTATCATATTTCCGGCTAAATTGAGTCTAATAGAAAGTGAATTTATTAATTATTTATATCAATAAATATTATATTAATGGTATATTATTATTAATATCTTTCAAATTAAATTATTTTTATCCCTAATTATTAATGGAAACTAGCATTGCTATTAATAGATTTAAAGATTATTTAAATGCTAAGTTTGAATCCAATTATAAATCTCCGGATAATAGATTAATTATTGATGTAAATGATATGAGAGAATTTGATAAAGAATTAACTCATTTTATTCTTTCTGAGCCAATGAAAATTATACCAATAATGCAAGAAATAACGGAAGATGATATTAATTTAAAATTTGGATTTATGGGATCATTTGGAAATAATTTAGTTACACCACGGCTTATAAATAGTAATTTTATAGGAAAAATGATTTGTTGTCAAGGAATTATAACTTCAATTAGTTTAGTTAGACCTAAAATTCAAAAATCAGTTCATTATGATGAAAAAACAATGTTATTTTATCAAAAAATATATAGAGATAGAACAATGATAACTCATTTGCCTCCAACAACAACATCTTATCCAACGATTAATGAGGGAAATATTCTTACATTTGAATATGGTTTAAGTAGTTATTGTGATTTTCAAACATTTATATTACAAGAAGTTCCAGAAAATTCCCCACCAGGACAATTACCTCGTAGTATAAAATGTATTGTTTCTGATGATTTATGCGATACTATTAAGCCTGGAGATAGAATTAATGCATATGGAATATATAAATCATTTGTAAGTGATTGTTATAAAGAATTTCCACAAAAATTTCAGACAGTTTTGATTATCAATAATATTATTAAAATTAAAAATGAATTAATATTTAATAAAACAATAGATATTACAAAACTTACTATGGTTTCAGAATCAATTTTAAAATTTAAAGCCGTAGCGCCTACAATTTTTGGACATGAAGATATTAAAAAAGCATTAGCTCTTCAAATGGTGGGTGGAAATGAAATTATATTAAAAAATGGAGCTAAAATAAGAGGAGATATCAATATATTACTTATTGGAGATCCTTCAACAGCTAAATCACAACTTTTAAGATATGTTATTAATTTTATGCCATTAACAATAGCTACAACTGGAAAGGGATCAACTGGAGTAGGATTAACAGCTGCTATTATTTTAGATAAAGAAACTGGAGAAAAAAAATTAGAAGCTGGTGCTATGGTTCTTGGAGATAAAGGAATTGTTTGCATAGATGAGTTTGATAAAATGAATGAGAGTGATAGAGTAGCTATTCATGAAGCTATGGAGCAACAAACAATTACTATTGCTAAAGCTGGAATTCATACAACATTAAATGCACGTTGCTCAGTATTAGCAGCAGCTAATCCTATATTTGGAACATATAATGAAAATTTATCTCCTCAAGATAATGTTAAGTTACCAGAATCGTTATTAACCCGTTTTGATTTAGTGTTTATAACACTTGACAATAAAGGTATAGAAATAGATAAAAAAATTAGTAATCATGTTTTAAAAATTCATTGTGGAATAGAAGATGAAAATGAAGAATTTATTTCACAAGAACTATTTAAAAATTTTATTCTGTATGCAAAACAATTTAAACCCAAATTATCTAAAGCGGCGGCATCTATTATTAGTAAAGAATATTCTAAAATACGCGAACATAAAAATGATAAATCATTAATTGTTAATATAACTCCTAGATTATTAGAAACAATTATTCGGTTATCAACTGCATGTGCTAAGTTACGATTAAGTGATATTGTATTAGAAGAAGATGCATTAGAAGCAATACATATAATTAACAATAATTTAATAAAAAAGAAAATAAAAAGACCTATTTGTAGTAAAATATCCACTTTTCACGAAGATATTAGTAGTGAAACTCAAGATATGAAAAATCAATTAAAAAAACAACAATTATTTAATGCATCTGTAATAGAAAAGGAAAATTTAGATGCTATAAATAATTCATCTAGAGACGAAATTTTAGATTTAATTTGGACTTGGAAAGAAAAAAATCAAGATGCTGAATTTTGTGATATAAATATATTATCAAAAATATCCAATGTTAATTGTGATGCAATAAAAATAGTTGCTGAAGAATTAGCTGAACAAGATATAATAATGTTTGATAATGATAAAATATATTTTTTAGATTAATTGCAAAATACATATATCCTTATTTAATGAAAATAAAAGATAGAATAAAATATTTAAAGCATATTAAAAAAACATCATTTAATATTTATTGGACAGAAGAAATTATTAAAACATTTTTATATTATGAAGAACCACAACAAGCTAAAAATTATTTAAAACATTTTGAATTTACTAATAAACGATGTAATTATCTTCATCTTTATATTAACACATTTGAAAAGTTAGCCTTAAATAAATTTGATTTACAAGATATTACTAATAATATTGATGATTATTTATCACATTTAAAATTATTTATTCCATTAATAGAACACTATCAATATAAAATTAATTATTTGTTATTAGTACAAAATAATTTATCAGCATTATCATTAACCAATTTATATTATCTTTATGAAAGGTCTAATGCATTCGAATTTTTAGAAATGATTGTTCAACGTGATCCATTCATTAATAAAGATTTATATAAAAAATATTCTGTAAAAAATGGAGTTAATAAAAAAGATATCTTCCGAATATTAATGGTTCATAATTGTGATTGGGCTATGTGTATAGCAATACGATGGAAATGGATACCAATTAATTAAATTATATTTATTTTAACAATTATTATATTTTGTAATCGTATTAATTCATTTCATCACGCCATTCATATAAACAAGATGTACATATATAGTGAGTATCAAGTGCTTTTATTTCGCTTTTAGTTATATAAGAAATACAATGATCATTTTTGCATTTTTCGCATATTTTATGTAATTTTGGTAATGTAACATCATTTAAAAAATATTTTTTATTTTTATTTTCCATATTTTTATTCTTATATCTATTAAATATTATATGTGTATCACAATCAATTACAACATTACATTTATTACATACTAATTGTAATATATTATTTTCGTATCTTGGATATAAAATATTATTACACTCTAAACAATATTGAATATCCATAAGGGTTACAGAAATTTAAATAGTTTTTATAAGAATTTTAACATATTATTTTATGTATAATTTATTGCTAATTTTTTTTAATAATTTTTAAAAATTTTTCCAGCCCTGTATGACATTTATTCAGATATTGAGAGAAATTCATCAAAAGAAGCAATCAGATATCATGAAATATATGACAAGAATAACACTTGTTGAAGCTCGTCAAAATGATGTTGTATTCCGAAAAGAACGACCATCATTTATTGATAGAGCTCGTAAATTAGGATATAAAGCTAAAAATGGCTATTCTATTTGGTCTGTAAGAGTAAGAAAGGGAGATGCTATTCGAAATTATAACAATGGTAATACCAGAGGAAAATGTGTTAATGCTGGTATTCATCAAATTAAGCCAAATTATAATAAACAAGCAGAAGCTGAACAGTTAGTTGGTAGAAAGTTAGGTAGTCTCAGAGTATTAGGAAGTTATAAAATTGGACAAGATCTCAGATATCATCACTTTGAGGTAATTATGGTTGATCCTATGCATAATTCTATTCGAAATGATCACAAAATCAATTGGATTTGTAATCCTGTTCATAAACATAGAGAAATGCGAGGATTGACATCGGCTGGAAAAAAGAGTAGAGGCCTAGGTAAAGGAATTAAATATAATAATACAAAAGGTGGATCATGTAAAGCAGCATGGAGAAATAGAAATACATTGTCATTAAAACGATATAGATAAAATAAATTATTTATTAAAACAATTTTAAAATCGTTTATAACCATTATAATAATTTGGTTTTCTTCGTTTGGAATGTTTATAATCATCTTTGGAGTATTTTTTATTATATTTATCATCATATTGTTTATTATGGTTATTATAATCAGTGTCCTCACTATTTTTCGAAGTAGTATAATTTTTTTTATTATAATTAGTTTTGTTATCATAATCTTTAGTATCTTGATTTTTTATAGTATCATTTTTTATAATAGTTTTACATTCTTTGAGCGAATTATCTAAATTTTTTGTATCTTGATATTTTTCTACAAATCCTCGCCTTTCTTTCTGCATTTGACAATATTTTAGCCATGTTTTTTCAGTAAATCCATAATTAAAATAATCTGTTATATCTGCTCCGGGTTTAAGCCATGGTTTATCTTTAATTTTTTCTATATCAACATTATATGCATTATTATCCTCCATTTGTTCAATTTGTAATTCTCCTTCTTGTGCCTTATCTATAATCAATGAAATATCGGATGAATTTTCATCGGATTCATTAGAACTCAATAAAAATTCATTTTTATCCATTTTAGGGTAAAAATGATCCTTACAAATGGAAAACGTTAAAATTTTATTTGCCTTTTTAATAAATTATTTAAATCATGATGAAAATAAATTAAAACAATTTGGATATCAAGTAGGTCGAAATATGTTAATTTTAAATCGATTTGATAAAGATAATGATATAGAAGGATTATTATATAAAATAACATTTTCATATTTACCTCTTTTTTATAATACAACAAGATATTTAGAAAAAAGAATTGGTGGTAATTCTACAAAATCACATTATTTAATTATAGAACCAAATTCACTTTTTAATAATATTTGTTTAAATAAATTAGACGAAACATTTTGTTATGAAACGATTTTTGCAGGGGTTATTGAGATGATACTAGCTGCATCAGAAGTTAGTGCTGAAGTAACCGCCCATAATATTCTTCATGAACAATATAATGAAGTAATTTATGAAATTAAATTGTTTTAAATATAAAATATAATACTCTAAATATTTTTTAACATTTAATAATTGAAAATAAATATTTGATTAATATATAAATTAAAAAGAATTATTTAAATTATTATAATATTCATTATTTAAATCATGTTGTATATAGTTACGCGTTATTCTTCTTTTTTTATTAATTTTACGCACTATTTCTTTATCATCAACTATAGACCAAAAACCACCCTTTCCTTTTTGATTAGCTTGTCGTGGTGTTTTTTTAAAAGCAGCATTTAAAGACAAATTATGTCTTATTGAATTTTTCCATATAGCATCCGCTGTTTTGAAATATGGATAGTTTTGTTCAATCCATGTATAAATTTCACTAAGGGTCATCATATTATTTTTTGACGATGATAATGCTCTTAATATCATATATGCATATGAATCATCAGGTTTTTCATTTGTCTTGTTAGTTGCATATTTATTAAATTCGGTTTGTAGTGATATATTTGAATTATTAAAATTTATTTGATTATTATTAAAATATGAAATATTATTGTTTATATTATCTCGAATAAATAATGAATTATTATTAGTATGATCTAAATTATTTTTATAATAAGTTTCTGAATTAATAAAACTATTATAACAAGTATCAAATATTGGAGATATATATTCTTTTTTTTCTTCTTTATCCATTAATATTTGACATAATTCTAAATAATTATCATCTGATGGTATTTTAAATATATCATTAAATGTTTCGTTTGTTACTTGATTTTTTTTATTTTCAGGTTCCTTAAGTTTTTGCGAATTAAAAAACATTGAGGGTTAAATTTTTAATATTTATTAAAACATATTGTGTAATATTAAAATATATATGAATTAATATTGTTTTTATTCTTTATATTATTCTTTAAACAACAATATTTTTTTACCTAATTTAAAATTAAATCCTTAAAAATTTTTGTAAATTAATGAAAGTTAAAAATGATAATAAATTTGATGTTTATTGTAAAATTATTATTAAATAATTATATTCTTTCTATTATTTTTGTTTTAATATTAAAAAATATTTCATCATCTCTTTCCATTTCATTTAGTACCTCTTTTAATAAATTTTCATCAATATTATTACAATATGCTAATAAATCATGAAATAATATTTTCTTTTTAGTATTTAATATATCTTTAATATTTTTTCTTAAATCATTTATTAACATATTTTTAGATTTTGTATTACCCGTCATTACCATATTCATATCAATTTTTCCAGTAAGTGGATCAACTGCATATAACAATAATGATTCTTTAATAAGACGAATAGCTTCCATAACATCTATTTTATCAACTGTTTTACTAAAACGCATTCGAGCATGTGCTTCACTAAGCCTAATTAATGATTCAAGTTGTCGAGTAGTTGCAGTTATTGAATTTCCATTATCAAGCTGTCTTAAGTCTATATATGCATTTGAAATTAAAACTTTTGATTCTGAAGTTAGTATAGGATTAATTTTTTTAGCTTCTTTAATATAAGCTTTTAATAAATCAATTTCAATAGTTTCAACATTAGAAGTATTATTTGAAAATAAATCAAAAATGTGTTTTGCAACATTTTCATCATATTTTTCATCAGGTTTATCAATTAATAATACAATTACATCAAACCTTGACAATAAAGTTGGGGGAAGGTTTAAATTTTCTATTATCGTTTTTTTAATATTATATTTTGATTCAATAGGATTACATGATGCTAAAATGGAACATCGTGCATTTAATGTTGTGATAATTCCAGCTTTAGCTATAGAAACAGTTTGTTGTTCCATTACTTCATGTAAAACACTTTTTGTTGAATCATTCATTTTATCAAATTCATCAATACAGCATATTCCATTATCGCTCAAAACTAATGCTCCTGGTTCTAAAATAAATTGGTTTGAATCATGATCTTTTATAATAGATGCCGTTAAACCAACTGCGCTTGATCCTCTACCAGAAGTGTAAATTCCTCGATCTATAATTCGGTTTATAAAAGATAATAATTGTGATTTAGAAATACCAGGATCTCCAGCTAATAATACGTTAATATTTCCTCTTAATTTGCTATTTTTTAAATTTTTACAAACACCTCCAAATAATTGTAATAATAATATCTTTTTAACATTATTCAAACCATAAATAGATGGTGCTATAGAATTAGTAAGAATTTCATATAGTTTTGGATGACGTATAAGTCTGTCGATATTATCTAATTGTTGTGTATTTAAACAATAAGTAATTTCTTTTTCTTGATTTAATTTTTGAATATTTAATAATTCAACATAAATTCTAAAAGAACTTTTAACTTTTCTATGATTTGGGTTTAATTTAACTGGAATAGCTTTTAATATCCCAATAATAATTACTTTATCTCCCGGAACTAATTTATCTACTAGTTCATCTTTTGCAGTAACTGTTAAAGTCATAGGCGTAGTTCCATCAGGAATTTTTTCTGGTAATTCTTGTAATCGTAATATCTGCTTATCAGAATATTGACTTTTATTGTGCTTCAATTCAAATGTAAATCGTCCACCACATTCGCATACTATTGGTTCATTAATAATTCCTTTAATAGATTCAATTTCTAAAACTATATTGCACTTAATACATTTATAAAATCCTTTTTTTATCTCAGGAATTATTGAACTAACTCTGTTTATCATACCAGTTACTTTTACTATGTGATCTATATTTTGCGGTTCTATATTTCTTATAATTATTTCTTTTCCTATATTATATGGTCGAATTTTAATTTGAATTTTTTCAATTGGTTTTCGTTCATAATATATTTCATTTAATGTATTTTCTAGAATAGGCAATATTTCTTGAGGATAATTTTCAATTTGATCATAAATTAACGCATATGGTTCTGTAAGATCTTCACAATTAAAATTTAAAATGAATTCTTTTGTTAACTCCATATCATTTAATTGATTAATATATGTCTCATTTAATCGAATAAATTCTTTAAATTTTTCACTAGTTTCTTGAATATTAATAGATGTTCCCCATATTACTTTAATTCTTTCCAAATCTGTATTTATATTATATGTATTAGATGTATCAATACAAGATTGAGTTACTGTTAGATGAATATCTTGACTTGCTTCATCATATGTAAAAATTTCTGAATGAGAAAGGTTTGTCATATGAAGGCTTAAAAATAAATTTTTACGTTTTATTAATTAAATTTATAAAAAATACTATTAACAAATTGTAATTCATTTTATTTATAATAAAACTTTCCATCATAATTAATATGAACAGATTTTTTCTCTAATGCTTGTTCATATATTTCATAAACAACATTCCAATTCACAACTTCAAAGAAATTATAAATATATGTTTTCTTATCATTTTGATATTTTAAATAATAGGCATGTTCCCAAATATCCATAGCTAATATAGGAAAAATATTTTTCTCATACATTTGTGGATGTTCTTGATTTTTCGTTGTTATTATAGATAATGTACGATTTTTAAGTTTATCAATTCTATTTGAAATTATTAACTCATCTGTAATAGTATTTGTTTCTGAAGAATAACATATTTCCATATCTTCATTAATAAGGCATAACCACACCCATCCTGATCCAATAACACTTAATGCTTTTGTTGCAAAGATATTTTTAAAATTTTCTAAATTTATATATTCATCAGATAATATATCGATAAATTTATTTGTTAATGTATTTTTTGCAATATTATATGGAGCTAATATATTAAAATAATATATATGATTAATATAACCACCTCCCATCAGTAATAATTTTGAAAAATCCTGTTTATCAAATTCATTTTTATTTTTAAATATTTCGCATAATATTATATTAATATCAAAAGTTTCTTGAAGTTTAGATAGTATTTCATTTGTTCCTATTGTATAACCATTATGGTGCTTATTATGATGAACTTCCATTGTTTGCTGATCTATATATGGCTCTAACTCATTATAACTATATAATAATTTTGGAAGTTGATATTTTATATCCTGATATTTTATATCCATATTAAGGGTGAAAAATATAAATTATATATATTAAAATTATTTTAATTGATCAACCAATTTTTGATGATTTTTACATAAAAAACTAACATTATAATTAAATCCAAGCAATGTAGATAATTCCTTAAATACTTCTCCGCCTAAGTATGAGGTAGGAATGTTACTTGCCCAAATTTCTATTTTAATATATGTTTTTTTTATATCTAATCGAATACCATTTACATTTATAGTAATAAATTTATTTGTCATAAAATTAAAAATAAGTTGTTCAAAAATATATGAAATTACTTTGCCATGATTAATGTTTAAAATATAAGAACATCCATTTATGTTTTTTGCATCTTCCCATTTGGGAGTTATTCCATCTTTAAAAATATTTATATTGAATTCTTCTATTACTTGATTATTTGCTTGAACTAAATAATCATCAAAATTTTTTATATGCTTTAAAAACCATAACATTTCTTCAACAGTATAAATAATACAAACTGGTGAGAAACTATCTTCATAAGAACATGTTTTATTAATAAATGTATCAAAATTCGTTGCTGTTACAACAAATGCATTATTTAATTTCATATCGGGGTAAAAGAAAATTTATTTTTTTACATTTATAAATTGTCAATCGAAATAAATACCGTAAATTTAACTAAAATATTTATTAATAGTTTTTATTAAGAGGTAATGTTAAATTAAATACCAATTTTTTTTGGTTATCATTAATTATTGAATCAAAATCACAACAAAAAAATCCAATTCTAATAAATTGAAATTTATCTTCAATATGGGTATTTAATACAGTATTATCTACATATCCATCTATTATTTCCAATGAATTAGTTAAATCAATATCATTCATATATCCTATTTCATCTGGATCAAATGATTTAAATAATGGTTTATACATACGCAATTCAACTTTATTATCTAAATTAGGTAACCATTGAATAAACTTGTTTGGTTTACATTCATCAACAGTTAATAATTCACATCGTATTCCATCAGATTCATCTTTAATATATTTAAGAACTCCTAAATTTATTAGTCCAACAGGCTGAATCTTTGTTAATCTTAAAAAATCTGAATTATTTGTTTCATCAAAATCATCTTTATCTATATAAATAATGTTGTTAACATCAACTGGTTCATTAGATATTATACCTGTTGTACATTTATTAATATATACTTTAAGAGGATTAATTATACAAGTTGCTTTTTTTGCTATTTGCATTAACTCTTTTAAAACAAAACTTTCTAAAATTTTAACATCAATAATAGTTTCACTAAAAGTTATTCCAACAGATTGAACAAATTTGTTGATAGCAGATGCTGGAAATCCTCTACGGCGCATTCCTTTAATTGTATAAAATCTTGGATCATCCCAATTTAATCCTTCATCAACAATTTTTGTTAATTTTCGTTTACTTAAAACAGTATTACTAATATTAAGTCGACTAAATTCCCATTGAACAGGTTCATACATATCCAATTCTTGTAATAACCAATGATATGGTTCCTGTCTTGTATAAAACTCTCTAGAACAAAATGAATGAGTAATGTCCTCAAGCGAATCACAGACACATAATGCGAATTCATAACTTGGATATACTATATAATTTTTTTGCTTTCTATCATGAACCATATTAATTATCCGTGCTCCTACTAAATCTAACATTAGTGGATTTTTACTTTTTAATGGCATTTTGAATCTTAAAACGGCTTCTCCATCTTTATATTCTCCCTTTAACATTTTTTCAAACTCAATTAAATTTTGTTCAACACTAGTATTTCTATAAGGACTTAATATTAATGGATCATCATTTCCTTCATCTCTTTCTTTTTGATATTTACTTCTTCTAGCTCGTATTTCTTCTAAACTACAAAAACAAACATATGATTTATTTTTTTTTATTAAAGTTTTTGCCATTTCATGCATTGCTTCAAAATGGTCAGATGATGCTGTAATAGCATATGGTTCAAATCCTAACCATTTTACATCTTCAATTATACCATTATATAATTCATTACTTTCATTTTTAGGATTTGTATCATCAAACCGCAAATATGTTATTCCATTAAATTTTTTTGCATATTCAAAAGATAAATTAAGGGCTTTTGCATGACCAATATGTAAATTTCCATTAGGCTCGGGTGGAAAACGAGTGATTACTTTACCCCCAGTTCTTTTTAAATGATCTTCCATAATTTTTTTTGTTTTTTGTGGATTTTCTCCAGGTTTATGCAAAAATTTTATTTCACCTTCATCTAACCAGTTTTTTGTATATTTTTCCATAGGAACATTTAGATTATAATTTTCCATTTCTTTTAAAAAAATTTTTGTATCATAATATAAATATTGCTCTTTTAATTTTGAACTAATTGTTGCTTTGGTCTCTTTCTTTTCTTTTAATAATCGAATAAAATTTATAATATCTTGTTCATTATAGGTATGTTTTTTACAAAAATCAATATATTCATCTTTGCTTTTCTTACCTAATTTAACTAGTCCTTTAAGTAAAGAATCATTAGTTACATATCCTTCTTCAATAAGATTAGATAATTCAATTAAATTACAATTTTTAGGTGCAATACATGCTAGCGAATATTGCATTTTAGTAAAATTATTCCCAGTATTAACTATCACCTCCATATTTTTTATTAAATTTGGCTTAGTTTTTAATTCATTAATTTTTTCATCACTTAATGAAATACCTCTTAATTTATCATCAAGATTCATATGTAGAAGGATAATAAAAATTTTTTATATAAAAACTCAATTATTCAATAATTGTTAAAGTTTATAAATAAAATGGTTCTATGGTGTAATGGTTAGCATATAGGTCTCTGAATCCTGTGATCTGAGTTCAATTCTCGGTAGAACCATTAATAATGCTTTTTTATTTACATAATTAAATATTAAACAAATGCCCTCATTGTAATGGGAAGCACAAAAGAAGTATTTGATCCATATGAATATATCAACAAAACTTATTCTGAAAGTAGCTTATATGAATTAACCGAAGCTCTTACATTTATGTCGAATATCAATGCAAAGACAAAGCATGAAAATAAAAAACTTATTTCGTCACATTTTTCAAAATTTATTGAATGTAGAACTACCTTAGAAAATATATATGAAGATGTCAAAAATAAAAATTTAAAACAGAATTTAACTGATCGTTTAAAAAAAGTAATTACTATGTTATTAAATAAATATAATGCAATTTTTATTAATGTAAAAGATGATCTCGAACAAGAAATGTTTAATAATAAACGAATTTATTATGAAAATGAATTTGCTGAAATTCTTACTTTAAAAGCTAACTTATCTAAAAATGTAAATAATTTTGAAAATTTTGTAAATTTATATAAAAGAGCTAAAAAACTTTATGAACCTTATAAAAAATCAAAGTTTTTATCAATGAAAATTAATGAAATTCAACCTGAAATTAATACTTTCTTAGAAAATATATATTCATACATTTGTAATGAACATCTTAATTTTGATGAATCATGCTATTATTTTGATTTATATTTTGAAATTGCTAATAATAAAACAGATAGAAAAATAATGAATACATTATTAGTTACATTTAAAGAATGTACATATGCTTTTAATGATGTTAACAATTACGAAGAGTATATAACTTACTTGGAAGAAAGCCTATTGAAATTTATAAACTATGTAGATGATGATATTAAAAAAGAAGGAATTAATCATTATTTTAAATGTTTAACAATAGTAATTACTGATGTTAAATTGGCAAAGATTGCTTTTAAACAAGTTGAAAAAATTATTAATAATTTTGATATAACTCCACAAATAAAACAATATTTTATTCAACGTAGTACAGAGGCTAAATCAGATATATTTTTTAATTTATTAAAAAATAATGAAAATTTTAAAATTGTAGATAACAATACCAAATACGATTATTTTATAACAAATAATACATCAATTTCAGCTCAATTAAACAAATATTTTGATCAATCAGTTATTATTTATACTGAATATCAAACTATATTGAAGGAGAATGAAGTATTACATATTCAAGATATATTATTAGACCATATTAAAATATCGATTGAACAACAAAATATTAATAAATATGAATTTTTTAAATTACATGTTACAAGTATAAGAAAATGTTTAGGAAGTAGAAAATCAATTAAAAATAAACAACTGGATAAATTTTTAAAAGAAAAACAAGAAAAAGCAATATTGGAACTATCTAATGATTTAGAAAAATTAATCGATACTACTATAATAAAAAATCCCAAAGCAGATAATATTGTAGATAATATCATCCATGTTTTTATTCAAATACTTCAATTATTAAATAAAACTTCAGTACCAATCTTATGTGAAGTATTATTTGAAACCAGAGGAATAATTATTAAAAACAAAATATTATGGTTTTTTTTATATAAATTTATTAAAATGAAAGATCCAGAATTAGATGATAATGATAAAATAATAACCAAAAATATTTTTAAACAATTTAGTTTTTTTCGATCGGAAATTATTAAATAAATTGTTTTAATTTAATGATTTAACCCTAATGTATTTACGAGAGCAAACCAAGACAGGAATTTTTACCTTTTCAATAATTGTTTTAATTCTAGTTAGTGGTCGAATAACAATTTTTATGAATAATTCTATAGGAATAGTAAATCAGTTGTATGATCCACTATCATTAACTATTATTGTTACTTGCTTATTTATTAATTTTGGTTTAGGAGTTCTTGGATTATTTATTATTATGGGATTTAATAATTTTTTAACACGTAATTTACCTAATACCATTGGATGCCAAGAAAAAGCAAAAATATTAGCTATTTCTGTTGTAATTAATGGTATTTTAATGGCATGTATAAATATAAAAGTTTTATTTAAGACAAAAATTGTGGAAATTGATAATAAATTAATATATTGGTTGTTAGGACTTTTTATATCTCAAATTTTATGTTTTTATTCTTGTTGGTTGGTACATTCGGATTTTTTTAATTTAGAATTTAAACAATTTAAAACTAAAGTTATATCAAATAGAACTCAACATCAAAATCTATTTGTTTAATACTGTGTAATTAAATATTTTTGTTATAGCGTTTTTATTCCTTATAATATTAATGGAAAAGAATATATTTAATGAATTAAAAAAAAATTTAAAAATGCAAAATAAACCGGAATTTACATTTAACATATATAATGAATTTGAATATGATCAAACATTATTAACAAAATGTAACAATTATTTTATACAAAATGGTGTATTAAATACTAATAGTATTCCATTTATATTTGATATTTTTGGAATATCACAAGATATTAATTTATCTGAAGTAGAAAAAGCATTGATGAAATATAAAGAAATCGATCAGCCACTTTTTGAAAATCAACAATTACATACTGATTTTACAGGATTTAAATCAGCCAGTACTATAAATCAATCTACTATGAATAAAAAAATGCATGAATCAAATACAAAAACCACAGAAAATGATTTATTTAAAGAAAAAATTAAATCTGATATTATTAAATCACATATGTCTATAAGCTGGAATGACATTATAGGACTAAATAAAGTTAAACAAGCCATTAAAGAAATTATAATTTGGCCTATGTTACGGCCAGATATTTTTGTTGGATTAAGAAATCCACCTAAAGGATTATTATTATTTGGACCGCCAGGTACTGGAAAAACAATGATTGGAAAATGTATAGCTGCTCAGGTAAATGCTACGTTTTTTAGTATATCTGCTAGTAGTTTAACATCCAAATGGGTTGGAGAAGGAGAAAAATTAGTAAAAGCATTATTTGAAGTTGCAAGAGAAATGTCTCCTTCAATAATTTTTGTTGATGAAATTGACTCATTATTATCACAACGTCAAGATAATGAAAATGATGGAAGTAGAAAAATTAAAACAGAATTTTTAGTTCAATTTGATGGTGCAAAAGTTGATGATTCTCAACAAATTTTATTAATAGGTGCAACTAATCGGCCTCATGAAATTGATGAAGCAGCCAGGCGACGTTTAGTTAAACGTATTTATGTTCCACTACCAACAGAAGACGAAAGATTGGAAATGATTAAACAACTTATTAGTAAATATAAAAATAACATTTTTGACGATCCAACTAATAATGATAAATTAGTTCAATTAACTGAAGGATATTCAGGATCAGATATTTTTAATTTATGTAGAGAAGCAACTTTTGAACCATTAAGAGAAGTTATAGATATTCAAACTTTTCAACTTGAACAATCAAGAGCAATTACCATAGATGATTTTATAAAAGCTACAACACAAATAAGAAAAAGTGTTTCAAATAATGACTTAATTATTTATGAAAACTTTAACAAGGAATTTGGATCAGTACATTATTAATTATAATTTATTATTTACTCTTGAGTTTGAATTCTCATAAATATGTTCATTCCTTGTAATTCTTGTATTAATAATTTAAAGGCATATGGAATATTAATATTACTTATGGTTGAATTATTACATCCAGTGCAATTGTTATTGCAAATTAATCCGCAATTATCACATACTCCACAAGTATAAACATCTGATACATCCATTAATCTTTCTTTTAAAAATGCACTGGCGCCATGAGATATAATACAATCTCTTTCCATCTCTCCAAATCTTAGCCCACCCTCTCTTGAACGACCCTCAACAGGTTGTCTAGTCATTATTTGAAGAGGACCTCTAGCCCTTGCATGTAATTTATCATCAACCATATGTTTTAATCGTTGATAGTATGTTGGCCCAATAAATATTTGAGATTTTAATTTATATCCAGTTGCTCCAGAATGCATTATTTCAAAACCTCTTTTTTGATAGCCATATTTTGCTAATTCTTTGGAAATTTCATCAATTGTTTGAGGTTTATTTGCAAATGGAGTAGCGTCTCCTTCTATTCCATTTAATGTAGTTAATTTACCCAATAATGCTTCAAATAAATGTCCAATAGTCATTCTACTTGGTATAGCATGTGGGTTAATAATGACATCAGGAATTAAACCATCAGATGTAAATGGCATATCTTCTTGTTTTAATGTTATTCCTACAACACCTTTTTGAGCGTGACGACTAGCAAATTTATCACCCATCTGTGGTATACGCATACTTCTCACTTTAACTTTTGTAAATTTATATCCATCTTTAGTTGTTACAATAACTTTATCTATGTTTCCTTGTTCAGTTTTTCTTATTAATGTACTGGTATCTTTAAAAAGATAAAATGGATTTCCAGGAGTTGATTTTTCTACATCTAATATTGGAGTCATTTTTCCTATAATAACATCATCTCCATAAACTTGTGTCCCTGGACATACTATTCCATCATTTCCTAATATATCATAATTATTTTTTTTGGGACGAAAAATTACACTTCTTTCAATTTTACAGATTTTTTCATTAACTCCAGTATATTTCATTTTTTCTTGATCAGAATATGTTCTAAATAAAAATGATCTGAATAAACCTCTATCTATTGAAGCTTGATTCATAATAATAGAATCTTCTTGATTATATCCTGAATAGCATGCAATAGCAACAATGCAATTTTGACCAGATGGTAATTCTTTAAACTTTATATATTCCATTGACTTTGTACTAACTAATGGTTTTTGTGGATAACATAATATATTGCTTAATGTATCCATTCGTTTAAAAAAATTTGTAGAATAAATACCCATAGCTTGTTTTCCCATGGCTGACTGATATGTATTTCTTGGAGATGGGTTATGAGTAGGAAAAGGAATAACAGATGCAGTTATTCCTAATATAATGGCAGGATGGATTTCACAATGTGTATATTGTTTGTTAGTGTCTTTTAAATCGTTTAATCCCATAGCTATTAAAGAAACTTCTTCTTCTTCAATATCTAACAATTCTATTTTACCATTTTTTATTAAATCATCCCATTTAATTTTGATATTTTTTAAATCTTCAATATCTTGTTTATTAACAAGTAATTCATTATTTTCGACAATAAATAATGGTCTACATGGTCTTCCCGCATCAGAATTTATCTTGATTTCATTTTCTTTAATGTCAAATATTATTGATATTTCTGAATCAATTTCTCCATGTCTTCTAAATTTTTTAAGAATTTGAACTATTCGAACAGGATCTGAAGTTACTCCAATCCATGCCCCATTAACTAATATTTTAGTTCCGTATATGTATTCAATTTCCTCTAATCTTTCTACTCCAAGTTCTTCGAGGATTTCAATTATTTGATTGGATGATTTTCCAACAGAAATATAAGCCATTAAACTTAAATTTTTTACTAATCCACACGCATGCCCTTCAGGAGTTTCAATTGGGCATATCATTCCCCAATGGGTATTATGTAACTGTCGAGGAGCTGCTAATTTTCCTTCTTTTTCAATAGGAGTGTTAACTCTTCTTAAATGAGATAACGTACTTAAAAAATTATATCTACTTAACACTTGAGCTACACCAGCCCTTGTTGACATTGCCTTTTCTTGGTGGCCCCAATTTCCAGTAGCTAAAGCATATTTAAATCCCTGTGTTATTATACTAGATTTTAAGCCTATAGTTAAATTAAATTCTCGATTATTTTCAACACATTTTTGTAAATATTTTGTAGTTTCTGTAAGTAATTTTTTAAATAATACTTTAAATAAATTTGCAAGTAATGGCCCAGAAAGATCCATTCTTTTTTTTCCATAATGATCTCTATCGTCCACATCTCGTTTGCCAGTAATTACTAGTAATATTTTATTAATCATATATCCTAAAAAATATGCTTTTTTTGTTTCACAAAATTCTTGCTGTCCAATATGTGGTAAAAATTCTTTAATAAGAATTTGTTTAGCAAAATCAATTCGTTTTGATTTAGGTGATCCAATGGGTGCAGAACGTTTTCCTATAAAATCAAGTGCAGATAATTGATCTTGAATACAACTAGCTTCCTCTAAACTATTTAAAAGATATTCATTTAATTCTTTATCATTATCATATTGAATATATGAAAGAATTTCTTTATCAGAATTAAACCCTAATGCGCGAAATATTATTAAAACTGGAATATCTTGTTTTACTAAGTTAATACTTGCTCGTATTACACGAAGGTTCTTTACTACTTTTAAAGAAAATATACTAGGTAATTTGGATCCTATTTCTGCAACAGATCTAATCTCTGCAAAATATGTATATGGTGCGGGCTGACTTTTTTTAAAAATATGAACCATATTTGTAGCTATTCTTTCTTGTGCTACAATAACTTTCTCTCCTCCTGTAATAATAAAATATCCACCTTGATCATAAGGGCATTCCCCTAACCGAATATTGTCTTTATCATTTAATTCTTTAAGAGCACAAAGTTCACTCTTTACCATTACAGGTAGTGATCCAAACGGAACTCGAATTGTTTTACTTACATCAACTTCTATTCCATTTTCATAATATGCTTTTTTTACATCAATAAACATATTTACCCAGTAAGTTAAATCCCTTAATCTTGCTTCATTAGGCAATATCTTAGATGATGTTCCATCGATTTCAGTATTAATTGGAGGTCCTAAAATGGATATTTGTCCAAAGTATAATTTTATTTCTCTTTCTATAGAATTTCCAGCTGTTGGTATACTTTTTACAGATATTATTGAATTTTCATCAATAATTTCTTGCATTTTTATCTTTACAAATTGATTAAAACTATCCAATTGTTGTCTTACCAAGCCTTTCTGATCAAAGAATGAAGTTATAATTGCCCAATTGTTTTCTTCATTGCCAAGTATGTAATCTTTATTATTATCTGCAGAGAAAGTAATGTCCATAGGGAGTAAAATTTTTTTGTTTAAATATTAGTATTTAATTTTGATCCTACCAATGATATATAATTTAGGATCATCTTTTATGGAATATTATAACATAAACAATTTTTTATCAGACGAACAATATGTATCAATAATGTTCAACGAAAAAATATATCCAACTAAGTTATATAAAGGGATAAATCCATTTAAAAAGATAGAATTACCTATCTTTTTGATTAAATTTTTATTAGAAAATGAATTTTGCGAAATATGTGCAAAATGTGATAATAAATTAATTGATAATCTTTTAATATTTAATATAAAAAATGATTTATATGCACAACCAGAATTAGTCAATTTTCAAAATCAATATTTTTATACATTTATAAATGCATCTGAACAAATTTCATTTGATACAAAGTTAAATTCGTTAAATCAAAACACTTTTTTTATAAATACTATATTTTTATATAGAATGGGTTATTTTTCCAAATTGCTAGTTAAAGATAATTTTCACGAAAAAGATTTAGAATATTTGAGTTTTGAAGAATACAATATTATCTTAAATGCAAGATACATGTATCAAAGATATCAAAAATACGAAATAAATTTAATATCGTAAAATTTATTTTCTCCATAAGAAAAAATAAAATATTGCTAAGCATATTATAAAAATTATAATATAATTTTTATATTTATCAAATTTTTCCCGATTTTTAATTTCGTTTATTTTTTTAGAAATAAATTCAGATTCAAATTTTAAATTTTCAGCCATATGTTTAAGATTATTAATATTTTCTCCACGATAAATTAAATTATCTAAATTTTCAACAAGATTATTTTGTGTTGTTTGTAAAATTTCATTTGTATTAATTATATGTTCTGATTTATTAAATTCGTCTGCAATTTGTTTAATTTTTTCATCATTTTGATAACAAGCAATATTAGGATTTTGTATAATAATATTATTTTCATGAATACTTTTTGTTAATTGGTCTAAATAAATCAAAATTGTTTTATTAGTACTTTTAGTATCTGTGATACAAGTTATACATAATTGTTGATTAAATTCATTGGATATATGAATATAAAAAACAAATCTATTATTTTCAGATACTTTAGAATATATTTTATTTGTTTGAAAAATATTAATATTATCTTCAGATAATACCTTAGTAACAATATGTTTTAATTCCGTTAATATAGCATAATCTTCTTGTAAAAATATTCCTTGACTTGAATGACTTCCTGTTAATATTTTTTTAGTAGTAGCATTTAAAAATTGAGTGTACAAAACCGCCATATATATTCTAAGGGTAGTTCTATTTAAATAATTATCACAAAATAAAAATGGATAAAATGCAATTTTATTAACAAATCATTATGAATTTATAGTTTAAATAAAGAAATCAATTTGTATACATCATTTTTATTCATTTCTAGCATAAATTCTTTTTGGAATTTTAATCTAATATTATTTTCATTATCTATAATTATATTTTTTTTATTGAATATTATATGATTTATTTCTTTTCTATTTAAAAATATTCCTTTAATTTCATTGTCTTCAGTATTATAAATATATATAATTCCATTTCCAACAATATTATTTGAAGCTGAATAAGTTATTAATTTAACAATTACAGTATTTGTATAATTAATTATTTTTAAAAAATTTTGATCTATTTTTTTAATAAAATTGTTAATAATATATTCTTGTCGTATATTCATTATTTATAGGGATAAAATTTCTATTTTTTAATTATATTATATTATTATCATGATAAATATGATTTTTTAAATAGGATCCTCCCTTTAATTTTCAATTATCATATTTTTTATTCCCCAAATCAAATGAATATATTGTGTAATTACTATTTTGTATTTTTTTTGCAAGTATTAACATCAGAATATACTAAAGTATTAGTTTTTACATTTAATGTTGGAAATATTTCTACATTTGATATTCAAAAATTTTTAAAAAAATATGATAAACGTTGTAATATTGATTATCAAAGATTCGATATAATTTTGTTTAATTTACAAGAATTTCGTCAGTTAAATTATAATTTAGATGGATGGAAAGCTAAAAAATCACAAATGGGTCAATTAGCAACTATTGTATATTCAAAAAATAATGTAGAATTTCAGTATGAATGTATTGGACATGGAGTGTTAGGATTTATTAATAAAGGATTTGTTATCTCTAAAATAGGAAATTTAGTTAATGTTAATATGCATTTACCTGCTCATAGTTGGAAAGAAAAAGAACGAATAAAGTCACTATCTTATCTAAGAACTATTCTTCGACCATTTAATATTACTACTCATAGAATTATTTTATCTGGAGATATGAATTTTAGAATAATAAATGGTGAAGATGAAGGAAAATATACACGAAGTGTATTAAATAAAGATGTAACATCCTTTTTTTACAATGAATACAATGTTTGCTTTCCAAAAACATATAAATATATTAGAGGAACTTCAAATTATATATCTAATCCTAGCAAACGTTGTTGGACAGATCGAATTTGGTATATTTCTAATGACAATATAACATCATTATCATTAAGTAATATTGACAAATTAGAATCAAACGTTAATCTTTTTAATAGAGTAAAACCATGTATTTCTGATATATTTATTTATACCTATAATTCGATCGTATTTGAAGAAATATCTGATCATAAACCGGTTTATGCGAAAATTATAATTAAAGATATAAATTTACAAGAATCAAGTCCTATTACACTTAATATTGGATCGTATAAATTAAAATTGACTAAAATTTATATATTTATCTGGACTTATTCCATTTTATTAGTTGTTACTAGTTTATTTATAATACTTTGCACATTATGTACATTTATAATTAAAAAATATCCACTTAAATTTAAAAAATGGCTTCATAATAACTTGGAATTACATTATTATAAAAAATTATTTTATAAATAAACCTATTTTATTATATTTCCATAGAGTTTTTTCAATATATCATTATTAGTACTATATTTATTTAATAAAATTAAAAAATTATTAAAATGAATTGAATTTTGACATACATTGTGAGGATTTATTACAATTTGAATAAATATATCTGCAAATTTATAATTTTTTAATAATAAAAATATTCCTAACGATAATCCTAATGATGTTTTAAATAAATTACTATAAAAATTGGCATCATAACAACTAATATCTATTTTAAAATCAGTATTTGCTTGAGAAATTAAAAAATCAAAAAATAATTTATGTTCTTTTTCATTAAAATTAATTTGAGATATAAATTGTATTAAAAAATAATTAAAAATTATATCTTTTTGTTTATATGTATTACACATGGTAGTTAAAAGATTAATAAAGCTATTCATAGTTCTAGTTGTATCTATACATTTATAGTTTTTTAAAATAATATCAAACAAGTCAAACTCAAGTTCTTTTCGAGTAATATTATCAATTTCAACATTATTAAGAATGGTTAATGTATCATAAAATTGTGAAAAGATCAATTTAATATTTAATACTTTACTATTGATGCTATTAGCCGATTCTCTCTGTAATTTTTTACTTCTTTTATTTTGTTTAGCAAAAATTTGTTTTATTTCTTTATTTTCATCCATTTCTTTTAACACAATTTTTTTTCCATTTATTAAATTATGATAATTAATATTACAACAATTAATTGTTTTTATTAATACATTAGATACATATATTTTTTCTTTATTGGACATAATACTTTGCGTAGTATTGACTTCTTTAATAATATTAAATTTATTATTCATTATTAGGGATAAAAACTTTATATTTGACATAATATTCAATTACCTATTATATAATAAATTTATTATAATAATAGTAAAAATTTGACCAATGGATAACAAAAATTTTTGTGAAAATAAAATTAAATTAAATGATTTGCTTGATGAAACAAATATAAATATTGATACTGATGAGCATAAAGCATATGCAGTATTTAAAGATGATCTTGCTGAATTTACAGTTTTAAATAATTGTACATCACAAGTAAAAAATCAATTTTTCTCAACATGTCCTCAGACAGAACTTACTACAAAATTTAATAAAATATTACATACTACATCACAAAATAATGATAAATTATTAACTAAAAAATTAACAATGGAATTTGATAAAAAAATGGCACGTATTAATAAAATTAAGAGTAAACATTATAAAAAAATGCTAAGGAAAGATCGTAAAAAAGATTCTAAATTATATGAAGAATTAAGTATAAGTGTAGATGAAAATTCATCATCTACAACGCAAAATAATTTACATGTTAATTCTATGTTAAATATTAAATATCAAGATAATTCAACTAGTTGTATTAAAGATACAACTAATAATGTATTAAGAAAAAATGTAAATATTGTTGAAGATATTTTTGAAGAAAATGATAGTTCAGAAAGTAATGATTCTTTTATTCAAAAAAAAATTATGGTAATGAAAGACGAAGCACCAGCTATTGAAGAAATTATATTACCTGGATTTGATGGTGAATGGGCAGGGAGTACAATTGATACTAAACAAAACATTTGATAATACCGTTAAATATATTAAAAATGGTGTTCCAGTATATAATAGAGTAGATTTTACAAAAAGTAATGTAATAATAAGTGAAAATATACAATATAATGATAAATATAATGCAAAATTATCTTCTAAATATAATAAAGATTTATATAAGAAAAAATTAAAAATTAATATTACTACACGAAAAAATCATATAAGTGAATCTGAAGAAGAAATAGTATGTAAACAATTTTTAAATGAATAAATTTTATTTATATAATTCTCCTTTATATGAAATCATTAAAAAAAATTAAAATTTCAAAAGTACTGAAAGGTGTATTTCCCAAATATAAAAAAAAAACTCCAAATAATACTATAGATCAAACTATAATGTCAAATGAATCTTTTCAATATAATGATCAAGAGTTATCTATTTTTATTAATCGTGTAAATGTACTATTTTCAATATCATATTCAAAAATACTTAAAAATGATACTAATTTAAAAATATTATATGAAATGAAATCTTTAACAATTAATAAATCAGATAGAGAATTATTAGAAATATTAAATCAATCAATTTTTGACAAATATTTTAATTATATATCTTCAAATTCAACTAATATGGATGAAACTAGTTTAGATATTATACCAAATTCTTCAATTAAGCCTACTATACCTATAACTCAACTTAATCTAAATGACATAGAAACAGAACCCATTTTAAAAAATGATACTACATGTCAATTATTAAAAAAAATAAATAATAATATAGTAAAAGATGATAATGTATTTTCAACAAAATCTTCTAATTCAAGCCTTTCAATAGATAATGCTATTAATAATTGTAATATTCCAACTATAAATCTATCTAATACACCAGTTATTCATCAAAATAGCGGACAATATATTATTGAATTTAATGTTACACAGTTAACTAAAAAAACAAATATGTTTTTATGGATATGTAATATTTTATTAAGCTGGATTTATTTTATATGGGATAAATTATTTAAATGTTGTTGTATACAATCAAAAAAACAATACTTATTTTTATGTGTTAATTCAATTTGTAAAGAAAATATTATTAATAGTTTAAAATATATTTATCAAAATATAACAGTAACGAAAATACAAGTAAACTCTAAAAATATATTTACAATGGATGATATTTTACGATTTTATACTGTAAAAAACTTAAATATAAATGATTATACTTTAACTCAATTATTTTTTATAACCGTATTTATTTTAAAAACGGATCTAAATGGATTATTTGATACTAATAGAATAATTAATTTGTATAATTATTATATTAAATTTACAACTATTGAGTCTACTATTATAGAAAATGAAATTAATTTATTAGATAATTTTAAAAAAGAAATTTTTATATTATGTAAAAACATTGTTTTAACTATAAAAAATAATAGTCTATATAATATTCAATCTAGTGAAATTTTAATTAATGAAATATGTTTTAATGTATTTTTTTCAAATTTAATAAAAGAAAACACTATATCTTCAAATATTTTACAACTTAATAATTTTGAACTTATTTATAAGGGAAATATATCCAGATCAAATTTAAAACAGCTTTTTACTGTATTATTAATTTAAATTAATTATTTTAGTTTATTCATAATATTATTTGATAATTGTTGAAATTGCGAATCATTTTCATCTAAAAGCAATAACTCTAATGCACTAATTTCAATATCAAAATTAAAATTATTTGATTGTTCAATAAATTTTAATTTAGTTATACACCACTGTATAATATGTTTTGTTATATATTGTTTTTTAAGCAAATACTTTTGATAGATATTGACTAAATATTTTAATAATAAATTTTTAGTAAAAATATATTTTTTAGATATTGGTAAATTGGTTTTCATTAATTTATATTCAGTTATTTTTGTTCCACAAAAATTATTTTGAAAATGATCTAAAGTTGATAATTGATAATCAGGAATTGTATTATAATTAAAAATATCACTTTGCTTTTTAGTTAATTCTGAACAGGATTGATTAGTTAAATTTAAAATCTGTTTATTAAAATATGAATTTTTAAATATAGAATATTGATCATTTAAATTATACAATATATTGAGATATTGTTCCTCATTATTAATAAACTTAAATGCTGAGTTAAATTCATATTCCATTTTACATTCATTATTAAAAAATGTATAAGAAAATATATTTACTATTTTGTTATCAGGTTTATATGAAAATAATCCTGAATAACCTGTACTAGATAACATATTTAAAGCTTCAATAGCACTTAAATTCACTCCATATTCATCAACTGTATCATATTTACCAAGTAATGTATATGCATCATTATATTTGCCATAAAAATACAAACATTGTGCTGTTAAAATATCAACTTTTTTTCCTTGTAGTTTTTTTAATATTCTAAAAGCTCGTTTAAATTTTTTTAGTTTATAATAACAATATGCTTTTTCAAAAGTATTATTATGCATATACGTTACTGCCTTTTCAAATTCACCTAAATATATGTGTGCAATAGCTTTATATTTATCATATTTAGGAGATTCTAGCATCAAAATATCTTGATGTCTTTCTTGCTGAATCAAATCATGTATAAACATAAGGGATAAAAATAATAATAAAATTATACAAAATTACTTATTTATTAAAATATCCTATAAAGAGTTAAGAAAATTATTAATCCATGTTTTTTCAATTGTTCCTTGAAGAGTATTATTTATAATTCTGACTTTATTATTTTCTGCAATAATTTTATTATATATTTCATTTCCATTATTTAAAAACAAATAAATTAAATATTCTGTTTCATTTATATCCATATATATATAAGAATTAAGTTGTAATTCAACAAAAATATTTCCAATTGATACAATTATTTTAGAACAAACTTTTTTATTTGCTTTAAAATAATCTATTTTTTTTTTAATAATATTTACTTTATTATTAGTTAAAGTTAATTTTGCTAAAGTATTATTTAAAATTTCATTTTTAATATTACATATTAATTGATCAATATGTTTTTGAGTTTCTTTATTTTGTTGTTTAATTAATTCTTTATATTGTATCTCAAATTTTCTTTTATCAACAATAGGTAATAATATATTTAATGGTAATATCTCATTGTTTTCAATTGCTTTTTTAATTTTTATTACATTTTGGTCACATTCTTCGGCCAATTTACCAGATACTAATGTTATTCTTCTTACACCCATACTAACACCGTTTTCATTAATAATTCTAATTTTTTTAATATCTTGTAAATTTTCTGCATGTGTTCCTCCACAAAGTTCTTTTAATACTAAATCTTTAGATTTCATAACAATTACTCTCATAGGCTCTGGATATTCAACATTTTTCATTAAAATTATATTTGGATCATCTAATACTTCTTTTTTAGATAAATATTGAACTGTTATATTAATTCCGGAACAAATAAAATCATTTGCCTTTTTTTCAAGTTCAATAAGCTGTGAATCAGATATTTTACTTGGATAATTAAAATCATAAGTACATTTAAATTCATCCACATAACTACCTTTTTGTTCAACGTTTGTACTAATAAATTGTTTTATTAATCCACCTATAATATGACAAGATGAATGATTATTTCTTATATTGATTCTTCTATCATTATTAATTTTTAATAAACCATTTATAGAAATCTTACCAAATAATTTTCCATAATGTAAAACATATCCCCTAACAATTTTTGTATTAATTACATTAAACCAACCCACTTGAACATCATTATCAAAAAATGTTATAGTTCCTGTATCTCCTACTTGACCTCCACATTCTCCATAAAAACAGGTTTTATTAAAAATTAGCTGTGCTTCTTGTTCATTATCTAAAGAATGAACAATTTCATTATTAAATATTATAGCTTGCAATTCTCCCTTAATCTCAGTATGAGTATATTTAAATTTATCATCTGTTTTGTCAAATTCAAATTTAATTGATATAATATCTTTTTCTCCCTTACTTTTTTTCTTTTGGTTATTGAGTAAATTCTCAAATTTATCTAAATTAATTTCGATTCCTTTTTCTTCAGCTATAATTTTTGTTAAATCTTTAGGAAATCCATAAGTATCATATAATAAAAATGCATTTTCGCTGGATATTTTCCCTTCCGCTTCAATAATTTTATTTAATTTTATTAATCCATTTTTTAATGTCTTCTTGAATAATTTTTCTTCATAATCAATAACTGACACGTTTATTGAAGATAAATTTAAAACTTGAGCTGCTTTTTGTACTATTTCAGATAAAACATTGTCATTTATATTTAATATTTCACTAGCAAATCTAACAGCACGCCGTAATATTCGTCTTAAAACATAACCTCTACCTTCATTTGAAAATGATACATTATAGTATAAACATATAGCCAAAGTTCTTGCATGATCAGCAACTACTCTAAATGCTGTGGTTATAATAGTATTTTCTTCATCATTATACGTCATATTACACTTTATCTCAATAAAATTAATAATGTTTAAAAATGTATCAATTAAATAATTACTTTTAACATTATTTAATATAGATAATAATCTTTCTAAACCTATTCCTGTGTCAATTTTTTTAACTTCTAAAGGAAGTAATCCCTTAGGAGTTTTGTTAAATTCCATAAATACAATATTCCATATTTCAATAACATTTGGATCATCTTTATTAACTAAATTTCTAGCATCTCGATTTCCTATCCTATCATAATGAATTTCTGTACATGGACCACAAGGTCCAAATTCACCCATTTCCCAAAAATTATCTTTATGACTTCCATTTATTATTTTTTCATCAGGAAAATATTTTTTCCATATATTTCGACTTTCAACATCCATTTCCTCATATATAGTTACATATAATCTGTCTTTATCTAATTTCAAAACATCTACTAAAAATTCATAAGCATATTGAATTGCTTGTTCTTTAAAATAATCTCCAAAAGACCAATTTCCCATCATTTCAAAGAATGTGTGATGATAATTATCTTTTCCAACATCATCTAAATCATTATGCTTACCTCCAGCACGAATACAATGCTGAATAGTATTAACTCGTTTATACATAGGTGATTCTCCTAAAATAATATTTTTAAATTGTACCATTCCGGAATTAACAAACAATAATGTCGGATCATCAATTGGAATAACTGAACTACTTGGTAGTGTTTTATGATCATGCTGTTCAAAAAATGCGTGAAATTTCCGTCTTAATTGTGTACTAGTCCACTTCATTAATAAGGAACAAAAAATTTTATTATTAAATAAATTATAATATTAATACAATAATATTTAATAATAATGATAAAAATACTTTAATATGATAAATAACATTCAAAATTTATTGTAAATAGCATTATAAACTATCTTGATTTAATTCTGTTTGTACAGTATATAAAAATTGTATCATAGTATCTGAACTAGCTCCTAATTTAAATAAATTTAACATTATCTCAATAGATTGGTTATCAAATTTGTATCCTAATATATGTGCCATATCTTTACAAGTTGCAAGTTCAGTGTTTGAAAATTTAAAAAATTTGTTTTCATTCATACGGATAAAAATACTATAAATTTATTTTATATTTTTTTAAAATTTGTAAATCGACCAAATGTGCTATTTGATGAAAATGTTGGTGATTGTAATACTGTATCAACATTAGTTGAACTATTGACATTATTTATTGAATTTTGTGTATTTTGTTGAATAATATTATTAAATAAATCGGATGGATTTGATACTATATTCACAGAATTAGAATTGGAAAAATTAGTAAAAAAAGTTTTATGATTTTGCATAGGATTATTTGTATTGGTGGAAATTAATGGTATTACTGGATTACTATCAGTTTTCACTTCATACATATCATTCTTTATATTATTATTTATTATTACATTTGTATTATTATTATTATTATTATTATTATTATTATTATTATTATTATTATTATTATTATTATTATTATTATTAGTAGTAGTAGTAGTAGTAGTAGTAGCAGTAACAGCATCATTAGACATTATGGTTGTATCAATTGTAAAATCGTTATTTGAAATATTATTATGTTTAGTAGTAATATCACAATTATTGTGTGCTTCTTTTAATTCTGTTTTATTATTGTTTATAGAAGGATCAGTTATTATACCTTTTGATTGAATTGTTAATAATTTTATTCCATTAATAATATTTTGTACTTTTTCTTTAGATTTCTCTATATAAATATCTTTTCCTTGTAATGGATATTTATAAGTTACATTAATTAGTGGTTTATGTCCTTTATAAAATATTTTTTGATCAAAATATTTAATAGTTTCTTCTATGTTTTTAACATCAATGGATGCATATTCATCAAGTTTAGTATTCATTTGAGATAATACATTTAAGTATCTTGCTGAGTCTTGTTCAAATATTAATTTATATAATGTAGATAAATCAAAACTACTATCTATTAATTCAAAATAGTCAACTGTATAAGTTATTAATTGAGACTTATAATTATTTTTTAAATAAAGTATGTCATTCTCTAGTATATCATTGAGTAATTGTTTATTATTCATTTCATTATTTGACATTATTGATTTTTTTTCATTTGAAACATTATCTGATTTTTGAATAAAAGATGATTTAAATATATTTGATTTAAAATTAGTTAATGGTGAATTACTATTTTGAGAATCAAGTTTTAAAATATTATTTGATACTGGATGTGTTTCTTCTAATAATTTATGATTTAAATTAGTTTGATAATTTGATAATTGTTCATTTTTTAAATTATTATTTAGATACACTAAAGAAATTTCTTTGATATTCACAGTTCCTTTTGATAATATAGAATTATTAACCATAATAAAAAATGAACTAAATTTTTCATTATCTTTCAAATAAATCATATTATTATAAAACATAATAGTATTAATAAATATTGGTTCAAAATTATCATTAAATCGTAGTGATAATCCATTTTCTTCTTCCATATTATATAACTTTTGATTATTTAAATAAATAAAAGTATCTGATTTATTACCTGATAAAATTATTATTTCATTATTATTTATATTAAATTTAGTACCAAAATAATCAGTTCCATCAAGAATATTATCGCCTATTATATTATCATTAACAATAATTTTGTCAGTTAATTTAATATACCTATTATCTAATATTACCATATTATTTTCAATCATTCTAGGTTTATCATCTATAATACAAAATTCATTATTATGATACTTAAATGAACAATTATCGATTTCTAATACATTAATAGTACGTTTAAAATTTACTTTTTTATAATTTTCGTTTTTAAATTTAATTATCAATTCTGGATTAATCATAACATAGATCCATTTTTTAAATACAAATACATCTAAGGAATAATCGATGAATTCATCTTGTTCATTTTCTTCTATAAAATTTTTATGTGAAAATACATCTGATTCTTTAAATCCAAAATTAAACATAAGGGTAGAACAAATATAATATTCATTTAAATTTATATTTAATACTATTATTTATCAAAATGTTTAATAAAAATTATTATTATTGGCTATTAAAAATTATAAAACAAATTAAATTGAAGAAAATACATTATATGAATAATAAATGTAAAATACTTGGTAAAGAATATTTGGACCAAAAAGGTACTTGCCAATTATATAATACTAATGATAATTTATTTGCCAAAGTATCTTCAGAAAGTTTTGATGAAAAAAAAAAATTTATTGATGATATTCTTACAAATTATGAAAACGAATACAAAATTATCATTAGATGTATTAATTCTCAAATACCATATTGTGTAAGACAATATATATTAATAACAAAAGCTTTAGAAAAATATTTTAGTAATAAACAATTTGAAAAAAAACAATATCAGTCAACAACAAATATTGCTGTAATTAAATTAGAAAAAAAAGATTTTATTTTACATAAATATAATAATTTATCTAGCGAAAGTATATCTAAAATTAAAAATAATAATAAATAGTATTTATTAGCGCATTCGTTTCTGTTTAGTATATGGTATTAAAATAAATTCAAGGGGCTTATCACAAATAAAAAATCCAATTCTTTCAATTTGTATATATGTTTTTGGAGATACAGATATTAATGCTTTTTCTCCCAACCAAATTTCTTCTTTTTTAGAATTAGTATTAAATTTTTCAGCCAAATTATCTGTATTTTTATCATTTTGTAAAGTATCATATTCCAAAATTTTAAATTCAATAATATTTTCTTTATTAACCCATGTTAATTTATTTTTTGTTGTTTTAACGTCTCCAGCAAGATTTTCCTCTAATATTATTTTTATAATTATACCATTCACAATTTGTTTTTCTTTTACTTTCATATTTCCCCAATTCATCAAAGTAAACTCTTCGTTATTATTTAATATATTAGCATCTTCTTGAGATATTAAAATATGTGATGATTTAATTATAGTTTTATTTCCAAGAGATAAATTTTTTTTAAATTTTGGAATATCTTCCGTTTTAGTAATTATTTCATTATTGTTTTTATCTAAAATCAAACATTCTACGCAATATAATTTAGGAATTGCAGAATACCGTGCAGACTTATGATCAATATTTTTTTTATTTAATGACCATACTTTATCCCATGAAATAACACTAGATTTTTGTGATGAACCCTGATTAATTATATATTCTTTTAATGTATCAATATCCATTCCAAGTCGTAAAATACCTCTTAATGTGCTAAGTCTTGGATCATCCCATCCTGAAACAAAATGATTATCAACATAAAATTTCATTTGGCGTTTGCTTAAAACAGTATTTTCAAAGTTTAATCGACTAAAATCATAAATTTTTGGTTTATTGGGTAGATTTAATTTTTCTAAAAACCAATAATATTGCTCATTTCTATCTCTATACTCATTTGTTCTTAAAGCTAATGTAACTCCATCCAAAGAATCAATTATTGGACAAGCAAAATCATATGTTGGATAAATTTTATATTTATTTTTAGTAATATTATGTTCTTTTTCAATATGTCTATAAATCACAGGATCTCTTAATGCTTTGTTTAAATTAGTATAATCAATTTTAGCTCTTAAACAATAATCCAAACAATTACCACTACTCATTTTAGAAAAAATACTCAAATTCGTTTCAATATCAGTATTTCTGTTTTTAGATGGTATCCCTTTTGTTCTCTCTTCTCTCATTTGTAATTGATCTGTATTATCACAATATGCTAAACCTAATTGAATTAATTGTATTGCATAATTATATAATGAATCAAAATGATCTGAAGATCTTACAATAGTGTAATTTTTTATTCCAAGTGTATGTAAATCCTCTATAATAACATTTTCATACATTTTTTCTTCTTTTATAGGATTTGTATCATCAAATCTAATTAATAATTTCCCATTTTTTGCCATTAAATCATTCAATAATGCTGCTTTTATATGTCCTATGTGCAAAAATCCACTTGGTTCTGGTGGAAATCGAGTTACAACATTTCCTTCTACTGCTATATTTATTTTTCCTTTATCTTTTTTGTTATATTCTATTATATAGTTTTTATTTGTATTTAGAATATCATTATAAAATTGTTTTATTTCAACAAACGAAAATGTAGGATTCTTAATTAATTTTATAAATTTATTACATGAATATAAAAGTCCAAAAATTACATCAGCTTTTGAAGAATTCAATTTAGTATTTTTAAGCCAATAATCCAACTTACTTAAAAAATTATTTAATTCTTCATCAGTAAAATCAATACTAAAATCTAAAAAAAAATCATCCAATTTTTGTTCTCGTATATGTTGTTCTAAAATAGGCAATGTTTTAGGTTCTATATTATGTTTTTTTTCTAATAAAAAATTTACAAACTCTGATTTAGAAATATTTTCAAGCATAGGGTAAAAAATTGTTATATTAATAAATAGGTTCAACATAATAAACATCTTGTTTATAATAATTTAAGTTTAATCCTATATTTTTAATAAATTCTTCAGTATAAATAATAAAGTTATTGAAATAGGCATGATTTACTGGCCTAGTTGTAAAATAAAAATTGGTCTTTATTCCGATATAACTAAAAAAATTAATATAGTTTTCTCGATTTTCATTTCGTTCTTTTAAAATTGTATACACCATATTTAATGCATGTTTAATAACATCTAAATTTTCTACTTCTCTAAATGATTTATCTTCTTTAAATACTGTTATTAAATGCTTATCTAAAGTTTCATATATAGAATAATTTAAATTATCATAAATTATCGGATCATTAAGTATGAACTTAATAAATGGTTTTTTTATATGGTTTTCAAATGATTCTATAGTATATTTATATCCTTTATATTGAGTATCAGAATTTAACGTAGTTAATGCTTTTAAATTAATAATAGATTCAACTGGAACTAAAAACTTTTTAAAAATATCATTACTATGTGTTAATTCATGTTTTGATCGTAACATATTAGATATTTTTTCTATATTTAATTTTTTATATTCAATTTCTACTGATATTTTTTCATTATTATTATCTATTTTAACAATTCGTTTGTCATTATCTAATATACTCTCTATAACCTTCATAGGGGTAAAAACTTATATAATTTTTAGATTATAAAAATTAAATACCAATTTAAGTTCTGTATTTTAATTTAAATTATTTTTAAACAAAAATTAATAACCCTTATTTATTTATGGAAAATGAAAAATTAGAATATCCATTAACAGATAATTTAATTAAATTTATCAATGAACAAAGACCTATAGAAGGTGCTTTTATTGTTTCTATTATTGAATCGTTATTTCAAAAAAACAATTCTAACAAATTTTGTACAAAAATGATCTTATAAAAAAATTAATTCCTTTACTTGGTAATCAAGCTGAAGATTTCGTTAATAGGCTATTTAAAAACATTAGTCAGCCCATTAAACATGAAAAAAAAGTAACATTTAAAACAGAAAATGTTTTTAAAAAAATTAAATCGAATGATAATAAAAAAGTAGATGTAGATAATAAATTATCTTCCGATTTTATTGGATATGGAAGTTTTTTTACTTTTGAAACTTCAACTGCTGCAACTACAGAAAATAATACTGATAATAAATTAGCAAAAGAAGTAATTTTAAATAAAGTTAATGATAAAAAATATTCTCCAAATGAAATTAAACAATTTTGTGCGAAATTTGGAAAAATAGATGCTTTTAGAAAACTTAATAGAGAAAAATGGTTAGTTACATTTACTGATTATAAAAGTGCTGCTAATTTAGTAAAAACAACAGAATTTGTATGTGGAGATAGTGAAATAAAAAAGTATTTCAATGTATTTGCACCAGAATTTGTTAAAACAACACAAATAGATGGTTTTTTAAAAACAGACTTACATTCTTTATTATTACAGCAACAAGATCTTCTTGATAAAATTAGTCATACACAAAATTTCGATGATTTTATTGTATTGAAAAATATTACAGACACTATACGACAAATTATTTTTTCAGATGACAATTACAAAAATTATAATAAAAAACACGACAAAACATCCTATAAAACTATTTCAAAAAGTCATCTAAAGAAAATTTCCAAATTAAACAATAAACATAATAAAATAGAAGAAATAGATTCAATTTTTACACAGTATTTTACTTAATTACTAACTTTATCATTAATTCTGGCAAATCATTATTATAGATTTACCTAGAACAAGGATATTTTTAGTATTATTTACATTAAGTATACAATTAGTAGCTTCATCATAAGTAATTAATTTTCCATATATACTCTTCCCATTAGAAAGAAAACAAATTATTTCTGTATTTAAATATGATTCTAAATTATAAAACAACGTTTTTTTATTATTTCGTATTTTGGTTTCATTGATCTTTTCACTCATATAATATTCTTGAGGGAAAAATTAAAATTTTTTAAAGTAAAATTTCATTCAATCACTATTTTTCTTAGATTTATTATTTTTTATATTTTTATTAGGTAAGTCAATATTGCACAATAGACTCCAATTTTTTGAGAAAATTGAAATGTTTTGGTTAACTAACTTACAATCTCCTAAATAGTGATTATGTGATAAACACCGAGGACAAGAAAAGTTTTTTTTTATTTTTACATCACCTAATAGTTTATATCGCTTCCATATTTTTGGACAATCATCAATATCATGTAATATTTTACCATTACTTTTTTGTAAACATATTTCACAGGGAATTATTTTTTGTTTTTTTGTAGGACAATATTTTTCAAAATGACCTATATTTCTACAATTTGAACAATATTTAAATTGACAATTATCTGTTGTATGCTCCATATCACACCATTGACATCGCCGTATGCTATTAACTTTGCACTCACGCATTACATGTCCAATATTTCCACATAAATAACAAATTTCTTCATTTATAAATCTATTTTTTTCAACAGTTACTAATTTTAAATTACTATCCTTTTCAACTGGTATGAGAGATTCAAAAAATGCAAATTCTAAAATGTCATTTATAGATATTTTGTCATCCAATGTAATATAATAGCAATCATCTTGATATTTTGCACATTGAAATGCATTTTTTAATGAACAATCTAAATTTTTATCCATAAATTTACAAAAAAGGGTTAAATGCACTTATTATAATTTTATAAATATTGTTTTTGTATTTATTTTTTTTAATATCATAAAAAAGCGCTCATAGTGTAGTGGTTATCACATTCGCTTTACACGCGAAAGGGCCCCAGTTCGATCCTGGGTGAACGCAACGATAAATTTTTTCTCTAAATAATAATTTGTTAATTATTCTTTTTCTTTTGACACCATAAACAATTACAATTCTTAGGATTAATGATATTTACATTACAATAATCAATAGTTATTTCCTCATCAGGATATATTGTTCGTATTGAATAATATTTTATGGTATTAGTATCTAAACACTCATGAGATAAACAATTTGGTTTACATGAATGATTAACATACCTAGCTTGGTTTCCTACTAGTGTTGCGTCTATAATTTTATCTTTTCCTAAAGCAAACATATAAATTGAATTAATTCCATTTTTAATATAATTTTTTTCACGTTTATCACTCATTATTTTACCAATAATTTCACCTTCATAAGAAAATAAAAATACATTTTCAGGTATCATAATTTTGGAAAAAACACCTTTTCCATGAATTAAACTATTTCGAATTTCAAATGCGTTTTTTACCTTTTTTTCTAATGAAACCAAGTTCATTTTTACATTATTATGCTTAATTTGATCTTGATTATTAGGTGATATTACATTAAGATTATTTTGAATAATTTTCACAATATCACAATATATTTCTTTATTAAAAGTTTCAAACATATTATATAATTTAATACAATTGTTCTTTATAAGTTTAGATAAAACTTCATAATTTTTCTGATCAATATTTTGAATAGTATTCCTAATAAAAATTTGAGGAATTGTTGTATTATTTAAAATTATATTTGTATCTCGTTGTTGATTTAATATTAATTCTGTAACAATTTTTACATTATATTTAATATTATTTATTGTAGAGTCTAATGATGTCTTTAATATAAAATGATTATCATTAAATGTAGTATTAACTTTAGGTATGGTTTTTAAATTTATTTTTATTTTATTATTACATGCTAATTTTTTCATTTTAATTTCCTTAATTTTCTTTGTATTTATAATATGTACATATTGATATGTCAATTTTTTTTGCATAACTATATAGAAAATATAATGGATAATAAAAATTATAAAAAAAATATATAAGTATATTATAAAAAATTTAATCTATTATTCAAATTTGATTTGTTAATTTAAATAACAATTTTATTCTAAAGCCTGATCTGTATCAGCTTTTAAAATCATAAAACTACAACAAAGAGATTCATCAACACTCATTATAGCAGCTGCATTTTCATAATCTCCACAATAATTAGGTGCACTAAAAATAGTGACAAGATCTTTATCTCCAAAAAATTCATATCCATCTTGAACAACTTGATGTGCTCTAGCAATAAAATCAATATTAACAGTATCTAGAAATGTACGAACAGGTCCAACTCCAAATGTGACAGATACACCTCTATCGTTATCACCCCATCCATCTATTCTATCATCTGGATCAGCCCATAGTAAATCACATAATAATCCTTTATCTGGAATATCTGTAGGGCGAGATATGTCTTGAATTTGTCTTAAACTTCTTAAATCAGGCGATATTCCACCATGCATACATAAAATACGTTCTTCAACCAATGCACATATTGGTAACCAGTCAAAAACTTCTGTAAAAATTTTCCATAAATTTACATTATATCTTCTTTTGCATTCATCATAAAATCCATAAATTTTATTTATACTTTTTGATTCATGATTTCCCCTTAATAAATAGAAATTATTCGGATATTTAATTTTATAAGCAAATAGTAAACAAATACATTCTATAGATTGTTTTCCACGATCAACATAATCACCTAAAAATAGATAATTAGATTCTGGGGGATATAATCCATTTTCAAATAAATGAAGTAAATCAGAATATTGCCCATGAATATCACCGCAGATTTTAATAGGCGCCGATATTTCTAGCAAAACAGGTTGTTTTTCGAAAATTTTTTGTACTTTACTACACAACAACTCAATTTCATCTAAAGTTAAATTGACTAATTTGTTAGGTTTATTCTTTACACTTAGTAATTTATCAATAATACTATCCACTTTAGATATAATTTTATCATCGACTAAATCAACTATTAAATCTTTTTGTAACACCATTTAGGGAAAAAAATTTTAATAAAATGATAATTATTGTTATCAACAGATAAAAAATAACTAATATTAAAATAATAGTAATAATTTATTTATTAGATTTTAATGTTTCTAATTCATTCATAAGTTCATTAATAGAAAATACCATATTTTGTTCTTCTATAGATAATTCTTCTCCAGATTGCATTTTTTTTCTCAATTCTATTGCAATTGAAAGTTCTTTTTGAAGTTGATCAATTGTTTTTTTACAACGAGTATTAGATTTATTTCTATCTTTTTTAAAGTCTATTTTTATTTTATCATTTATATTTGATACTGACGGTATTTGAAATATATTATTGTCTAATGTTTTTTGAGAAATATTAGAAGAAGGTGTTAATATTTTTTCAGGTTCATCAGGCCCAAATATATCTACTTTGAAAAGAGTTTTACATTCCATCGTTTCACGTTTATCTCCATAATATGTATATACATCAATTTTGTTTCCTTCTTTAAAATAACTTGTAGTAGCAACGATAAAATGAGTTCCATCATTAAGCCATTTAAATACAGACGATCCAAGAGATTCAAATTTACATAAAATTTGATTATTTTCTAAAATTGTAACAATTCCAGGTAAATTACCGTATCCTCCACATAAAATGCGATTTTCACTTTTATTAAAAGCAATAAAATTTCGAATGGTCTTTTTAATAAATTTTGTTCTATAACCAGTTTTTTTGTCAAATAAAATACAATTAGAAGGCTGTTTACCTGAACAAACAAAAAAATTATTTTTAAGAAATCCTACATCTAAAATATCACCTAAATTTTTGTATAGTAAAATTTTAAAACTTCCATCATTATTATTAATTAAATAATACAGACTTAATTCAGCAAAATATGTTACTGAAGAATAATTTGTTTCCGCTAAAATTAAAATTCCTTCATTAGATTCAAAAGAAGTTATTTTATTACACTTATCAAAATATAATATATTTTTTGTATCACTTTTTTCACTTATAACATCAAAATATTCAGTAGATACTATTTTTGACGTATTTTTACTACAATTATATGTTGAATTTATTCTAAAATATATTAAACTAGATGGATTATTCATTAAAATCAATAAATCATTCTTTAGCATTGATATTTGTTTAGGTAAATTAGTAGTCTTAAAAATCATTTTTCCACTTTTAATTTCAACAATATAGCATTCAATAGATGTAAAATAATATATTACTTTATTATTAATATCAAAATTTTCGACAATATCATTAAATTCATGAATTTTAGTGTGATTTTCAAATACAATTAATTTGTTATTAAATGTTAAAACAGCACTATATATACCATTTAAAGATATTTTCATAGATTTTATTTTTCCAACATTTACTTCCATAATATTCATAGTTAACAAATCAATAAACTTTAAATGATCTTTAAAATATGAAATTAATTTGTTATTATTTATAACATAATCGATTGATTTATGAACTATAGGTTGTTTGCCAAACACATCAATAATTAATCCAATTTCAGATAATGCTATTACTGGCATAAGGCAATAAAAATTTTAATTATATATAAAATTTTATATTGTAATGAGAACTACTAATTTATTTTCCATTTACTAAATTTTAATTACATAAATACTTTATATATTGTTATTAATAACAATACCCTATGAGAATATCTACTTTGCAAAAGAATGTATTACTATTTATACTATTAAACATAACAAAAACAATATTAGAAGAACACATACTACAGGTTGGTTCTACTCCATCTTGGGTTAATTTAATATCATCTACTATTCCATGTATTATATCAGGTTTAAAGTATGAAAAATTAATTTTTTATGATTGGAAATTAATAATAATTGGATTATTTATAGCTTATGATAATTTTATAAAATTATGTATTAATCAATCTGCATCATCCAATTTAAAAGAAATTGTATCTCAAAATGCCTATATGGTTTTTGGTAATATAATAAATATAAAATCATTGGGTCAAAAATATAACTTATATCAAATTCATGCAATGATTGCTATTGCAATTTTGGGTATTGGACAATTATTCTATCCATATGAAAATGGATTTAATTTAACAGTAATGGATTTATTCAATTTCATAGGATCTTTATTAGATGTTTATGGTTTGGTATTGTTTAAAAAATATATTGAATTTACTATAAATAACAAATGGAATTATTCTATGATATTATGTCTTATTATTTTATTATTTTCATTATTATTCTTTATAATTGATATTATATTATTTGAAAGTATATCAGTTATTAAAACTGATTTTTTGCACACTTTAATTTTTATGATACTAACTGGTTGTTCTCAAATATTATTAATTCAGCTGAGTTTTTCTCTTAATCCATTTTCATTGTTAATATTTAAGCAAGTTATTGAGTTTTGCTCATGTTTTTTTTCCGATTTAATTAATAACAATTATACTAGTACTTTTCAATTTATGCTTGCTTTTATGTCAGTATTATGCTGTAATACAATATTATTATTTGATGATAATAATAATAATGATAGTTTCATTAATTATTAATTTTATAAATTAAATATATTTAAATAAATAACCATTTTTATTTATGAGTAGTTGTTCAGAAAAAACTAAAAAATGTAAAATCAATAATTTATTTTATAGACTTAAAACCTTACAACAAAATGGAATACTTTTTCCTTTAAAACCACATGTCGATGGAACTGGTTCTTTTTTATTTAATACAAATCAAATAGATATTTTATTTCAAGCATATAATAAATATAAAACAGAAGCAGAAATGACGGATAATGCTGAATGTCTTAAGGCTTATTGTAAAGCATTTATATATTGTTTAAAAATATATATTTTAATTAGAAATCATAAGCAACAATATTTTAAAAATTTAAAAAATCTTCATAAACAAATAATTTTTTATTGTGAAAAATATAATAATACAAAATTAAAAGAAATTTTGTTTTATGCTATGTTTATTATCTATCAAAATCTTATATTTAATTGCACTACAGATATAATTAAATCTAAATCATTAGAGCCGTTACAAAATATTAGTTCAATGCTAATGAATCTATTAGGATTATTTAAAATTATAAAAAATTCTAATTATTCAATTATTACTTTAGATTATCTTGAAACAGAATTATCAAAATTATATCCTGAATACTTTAAAATTCAATAAATAAAATTTATTTCAATTGCTTATATAATTCATTATATTTATCTTTGATTCCTTTTTCATTTGTTGTGACAATAATAGATTCAAATTGTGTTTCCATAGCAGATACTTCAATTTCAATTTGTCTAGGCGACTGTGACATTGAAAATAGTGATATAATATTAAATCTACTTTTAGTATAAATTCGATTTTCATTATTATATTTTAAATATTTTAAAATTCCCCAAATATTAGAAATCTTATGGTCAAAACTATTAATCAATTTAACGAATTCTGCGTTATATTGACTAAATACTTTTAAATCAACATTTTTTTGTCGTTTTCTACTGTGATAATCATAATACACTTCTCCAAGACATAATTGATTTAATTTTTTCATTTGTGAACTTATATTAGGATGAGCCACTTCATCGGGAAATTTATTATCCTTATTTGTTAATACTTCAAATCCCGAATATAATAAATATATAAAAGTAAAGAACTTATTACACATTGCATTAATATGTAATGGAGTTAATCCATTAAATTCCTGGTCTATTTTAAATCCATATTTAGTCATTATTAAAATAAAATGATCATGATTTTGAACTATAAAACTTAATCGTGAAAGACTACCTGCTTCTGGAATATATTCTAATAATTCTCTTAATGTACTTATTCTTTTAGTCATACAAATAAAATCTAATACAAATAATGGATAGTTTTTCAATCCTTTTTTTAATTTAACACCTCTAAACTTATTTAGTAAAGAATATTCATGATATGTAATATAATCTAAGTTGAAAAATTCAGGAAGAGTTTTATTTGTCATAGAGCATAATAGATGTGGTGTAAGGCCTACCCATGATATATTATATGAAATGTATCTATTTAAAAAACAATTAAACACCTCTTTTGAACAAGCACAAGCTAATAAAAAATAGGAGGGATAATTTGATGAACCAAAAATAGAATAATTAATATTAATTAATTTGTTTTGTAGAAAAAAATTTACGATATCGACTCTATTATAAATCATGGCTAATAAAAACACATTGTTTGCTTGCTTAGTTAATATAGGCGTTACTTTAAAGTTAATATTATAAATTGTATTGTTAATAATAGCTTTTTTCATTAAATAAATTTCAAATTCCCATTCGATAGTGGAATGTTTTTGAATAGTATAGTTTGTTTGAGAATGAAGCTTTTTTTTATTACCATATTTATTTGAAAGTAATGCTAATTTAATCAAAATTCTTTTTTGATCTGTAAAATTTTTATAATTTTTTACAAATTCTGTATACGAACATGTTTTATAAAAAATAGTTTTAAGAAAGTTATCATTCATATAGGGTCAAATTATTTATTATTTTCTTAAAATTACTTAATATTATAATTTTCAATCAATTAATCTAAATATATAAATGATACTACAAAAATTTGACCATGCCTCAAAATGATTTTGTTGATCAAAAAATTAAAATATATGGAGAAGCACCCAATGAGCAATTTAAAAGAGCAAAACGAGAAGCTAGAATGGCTAAAATAATTGGAAAACAATCTAGAACATTACTTGGAATAAAAGCAAAATTATTCCATAAAAGACGATCGAATGAAAAAATTCAACTAAAGAAATCATTAAAAGAAAATTTGAAACAAAAAGAAAATGTAGTAAAATCTAGTGCTATCGCATTACCTGCATTCTTATTAGATCGAGATATTAATTATGATACAAAAAGTGAATTTAATAATAAAATAAAACAACAAAGACAAGACAAAACAACTAAATACTCGGTTCCAATTGCTAAAGTTGATGGAATTATAGAGAAAGAAGTTTTCGGTGTGATTAAAAGTGGAAAACGACAAAAAAAACAATGGAAACGGATGGTCAATAGGCCATGCTTTGTTGGAAATGATTTTGTAAGAAAAGCACCAAAATATGAAAAATTTATTAGGCCCATGTCGCAACGTATCACTGTAGCTAATGTTACTCATCCTGAGCTAAATGCAACATATAAACTTCCTATTTTATCAGTTAAAAAAAATCCCACAAGTCAATTAATGACATCTTTGGGAATTTTAAGTAAAGGAACAATATTAGAAATTAATGTAAGCGAATTGGGAATTGTAGATGGAGCAGGTCAAATTGTGTGGGGCAAATATGCACAAATTACGAATAATCCTGAGAGAGATGGATGTGTAAATGCAATGTTACTTCTCTAATAAATAAATTATTTATTCATCTATATCAGCTATTCCACCTTCAGTAATAGAAAATGAAGCTTCAGATTCTGGTAGTAATGGAGAATCATATATTTTGCAAATTCGAGTTTCTCCGCGACCTTTTCTAAAATACAATCGAGTATTAGATGCATGTGCAATTATATGTCCACCAATAGGTTTTTTTACATCGCCTCCGAATACTGCAACTGCGCCATCAACATTAGCTACAACTTGATTAGTGATTATTACAGCAATTCCATATGTTTCAGCCAAATTTGTTAATGATCGTAAAAATCGAGCTAATGAAATTTGACGAGCAGATAATTCACCTCGACCATTGTAATCTGTTCGATAAAGTGATGTTGCAGAATCTACAATTAATACTGCATATTTCGTTTCACTCATCATAGCTGATGCATGAATTAATAAATTATTTTGATGATCAGAATTAAATGCTCTAGCATATGAAATATTATCTAAAACTTCATTAGGATCTAATCCTAAGCGTTCAGCTATAGGTATAAGACGTTCTGTTCTAAATGTCCCTTCAGTATCAATATACATACATTTACCATTTCCACCTCCATTCTCTTTAGGCAATTGACAAGTAACAGCTACTGTATGACATAATTGTGATTTTCCAGTTCGAAATTCACCAAATACTTCTGTAATTGTTCCACTTTCAATACCACCATTTAATAATTTATCTAATTCGGTTGACCCAGTGGTTATATATACTACTTCTTTTCTTTTTGAATGATATTCAGTTGCTGTTGTAAATCCCATTGGTACTAGTTTAGCTGCTTCTTTAATTAATTTATCAACTTTAGCATCAGAAAAACCTTTAATTAATAATAATTCTTTTCGTGGTGCAAATACTAATGATTGAACTGTATTATATCCAGATTCAGATAATTTAGATACGTCTGATGCACTAATCCCAACGGATTTTAATTCATCTATACTAATAAATCCCATATTTTGAACATTTTCTGTTTCGTCATATCCGTATTTTTCTTTTCCCATTTAAGGATAAAATTTTATGTACTTGTACTACAATAAATTATAATATAAATAATGAAAAAATAAAATTTATTAGTTTAATTATAATAATATGGTCTGCTTATATTTATTCCCATTTCTTCAAGTGCTTTTTCTAAATCAACCACTTGTAATGTAATTTTTTTTTCTTTAGCAAAACGTTTATCTTTTAATCGTGCTTTTAAATGAATTTTGTGAAATTGAAATGCATTAAGTGCTACATCTGTTAAAAATTTATGTGCCATTAATGAAATAAGTTTTTTTACATTTTCATTTTTTGTATTAATTCCATTTTTTTCTAAAAAATAATCTATTACTGAATCGGGTAATAATGGCGTATAATTATCTAATTTACTTTTCAATTCATTTTGCTGTTGAGAGTTCATGAGGGTTAAAATAATATAAATATAAGAAAATTGCATTATTATTTAGTAAGTTTTTCATCTTTTCGCTGAAACAAACAACTAGATATGGTTTCTATTGTATTATTATATTGTGTTTTTATTTTTAAATAATGCTTTGCATTATTGAAATCAAATCCTTTGACATTACCATTAAAATCAATCATTAGAAATTTATTAATTAATGATTCAATACTTGATAATGAATATCCTTTTAAAATTGTTACTAGTTCATTTAAACAATCTTTATTTGTAGAACATATTTTTAACTTTAAAAATAATTGATTTATAAATTGCAATATTTCATTTTCAGATGGGTTCTTAAAATGTTCTTTATTAGAAAATCGTCTTAAAATAGCTCGATCAATAGAACTTTGAACATTTGTAGCTGCAAAGAATATAATGGGCATTATTTGATCTTTCATATTATTAAATTGAATCATAAATTCAGACATCATTTTTTGATTTGTAGAAACATCGTTTGTAATATTATTTCTATTTGAAAACAGTTGTTCTGCTTCATCGAAAAATACAACTGTTGCAGTAAATGGTTCACAATTTTCTTTTAATTTTTCAAATAAATTTTTAATTTCAGTTTGAACTTCCATTTCTAAGGTTGACTTTAAATCTGAGGAACTTATAGAAATATATCGTATACACGATGGCATTTTATTAAACTCATTTATATCTTTTTCATTTAATTTACTGAATTTATAATTACGCAAATTTTTATTTTTTGTATAAAATTTCTGCATATGTTTTAGTTTTTTGTCTAATTCTGAACAGAATTTTTTAACATATAATGTTTTACCAGTACCTGGAGGTCCACACAAAAATGTTCCCATAGATTCTGATAAAATAGTTTTTTTAAGTTCATTTTGTGGTTGGGTATTTATTTTAAAAATTATATTTGCATAATATTTTACTTGTGATTTTAATCTTTCAGATTTTCCAAAATAATCTATATTTTGAAATTTATTGCCATCTGTGAAAATAAATTCTTTCGGAACTATATTTGGTAAATCAACTTTCGCATTCATAATTGTATTATAATTATCCTTAATCATTCTAAAGTTTTGTCCAAGAAATATAAAAAAAATACACATTATCAATACACATGATAAATATACTAAAATGAGTTTTATTAATAATAATATTCTATCAGTAATTTTTTTTGTGTTAACATTTGTCATTAATAAAATAAAAGGGGAAAGCAAATCAATAAAAATATTATTTATTCAATATATTTCATTAATGGCATAATCTTTTGCATTTTGAAAATCAAATCCAACATAATTACCTTCAAAATCAATTTTTTTAAATTTTAAACATAACGATTCTAAGAATGAAAATGATCTATTTTCTGTAAGAGCAACAAGTTGCTCTATTTCCAAAGAACTTAATTTAGAATTAGTTAACAAATTCATTAAAAAGTCCCTACGATATTGCTCAACAGGATTATTAAAAAATATTTTTTTAGAAAATCTTCTTAATATTGCTTCATCAATGGACCCTTCCATATTTGTTGCCGCAAAAAAAAATATTGATTGAATGTTTGTTGCCATATCATTTAACTGATTTAAAAATTCAGACATCATTTCACTAATTATAGTTTTAGATGCAGAAGACATTATTCTTTTAGAAAATAATGATTCTGCTTCATCAAAAAAGACAACATTTGCTTCATATGAACCAATATTTGTTTTAATTTTATTAAATAATCGTTTAATGGCTTTTTCTGATTCTCCTACCCACTGAAATTTTAAATCAGATGGTCTTATAACAAATAAATTAACTTTATTTGGATAAAATTGACAGTCCTGTTTTTGTTTATCGGTTAATTTAATATTTTGCAAATTAAAATGTTTAATTTGATCTGGATTATTTTTATATTTGTAATCAAAATATTTTAATAAACAATTGAGTTCATAACAAAACTTTTTAACAAATAATGTTTTTCCAGTACCGGGAGGACCTAATAAAAATGTTCCCATATTTTCTACATTAATAATTTTTTTTACTTCACTATTGGGCTGAGAATTAATAATATGAATGTAAGGCAAATACATTTTAAGTTTATCAAAAATGAAATCAGCTTTACCAAAATATTCAATATTTTCAAAGTTATTTACTTGTTGTGGAACAAAACAACCTCTAGAAAACATTAATTCACGTAATCTATTACCTTCTTGTTCTTCTAAGTCAGCATATATTTTATTTATAATTTTCATTTTGTCATAATAGAATAATATTGCTAATGATATTGGAAACAATATACAAGTATAAAATATAAACCATTTAATATAAAATTTTCTTGTTGTTCTTTTTTTAATAATGTGCTTAGATGTAGTACTATTATCCTTATTATATACATTACTATTATACATTATAAAAAAGGATAAAAATTTTAATTTATATTGTTATTTAATAATGATTTACAATTATTTTTCTTGTGCATTTAATATGAATTTTTCTATACCTTGTGTATCGACATTAATAACATTTCCATCCCAATCTAATGATTGAAATTTATTAATATAACATTCAATAGAAAAATATGATTGATTTTCTAATGCATTTACCAATTCGTTAAGATTTATGTTTGATGTATCATTTAATCGTTGAGTTATAATTTGTTGTATTTGATTTTTATCTGGGACATTAAATTCAATTTTATTATAAAATCTTCTTAAAATAGCAGGTTCAATTCCTTCTTTGACATCAGTAGAAGCAAATAAAAATAGTGGTTTAGGGTTGTCATTAAGAGCTGATATCTGTAGCATAAATTCATTAAGCATGGATCTTGAAATTAAATCATCATCATTAATAAATTCCTTTGATCCAAATAAATTTCCACAATCTTTAAAAAATATAACATTTACGACAAAATCGTTCATACTTTTAGTTACAGTATTAAATAATTTTTGAATAATTTTTTCTGTTTCACCTGTTAATGGAAATTTAATATCTGTAGATTTAATAATATATAAATTAACATAATTTTCATAAAATGAAATATTGTGAGCTATATCACCATTATATTCATGCTTAGGATCCATATTTAGCCATATCAATTCAGTATTTAATAAATAACAAAATCGTTTCACAAAAAGTGTTTTTCCATTTCCTGGTGGACCAAGTAAAAAATAATTATTTGATTCAGCCAATATTTTTTGTAATTCTGTTTTTTCATTATGAATAAAGTGTTTTATAACATTAAAGCTATTTTTAACAGCATTTGCTAATTCATTTTCTAAACCAATCATATTTATTTGTGAAAACTCACCACTTTTTTCATAATTAAATGTAATTTTACTATAATCTAATGGCTTTAGAGTCAATTCTTCAATTTTTTGTTTATTAATCATAGTTGTAGTTATTCCGACTTCTGATAACATGTTAGTTAAATATAAAAAAAATATACTAAATATTGGGCTATATATTAATATAATTGAAATAATTTGCCATATGATAGCTATAATATAATCTTTACGTTTTAAATTGTGAAAAACAGAATTAACATTTTCTGTATCCAATTTTGAAATTAATTCAATGGACATTAATCTATCCACTGCTCTATATGAGCATGTTGATTGATTATTATCAAATGATTTTTTATTATTAGAATCCAAATTCATAAAGGATACTAAAAATTTATTTATTATAATAAATTATTGGATAATTTAAACATAATTGTAAATATAAAAAAATTTAATTAAAATGATTATAATAAGAAATTTAATATATAAAATAATATAATAATGAATAAACTTTGTCTATCTATTAATTCATTTATAAATCTTTTTTTACAGTCATTTTTAATGTTGTCTACTTCAGTTTTCCACAAAGTAATTATTTTTTGATCAGTAGTAAATAAAACCATATTTTGCTCAAAATCCCGTGTATTACTCCTTCTGTTATAATTTGATGATCCAATAAATTAAAAGCTGCTGTTTTGAAAATAAGCAAATAACTTTTTTATGAAAACTAGAATTAGGTTTAACAATTCCTAAAATATTTTTGGTTAAACCGATAATGTTAATAGGGGGAAAACCCATATATGAGTTTTATTTGATTAAAAACCAAAGATTAAAGTTTTATTT

>Scaffold_2496

TCATATTTTAAATACATCTTCTTTAATCACAAGTTTCTGCATTTCACCCTTTTTTTCAGCCATTTCCATAATTCGTTCTTCAATAGTCCCTTTTGTTATTAGTCTATATACCGTTACATCTTTTGTCTGGCCTAATCTATAGACGCGATCCATGGCCTGTTGATCAACCGTAGGATTCCAGTCAGAGTCATAAAAAATGACAGTATCTGCTGCTGTCAAATTGAGACCAACACCACCCGCACGAGTGCTCAACATAAATATAAAATGTCTATCTGTATTTTGCCACAAATTAACTAACTCTTTTCGTTGGGAAACTTTGCAAGTTCCATCTAATCGTAAATATGAATATTGTTTTTCAATCAAAAATTCTTCAAAAAGATCCATCATTCGTGTCATTTGGAAGTATATAAGAACCCTATGATTACCTTTATTTAGCTGAACCAGCAAATCATCTAATACCTTCAATTTTCCTGAATTATTGATAAATGTATTTAGAGGGTATGTTTGAATCATATCTTCAGGATTAATTTTTATAGTATTCAAATTTGTAATATTTGAATTAAAATAATCTAAGTGACACTTTTCAGCTCTAAATATAAATCTATTTTTAAGCATATTATCAAATTGATAATTAGCTTCAATTATGTGAAATATTTCAGTGGGCTGATTTTTATAATTAATTTGAAATTGAGATTCAGAAAGTAAGTTTGAAAAGAATTTTATGCTAAAGTTATCGTTTAAACTAGATTCTTTCACCTTATATTCACTAATAATAGTTTTGATGGTATCATCGGTCCTTATAATATATTCGGGGAGAATAATTTTGTGAACGGCTCGCCGTTCAATTAGATTATTCGAATGGACAAGTGGGATATACAAATTTGAATGGGGTTCTAATTTTTCAAACAGATCGGGGTGGTTACAAACCTTTTTAAGATGCATCATTATGTTTTCATAATCTAATTTGCTATTAACTATCTCATCATATAAAATTTGCTGTCGAATACTTAAATCACAATACACATGAACTATTTCTTTATTTCCTAATTCACTTTTGATATCACTTTTGTATCTTCTAAGCATGAATGGCTTCAGTATGGTATGGAGCTTTTGAATTTGTACATCATCAATCTTTTTATTTTCAATTTCCTTTGAAAACCATTCGGAAAACTCACTTACAGAATCGAATAGGGTAGGCATAATAAAATGGAGAAGTGCCCATAATTCAGTCATCGTATTCTGAATGGGTGTTCCAGTAAGCAAAAGCCTATTTCGTGATTTAAATTTTAACAAAGACTTCCATCTTAATGATGTATTGCTTTTAATAGCTTGAGCTTCATCCAAAATCATATATTGGAACCTGATTTTCTTCAAATAATTTTCGTCGGACAATGCTAGTTGGTAACTTGTTATGATTGCATTACAATTTTTTATTTTTTTTCTTGCATTTTTACGATCATTAATCGTTCCCCAATATTCAACTACTTTAAACTCAGGAAGAAAAGTTTCGAATTCTTGTGCCCAATTATGAAGTGTACTAGTAGGTGAAATAATGAGAAATGGCCCAGAAATCCCCTTAGTTTCATATAAATACCCTAATAGGGAAAGAGATTGAACAGTTTTTCCAAGTCCCATATCATCTGCAAGAATTCCATTGATTCCCTGGTTATATAAGCTTACAAGCCAATTAAGTCCTTTAAGTTGATGCTCCTTTAATGTTCCTTTAAGGATTTTTGGTTGAAGAACATTGGTATGAACATATACTTCTATTTCCTCTCCAGCAATCTCAGCTACAAGTTCTCCATCATGCATTTTTTTAGTTTTTTTTCCCTGATCAAATTCACGTGTTGATTCAAGTTGGCGTTTAGCCGCACAAATTCCCTCAATACCTCCTTCTGTTTTGGAAATACGATTCAGAAAATAATTTGCAAATGCCTCTGTTTGATTAAGTAAATAATTGAATTTTCTTTTTTGACGGTTTTCTTCCTCGAGTTCTTCTAATTTTCTTTTTTCAACAGCCGCTTGTTTTTCTTCTAATTTGCTCAGTATTCCAAGCGATTTTTTAATACGTTTGTCAAAATTCATTAACTCTTTGTTTATCTTTTTACTCTTAAGCATTGGATTAATTTTACTAGTTTTAGAAAGACTTCTTTTGAGTTCACGTGAACAAACAGCAGCTAGCTTTTTTTTGCGTGCGAGGATGACTTGATAGTTTTGGTTGAAGGCACGCCTACTCCCAGATAATTCTCTTATAATATCCATTATAGATTCAGATGTATTGTTTATAGGTTTCTGAAAATTTTTGTGTTTACATTGGCTTTGATAATCAGCTATAGTTGCAAGAATCTTGCATTCTGAAAATCCAACCCTTTTCTCCATTCCTATTTTACTTTTTAAAGAGTCAACCTTTAAAATTAACATTTTGTTACTCTCAAAAATTTTCTTTAAATCCTCATACTCACTCTTGCGCATGCCAGGGTTAATTTTCCAAAAAACTGCGATGTATTCGAACTAGGGTTTCATATTCAAAATAAACTGTGAAACAAAGAAATATGCGAAACGAAACAAACACATATGTTATGGAACATATTAATACCACGTGTTGCTTCCATAATATCAATAATAAATGATTTGGGATTATCGAAAGATCAAGTTACACAAATACAAAATGAGTTGTCTAACAAATTTCACGATTCTGTGAAGATTGTTTCGGAAAGTATAAATCCAACAGTATCTGAAAGCATTGTAACATCTAATCCTAGTAGTACTCCATCTCCAATCTCTAATTTTAAAGATATAAAGATTCTTCAACCATTAAGCATTGCAAATGTTAATTCACAAACTAATCCTACAACAATGTCTAAAAACACTACATATCCAAAAGCACCATTATTCTTTTTTGATAAGTTAGATTCAACCAAACCGACATCTGAAACAAAACACAATACATCAACTTCCCCTACCAACAGTACAGAATTGTCAAGTGATAGTAGTTCAACATCATCAAGTGAGAAAGAGTTAGAAAAAACAATAAAGAAAGCAGTTAAACCAAGTTATAAAAAAAAGACTGGCAAATTTATCTTACCAGATTTACAGTCAGAAAATATTGAAAAAGAGCAGTTTAAGAAAGATAAGAAAGCGCATGAATTGAAACATAAATGGGCAAAGCAACATATGAAAAATGAGAAACAAGCTTTTGAGGCAAAAAAAAACTATTTGAATATTAAACTTAAAGAAAGTAAAAATACTAGTAAGAGTACAAGTGGAGAATTGGTAAAACTAAAGAAACTGAAAAAAGAATTAAAACTTCGATGTGGAAAATTTAAAACTGTTCAAGCAAATATTGACATGATTGTTGCCAAGATAGATGCTGAATTAGAAAATTTGCAAACATTTAAGAATAATGGTCAAGATGAAATAAATGATATAAAATCCAAAATACGAATGACTCAAAATGAGATAGCACACTTGTATAAAAAATTAAATCGGGAGAAAGAGCATTTACAAGAATATCTTGATCGTATTCGTGTTATAAAAGATCGTTTTGGAAAATATGAAGAACAAGTGAGAGAAAATCAAATTAAAAAAGAAATTAACCAAAAACGATACCAAGAATATGAGCAACTTGCAAACACTACTGAAAGAGCACTACTTAAGGTACAAAATAAAATTCAAGAATTAGAAAATAAAATCGCAAAAGAACAACAATATCAAACTAAAATTGAAATTGAAAAAAATGCATCAGAAGAAAATATACATTTGCCATTATTTTTGGCTACTGCATAATAAGTAGTTTATTTATGCGATTTTTTTCTGAAAAACTGCATTTCAATTTTGTAAAGTAATAGCATAAATGTAATGTGTGATAAGGTTAATTCGGGCCACAAACAATTGACAAAATTTACTCCGCATCCGCTAGCAACTTGCCGAATCATAAAATCACTCAATCGTTTTTCTCCTGAAGTACGAATTACCAAATCAACATTATCATTAAAAAATTGTCTTTTATTGGTTTTATCCTTGTCACAATTTGTACTATTGTACGAAATAAATATATTTACGATAAAGCTATTGTTTCTTGAGTCACTTTTTTCTTCTATTTCTAATAACTTTTCAATAATAGTTGCATCTTCGAAATATGAAAAATCACCGTATAATTTTAGTTGAAATGGATAATTTTGAATTTTTCCGTTTGTTATAAACTTCTTAATTGCCATGATTTCTGCTGAATCACGTTGAAAGTTTCTAATTGAAAAAATATAGAACGAAACAGAACGTAGACCATGTAAATAGCTAAATTCAATAATTTGTTCTATTTTTTCAATTCCATATTGAATTTTTGTTTGATTATTATCAATATTGTTTTTTGTGGCCCAACGACGATTTCCATCACAGATGAATACGATATCTAAGTCTTTAAATAGTTTACTTTTTTCTCTAATTTTTTTGAAGGTATCAATTTGAATATGTACTTTATTATTAATTGAAAGTGTACCATTGTGAAATTCGATCCTCCACATCCATCGCATCCACTGGAAATACATCCAAGAAAAAAGCTTATGAATAGAATAGCACAATCTATTAGTAATAAAAAAATCAATGAGCTTTAATAAATATAATTTTAAAAACAAGGAGATTCTCATGGGAAGGTAAGATATTGGATTAAATATTTTTATTCATTAAGTAAAATGATATGTATTTGATATAAAGAAAAATAGAACTATACATGTCTTTTAAGAAATAGATAGACAAGCCAGCATACAAAATTTTGTCAATAATAAATTCATATTTCAAAATACGCCAAAATGTAAAAAAATAATTGTATATTCCAAATAATGACTTGCTGGAATAAAATGCATAAAATGTTAATAGAAGTAGTAAACACAATCCATGGCATAATTCGTGTGAAATAGTTCCATCAAAGGACTCAAATAGGTCATCCATAATGGATAAATAAGAATGGTATCCGACAATAATTATAAAGAAAATTACGATATACATAACGAATATGCTGAATCCAGAAAGAACATGTATCCATAATTTATGAGGATTTCTATCATTTAAAATAATATAATTCGACTGACATTGTTCACAAATATTGATTTGGTCAAACCGTTTTGTTCCTTGAAATCTCCAAAATCTTAAACACTTTTCATGAATGTATTTTACAGATCCTCTGCATAGGCACGGACTAATTAGTCTATCATCATGTTCCTCTTCTCCGTAGCATATTCTACAACATCTATCGCTTCCACATTCCATTGTTCCTCTCTCCTTTAAGATAGATAAAAAAAAAAATAGCCAATAATTTTATTTAAAAATATACACTCGTTGGCACTAAAAGACATGAACCTATTAGCTTCCGTGGAGTAAGATGTATTCAAAATGGCAGAACGAACTATAAAATTCACTCTGTGGCTTTTGAATATATAGGGGTAAATTTTGAAATCATTTATTTCTATCAATACACCTGTCACATATCCATGGTATTTTGGGAGTTTTTATCAATCCAACACACTTAAAGTGATACCAATGGACTTCACATTCAATGTTATCACAACATATCATACTTCCAAATGCTCTTCCATTGCATGTACAATACGTTTTGTTGTCATCAACTTCATTTTTTAGTTTAATAATCATAGGATTCTTAATTTGAATCACACATTGTGTTATATCAATTGGATTGTTTCCAAGCTCATTAAGCTTTAAAAAAAAACAAATATGCTCTTTCACCTTATTTTCTAATCGATTTACTAAGGTAAGTTTTTTTTTATGCATTTTAACTAATTTTTCAGTCACATTGTGGAATTTTTTTTCATTTTTTTTAGGGAGTTTTTTAATAAGCTTAGTTAATCGAATTTGGTATTTTGAAATGATTGCATCAATGCGTTCTGTGTGTCTTTTAAAATAATATACCTCCATAGGAAATTGTTGTATGGTTCTAATAATATTATCGTATATGTCTGTAGGATTAAAAAATTTCATCACTCATGGGTACAATTTTCCCCCAATATGGTAACAAACCTTCTAGATGATTACTATGCGTCTAAAGAAGAAAATGCTCAAAAAGATATCTTATTATTGACAGAACGAATAAAAAAGTTAGAACAGGAACGTGAGTCATTAACGAAGCAGGTTAGGGAGATCCGTGAAGAAATATCTATAATTACAGACAGTAGTTCAAACGTAGGAGTTGTTTGTAACAAAATGGGTGACCGAAAATGCATGGTGAAAATGCAACCAGATGGAAAATATATTTGTAATGTTGAACCACATATTGATTACAATGCTCTCAAACCAAACACACGAGTAGCTCTTCGAGCAGATAACTACGATATCCATCGTATTCTTCCTACGAAAGTTGATCCAATAGTTTCATTGATGATGGTTGAAAAAGTTCCTGATTCAACATACTCAATGATTGGAGGCTTAGATGAGCAAATAAAGGAAATTCGTGAGGTTATAGAGTTACCTATTAGGCATCCTGAACTATTCGAAAACTTAGGTATTGCTCAACCCAAAGGAGTATTACTATATGGACCTCCAGGAACTGGAAAAACATTGTTAGCACGTGCTGTTGCACATCATACCAAGTGCAAATTTATTCGAGTTTCAGGATCTGAACTTGTCCAAAAGTATGTTGGTGAAGGAAGTCGGCTTGTAAGAGAATTATTTATCATGGCTCGAGAGCATGCACCCTCCATCATCTTTATGGATGAAATTGATTCCATTGGATCTACTCGAGGAGATGGTGGTCGTAGCTCAGATAATGAAGTTCAAAGAACAATGCTGGAACTGCTCAATCAATTAGATGGGTTTGAATCAGAAAAAAATATTAAAGTAATTATGGCCACCAACCGTATTGACATTCTTGATCCTGCATTACTCCGAACAGGAAGAATTGATAGAAAAATCGAATTTCCAAATCCCACTGTTGAAGCAAGAATTGAGATTTTAAAAATTCATAGTAAAAAAATGAATTTGGTGAAGGGTATTAATTTTAAAGCAATTGCAGAAAAATTAATAGGTGGTTCTGGAGCTGAGGTAAAGGCTGTTTGTACAGAAGCAGGAATGTTCGCATTACGAGAAAATAGGATTCACGTTTTACAAGATGATTTTGAAATGGCTGTTAGCAAGGTAATGAAGAAAAATAAAGAAAACGAAACTATGCGTAAATTATTAAAATAATATATGATCAAATTCATCTATTTATTTTTTTAAGAATGAGTTGCCATAAGGATAAATTTGGCTCTATTGGATTTGTGATTATTTGTGAATTTGGAAAATTTGTTGTATCTTTGCTATTTACTACAATAACAATCGTCCGGCACACATGTTTTTTAAAAAACTCTACTGTACAACACATAGAATTATCGTCAATGGATATTAATTGTTTGTCTTGAGGTAGAGAGATATCATTTCCAGATATTGCTAAGTCAATTTTTTCAAGACGCTTTAAATATCTAAATTTTCCGTCTGTATATTGTAGTAGATCCATCCGAGTGCAATACAGATTACTAATAATTGTGTCATAATCTTTCATTTTTGTATTATCTTTATATATAATAGGAATAAACAACTTCCATTGAGTTGAATTTATTTGGCGGAACTTATTATTTTTAAAATATCGTGCAACTGCTCCAGGAAAAATAAATAAATTACATGGTTTACCATCAAATTTGCTTAATTCAGATATCAATTTGTTAATTATAGAAGTATCGTTTAATTTTTGCAAAAGTGTTTTTTTTGATTCTTTAGGAATAATATATGTAATTCCATTTAACTCTACTTCTGTGTCAGATTTGATTTTTATTAGTTTACCAATTTGATTAATATCATCATATTTTTGAGACATGCTTTCTAATTCTATTTCAAGCTTATGCAAAGCATGTTTTGCATTATTAGTAATCCCCATAATATTTTGATGAGATTCGAAAGAGTTATTATTGGTAGGATCATTTAATGGTTCATCATTTTTTGAGATAGGTGGTTCTTTACTTTTATAATCTGTTGTCGATCTTTTTTTCACTCCAAACATTGGCTTTCTCCTTTTTACAACTTATTTATTTATCATTTACATACCCTTCAACAATATCAAACTTTTATATCATGGAAGAGTACGTAGCGGAGTCTTTTATTTTGCATTTTTGCACACACTTTGGTTTCCCAGCAAATGTAAAAAGCACAGCATTGCAACTATTAAAATGCACGCAGGGTAAAATTGTTATTGATATCCATAATATGATATATTTAGTTTTATTATTAAGCACTAAACTTGAAGATATTCAGGAATCTGTATTTCCAAAACTTAAATTATTTTACAATCGTCAACTTAAAGAAGAACTTATATACCAAAAAGAAATAGAAATGATCAAACTCATTGATTATAATTTCTCATTTAGCAATGCATATATGGCAATGTTTGGTATTTGCATCAGTTTAAATGAAAAAGGAAAAATTAATTTTCGAGATGATGATTTTAGTAAACTTGAAAAGCTATTAGACTTATGTCTAGTTGCATGCTATCCAAATTTAAAGATTGCAATCATCTTTCCGATAATAAAATATTATAATATATGCGGAGAAAAATTAAAATATCTTTTGATCACATATAAACTCGATGAACATAATATAGATGTTATGGCTATTAATGCCAAAAATGTCAAATTACTGAATAAAAAAGATTTATTTCACATATTGGATACTAGTAATAATAATTGAATGAAGTTAACGTTATTGAACATGTAGTACAGCTACTCCACGAGTAAGATTCTTAGCTTGCTGAGTTGCATCTAAAGAAACCCCATTAATGGTTAGTTTCACCTTTTTATTAAAAAAAGATTCAAGATAATTGAATACTTTGGACAATAACGAATTAGAATTAATTTTACAAGTTGTGATTGATTCCATATAGCAAATTTTAAATTGCATATCATAGTTTTCATCAAATTGTAGTAATGAAGGCAAAATACCCTCATATGTCTTTAATAGTTCTAATTCACTTGTATTTTGATTGTTTCTATCACATGAACTGTTATATAATGGATCAAGCACTTTATCGTGAGCTGTCTTCAAAACAATTTTTTTTTCTGTTTTCAAATCAACATATATTACATCTATGAAATTTTCATTCACTCCAAGAACATTCTTGTCAAACATGCCATTATTTAACATATTTAATAATTTCTTTCCTTGAAGTGTTGTGTTTTCATAAAAAATATCTTTGTCATTAAATTTTATCTCCAATCCGTTTGAATATTCTGTTATGGTAGGTAACGTTCCTCGATAATAACTTTTTGCTAAGTCGATTGCTGTTCCAAGATGATTATTAGCTTCATCAAGTGCCTTCATTGCAACGTTTCTATTACATCCTGATTTCTTCATGACTTCAATAAGATTGTGTTCTTTATTTAGCAATGTTGTATTAACTATATTTCTTTCTATATTATGACCCTTGATTTCTATTATTTGTTTTTCAGTATTTTTAACCATTAAAAATTAAGGGAACCTTTTTAATTAAATCTAAAGTTTTGAAGCAAAATTTTATTTAACGATGTTATTTTAAAATATGGTGTAACAATAGAAAAATTTAGTTATTTTAATAATTAGAATAGGCCGTGTGGCGCAGTGGTAGCGCTTCTGATTCCAAACCAGATGGTCATCGGTTCGATCCCGGTCGCGGTCAATGTTCATTTTTTTTATCCCCATGAACAAAAAGATTGATTGGGCATCTTCTTTTGATTCAAACGAATCATCATTTCCAGTATCATTATCAGAAATTCAACCAATTACCACAGAATCATTTATCTGTAATCATGAAGTAGCTTTAGAAACGAGTGATTCATTTGATATAGAATACTGTTTTTCCAGTGAATCGGAGTTGTTAACTATGTATCCTATTCATGGTCTAGAATTATTACCACTCGATGTTCAATATCATATTTACAGTGTTTTAAAATTAGATTTACTTCATAATAATGTTCTTGTTGGATATTCCCAGCCAACATATGCCTTTATAATTGAAACCATTAATGAAGCCCCAAGTTATACAGTACTTAAGAACTGCTCTGCTAAGGTTATGGTTTTAGGAATGTGGAAGAAAATTCGCTTTTGTATTGGTCAAAAAATTACACTAATGAAATGCTTATGCTGTAAAAAAGATTCTCTTTTTATAATTGATAATACTCACAATTTTTTGTTCGTTGAAGATGATGTTTTATCTGTTTCAACATTTATTAAAGCATTTAAATGTATAAATCAACCAAAGATTACAAATTTAATTGGAGATATTAACTTTAAACATGGTCATCCTTCACTAGTTTATGGGGAAATATTCCATCGGATAATTCAGTTTGGATTTAAAACAAAAGGAATTAATTCTAAAATTATACAAGATATCATCAATGATTACGTTTATACCAATGCATTAAAGCTATATATTCATGACACAACCAGAAATTTAATTGTACAACAAGTTGAATCTCAACTTCAAAATATCATTGAGTTAATTAATAGATTTTGCCATGTTGATCAAACGGAATGTCCTGTATTTTCTGATTTATTTGGATTAAAAGGAAATATTGATGCTGTTTCAAAAGATTATGTTATTGAATTAAAAACTGGAAATACTAGAGATATTACGCATAGAGCACAGTTAATTTATTATTGGCTTATTTATAATTATGAAAAAAATGTTAATGTAGTTATGCCTGAATGGTGCTTAGAAAATAATCGCAATAAACCAGTCTTGTTCTATATGCATTCTAAAGAAATAATACAAAATATACCTCGTCACAACGAAATTGCATCGTTAATTATAACAAGAAATCTTGTTGCCACAATGCAAGACATAGGAAATTGTAATTGTAATAAAAATGAAATATGTAAAATATACTATTCTATATGCAAATTGAATGACACACATTTTCTTAAAACATCATTGTTCCAATTGAAAAATGAATATAACCGTCGTACTTTTTATACTCTTTTAGAATTTAGCATTATAAACAATCAATTACAATTTGAATGCAATGACCAGATTTATGATAAAATTCAATATGCAACAACAGTATCACTGTATACTCTTAGAAAAACGTTTCTTTGCAAAGCATTTATATTAAATAATCACAAACAAAAAATCACTCTTGACATCGCATCTATATGTATTCCACAGGAAGTATTAATGTATACAATTGATGATAATGATAATCTGTATAAACTATGTTTTTATTCACTTATTAATATTGCTTATTTCATCTACTTTCCAGAACAGTATTCGAATGTAAATGATATTCAAGACAAATTTGTTTATAATACTCCATTTATATTACCTGGGCAAAATGATCATGATATATCAGTACAGTCATGCAATACAAAAAAGCAGAAATCAAATATGTATGCATATGATATAAGAAACAATTCTATATTATTACAATCTAGCATCACTCCTATTCCAGATATGTATAAGCATGCATTTTACCAATTAAACACATACCAACAACAAGCTTTATTGCATGCTATAAATTGTAAGCACTTTGAAATTATTCATGGAATGCCCGGAACAGGAAAATCTACATTGATTGTATTGTTAATCAAAATTTTAGTATTTTATAACAATAAAATCTTGCTTATTTCGTATACTCATTTGGCAATTAACAATATTCTTTGTAAATTAGATAACATTAACTATTATAAGATTTGCAAAACAAAGCAAGATCAATTCAAAGGTATGGAACCAAAAAATATTAAAGACTTTTATGATAAAATTGAAGTAGTTGCTGGGACATGTTATTCATTCTACGATCCAATTTATATACAGCGAAGTTTTGATTATTGTATTATTGATGAAGGGTCACAAATAAACTTATTGATTAACCTAATACCTATATCAAGATGCATCAAATTTGTTATTGTTGGTGATCATTTACAGATTAGTCCTATAAATGGCGTTGGATTAAGTTTATTTGAACATCTATATACAAATTATTCATGCAGTGAACTTAAAATTCAATATCGCATGGGTAATTCAATTATGCATATCAGTAATGCAATGTTTTATAAAAACAAAATGATTTCTGGTGTTCAATTCAATGGATGTATCAAATTTCACAATACTGAAATAGAACAATTAAATGATATTTTATTACAATATGAAAATGAACAAGCCACTATATTATGTTATTTTAATTCGGTAATCAAACAATTACAGCATACTCGGATATCAAATAAAAACATTTTTACAATTGACAAATTTCAAGGTTCTGAATCAAATACAATAATATTACTGCTTGATCCAATAACTAATAATGAATGTCAGCTAGATCCTAAACGGTTAAATGTTGCATTAACTCGTGCTAAGCAAAAATTAATTATTATTGGTAACAAAAAAAAAATGGTAGAAATTGATATATTCAAAATATTATTACAATATATAGTTAATTTATAAATATATTTATTATTCATCTTGAATTTCTTCGTATGACATTTTAAATGTCAATGGTTTAAATTCAACACCTTCACCATTGTAAAATTTTCCAAAAAACTCAATCCAATTTAAACGGAGAGCATGAAGACAGCTTGATAATCCTTCAAGACCGATAAGTAAAAATAGGGTTGCCACTATATATACAGGAAGTATAAGAATTTTGTATAATAACCCTCCGCTCCCAATAGTAAATTGATGTAATACTGTTGTTAATTGAACATGAGCTAACGAAACTGCCCACAATCTCAGATAACTTGATGTGTTAGAAATTAACCCTAATCCAAATTCAATAGTGTGAATACCTGAATTTATCCATATATCTAATATATCATCAGATTTGATTTTTTTATTATAAATATAATATATCGGTTTACTAAAAAACATCCATGGAATGCAAACTAAAATCAATCCGAAGATTCCAATCTGAACATAGATTTGACCAGGATACATTTGATCTTCGATTGCTAATGGATCTGTATACATCCCTATAAGTGTTTCAACTAAGCTAGGATAATTAATTGTCACAATCCATTTATAAAAGCACAAAAATACTAAATATCCTAAAAATGCAGTGAATGCAATTACTTGTGGTAGTACTATACAGTAAAAATTAATCCACTCTTTAAAATATATTGTATTAATAATAGAGATTGCGGATCCTAATGACATATGAATGAATCCAATAATTAAACTCATCTTCATTTTCAATGAGTTTGTAAACGTCATAGAATTTTCAGCATGATGCCATATAGGATCTATACCAAATGGATATGTTTTTCCACTATAAAATTGAGATTTAAATAATGTAATAGGAAGTCCAGCAAAGTCACCATATAATAATCCAAACCATAATGCACTCACACTACAGCATAAAATAATCCATCGTCCATTGAGCAGTATTTGAAATACTCCACAATTATGATTCAACCTTTCATAATTTTTTATCATATAGGAAGAAATAAATAACAAAATAAAGCCGTGAAATACATCTCCAAACATTGCACCGAACATACATGGAAAAGTAAATACCATGAACACAGCTGGATTGATTTCACGATATTTAGGGACTCCAAAGACATTTGTAATATTTTGAAATCCTTCCATAAATACATTTGTTTCGAAGGCAGTAGGAATAGTTTCTTCAGGACGAATTCTAATTTTCTCACAAAAAAAACGATTTTTATAGCTTTTACATCTTTCATATATTGTAATTAAATCAGACTTTTTAATCCATGCTTCTCCTGTATAAAACACATTATTAATTTCGTGAACATTTTCAAATGTTGTTTCAACATTAGGAATTAATAAATTAATTGCTTCGTAAATTTTTCTTTCCTTTTTTACAGTTAAAATCCATGCTTTATATTTTAATTTAACAGATTCTACTAAGGCTAAAATACTATTCTTTACTTCATGATATTCCTCGTTAACTTGTCTAATATGTTTTTTTAATTTTAAAATATTCTCAATTGTATATTTATCAAATAATGGTAAGGTACTTTCCTCAGTAGATGCCATTTTATCATATGTATCTGCAAATCGTCCGCCCATAGTTGCTATAATATTTCTTACTTTAGACTGAGCACTATCTCCATATACATAAACAACAAGAATTGTTTTTTTAATATCATCAAATGCACTATCAACATCCTCCATCTTTATGTATATATTGTTTTTCATTAATGTTTTGAGAATTTTTTTAATAAGAAATTTTTTATCACGTTCAATTATTCCAACAAGAAATATATCGGACACTTTACTAGACGTATACTCTATAAATTGATATGTCGTTTTATAAATTTGATATGCTTCTTCAGCATAGCTTCTAACTGTCCGAAGTTCTTTTTTTTTATGCTTTAATCTAACCATTTCTTTATAAATCTGTTCGATTTCTGCAACTGTATCATCATAGTCCATAATTTTACCGCTATTATCAAATATTTCAGGTTTTAAATAATTTAATTGTTGTTCTATCTTTTCAATAGCATGAAGCATTAAATTATTTGATTTATTTCTAATAGCTTTAATCTCAATTTTTTCTAACAATCCTAATTCCTCAACTGTTCCTTTTATATCATTTCCACCAATGTAAATAGCAACAGATTCCATTTCTTCAGAACGTAACATTGTTTTTCTAAGTATATTTTTATTTATTTAGATTTTTAAAACACCTTTAACAATCTAAATTGCACTTGATTCGAGTTGCTTCAACCATGTGCTCTTCTAAGCCATTTTTTTTCAATTCCACAGATTTAATTGCAGATCCCCGAATAGAACAGAGTTGAATATTTGTTATGGATTTATTAATTTTAATAAATTCATTGGTGCTTATTGCTATAACATTTTCAATTTTAATATTCAAAAATGGATCTACATTTTTTAAAATTCCTGAGATAGATATGTCATTTTTTAGATTGACAATAATTTCATTGTTTAACTGACTTTTGAAATACTCAAAAAATAGCATACGTAGGGGCAACATTTTCATGTGTTAAAGTTTTAACTTCCCCATGAAATCGATAATATTCAACAGTGAAATAAAAAATGATCATCTAATTAAAATTGCTGTTCATCATACATATGGACTTATAAGTGCTATAAGCCAAAGGTCAATCCTGCTGTTAAATCATGATATGAATATAGTAGGCCAATATTTTGATTCAGATCCATCTGAAAAGTACTATTCCCTCTGCTTTATTGACTTAAAGTATAATTGGAATATACAACACATTATTTCTGATCAAGTACATGAAAATGAAACACTTACAAAGCCATTTGTGGCAGTTGGTGGAAATAGCGGATTAATTAAAATTGTAGATATTGAGACTGGAAAATTTGCACAAATACTTCGCGGTCATACTGGAATAATAACAGTTTTAAAAAGTATTGATCACTACATAATTAGTGGATCAGGAGACAATACGATCCGTATATGGGATTGTCATACAGAAACATGCATAGGGATTATGGGAGGGATGTTCGGGCACAAAGGTACCATCCTTAGCATAGATATCCATTATAGCCAAAAAAAGATTATTAGTGCAGGAATTGATTGCACAATTAAAGAATGGAATATTGAACCCTTCTACCATTCTGATAATGAGGATAATTATCTTCAATCTCCATTATACACATACGAAGAACTTTATAATTCTCCTATAGTACAGGCAAAATATTATGGAGACATTATTATATCTATGTCTGATTATGTAATGATAGCTGTATTGTCTAATAATTTGTATAATACTATCCATTCTAATTTACCTCAATTTATTAATAATCAAGATATATTATCCATAGACAAACCATTTATACTTGGACATATACAATTAAAAACACCATGTCTGTCATTTTCAATTGTTCAAAATACATTTATTGGACTAAATACTAATGGCGAAATATTTTATTTTGATTTATCATCTGGATGGTCACAAAATACTTCATTAATTAATTATTTAAAATTAATTCATTTTTTGGATTTTGTATATGATAATAATTTCATTTATATAGTTCATGGCGATGGAATCAGCAGAGAAAATATCAGTCCTGATATAATTAAATAAACTACAGAATCGGTTTCATTGTGTTTATTATTTCTAAAGATTTATCCTCCTCATTTTAATATGAGAAGAGCAAATAATAATTTGCAACAATTATATGATGAAATAAAAATAAAGTATACACAATTTATAGCTATAACTTTCGAAGAAAATAAAAAATTAGTTCATTATCTTCATACGAGGAGTACTCAAAATTCTTATTGCAATTTGCAAATAGTAATTGCACAAAAAAGTGTTCTTCCATATATTCAATCCGATCCAAAAACTAATTTTTTAACATACACTAAGATAAACTTGTTCACAAAAGACAATTCAATGTTTAAATATAAACCCAATATTCAGATACAAGGTCTAAAATACCAGTATGAATATAGTAAAGATATTGTAAATAAACCACTGCTATTTACGACTGACATTATAAATCTAATTTTCCTTAAAATTATGCATTATAATCGAGAAGCACATGAATTTCTAAGAATTCCATATGCTATACAAAAGCCAAAATCATTTCAGACCTTTTTTTGTAATACATGTTTGTTATTTAATTGTGGGATTCATAAAATCACACCTGGAAAGTGTTTGCACGCTAAGGAAGTGTCAGGGTGTATCTGCTCTGATACTAATAGTAATGATTATACCGAGTCTTATGTAAATAACAACGAAATCGTCCAAAAAGTTGGATTATTGGATCTTAAACCCTGTGTTCTAGTAAAAATTGTTCAAATGTTAAATGGGCCTAAAATAAACTGTGATGTGTTTAGTTTTAAGAACCTTCCTCCCATCAAAAAAATTGATAAAAACTATAAAGAAACATTGCAAATGGAATATTTTGTTCCTTGTAATCATAATGGGCCATGTAATAAAAAAACATGCACATGTGTACAAAAAAACATATCTTGTGAATTGTCATGCTTTTGTGCTAATTGTACTCGGATGAAATTTTGCAATTGCACTAACGCATGCCAAGAAACATGTCTTTGTCATCGTCATGGACGGTTTTGTGATCCCAACTTTTGTGGTTGCACCCAATATTGCGATTGTACAAATAAATACACAACTAAATACAAAAAAACAACAATATTTAAATCTATTTACCATGGATTTGGATTGTTTAGCAATGAAGATATTATTCGAAAGGGTGAATACGTTATTGAGTATACTGGTGAAATTGTAAGTGATGGAGAAGCTGAACGACGAGGATATTTCTATGAAATGAATAATCTTTCATATTTGTTCAATATGGCTAATAAAGGCGTAGATGTAATGTGGTCGATTGATGCATTTCAAATGGGAAATGAAAGTCGATACATTAACCATTCTGTAACAGATGCAAATTTAAAAACATCTGTTAAGATTGACAAAGGAATTAACAAAATAATTTTATATGCAATACGAGACATCTACAAAGGGGAAGAATTATTGTTCGATTATAAATTTACAGAGGAATATCAGAGAAAGCATGGAATGATTAGTGATTAAATTTTCATCCCCCTCATGGTGGAAACACGTTATTTTAAAATTATAGACCAAAAAAAGATTCTAGATAAGGATATTAAAGGATTCACAAATGATATGACAATCGTATGTGGTAGAGAGAATATAATAAAGGTACTTAATGAAGATCAAACATATTCAACACTTCCTGTAGATTCACATATTAATACATTAATTTCTGATGAGGACACAATATTTTGTGGGTGTCAAAATGGAAATGTATACAAACTTAAGGATAAACAACTTCACTTTTTATATAAACATTCAAATAATGTGTGTTCTATAGATTTATATAAGGAATATCTTATAACTGGTGGATGGGATCACAATTGTATTGTGTATAATACAAAAACTGGTATACAAAATCACATCATACATCCTGAAAGTGTATGGGTTTCTAAACTATATACAATCGATCATGAATTGATACAAATAATAACTGGCTGTGCTGATGGAATAATACGAATATTTATGTATAATTCCAATGGAACATTTACGTTAATTAAGCATTATGATTATCATAATTATCCTGTAAGAGATATTATTAAAATAGATCAAATCATTTATTCTGTTGACAATGGTGGTAAAATATTCAAAATTCGTTCTGATGGAAAATTGTTAAGCATGAAAGATACAAAAGAAATGTGTTTTACTTCATTATGGTATAAAAATAATCTTGTTGTTGCTGGAGATCAAGGGAAAGTATGGTTTTTAACGCCTCATCTGGATGAACTTTGCCAAATAAAAATTCCACAATGTAATGTTATTTGGAAATTACATAATACCGACACAAATATACTAGCTGGTGGTAATAATGGTACTATTTACCAAATTAGAGAATGTAGTTATCAAGAATGGAAAGATTCCAATATATTATCAAAAGAATGCACTAACGAAATGAATAACTATAATGTAAAAGATCAAATATTTACAGCAAATAATCAAAAATATAAAATATCAAATAATCAAGTTTTTATCCAAACAAGTAGCGGAAAATGGGATTTGATTGGAGATGCAGAAAAAGCGTATGATTATTCGTTTACTGTTGAAGTAGGTGGGAAAAACTATACTATAAATTTTAATAAAGCAGACGATAAAATGATGATTGCTAGAAAGTTTCTAGAAACACATAATCTTTCTTTAAATTACCTTGAAGAAATAATAGAATTTATCAATGCAAACTTTGAAAATACAGAGTTTAAATTATTTACTACTTTGAATATTGGTGGGATTCAAAAAGTTCTTCAAAAACAAAAGCAAGGATTGGATGTAGATTTTATAATGGAAACATTATGTAAAATAATGGACAATGATCTAGATATACAGGTTAATAAGTTAGAAAATGAATTAAAGAATATTGAAGAGAAATATGTTTTTTATGATATATATAAATATTTAGTTTATAAAAATATTCCTGTGGATTTAAGTTTTGTTCTAAATGATTTCATTTTATATGAAAAAGATGCTCGGGCATTCGTAATGCTTATTACGAATTTAATGGTAGATTCTCCATTTAAATTAAATTTGTTAGACAAGAGAGTGTCTGCTCTCCAAGATCAAGAAATGATTAGTTTTTCTGATTTAGAATATTATAATAATAATAAAATACTTCTTAATAAAAAACATAAGTATCGCTCATCAACTTAGAATATAAAATGTTGATTATGCAATATTATGTTTTTTTTAATAAAATAAATGCAATTTTTTTAAATTATAATATATTTAATACTAAGATAAGATGGTAAATTATTTTTCCTTAGCATACCCATGTTATATATTAAATTACAAAAAAACTTATCTACACTTATAAATCATTTTAAAGAACTAATTGACATTAATGAATCAGTTGTTTATATAAATGATGAATCACTTTTTGAGGCTAACACTTATCAGATAGTTGTTTATGATAAATATGGGACTAAAGAAGCTATTTTTAATAGTTATGAAGCATTTTCTCCCCATTCAATCGTAGAAACAATCTTAAAAAAACCAATTTATGTATACAAAATTCGATGGAGCGTAGCTAATTATGTGCATGGAGAAATGAAAGTAGGAAAAATCTTTGATAAAAAAAATAAGCTTATAGGAATTCTTATTGAGTATCCAGATATTCAAAGTGTACAAAACTTTATAGTACATTTAGGATTCAAAGTAGTAGAGTACGACAAATATATGCATGAAATGTTTATTAAATATATTTAATGTATTTTAATAATATGCTATTATTTATGTTTCAATAATTGTTCCCGTAATTCCGCAATCTCAGATATGAGATCACAATTTTCTTTAAATAATTTTTCTTTCTCAGTTAAAACGTCATATAGACACGTTTTCATATCTAGTAATATTTGATTTTCTTTACCTTCTAAAATTTCAATACATTTTCCTAGATCATATTTTAAATCTAAATCCTTCGTCTTCAGAAAACAATTACGGATATATTCAAGTATATGTTTTTTCTCAGAAGATATAGTATTCTTCAAAGGTTTATGCAATTCATCGCTAGGTTTATTAGTTTTGTCCGTAATTTTTAACGTCATTAGGGCTGAGGAAATTGTATGAGGTTAAAGTTTTTGGAGGGAATAAGTAAAAAATACAATCAATACTTTACTGTTTTTTCGGTAGAATGACAGGAAAAAGCATTCGGGAATAGTATCATTAGAACACTCGTGATATTTTTGATGTTTTCTTTTGAATCGGTATGAATATCAACCTTATGGGTTTTTAATGTAAAACGTACTGTACCCTTATTTATATTAGTTATACCAATTTGTGCATCGGTAATATTAATGGTACTTACAGGCTTTTTTGTTTTACCATTAAAAAGATATATTGTATATCCATACCATTTTGCATATCGTTGTTTCCATAAGAATCGAGTCTTATCAGCCAAATCACCCTTCACATTAATATATCCGCTGAAGAGCAGATCATATGCATATCTCCTATTAACAATCCATTTGCTTAGAGTAAGTAAACTGTATGGCTCTGGTGCCTGAATCGTGAAAAGTTCATCTTTAGATATGTACGCACAATATAAAGTGCATGAAGCTGCAGCAGGAACATTATCAGCAAATAAACTATGAAATAAAAACAAATTTTGATCGTATTTTTTAAATGTTCTAGTTGTTTCACCTAACCGATTATGAATACCTGTAATAGTTTTTTCATCGAGAATGAATTCACTTACCGCTATTTTTTTAGAAACTTTTGATGCTAAAATAAAAATTTTTATTTTTAATTTGTTTTTCATTGATGAAATTGGTATTTTAACATAAAAATCACTTCTTATTGTTTTAATAACATTATATTCTGTAGATTCATATATAGTTTTATTAGCTTCAATTATAAGTCGAATAAAATCATCTTTAATACGCTTTTTAGAAGCAGAATTAATACTTTTAACTATTAGATGTAACTGGCCAGGAAATTTATTAAAATGAAATGGTCGTAATCGATTTAAAGTATCTGTTCTACTTATTTGTGATGGATCGAAATTGTTTACTGGTATTTTTTCTATTGTTACTGAAGTATCGTGGCTATTTTCTAATTCATCTATTAATGAACTATATTCTGGTTTTTGTAAACTATCATGAGTTAAAATAGTATCATCATCAACAGTATTTTTAAAACGATTTATTTGTTCATTAGAAACATCAACAACCAGATTATTATCTGTGCATGATATGTTGCACTTGTCTATAACTTCTGTTGTTTCTGGTAGCATAATATTGTCATTCATTTTTAATTTGTTTGAGAACTGTTGCATAGTGTTACAAATATTTGGCAGTTTTTATGAGGTATAAATTGCATGATTAATGATCTTACTGCATATTTCAGGATATACCAATTAGAATATCATCAATATCGGAAAATAACAATTGACTTAAAACAAGAGTTTGTAAAAAGAACTTCATACAGTGGATATTGTGTTTTTTGTGGCCAACTTAATGCAAATAAGGTATGTAATAAATGTATGCAGTATAATCTTAATTGTCCAGAACTGGCAATATTAAATAGAAATGAGCGATACCTTCATGAAATTGAACTAATTGAAAAAATGAATGTCTATATTAAACAATATCCAGCATATGATTTTAAAAAATATGGAAGTCAATATTTTTTTAATATAAGTCAATTATTTCATCAAATAGGATATAAATCTAAACAAAAAACACACTATAAGACTATTATATCTGATGAAATTGTAAATATTTATAACTTTATTGTATATGATGATGAATCCATATATATGTTTCATCCCAGCCAAGATACATTTTTACTCAGATTTGATAAAATTCAATTTGATAAATTAAAAAAAGTAAATTTCGATTTATATGTTATATTTACACATTACAAAACCAAAAAGACCATTGAATATTTAAATAATTTTTATGTAAAACGTATCGAACGTAAAATCAAGCTAATTGATGAACAAATAGAGTCAAACCTTCAAAATTTAAAGACAGCCACTATTAATCAGCATACTGATGACATAAAAGCATTCAATGATATAAATTTTAAATTATGTGAATTCAAAGAAGATTTATTGCGAAAGAAGTTAAATTTTATTTAAATTGAAATATATATCCGATTAGATTTTTAAATATGATTTTATTGTTTTTGATAACAACTGTGATATATTTGACACAACCATTTCATTTCTTGATCTCACACGAGTTTCAATAGTTTTATTTCCAGATAAACAAATATTTATTAGGACTGGTGTTTTTTGAATGGTATAACATATAAAATTACACACACAGAAGTTATATGCATCTTCTTTATTACATATATGGCCATTGACAGATTTAATTAATATTTCTAAAATTTCATTCATATTATTTATATTTGCATTGACTGAATAAATATTTTCCCATTCAAGTTTTCGCCACTCTGATTGAAAGTTAAAATTGAGATTATACAAACTTTCACCCATAAGGAATTCCCTAATATCAAGCTGTATTTCATTTAAATGTTGGATATATGGAGTATGACTATAGTCTCCATCTTTTAAAAATCTGAAAATGCATGCCGCTGTTATAAAACTATTTGCAGATTCAAGAACATTAAATACAATTTTTCTGGTTATTGCAGACATTGGTTGAATAGTTGTATTTTGTAAAGATTCATTGAGCTGTAAATGTGGAGAATAAGTGAATTCAATAATAAATTCAGGAAGATAAAATTCTGTTTGATTAATAATTAACATGTCTAAAATGCATTCATATCGTGTGTAAATCAAGTTGCATTCAATATATAAAGGGTCTCCAATTCCACTAAGTTGAATAACTTGCATTTTAGTTTTATCTGTTTCGGCCCAATCAAACTTTTTTAGTGATGATGGTTGATCAAAAAATGGAATTCTAAGTGGGTCAAATAAGTTGCAATTAATAACTTTTTGTTCATATATCGATTTCGTATATTCTGGTATTTTATTTGTACTTATTAAGTATCGTAAATACAAATATATTGTGCTTTGTGTTGAATGATCTATAAGTTGATTTTCTTTTCCGTACTGTATAAATTTTAGACATAATGCTGCTGTTCTTGCTTGAACATCTTCCACATTAAACTCTAGTTTTTTGTAGCTTTTTACTATGAAAAAACATATATACCCACCTAAATATATTTCCGATTTAGAACTTAAATAAAAAATATCACCATTTCCACATTCAATATTATTTTCAAATTTGTCAATCAGTTGAAGAAACATTTCTTTATTAATATTATGGTAAATAGTATCTAAGGCAAATCTAAATATTTTGCCATATTGAATGCTATCTAATATTTTAATTAATGATTGAGCAATTGTATGGTTCATTGCAAATTTATTTTTTTCTAATACATTTAGAAATTTAAGACTTGAATATTGAATTTCTGGAGATAAACTGCAACGATTGTCAACAGTACCATCATTTGTATACCCTAAATTATTAAGAGACTCATCGATTAATTCACTTTTGAATATTCCATGAGCATTAGTAAACTCAGTTATTTTGTCTAGTAGAACAGTTTGAAAATGTTTTCGAATATCGCCTGAACTCATGTCTTTATATAAGTAAAATAATTTGTCACTAATTTTAATATATTCCGATGGTGTGGCTGTTTTGATAACCCTATCAATTATTTTCACACTGAATTGAACATTAAATGGATCTAATAAATTCAAAAATGGAAAAAGATCGTGACGGATATTCTCTATCATATTAATCATATCTAATTTAAAATAACAATTCACGCTATTTGCCATAATCATGTACAATTTATGCATGTTTATTGGTAGATGCATTTTTAAAAGTCTATACATGGCCTTAAAAGCTATTGTATCATGATTTGCTGTAGAACATCGTTGTAGTAATTCAATATCATTTGCATGCTCTACTAAGATCTCTTCAATTTCTGAATCTTCGTTATGATGATTAAACATGATATCAGTTATATCTGCATTCATTTTCAAAAGACTACTCAAAATGATTGATTTAACTTTGATATTTGTTTCTGTCTTTGCAAGATTTATTAATTCATCTTCTTCATCAAGATCAAAGCGGATTGAAATGTCACAAAGACATTTGACTGCATTCATCCGAACATAATAATGTTTATGAACTAGATTTTCTTTAACTAACTTCAAATTTACGTGATCATAATTTTCAAATCTAGATACCAATTTTAAGGCAAGTCCTCTAATATATTCATTTGGAGATGTTAAATCTTTGTTTATTTGATTTACACACATTACAAACTCTGGTTCATGCATCATAGTTTCAAAATAATAATACAGCAATCGTTTTGATTTGTTATTATTTAGTGATGCGGAAATTATAGCATGAAATATTTCTTTATTAAACATACCTACTGATTGCTGGCTAATAAGAGTTTTTAAAGCATGATCACAATTTTTTTTAATGAGTTTTGCTTCAATTGGTATTTTTTTTGTATCAATAAATATTGTTTGCAACATGAGGGGTTAGAAACAGAATAGCTAAAAGTTTCTGACAATGCAACTTTTTATAAATAAGCTAATTTACAAACACAATAATTTAATGAGTAGTAATATTGACTTGCTGATGGCATTATTATCTATATTAGAATTATTTTTAAATTTTTTTATGAAATTTTACCCTATTGAATGGCACAGCTTAAAGAACGAGAAGTTTTAATGATCGCAAAGGTTCCAATCGAAACTATTCGAGGAATTGGAAAGAAACAAATATTAATATCTCACCGAAACCAAGCTGCGGAAATATATTCTCTCAGTCACGATGAAACACCAAGCAAAATCCAAAAAAAGTATAGACCATCAAAGAGTCTTGTATCTGATGCAGTATATGCAGAAGAAGGCTCAAAAATTATTACTGTATCCCGTAATACTTTGTCAATTTTTGATAAAGAGACAAAAGATTGTGTGGAATTTAAGGGACATTCTCGGGATATTTCATGTGTTACAATAAATAAAAGTAATACTAAAATTGTTACCGGGAGCCAGGATGGCACATTTATTGTGTGGAATACACAAGGACAACAGATAGAAAAATTTAGTTGTAATGAGGGAGGACACCAAGGATGGATAAATAGTGTCGGATTTATTCCTGGATCTGATGAGTTGATAGCAACTGCATCTGAAGACGGATCTGTTAAAATTTGGGATTTGGAAAGCAACACTCTCCTAAAAACATTTGTTGACGGTCAACTTATTGATTTGGAAAAACTTGCAGAAAATAAACAGACATCCCCAATAGGAAATACAGATTTTGCTGTTAAGTCTTTGTGTTTTTCAAATGATGGATCTCTTATGGCATATGGAGGGCGTAATGGGAAAGTATACATTATTAATCTTGCAGTAAATGAAACACTTCAATCTTTAGAAGTTCCGGGAAGGATTACAGCATTAGCATCTGGAGAAAATCAACCTCTTATTGCAGTAGCTATTCCGAATAAAATACTCTTATGGAACATTATTGAAGAAAAAATAGTAGCTGAATATGGATTTAATTACAAAAATGAAGTTTACTGCTATTCAATGACATTTGTTGAAGATGAATTGATTATAGGACTTACAACAGGTCAGATTATTCGACTTGATATAATAAAAAATTAAATCTATTTTCGTTTTAGTTTTTTTTTAAAAAAAAATTTCCCCGCCGGGGAATCGAACCCCGACCGCCAGAGTGAAAGTCTGGAATCCTAACCGTTAGACAAACGGGGATATTAGGTTCACATTAGTTATCCATGTACTATTGTGTTTATAAATACCCTTATGAACAAATGGGGATTTCCATATGAACTATATGACATTCAAAATCAATTTATTGATGATGCAATTAAAGTAATTTCAGATGGTAAAATTGGAATTTTTAGTTCACCTACTGGAACTGGAAAGACAATTTCACTTTTGTCAGTATGTACCAATTTTGCATATCACGATAATGACAATTTATATAACCTTCTATTTTCTGCAAACAAGACTAAAATATATTATTGTTCTCGAACACATACGCAGCTTGCACAAGTCTTGCATGAACTTGATACAAATATTCATCATTATAATACTGTTATACTTGGATCTAGAAAATTATATTGCATTCATCCCATCCAAAGTACATGTAACGATATTGAAGAATTAAATTCTAAATGTCATGAACTAATTGTAAATAATAAGTGTGAGTACTATAAGGGTTCTTTTTATCAGTCAGGTAACGCCAGTGTCAATGAATTACGTCAAAAAGGTTTGTGCGAAAAGTTTTGTCCATATTATTATTCTAAAGATAGAGCTGCAATGTGTGAAATTGTTTTACTTCCATATAACTTGTTATTCACACAAGAAGGACGAAATAGTGTAGATATTGATATTGATGGAAAAATAGTTATTGTTGATGAAGCACATAACATATGTGATACAATAATTGAATTAAATACTGTAGAATTAATATTTAACGAAATCAAAAAAATTGGTTACTGTAAGGGCATATCCTCTAATCTTAAAGATATCATTACTAAAATATTATATTTTCAAAATGTAACTACCAACATTTCTGATACTATTATATCAGTTGACGAATTTATCCATAAAGCAAGACTTGAACATTATAACATGTTCGATATAGAAGAGTTCATTAAAAGCAACAAGTTAGCACAAAGAAACAATAATCCATTTATTTTCAAATTTAGTCGATTTTTGAAGCTGTTAACATTTAGTAACAGTAATGATATAATAGTGTATAACAAAACTAGAATAAAATTTACACCATTGAGTCCCCAATTATATCTTAAAGAGCTATATAAATGTAAATCGGTGCTATTTGCAGGAGGTACAATGGAACCTATTGATCAATTAGCTTCAATATTTGACCCAATAAAATATTACAATTATCATGTCTCCAATAGACATATCTTGCCAATAATATTAGGAACAGATATTACAAATAAAGAAATCTGTCTAATTTATGACAATCGAGATCGTTTGCTTAACCCTATATTAAAAACACTATATTCTTTATCATATATTGCAAAAAACGGAGGAGTCATTGTTTTTTTTCCATCTAAAACATTTTTGGAATTAATTAAACAAAATATGGATGTAAATTCTTTTAGAAAACCTATTTATTTTGATGATTCATTCGAACTATTTAAACAAAACCAAGGAATACTTTTCACTGTGATGGGAGGAAAATTAAGTGAAGGAATTAATTTCGAAGATGATGCTTGCCGACTACTTATTGTTGTAGGCGTTCCATTTCCTTCTAATACAATTGAATTTAAAAAGCGTATCCGGCATGATAGCCAATATGGAATAAATCAAGCTATGAACAAGGTTAATCAAGCAATAGGAAGAGCTATTCGTCATGCCAATGATTATGCTGCTGTGGTATTAATGGATATAAGATATAAACGATTTACATCAAAACTAAGTCCATGGATTAATAGTAAATTGAAGATACTAAATTGTTGTCAAACACTTGAAGAAATTAATCGTTTTTTTAAATCTTTTAATCACTAACTATTCTAATAACTTAAGAGTCAATCGAGGGCCGATTTCGGCTAATTTTACAACATATTCAACATCGTTATCACGGACATTATAACAAAAATGTCTCAAATAAATTTCGCCATTAATTACAGAAAATAAAATGATACGTTTGAATCCAATTTTAATATTATCACTATACATATTTTTTAAAAACATTTTAAGTTCATTCATCTGTCCAAAGTTATTTAATACAACCATAGGAGAATGGGTGTATAATGTATTTGCAAATGTTTTAAAATTATCTTCATAATCTACAATTTCAAATTGATAGACCTTTTGCGTTAAATTATGTTTCATTTCTAAAAACATATTTTTCCCTGTTTCCCAAATATATATTGAATAATCAATGTTTAGATTCTTATTTAGTTCTATTATCATTTTTTGTGTGAAACACAAATTCTTTATACCACTGAGGCCATTTGGTTTAAAAAAGTCACGAAGTTGACGACATAATACAATACAATTATTAGACTTTTTTTTTGATGTGTATATAAGTTTATTTTTTAATTCGTTTATTGATTCTTTGCCACTAGTTTTAAATTGAGTTTTTGAAATTGGCTGTAAAAATTTCTTTTTACTCATACAGGTGAAAATTTTAATCAACTTATTATTTATGCTGACCATTTAATAGTTGATAAATCAATTCCTGACTTTACCATTTCATACAATGGAGACATTTCTCTTAAAGATATTACTGCATTTGCTGTTTTTTCTGCTAATTGTTTGACTGTAGTTTCATCATTAAATTTATTGAATCCAAATCGAATAGATGAATGAGCTAAATCTTCAGATGTTCCTAATGCACGTAGTACGTATGATGGTTCTAGAGAAGCAGATGTACATGCACTTCCTGAAGAAAGTGCAAAATCCTTCAGCTTCATTAAAAGACTTTCCCCCTCAACAAAGGGAAAGGACAAATTTAAACAATCTGGAACAGTATCATGCCCATTTTTAATAAATCCATCCCCAAGTTTATTTGTTAAAATATTCAGAAACTTACTATTAAGTTTTGTAATCTTATCTGCTGTTTGTTCCATTTCATCATTAGCTAATTGAGCTGCTTTTCCAAACCCAACAGCTAAAAATGTTGGTGTTGTTCCACTTCGTAACCCCCGTTCTTGGTCTCCACCATGTAATTGTTTTATTAACCGAACTCGTGGATTTCGACGAACATATAATGCACCAATTCCTTTTGGACCATATATTTTATGTCCAGATATACTCATCATCCCAATATTTGCTTTTTTGACATCGATTGGAATTTTTCCAAATCCTTGAGCTGCATCCGTGTGAAAAATAATTCCATGTTCTTGGCAAATTTTACCAATTTCCTCAATCGGTTGTTTTACTCCAATTTCATTATTAACAGCCATTATGGAAACAAGTTTTGTATTTGGTTTAATTGCTTTTTGCAATTCTTTTAAACATATTTTACCATTTTTATTCACAGGAAGATATGTAACTTCAACACCCTTGTCTTCTAGATATCTACATGTTTCTAAAACTGCCTTATGTTCAGTCTGCTGAGTAATAATATGGAGTTTATTATTATAATATTCAGCTACACCCTTTAATGCCAAATTATTGGCTTCTGTGGCACCACTTGTAAATATAATATCTTTAGAATCTGCTCCAATAATATTTGCAATAATTTTCCGACTTTCATTCATAGCCTTTTCCGCATTCCATCCAAATGAATGAGTTCTACTATGTGGATTTCCAAACTCTGTTGTCATGAAAGGTATCATAGCGTTAATCACTCGTGGGTCTATAGGAGTCGTTGCTTGTGAATCAAAATAATTTGAAGGAATAGTGTAGTTTATTTCTGATGGTATTTTTAAAATTTTTTCTAACATGGGATAGTATTTATTATTTTTTTATTCATTTTTCATACCTAGTCTATGGAAACAGAAATAGCCTTAGAAAAATTTATTATTGAAAATATTTATGATCATAATAGTATAACTGTTATAACTGGAAAATACGAAAATAATCTTTTTGTATATCTGATTCTTAATATTAGTATATTAGAATTGCTTTCAGATGATTTTATTATTAATGCTATTAAAGAGAATAAACAAGAAATTGTGTCTACAGTTAATTGCAAAACAATTTTAATTTATCCAGCTACTGAAATGCATGTTCGAAAGTATACTATGACGAAAGTATATCGATGCGAACTATTTTCAGAGTATCTAAATGATGAGCCATTTGCACCAACTTCATGGATACAGACCGTTTTTGATCATGCAAATGGTCTAAGTGTACCTATTCTAGGAGAAGAAATATACTATTTCGATAATGATATAGTAATTATAGCAGATAACAAGTGGAATCGAAATGTCAATGAGCTATATTTATTATGTCTATTTAAGAATTCTAAATATAATTCAATAAGAAATCTAAATGAAAATGATTTAGAGTTGTTAATAACAGTCAAGAACAAAATCTATAAAGTGGTATATGATAAATTTGATTTAACATCAGATAATCTATGTCTTTATTTCCATTATCGACCAACATACTACCAATTGCATATTCACATAGTTAATATTTCTAAAACAACATTGTGCCTTTATAATTGTTACCGCAGCATTCTATTGGATGATGTTATTAAAAACATAACATTGGATGGAAATTATTATAAAAAAGAAATATGGTCATTATACTGAAATGACTATTTATTAATAGGTTAAATTACTAGTGTGGTTAAGCATATGATTAAATTCGGATATTGAAATGTAACTTGAGTTTTCATGAAATTCGACATTATTGTCTTTAGAAGAAAAATATTTGTCATTATTAAGAATTTTTCGAGCATTTTTAAATTCATCTTCTTTAAATAAGTTATTAGAAAATAATATTGTACTTAGGGAATATAGCTGAAGAGAAATAGCGATAACTTTTATAAAACTTGCTATTTCAGTAAGTATAGTATTATATACATTAATATCAATGGATGCTAATTTTAAAAACATAATTCTATATCCGATTTCTAATACCATACTACAAATAAGAAATTTTAAAGATAATATCCAGGATGCAAATGTGTTATCTATTATAAAAGCATCTACTAATATTTTATCTATTATTTGATCTTCAAAGCAGGAAATCAAATACATTAGATGTAATGCAATAATAACAATACTCAATGATTCTCCAATAAAAAATGCTTCGCTTAGCATTGCAAATATTGCATAATCATTATTAACTGTTTCAAAAATAAAACCCCCAACAACAAATCGAAGAAGAGAATACAATAAAATACTTTTTTCTTTATTCTTATGTTCTATAAAATCAATTGTCATCACATAACTCATTTCATTGAAAAATACTTTTAATGCAAAAAATAAGCATGATTTGAATATAACTTCTAATATGAAATGGAATTTTTTTAATATTAAAAAATCAGTGTTAGAATCAAAATACCATAATTTTCCTTGATAGGATCTTATTAAACTAAAATACAGACATATCAATTGAATTAGCATATTTAGAATAACTAGTAGTTGAACAATTTTGACTGATCTTTTTGTATTTTTTTGAATTTTGAATTTACAACTGTAATAAATTACATTCAGCAGGACAATAATAATGGTTAGTAGAACAGAAATTGGTCCTATACGAAGAATCATTAAGTAAGTATATAGTTAAATTCTACGATAGTTTTTAAAACACAATGTTTTATATCAATAAATATGGAATAAGTAATGATAAGCCTGATCTCTTTTTTAATTTAAAAATTTTTATTTTTAAGCCCTTATGGCGAGATACTATTCAGAATCTTTTGTTTAGTTAGTCATCTGTATTTGCCTACAGCAAATGCAACAACCAAAGCTGGAATGAAGATTAAAGTCCTTAAGTGAGATACGTCAATGATCTTTAGGACGATTCCGACCAGTTTCTTTTAATAAATTTTTTATAAATTGGTGTAAAATTTGAAGTAAAAATATGTTGGTAATAAAATTTAATCAATTGAAGATGCTTGAGACGCTATTTTTGGCCAAAGCTCAGCGACTTCTCTTGGGATTGGACCAGAGATTTGAGTACCTTTCAATTCTCCTTTCTGATCCACTAAAACACAAGCGTTATCCTCGAAGCAGATATTCACTCCATCTTTTCTTTTCCATATTTTTTTTTGTCGTACAACAATAGCTAGAACAACTTTCTTTCGTAACTCGGGTTTTCCTTTTTTTACGGAAACAACGACGACATCACCAGGCGCCGCTTTAGGGATTGTGTTTAACCGGCATTGTAAGTTTTTAACACCTATAATTTTGACTTCTTTGGCTCCTGAGTTGTCAACAACCCGAAGACGAGTTTCCGTTTGAACTCCTCGTGTGATATCCATTCTTGGCTTTTTTGGTAATCGATCTCTTTTTAACGGTGCTGCAACTTTAACTTCTTTTGCCATTCTAGGGTAAAAATTTGAGAAAATATAATATTACTATTGTGTGGATTTATTTACTTTTAGATTTAGGAACATTAACTTTAAAATGTAAAATATCTCCATCTTCAACTATGTATTCCTTTCCTTTTGTCATAACTTTTCCTTTTTCTCGTAATTTGACTTCGGACAAATTTTCTTCAAAATCTTTATATTTCATCACTTCACAGTTTACAAAATATTTTTCAAAATCGCTGTGAATTGCACCAGCAGCTTCAGGCGCTTTCATATTTTTTCGGATAGTCCAGCTTTTACACTCGTCTTTGCCAACAGTGAAATAATTTATTAAATCTAGAGAATTAAATCCATAAGTAATTAATTTATGAATAACATCTTCAGTAATATATTTACTAGAAAGAACAATAAGTTCATTTTTATACAATTCCATTAATATTTTAAGATGACGATTACCTTTTTTTTCACGAAAATGTCTTTCAGAAATATTTGCCACTATTGTCAATGTCTTAGTAGTCAGCAAATTAAGAGTATTTATGAATGCTACCTCATCTTTAGTCCACATTCCATCACGAACCCATTGGGTTTCTAAAATGTGAAGCATTTTATCACATGTTGTAATTTGTTGAAGAATTTTCTTATTGTGTGGATCAGATCTGATAGATTTTTCGATTTTTTGTTTATTTTTCTGTACATGTTCTATGTCTTTAAGGCGCAATTCACTTTTAATAATGTCAATGTCTCGAAGAGGATCAACAGCACCCTCTACATGAGTAATCTCTGCATCTTCGAAACATCTTGTAACCAAAAATATCCCGTCTACTCTTCGAATGTGTTCTAAAAATTGATTTCCAAGTCCAACACCATCAGATGATCCTTTTACAAGACCTGCGATATCAATAAGTGTAAGTGTAGCTGGAATTGTCCTTTTTGGTTTATATATCTCACTCAAAAATACAAGACGTTTATCATCAACTTTTAATACACCGACATGTGGATCTTTAGTACAAAATGCAAAATTTTCAGCTCGCACTTGAGAATTTGTTAATGCATTGAATAATGTGCTTTTCCCAACATTTGGTAATCCAACCAGTCCAATTGAAAGTCCACTTAATTTATTTGGTCTTGCAAATAATTTGACTTTCTCTGCAGTATTTTGAGAAGTAGTATTCATATGGGAATAAAAATATAAAAATTGTGATGCCCATTATGAAAACGCTTATTCCTGATCAACAAGAAGTTTTCCTTGTAAAAGTTCCAAAATATTTAGGTAAACAAATGCGAGCAGTTGGTAACAATAAGACCATTGGTGAGTTTCTATTTGATGGTCAGATAGCACAAATTAAATTTCCAACTACTATCTATCCGAACAAATATGAAATAATAGTTAAAGATGATGATAATAAATATATTGCAAATGTTGGTATAGATAATAATAATAATGAATGTTCTATATATAAAATATCAAGTATGCTTCATGTTGTGCCTGTTATGGATGCAGCGTATTTTGAATTTAAGAAAAAACAAAAAGAGCAAATTCATGATAATAAACATGAAATTCAGTTTTTAAATCATTTTGATGAACTTCGTAAGGGTGAAAAGTATGCAAATCTGAAAGAAATGGAGATGTATATAAAAAAAAAGAAACAGCAAACACAAGAAAAGAAACGCGAACGATTGGAAAAGCCTGATGTTCTAAATTTAATATTTAAAGCATTCGAAAAACATGATTCATGGACCGTTAAAGATCTTGCTGATTTTAGTGGCCAACCAATTGCTTATATACAAGAAATTATTAATGAAATTGCCATATTAGACAAAAAAGATAATAGAAATACATATGTGTTGAAAGATCAATTTAAATATAGATAAATATGAATAAATAATATTGTATTATGATAACAGTAAATATTATCTTTGTTAAAGCCCCTATGGTAGATAGCTCTTCCAATAATAAGTGGTTTACTACTTTTGATCATCCCAGTAGTGAAAATCTAAAAAGACCCCAAGCTTCAGAGAATGCAAATAGAGATTTGTCTATACATCCATATCCTAAATTAAAAATAATGAAGAAAAGTAATAAAATTTTTTACAAATTGTATGCTAGCATCAAATCACATAATGGACCTATTGAACGTATTTTAGTTGATTACTCTGTTAATAAAACATTCGCGATATCTGGAGGGGTTGACGGATTGGTTAAAATATTTGACATTGACAACCAAGTGTTGTTTCAAACATTGTATGCTCACAAAGCTAGCGTAGTTGATATTTGTCTTCATCCTAATGGAAAATATATTGCTTCTGTAGATACTTCTGGAATTCTAGTTTTACACAAATTAGATTCAATTAGTAAATATTTTATGTTAGATAAGTTTTTTGAAATCAATGAAGAAATAATGTTTTGTGAATTTTCTAAGGCTCAAGGTCATAATGAAAATATAATGTCTACTATATACACTATTCCGACAGAAAATGATGGTGATTATGATTTAATTGTATTAACTGTAAATGGGATTTTAATTCATATTGATATTATAAATAAAATAATTATTGGAAAAAATACATGTCTAGCTAACAACTATATAAAGGCCATTTGCATTACAGATGGAGGTCGATATGTGTTTTGTGGCGGAGATTGGCCATACTTGTTAGGATTTGATATTACACACGATGATACAATACTATATTTTGAAGACTTCAGCACTAAAATTAAGACAAATTTCATATCAATTACAAACATAACTGCAGCTAAAAACACGTTTAAAGTAGCAGCATCATGTATGAACATTGTTTATATATATACAAGCCTAAATCATCGAATTAAAAAATATTCAATCGATATTTCAGAATATAAGACAATCACAAGCAATACAACAGTTCTTGTACATCGTATGTCATTTTTAGTTTCAAATGTATTATTAATTCTTGGAACTGACAAAATACTACGAGTTTATGAAAATGATATGTTAATAACTATTAAAAACAATGTCGAACTTGGTGCATTTTATACGCATGGATCTAAAGATGTATTTGTTATTGCAGATGAGGAAAAGTTACGATTTTATGAATATTCAAATCAACAAATCATTGAATTGTGCACAATAAAAACACCAGTTTCTGTAAATGATTGTGCATTTAGTAACAATGGAGAATACTTTATTACAACTGATGATACAGGATCTATACATACATATAAATTAAACTATATGCAAAAAGACATATATAATACCATTCAGTACTTTCATTATGATTTAAAAAGTTCAAATACTGAGGATTATGCTAAGTTAGTTGATTTTTTATTTAAAATCAATAATAATTCTAAAAATGATATTCAATCATTGTTTTGGAGTATTTATGGATTTCACAATGAATTTTTTCAGTATATGTATACTAAAAAACTAAATATAAATGATATCAACATTGATACAATAAAATTGCTTGTAATAGATTTTCTATATTTAAATTCTACGTATCCTTTTTGTGAAATTAAATATGTACCAAAAGCTAAATGTCAAAATTATGCATGGATGAGTGAAAATGCTATATCACGATATTTAAATAAATTACGAATTACCTCTTCTAGTATTTCAAAAATGGATTATATGTATGAAAATGATATTGCTGAATCTTCTAATAGTATAAATACACAAACAGTGTCAGATAATGAGGCTATACCAGAAGCATCTTTAAATATACAATGGCCATCAAAAAGCGCTCGTTGTATAATAGATTCAGACAGTGATGATGTAGTTAATACTATGGCAACAAATTCTTCTCAAAAGCATAATATTTCTCGTAAAATAATGCTTCGACGACAACAAGCTATATCTGAAAAATTCAATTCTACAGAAGAACATAAAATATTAACACGAAGACAACAAACAAGACTTATATCAAAATTAGAATCCAATTCTACAGATAATTCTAACAGTACATTAGCAGAAATATCCATTAGTACAGAAAACAGTTTGATACTTAGTGACAGTCATGAAAAATAATCTGCTATTTATCTAATTTTATATTTTTAATACCTCTATGATGGAAACATTAGAAAAAATAACAGAAGAAGATTTTGAAATGTTTGAGATGGAAATGAACAGAGAAATTGAAAATTACATAAACGAATTTAATGAATTTAAATATACAATCAATAGAACAATTATACTACTTTCTCAGTTAAAACAAAAATTTAAATAATATTACAAATATTGTTCTATATACTTTTTTATACGAGTTTCAATAGGTATATTATTCATTTTAGAGAAGTAGAAAGTATTTAGATAATTATATATTTTTCTTAAACGTTGTATTGAATGATTGGCTATAATTCCAGATAAGATGAAAAAAAAACTTTCCAAAACATAAGCTGTTAACTCTGATGGTTGAGTATTAAGTATTGATGCTAAAAAAAACCATGTATCTGAAAAATTGTTTCTATAATACAATGCACCAAAATATATAGCATACACGCCTTTGATTTCAGACATAGAAACTTTTTTATATATTGCAAAACGAATATACTGTTCCATGTATGATTCCGATAGTGATGACAAAATAAATCCAAGAGGCTTATAAAAATCTATACAACTAGAAACTTGAACTTTCCCTTGATCTAATAATTTAAGTAAAAATAAATTTTTAAAAATTAAGTTATTTTCATACTGCTTTAATTCATTGACCAATTTTTGGGTATGCTCAGGATCATTAGAAATTTGATTGCATTTTTTATTAACAAATAATTTTAACTTTAATAATTCATTTTTATCCTGCTGCAAAATATTATCTGGATGATATGGAGAGTATTTATCCGTAAATTTATATGCAAATGGAATATTTGTTGTTTTTGGTTGGAGTTCGGAGATTAATTTTTCTAATTCTTCTTTCTGCTTTATATATTCTCTTAGTAATTTTTCTGAATCATTGTCAGCCATAATAGATGGGTATAAATAAAATTTCAGTTATAATATTATTGTAACATATAATTCTTCGGTATTTCAGTATAATGAATATAATTTATATTAAACGTTAATGTTTTAAGATCAAATTTGATTAAATGACCTGAATCAGTACCGAGTAGTAAATTGATGCCATCAAGACTTTTTAAAAAATGACAGCAACTAAATAAAGGTTCATTACATGAAGTTACAAATTCAAATATAGTAATTGGATTTCGTACTTCTATATTCATCTTTTTTAGTTTATGACATTCAACAAAATAAATATCTTTTTCCATCTTTAGTAATGAGTGAACAATTGTACTACTTGTATGTAAAACAGTGATTCCGACTTTGTAATAAGAGTTAAATTCACTACTACTATGTAAAATATTATTTCTAGGAAGAACTTTATGTGACAAATAATATTGAGCGACAACACCATTTGATAATGAAACTATTCCGTTATTGAAATCATGGAGTTTATATTTGAATTTAAATATTTTGTTAATGAACATCATTTTTTGAGCATTTATGTAAACATAATATATGTATCCTACATCTGTACCAATAACAAAAATATTATCTGCTTGATGATAATCAGAATGTTCAATTTTTATAATTTTACCTATTATTTTTATCTCATCCACAACTTCATTATTTTGAACAAAATACAAAAATCCTTTTAATGTTCCAATTAAATTGTAGGCAATATATGTTATATTTTCAATTTCATCTACTTTTAAAATCCCAGTTATGTGTTCTTTAGGTCGAAAATAATGATCAATTGTTTGAATAGATATTTTATCATATTCAAAATAAATTTTATCATGATTATTATAATCTTTAAATGAATCTTTTACAAAATACACGCTAACATCACTTTCAAAAACAATATTTTCATCTTTTTTATATACATGACGAATCATATCAAGATCGTTTCTATATGATAAATTAAAAAAGTGATTCTTGTAAAAAATTCCATTATGTTTTTTTGTTATAACATAATCTGTTATAATTATTATACTTGGCATTTTAACAATCTTATAAATGTTATCGTTTTTCATATATATGGCAATAGAATTTCCAGCATAAATATCAATTAAATTACCATCAATATGATATGAAAAATGCATGTATACTTTCATCATCTCACTAATATTATAATCTGTTTTGTATTTATCTATCTTTAATAAATAATTTTTTTTCTTTTTTGCATCTAAAAATACATATTGTTTAATATATGTTCTATATAGTGGAGTTATTAGAGATGGTGGTGAAAATTTCTTGGTAAGTATATTACCATCGGTAAGTAAAACAATTTCATCATTAAATACAGTTCCATTAGTATATGTCATTGTGTCTTTATAAACTTGCCGGCCGTTTTGAAAACATTTGAGTTTTCCATCTCGGCATAAAGCCCACACAATGCGGAAATTATCCTTAATGACCATCAGTTTATAAAAAAATTGTCTATAGTCTATTCCAACAGTTATTGTATTGAAACTGTTATCATGAATATTCATTCGGCTCATAAATATTTTTCGATCTTCTGCGCAAGTGTATATAACAATATCATTTAAGTCCTCTATTACTTTAATATCCATAATTCGTCCTTGATGCAAAATAATATCTACTGTAGTTTTAGATATCATATCTAATATTTTTAACGTACCATATGATCCAATAAATATTAATTGAAATTTTTCATTCATAAAGAATTCTGCACTGAATGCTGAATTCAGATTAAAATCCCATTTTTTATTCCACTTTGTAAAATATGTAAACACCATTCCATTATTAAATATAATATAAAGATGATTATTAAATATTATTGTTTTAAGAATGTAATAATTGTAATCAAAATAAAAAGTATTCAAAAATACATTATACTGAGGATTGTATAATAAAATAGAATGTGAAGTTACAATTGTCAATAAATTTTTAAAATTATAAAGATTTGTACAATTTGTTTCTAACTTCAAATATTCTTCGACTGTTGTATATCTTTTTTGAAATATTTTCCTACCGTTCGTATAAAATTCTACATCATTTATTTGCATATAGAAACTCATTTGTAGGGAAATTTTTTATCCTGATTTATGGTATGTGAGTTTTATGATCCAGTGTTTCAGAAATTAATTTTATCTAAATTATCAAAAGTGAATATTGCCAATTCCAAAGAAATATTTAATTATGGGATTGCGGTACTTAATACTTTACGTAAAGAAATTATTAAAAATATAACATATATAGACAACCTCATATCTTTTGCTTGCCAGATTATAAGGTTAATCAACAATTTTAATAAAGATCTCATGGATAAATATATTATTAATTTGTTAAATATAAAATATTCAAAAGAGCTAGAAAACTGCATTTCAACATATACTATTAATATTAGCACTGCCCTTGTAATAATTAAATATCGACAAAAGTTAATATCATTAGAAGATGTTTTGGAATTTATTCATATTCATAAGAATATAATAAAAGCCAGTAGTAAGTTAAAAACAAATGATAAAAGAATTATTAAAAAAGATTTGCTAACAAGTCTCCGAAACATTTATTTTGAAGCTAAAAATCAAATTAAAGAAAAAAATGCAATTATTATCAATACAGAATTTAAAAATTATACTAACTGTATTCAAAATACAATACAGGATTATTATATGCATGGTAATACTATCCTGACTATTTCTGAAGTAGAGATTATTCGATTAAAAAATAACGTATGGAATGCTTTGCATGTATATGATGAAACGGTATTGATAAAATTAATCTGTTACGAATTTGTTATTAACTACTCCAATCCCCGATTAATTCCAATTGTTATTATAAATAAAATTTTAAATCTATTTTACAATGGATTGCAATTTTTGAAGACTGGTAATATATTTGAAGGGGTAAGCTGTTTTAATGAAATATATAAAAAAGGTTATCTTCGAATATTACTAAGTGATTTACTTAGCTTATGTTATTTTTATGATTTAAAATATCTTGAGAGTCAATATTTTGCTATAGAGCTAATGAAATGTGGAGGATTTAAAGAAGGAATATTCATTTTATGTTTCTTGAAACAATTAATAAAAGAAATTAGTATTTATAGTCCTCTTTTACTTTGTATCGATTATATTACATCAGCTACAATCAAATTCCCCTTATGGAACCATTTGAGCTATACTGCTAATACACCCAAAGATGTTTTTCAAAAATATACAGGAATGGACTTTAGTTTTGTTAAACAACATATTTTTCAAAAACACACCGTATTACATTTCTATACATACAATAATAAAATATATATGATAAATTATTTTACATTAGAAACAATATTAATTCACGATAATTTTTCATATTTGTTAATGGTATTTGAAGAATTATTAATTGAAAATAAAAATAATATGGACAAAAATATATGGGCAAATAAATGCCATGTATTAAATGAAAAAATAGCCAAATTAATGGCTTCTATCAAAATTTGTAATTTAACGGCCTTAAGATTATTTATTATCTGTGAAGGAAGTACTTGTAAATTGCCTTTAGAAACTATTATTTATGATTATAATGAATCTGTTTTAGAAATTTATCGGTTAATTGATATAAAAACACTCGTTGGAATGAAAGTTTTGTATCTATCCACAGAAGATCTTAAACAGACATACTATTTAATTAATCCGGAAGGAACACTACAAGCAATGGAAGATAATATTTATACACTATTAAATAAAGTTGGAATTAACCATGGAACAATTGGTAGAGGCTTGTTAAAAGATGACAACAATTATATGAATAGGCATGTGTGTTTTATGTATTTTGGCCATGGAACTGGAAAAAAATATTATCAGATTCATAAAGATACAACACTAAAATGGATCTTCCTTTTTGGATGTTCATCTGTAAAAATGACAACATATTTTCCTATTGATATGCATAATATATCTTCAATACAAACCATTGTAAATAGTATTCAGCTTTCATCAATAATATCCTTCAAAAACAATGGATACATTTCTATGATCATTAAAAACAAAATAATTGTGGGATGTCTTTGGGATATTACAAATACAGATTTAAATTACTTTACAATGAAATTTTTAGAGAATCTTAAAACAAATAAGGTTTCCCAATATAACTTTAAATACTTTAAATCAAAGTGTAAATTAAAATGGCTCAACATGAGTGCAATAGTGATATACATAGGGTAGTAACTATTTTTAAATAAAGTATATATAATTTAGATAAACAAAAATACATAAAAAAATGTATTTTTATTTATTTATAAGCAGTATTTGAAGGTGATATAAATTTTAAAAATTCCTTTCCTGATTCACAGTTTTCATATCCATTTCGAATAATAAATGATGCAATATATTTTAAATAAATATCTAATTTAGTTTTTCTATCTTCCATTACTGTACGTTTTTTATTCCAAAATACTTTAGATGGAAAAGGTGGAAGATACATAATTTCTTTTTCAAGAATTTTGTGAAGTCTTTCAAAATCTGAATATCGTCTGTATACAAAATAACAATCATTTCCGAATTTAGGAATTTGTGCAACAATTACAATCTGATATTCAGTGTATCGTTTGGTGTTTTGGTACCTCGGTATAGATATTTCAAATAACTGCTTCTTATACGTTTCCATTTTGTTATAATATTTACATTTGTTTTAAATATAATAATAATAAAAATATTAAACAAACAATGCCTTTACATATTTAAAGGATTATGTATAATCCGTGATAATATTCATTTATACTAATATAGTATTTGTTTTTTTTTTTAATTTTTACCCATGGCAATTCCCAAAGAACTAAGAATACTTACGGCTACACAATTAGAAGCCCAACTGAAAGATGCACAGAATGAGCTTCAAAGACTTTATCAATTACGACATTCAAAACAAGTTCAACCAGAAGAGATTCGGGAAGTCAAAAAAGATATTGCACGAATCAGAACAATTCAATATGAGAAACAATTAGCTGAATTATGTGCTGAATTCAAAGGAAAGAAGTTTATTCCTAAAAAACTCCGATTTAAAGGAACAAGAGCGCTTCGCAGAAGTTTAACGCCTAAACAACGAAATTTAAAAAGCAAAAATCAACGTATTCTTAATAAGAAATATCCACAACAAATTTATACATTTATTGATCAATAAATAGTATGAATCATTAACAATGCATATTCTTATTAATTAAATAATTTTTTTAAAATTTTTTATAATTTGTATGCAAACATTTATGGACGAAAGTTTAGTTACTGTTTATTTTTCGGCAGAGGCTAATAGCATTGTGTTTAAAGAACCCATTAACCATTTAATTATGATAACATCTATATGTGATATATGCAATAAGCCTAAATATAAAAGTTGGTTTCTCGTTGCAGATACAATTCCATATAAGATAATGTCTGTAACTGTATGCGATGGGTGTGGACCAGGAAAACATACATCTGCAGACCTTTGTGAGCTTGAATATGGAATACATATTGATTTTAGATCTACAAGTTTAGATAACTTTAGTTATAGGATGAATTTTATTAATACAGAATCTCTAGTTAAAGTTTTTCGTAATAGAGAGTTTTTGATGGAATTTAAATGTGAGAATGCCTATGTTGATTCTATGAGCACTCTTATTGAACGAATGATTGATTTGATAGATACTATGATACACAGTGGAACGTCACCTGCTCTTATAAATACAAGAAATAAATTAGAGCAAATTATAAATGATGGAATAGTAGAATTAGAAATCGTTGATATGAGTGGATATAGTCGTATATGTACACAGCATATAGAATATACTTCAATTCAATCCACTCCTATTGAGGAATTAGAGTCTGATTCTTGTATTATTTATAAAAAATTTGCTCGATCAAAACAAGGACTTGATCTTCCAAAGATAGTAGTTGACAAGCTTAAAAAATTTGGAATTCATTGAATTTTAATACCCCTATGAGAGGAACACACGGGATTTTAAAAACTGTTAAAGAGAATAAGGTTGCAATTAGAAATTTGTATATTCGGCTTTTGGTAGCAAATTTGTTCATTGGAATTTTATTCTATCTAAGGAATAAACACGTTCTATCTTTTATTAAATGGAGTCTTGTTGAGATATTGTTTTTGGGATTACTTCATATTAATATTAAGCCTGTTATAATTGAAGAAAAAGAGAATATATACAAGGTAATATCGTGTCGTAGCATTGAAGAATCGGGACTACCATCAGCATTGGTTGATTCGCTTATAATTACTACTTTTGCCAAAGGAATTTCTATTTTCAATTTCCCAATGTTACCAATAATAACATTCTGTTTTATTTTAATAATATTTGTATATGAATGTATATACAAGACATATAAACAAATAGTTCAGAAGTAGAAGTAATGTTATTGAGATGTAAATATGAATATTTTCTGAATGTAAAACTATAATATTTGTAAAACAATGAGTTTTAAATGTATAAACTATAGATTATTATGGATGATGTGGTTAAGCTAATATGTGCTTCAAGCATCTTTATTCTGTTCTACTCAATGGTATCCAAATATATCCGTGATATGTTGTATATTCCAGATCCTTTTATTACATTGTTGTTTGGAATTGCTATTGGACATCATGGTTTAAATGTACTGAATACACACTATGTTTACTCAAAAATTATTGTCCTAAATTTCAGTAGAATAGTTTTATGTCTTCAGACTATGGCTATTAGTTTAAAAATTAAAAAAGAATATCTAAAAACATCATCGATGGTTCTTTTTAACCTTGTTGTGCTTGGGGGATTCATTAAATGTTTTATTACATTTATTTTGATATTTGGAAGTACATATTTAGATGTTCCAACCAGCTGGGCATTAGCTGCATCATTAACACCTACAGATCCCATTCTATCTTCATCTATAGTTTCTGGTGAATTCGCAAAACAAAATGTAGCAACTAAGCTAAAAAATATTCTAACAACCGAATCTGGAATCAATGATGGATTGGGAATTATTCTAGTAAATATTAGTATATTACTTTATAGTGCTGCAAACCAAACGTTTGGAACTAAATTCAAGGAGTTCATTATTAATACGGTTTTAATTAAAGTGCTATTTAGTATTTTGTGTGGGATTGTTCTTGGGAAAATTGTAAACAGTTGCAATAAAGTATGTTTACGATTACAGCTATTGAATTCTAACACACTAATAATACAATCTTTTGCATTAACTTTTCTTATACTCTCGCTTATGGCAATGATAGATGGTTCCGAATTGATATGTATATTTTTTACAGGGATATTTTTGAATGAAGATAACTGGTATATTAGTCATCAGTCTAACTTTAGAATTTCGTACGTGATTGAAAATTCGTTTTCTATGGCATTATTTGTGTTTTTAGGATCTCGTATTGATTTTACACGATTTAACATGCAAATGTTTAAAATCATTATTAATGCAATTTTTATTCGTATTATTACTATAATTATGTTATACAGACAGTTTTTATTCGAAGTAATAGGGTCATTTAAAGAGGCATGTTTCGTTGGATATTTTGGTCCCGTAGGAGTTGGAGCAATCTATTATTCATTACTATATGATGCATTAGTAGATGCATTGACAATCGATTACGCTATGTGCTCTGTTTTTGTGAGTGTAATAATCCATGGATTGTCAGTCCCTACGTACTGTGCAATCAAATATATTCTTAGGTATATATAATGATAAAAATTGCATATTATCATTTATAGCTGTAAATTAATAATACTCCTTTTTGACTATAAATATTTTATCCTCTAGACTTGCTTCTATGGAAACTAAAGTAAATGTCACACCCAAAATATGTCTTCAACAAAATGAACTGACACAATCTAGCGATAAATATCCAAAAAGCTTGAATGATGATAAAGTAATAGAGATAAAAAACATGATTTATATAGTAAAAAAATTCACAGATAATGAGATGAGTAATACATGGAATGATGATGTTAATATGAGTCAAATAAGTCTTAATGAACTAGTATTAGATAAAGTTCCAAACATTATTTCAAATATCTACCATAAAGACAATGATACCAATATTAATTCATTTATACATTTTGACGATGTTCATGCTATTCAGGAAAATGGATGTATATACAAAATGTACTTTAATACAGCACTGCATACAACAAAAAAAATTTCTTTACTAAACACAGCGTTTGTTTTTAACAAAAATATTAAGGGGAAATCAGAGTCCTTAATATTTTTTTCCTCCGATTTTAAATATTGCTTTAAAACAATACGAAAAAATGAATTTGAAAAATTAGTAAAGGAACTACAAACAATACAAACATATTTCAAAAATAATCCGACTACATATTTGGTTGAATATGTAGGAATTTTTTCGTTAAATTATAATCCAGACATTTGCAACAATGAATACTTTGTAGTAATGAAAAATATCTTTCAATCTCCACACAGATTTGTGTATGATTTAAAAGGATTAAATGTAGTTAGAAAGCAACAAACATCAATGGGAATCAATTTAAATTTAAAGCTAACATTATCTCAAACACAAGTTATCCAATTGATTAAAGACATTGACTTTTTAAATTCTATTAATGTAATGGACTACTCTATTGTAGTGGGATGTGACACAACTGATAAAAGTTCGATAGATTCATTTGATATAGGAATAGTAGACACTCTTACTGAATATACATTTAATAAAAAGCTAGAAAATATCTACAACATATTATTTTATGAATCGTGTAAGTCTACAATCGATCCTCAGAAATATGCAAGACGTTGTAAAACCTTTATTGAAAGTCAACTTTCCAATATAGTCTCCGAAGACATGCAATAATAAATTAATAATTAGCTTATACTTTGTAAAAACTTGTATATATGTTTAGGATGTTATAACATAAATAGTTCTTCATCAACATCTAGCCTCTTTCTTGTTTTGATTATGCTTATTATCATTGCTTCGAAATTATTTCTTTGTGATAAAATAAGAAAAAAATTCACTATGAACATAACAAGTAATGTACTCAGTAATATCCCAAATATTATTGCCAGTTTATTCATGGGGTAAATCCAAACAGACCAAAATATTTATTGTTATTAATAAAATAAAATTTAAATAACTTTATGATTGATTTTGTATAACAGTCTGAATAAAAGTATCTTTTTCTCTAAATATTTCGTATAAGTAATTTTTGCTTTTCATTGAAGAATCAACTTTTTTTATATTTAGTCTTATAATTACATGAGATATCTTTGGCGTCACACAAATATCAAATAACGTTAGTGGAATATCAAGATAATGTATAGTGATATCTACGAGGTAAGTAAATGATATATGGTTGAATATTAATTCGTATCCTTTGTATCTAGGACATAAAACATGAATAAAAGGAGTGATATTATGAGTTTTACACCACTCAATAGAAATTGATTTTTCTTGAGTTGTACATCGTTTTCCCTCTGGAAAGATACATAAATAGTTAGTGTTTGATTCCTTACAAAACAATTCAATTGTTTGTTTGTCTTCAAAAAAACTTCTTTTCAATGGTAAGAATCCACTTAATTTAAACAGTCGTCCAAATATAGGAATATATTCAAGCTCATACTTATAAAAATATTTTATTTTAATACTTTTTTTAAAAATATAATTAATGAGTATAGGATCACAGTTAGTTATGTGATTTGCAATAATTAATGTTCGGCCGCAAGGTATAGATTTATGATTATCATAGTATATTACAACACTTTGGAAAAAAAGTTGTTCGAAAAACCAACCGATAATTCGATATAAATAAAAAGTAATGATAGTATTTACTAATTGCTTATATTTATTATTAATAAATAAAAGTACACTACAAAAAAATATTAATGGAATAGATCCTAAACACGTTAAAATTAAACTAAAAAGTAATATAAATTTATATATCATACGTATACAAACATTATGTATAATTGCGGATTTCATAAATGTTTTTTTATCACATACTTTTTCCATAGGGTAAAATTCACTTATTATTTAAAAATATTGAATAAGAGCATAATTAGTGTATTTCATTTTTAGAATTATTCCCGAACAAGCAATCATTAATATACAAAGTCCCCATAAAATTGCTATTTTTAGTCGAAGATTATCTACAAATACGTACATAAGAGTGTCTCTTAGAATAGGAAATAAAATACTGGATATAGGAAGTGTATAGTTATATTTTTCTAATAATCCAAAATTAATAATAATTAGTTCAATAGCAATATATAAATGAAATAAATATTGTAACATTTGATTTTCCCAATATAGTATTATTGAGGTATATAATAATTTAAAGCATGTAAAAAAAATACATGTTACAGAAAAACAAGAAAGAAATTGGAAAAATATTGTTGGAGTAGGTATAATTAGTAAAATAAATAAAAAATAACAAAATAAAAATGTAAACGAACTTAATAACTTTTCCAGAAATATAGCAGAAAATACTGTTTTAAGGCTATAGATTTCACAATGAATTTCTCTTTTAAGAACTAACTTATATCTATAAATAATAAGTTGTTTGCCAAAAAATATAAAATATAAAATTATCAAGTAAATTATAAAATTAACTGACGGCTTAATTTTTTCTTCAATAGATAACATTATAGTAGATGCTTTTTTTGTAGCAGAATACATCGTAATTAATTTTGTATAATATATTTTAAATTCTTGATAATAAAATATGAGAAGCAACCGATCTTTCTTTTTATTAAAAAAATACTCCCAAAATATTAATATTATTCCACAACTTAAAAACTGAATTGTACGTTTTAAAACAACTAAAATTCGTTCAAACCAATTTTTTCTGCTTAAAATTCTTAGTTGTATATTAATCAATGGTAATATATACAATTTACCCGTTGTATGATCCCAATTAATAGAATTAAAAATCGTAGGCATTAAATTCTTATTTTCATTTCTTTCCCATTGATCTATATAATACTTATGTTTATTAATACTTGTAATGACTGTTGAATAGTTTGAAAAATCCACAATTAATTCCTTTTCAATAGCATTTTCATTTTCTTTGAGATGATTACAATGGTTATAAAAATATTTGTAAGCTTCATAATATGATCCAGAAAAAACAATATGCCCTTGCTTAAAAACAAGAATATTAAGATGATTTAACAGATTTTTGCTCACAGACATTAATTCAGAATATGCTGGATTTTCTATTAAATCTATTTCATTATCTTTCATAATAAATGTAGATATTTCATTTATACTGTAAATTTTATTTTTAAATTTTATTTCATAATTATCAAATGTATTAGAGCTGTATAATATTGCTATTTCAAATGTTTTACAAAGTTTTTCAATGATTTTTCGCATTTCATTCCATTCGTTATGGGCATCATATATAAACGGAACATCAATTAAAATGAATCTATTTCTTAATAAAAGAGAAATAAATATGTCTACCCTTAGCTTTTCGAATGGGGATAGCCGTCCATACTTTTTTTTTATAATTTTGTTAAGATCTAAAAGTTCCATATATTTTTCAACTATAGTAATTGAGGCAGAATCTTCTTTTTTAATTTTGATTGTGAATGAATCAGAAACAGAGTTCTTTCTGAGTTTTTTAAATTGCTCATAATTTGTATAATTTAGTCTACTTGAATTGAGTTGAGAATATATTTCAGTATTTAATTCCATGTTTTCACGGTCATATGATTTCTGGGATATTGCATTGGAGGTATTAACATATGATTCTGAATTTTCGAAATAAAAGTTTTCATAATGGGTTTTAATTTCTTTTATACTCATGAAATTGTACATCATAAAATCTTTGTCAATATGACAAATAAGTTTTCTTGCCATTGCTTGTGACATAATATCACTGTTATATTTTACATCTCCATAGATTTGAAAACATTTTGGAAGTTGTAAAGCTAGTGATTTAAAAAATATAGAAACGATATTTTTTTCTGGAGAAAAAATACAATATGAATGACCAGTTTCAAGTCGGCATGTGACATGGTTAATGAATGTAATTTTGTGTCTGTCCATTACAGTTAAATTGTTGATTATAATTTCATGCTTCTCCTTATATGATGTCATTTAGGGGATTTTATCTTACAACAATTTTCAAATGTTATTTTTGATTACTCAAAAGATTTGTTGCCAATTATACCATTTGTATATGCCTTTTTTATACAGCCACTTACTATTTAGTCTCCTATTATTTATCTCCTATATGTAAATGTTATTTATCCACCCTTATGACAAAAACATTAGAAAGCATCGAAGAATCTATAAGATTGATGGATTCGATATACGATGCAAATTTTGGAGAATGGATTAGAAATGAACAAAATTGTAAGGTAGTTGGTCAGAATATTAAGAAATATATTGGCAAATATGATGATTCTGATTTTATAATGGTTATTAAATGGATCGTGAAAGAATGGACCTTAAAAGGAATTATGCAATTTACAAAAAAGTTTATTACAGATGATTTGTTTAAAAGTGATAAATCATTATTTTCTAATAAAATTAATATTTTAGCAGGAATGATCTATACATGGAATTCTTTATTTATATCTGAATTTTTACTGACTACATCTTCTTCATTTAATGTTGATGAGAAATGTGAATACTATTATTGTATACTAGATGGTTTCGATCAATCCAAGCTGTGTGAAATACTTTTACAAATGGAAACAAAAACAGATGAGGAAACAAAAGAAAAACTCATTGAAAAATTTAATAGAGATATTTATAATGTTAAAAAGAAACGTACTGGGAGTATCTTAGATGCCATGAATCTATTATAATAATATCATCAATATAATTTTATATTTATAAAAACCAAGACATACAACAATATATTTTATTAATTAAAATTTTAATAATAGAATAATATAGTCTTCCATGGTAAATAAACTGTTTCATTCTTAATTGTTTGAACATAAATGTTTATCATCGCAACATTCAGGGTGACCAATCTTTGCTTCTGTATCTGTCGTTTTAATTCCTTTGAGATCTGCAAGAAAGTCTTTAGGAAGTCCTTCCATAATATTTGCTTCATCAGCAATAGTTTCATCACCGGTTATAACAAAGCAATATGGTTGTTTTTTATTTTTATAGATAGATCTTATTTTCGGATTGTTTATAGTGTGAATATTTGTGCCACTTTTAAACGAGCATGACTTAAGAATAGTATTTGTGACCTCACCTTTAAATTCTTGATTAAGAATAGAATTTACGGTTATTTCGGTTGGCGCTTTTTCTTTAATGGGTTTTCGTTTTCCCCCAATAGTTTTCGTAGGTGCAAAGATCTGTTCTTTTCTTGATACCATATAAGGAGTAAATTTTGCACAATTCTTTTATATTGTTATGAAGTCTATTTATCGATGTGATGGTGATCTAATTGTGAAAAAAAGTCGTGATGAGTTTCTTAATATAATTAGGAAGTATGAAGCTGAAGGAAAGAACTATAAAGTACTACAAATCAGTGGAAAAAAATTAAATGTTAATAATTTGATATTTTATGAATCCTATGATGAATTGATACAAATATTATCAACACAAGATGTTACAATATTTATTTATTCACTTGATGATTTAGTTATTACACCCGATCTTGATATAGCAATTAAAAAATATACAGTTACTAATACATTAATTGTATATTTCAAATATTAGTATTTTACTGGTTTTTTTTATAGATATCTACCATATTTTCTCTAATAGTATTTACAATAGAATATATAAGATATGTAATTAATATAAACACACCGAACATAAATAACCATTCTGGAATGATATAGAATATAGTAGATACTATGTCTATTATAATAAAAATTAGCATGTTAATATAGCATATAAGAGTATTCCATATAGTTCCTATTTCCATTATTGATGAAAAAGGCTCGAAATATATTGAGTGCAAAGTAGATATATCTGTTAATAAATTATTAATTACATGAACTAAATGATTTCCATTTTGAATGTTTATGACTTTGTATGTTCTTTTCTGATATCTGATTATAAATGTGGGGAATGTATAGCTATTAAATTTACTACTTAAAAATATATTTGTATAGAAATTAATAATTTTAATATCCATATGTATATTAGATAAATAATTTCTAAAATAAGCACAATTTGGACAATGAAAATTAAGTCTATCATAATATAAATAGATGAAAAACAAATCATTTCTTTGCAGATCATCATAGGTTAACTTTTTAAACTTCTTTTTAAAATCTACAGTTTCACGTGATATTGATAAATTTGAAGATGGTATTAGGTTTGCACTTGTATTTTCACTACATAATAGAGTCAAAAAAAATTCTATGGAAAAAAGAAACATTGCAAGGGTAAATTTTATAAAGCATATTGTACTATTGAATTTATTATTAAGAAATACACCTCTATGCACATAAGTTTTGTGAAAATTTTACCCCTATATGGCTAAAGTAAATGGAGTTGAGTTGGAGACATTGTATACTAAACAAAAGTTAGAACCTGGAAAAGCATATAAATTTAAAATATGTTTGATAGGAGATGGTGGTGTAGGAAAAACTACATTTATTAACAAAGTATTAAATGGTGTTTTTGAAAGACGATACATTGCTACACAAGGGGCTGTTACTAAAGATATTGTCCTTTGTCTTGGAAATAACAGTTATGTTAACTATGAAGTATGGGACACTGCTGGTCAAGAAAAATATATTGGGCTTCGGGATGGGTATTATGTCGGTGCTGTTGCCGGATTTTTCTTTGTTGATGTTAATTCAAGAGAGACATTTGGATCACTTAGTAAGCATTTAAAAAGTTTTAAAAATGCATGCGGAGTTGAAAACCCAAAATTGATCATTTGTGCAAATAAATTTGATATTTGTAAAAAGCCAGCATTTATGAAGTTAATTCCAAATTATACTCGCGGGTTTGATGTCGAAGTAATACAGATATCTGCTAAAACAGGATTAAATTATGATAAACCATTTTTAAGTCTATCACGATATTTGTTTAATGATCAATCTATTACACTGTCTGCTGATATTTCTCTAGAGGCATTGCCAAATGATTATGATTTCTTAAATAACCCAGAAGACGCCCAACAATTTAACGAAGCAGCAGCTTCTGTTGCACACTTTAAGCCAGAAGAGTGAATGGACCAATAAAATTGTGAAATGAATAAATAATGGTACTATTTATAATTATATATGTATATTTTTAAATTGAGTGTAAGTAAAAGTAATTAAAGGTTTTTTATTATAAAATTTGCTATTAAATAAAATTTACTAGCAACCCCTATGCCGTTGTATGGGTTTGGTTCAAACATCATGGGTCAGTTAGATCAACCAGAAGATAAAATTTTTATTCATCAGCCTATAGAAATTCCATTTTTTCAAAGTAAAAAGATAGTAAAAATAGCATGTGGAAAAATGCATGCACTTGTATTATGTGAAAATAATGAGCTTTACTCATGGGGAGTGAATGATGATTATGCGCTTGGCAGAGAAGGTGTTGATGAAGAAGGAATTAAACAAGTTGTGGTTAATACCAATGATCAAATCATTGATATTTGTGCTGGTGCATCTTATTCTGCTTTCCTAACCCAACGAAGAGGATATGTATATGCATGTGGTACATTTAAGTCAACAAATGGAATTTTTGGTTTCAATACAAATAATAAGTTTGGAATTGGATTTCAACGTATTCCGAATGCAAAAAGTATCATTTCTATTTCGGGAGGAATGAATCATATTCTCATGCTTGATAAATTTCAAAATATATGGTCTATAGGGGCCAATGAATCATACCAGTTGGGTAGAAAACATCGAGTAAGAAGAGATAAATATATTTTAGTTCCAATGGTTGTGAGTAATCAGCGAAATCAAAAAGAAAACTATAAATTCATAAATATTTCTGCTGGTGGATATCATTCAGTTGGAATTAATGAATTAGGAGAAGCGTTTTTTTGGGGATCTAATTGCAATGGTCAATTAGGGAATGAGTCCCTTCATCCTACGGATCATAAATATAAAATAGGACTAAGTCATGTTGTTCAGGTTGAGTGTGGATACAATCATACACTCATGCTTACAAGAGATGGAACAGTGTATGGATGCGGTGAGAATAGTCAAAAACAACTTGGTACACATTTAGATTCTAACAATGAAAAAACTAAATTATTAGTTACCCCAACAAAATTAGGAACTGGATTTTGTAAAATCCGTTCTGGGGGTGATTTTATTATTTTACAACGTAATAACAATCTATATGCTATGGGTATCAATACTGAATGTGAGTGTGGTCTGCCAAATAATATAGAGGATGTCTCAGAGCTTACAAAAATTCAATTCAAATTTAACAAAATTATTGACTATCAATGTGGTGGTAACTTTACATTAGTCTATACAGAATAATCAAATAAATATATTTAATATAAAATTTATTATTTGTATAAAATTACTTGAAAGATAGTCATATTATTCACTGTCATTATCACTTGAATAAATAGTGAATGGACTTCTGTTCAACGGTATAGTTTTTTTTAACTCTGCAATATTAATTTTTTGTCTTCTCAGTTGTATATTTTCAGTAACTTGCTTTTTTATTGTAGTTTCTATAGGTGATTGTATATTAGTAGTAACTTTAGATTTATTAGTTATATTATTTTCATTACTTGGTAATGTTTCAGAAAATTTTTCAACATTTTTTTTTTCAATAATACCAGTAAATGGAACATAAGTTTTTTTGTATATTTCATCCATAAATGTATCTTCAGGAAAGTCAAGTTTATTTTTCTCAACTCTTTTGTCTATTATGCTTTTATATTTTTCATAATTTGCATATTTTTCACAAAATGGTTTATTATCAACTACACAGCCAGAATTTCCACCAACAAACATTCTTCTTAAATCTTCAATTGTCCAATCATTAATTATAATGCTCTTTACTATTGATTTATTGCTCCCTAAAGATCTGTGTTTCCCAGCACATTCATGACAAATAAATATCCCAAGCGAAACTGATGCCCACGATGGATCAGGACTTTTGCAATCTGCACACTTTTTATTTTGAAATTTTAGTAATAAATAATTGAAAAACTCTTGTTGTTTTTTTATGTTTGAAGTCATATGGGTTTAAAAAATACTATTATCCTTTTGAATAATTGAAAAATAATGTTTCAGATTTTCCATATTAAATTTATCATCAAATACTTCTATATTTTCTTCGATAAGAAATCCATTTTGAACTTTTCCTTCAACTAATATTGGTGTATGTCCGTATTTCATTTGATCTTTATATCTAATACAAATTTCACGTCCGTCTACATTTAGAATGGCTACCAAATTTTCAAATCTTATTAATGTACCAATAACACAAACATACATATTTTCATATTCTTCTAATGAATTAACTATCATCCAGGGGTAAACGAAAATTTGATTAATGACAAATTTGTTTTATCATGCCCTTATTATGTCAAAAAAGGTAGAATTAACAAACAACGAAATCATTGATATGTTTATTGAGTCAGGGATGAAAGAAGAAGAAATTAGGGATGCTATGCGCCAGTTAAAGGACCGATTTCCAGAAACCATACTTGAATATTTGAAAAAGATTAAGGAAAAGGCAGAAGCTGAAAGAACAGCTAAGGAAGAAGAAAGATTAAAAGCCGAAGAGAAACATAAGCAAATGATTAAACAACAAGCTGAATATAAAGAAACACAACTTAAGAGAATTAAAGAAAAAATAAAAGCAGCCCAAGATGAAAATAAGAAAAAAGAAGCTGAATTTGATGCAGAAACTCAAAAAGGATATGAGGATATTGATGTTCATGGATATTTTAAAGTTCGTATTTTCTTAGAAAGCGGAATAACTGAATTTTATGGATTTGAAAAGAATGCAACCGCTAATACACTATATCAGAGAGTGCAAACAGATCTCGGTATTCAAAATTGTGAACTGTCTATTTTCAGTACAACAACAATTATACAACATGATAGTACACCTTTGGAAAATATATTTAATTTTCCTGCAGTTATGTTATCTCTTTCTACCAAAGAAACTATAAAGAACTTAAATCATAAACGAATGATTCTAAAATCTACCCCAATAAAAGAAAAATCTGAATAATTATAGATTTAAATTGATTTTAATTTCTTGAAATTTTTTAATGTTTAAATAAATGTTTCAAAACCCATAATATATGATTCTTAAGTTAAAATCGTTGACAGGAAAAGATAAAATTATAGAAATTGATGGTTCTTGTACTATTCAGGAGTTAAAAGAAAAAATTGAAGAATATGAAATGATACCTCCTGAACAACAAAGACTTATCTCTGGTGGAAAGGTTCTTGTAGAGGGAAATAAAACATTAACACAATATAAAATTGGATCAAACAGTGTTATCCATTTTGTCTTGGCTTTAAGAGGTGGTAACAACTTTAAGTAAAAATTTATTTTATTTATTTAGTATATCTTTAATTTCGTTGTTCTCCAATTTCTTCGTGCCCTATTTCTATTATTCTTGCGTCTATTTTCTGGAAGCATACGTTTCCATGCTGGAATTGTACTGTTAGAAACAAATGCTTTTGTCAGTCTCTTTTTTATTAAATATGATTTTCTCGATCCCATATGTACGGTTAAAATTTTAAAAAAATTAAATACTAATTTTTGGATAAATATAAATATATAAGTTTAATTCTTCAGGGGATATTTCATTTCTATTGTTTGATTAGTTTCTTTTAAAACATTATCAATTAAAGTGTCAATATCATACTCGATTTTTTTTTCAGTTTCTGATGAATTGTAGAAGCGATATAAATAATAAATAATGAGTATTCCTAACGTAATTTGAAGTTTATTACTCCATTTGGATTCATTTTTAATTCCTAAAGCATCATACGCCAATTCTAAATATAAGGTCATATCCGGAGCAAAAATTCCCGTTAAGATTATTGCAAAACATAAACATTCAAAAATTGCACAGAACTTGAATCCTAAAGATAATATTATATTATTAACCCGAGTATTTTTGATAACATATTTAATAAATAATATATTAATTTCAGCTACAAGCAATATTACTAAAAGAGATAAAATCCATCCTCCAGTAAATGCATATAAATCAAATATTAATCTTAACCCTAAGGAAATTGTGAATGCACTTACAAATAGTATTGTGTGACCAAAAGATGCAATTAATAAGTAAGATAATATATATAGACAAAACATGACAATAATGAATATATTCATTTCATCTTTATAAATAGTGGATATGTGAGAATATGCCAGTTCACTAGCATATTGTATATATTTATTATGTTTCATTATAGTATTAACATTGTTAACAACAGTATTTAAAAATATATTACTTGGATAAAAACACATTGATTTCGATCTAAATGATGCAAGTAATATTCCTGTCATTCCATAATTATAGTTGCATATCCTAATTTTTGAGTTTAGACCATAATAATAAATAAAATTACATATGGAAACAAATCCAGAGTATAAAAGAAGGCTCATCCAAAACGTATACAAATAGTGATACCGCAATTTTCCATCATAATTGTGAACAATATACATAAATCCAATAATCAACATACTCCATAAAAGACTCAAATCGGGTTTAGTAGTAGAAAAATATTGATTTCTACCGCTGAGATTAATTAATGTGAATAGTATTGGAGAAAAAAATGACATGGTATACTTTGGAAGTATCCAAATTAGTATGATAATTAGCATTATCTTATGTAATAAAATATGGTTTTCTATTAAGTATTTTATCATATTATTAAGGAGAAGAAATTTTTATCCCCAAATATGAAAAATATTAGTTATAAAACAAAATTAGATGAAAGGGGTTATTTGTTTCCATTTGATACATTTCTGGAAAGAGAGTTAAAACTTAGTGAGGACGCACCTGTATATTGTCCAAACTTTCAAATAGGACAATGCCGTGGAAATTGTAATCTTTTACATATTAAATTAGCATCTGCCGTAGTGTGTAAGCATTGGTTGCGTGGTTTATGCAAAAAAAATGAAAAATGTGATTATTTACATGAATATATTTTAAAAAAAATGCCTGAATGTTTTTTCTTTAATGTATATGGCGTTTGTAATAATAATGAATGCATGTTTCTCCATGTTAAGCCAGATTCTAAAGTTCGGGAATGTGTTTGGTATACTCGAGGATTTTGTAGAAATGGTGCTCAATGTAAAAATAAACATATTCGAAAAAATCTTTGTTGGGATTATTTTAACGGATTTTGTCCAAAAGGTCCTGAATGTAAACTTGGGCATGCTAATTTTGATGTTGAATTTAAGGAAATCAATGAAACTGATATTTTGAACAATCAACGTCATTAATGAAAAAATAATAGTATAGAGTAATTTTTTTACCCCTAAGATATGGTACGCCATCGTTCTAAGAAAATCAATAAGTTAAATCAAATTGAAGATTGTTATTATTTTTTATATTCTACATGCAAACGAGGGAGCAATTGTGGATTTCGCCACAACTATCTTTCAAAACAGTGCAAAATCATATGCGAGAAATGGAATAGGACAGGAGAGTGTCGCGAAGAATGCCCCTTCCGACATTCCCGGTATCATCTAGATAAAAATCGTTCTGAAGAACAGTGCTGGTTTGAAATCAATGGTGGGTGTCAAAAAGAATTTTGTGAATTTAATCATACTGAAATAGGAAAGGATGATTGGAAAAATGGAAAAATCCATAATTTAGAAGATATTCAAAAATCTAAAAATGTAATTCATTCTGAAGTGGTTGTAGATCCAAATGAATTTGAACAAGAACGACGTAATATAAATTTAAAAAAGAAAATAGAATTTCAAACAAAAAGAGCTAGACGAATTATGGCAGCAAACTTAAAACGAGCCATGGAAAGTATGGATGAAAAAGATAAAGATGAGTTTCAAAGAGTATTACAAATTATTGAAAAGAAAAATTCTGCTCATCTACTAAGCAATACCAACAATGATAAGCTACGCTCAGATGGTATATCTTCTTCATCATTACCTACAAATGATTATGATGACGAATTAAAAGAGCTTGACAAATTATGTAATTAAATTGTTTATTATTATTTTTCTATTTTTATTACAAGTGATAAATACCCTTACATGGATTTATCACAACATGATAATGACAATATTATTAATCAGATTACATCTAAATTAATTACATGGATGAAGAACACAGAAGCTGAAAAACAAATTGAAATAGAGATTCAAAAGTTATTATCGCAAACAAAAAGCCATGATGAAATTGTGAATGAACTGTTAGTGATGTTTGATAATTTAGTGCCCAACGATATTAAACAACAGTTATATAGAGAAATAACAGAATATATTTAATTTAATTACAAACTTATTTTCTGTAAAGCAAATGAATGATTGCGATCTGATAGATCATTTATAAGTTGATAGTAAAAAATATTTTCTAATAATTTTTCCAAAAAGCACTGGATAGTATCATTATTACCAGTTAAATAAAAAAATAGTTTTTGATATTTTCGAGTTCGAAGAATAGTTCTACAAAATATGATTGTAGAATTAAAAATTAATGCAATATCTATTGAAATTCTAATGTCACAACTTCCTGTTCCATATGCTTTTGTTTCTGTTTTATTGCTTTTTTTGTACACTAACCTTACTTTAAGATTATCTAATACTACATATTTCAATAATTGTGTAGCTTCTCTTGAGACCGGTGATGATTTGAAAGTAATTTTTGATGGATCCTTTGTCATATTTTACACGCTCAGGGGTAAATATTACACATATTTTAAACAATATAAATGTCTAATCAACATCTATTTCTACTGGATCATCTTCAAGTTTGTCACTTAAATCTATAGCATTATTAGAACTACTTTTTATAAATTTAACTATAGAATATATATTTGCAGTTATTGTTAATTGAGAACTATATAATTTAATTAATTCATCAGCTTTATTAGTATCGATTTCTTCACCCGATAAAATACTTTGTGTAGTGGCAATTAAAATCTTGTTCATTTTGTTCATATCATCTATAATCTCTTCACTCTCCATTTTTTCTTCTGGTTTTTGTGATTCTTCAGATACTATAGAGGGAATTGAGCGTTTATCTTCTATAGTATTAATTTGTTGTAGTGCTATTTTTTTATTCCACCACAAAAATATTCCAATTAATATCAAGCACATAATTATTAAAATACTTATTAAACACCATAATATTCGTTTTTTATCTTTTTTTGATTTTTCTGATATAATGTTATTAACTGTCATTGTACTACTTAAATGACATTTTTCTTTTGAAATATCATGTATTGTTTCCTTATATGTATTATTTGAAACAGTAGTATTAATTGTTTTGTCTGTATTCATATGGTATAAAATTTGCATACTTTTCTTAATATAAATTATTTTAATATTTTGACTATTTTTTTGTGTAATTCATTTAACTCATAATCATTCTTTAAAATCATCAATATTTCTTTACTAAACAGACCATCAGGAATTTTATCTCCAGTCAATTTACTTCTTATTGCTGCATTTTCAAGCAATTTTAAATATATTCGGAAGACGTTTTTTAATAGTAATGGATTTTTGGTATTAATTCCATACATAATTAGTGGTGAAATAACCATGTTAATAGCATTAAATCGTTCAGTGGTTTGGATTGCGTATTCTAATCCTTTATATGTAAAAACTCCTTGTTCAACATTATCACAAATTTGCACAGAAATAATTAAAAATAGTAGGTATGTTCCTTTAACTTTTAAACGTGTTTCTGCAGAATTGATTATACGGAGTATAATTGGAAGGATTTCTCCATATGATACAATATCACAGCTAGTCCCATTAATTGTTTTAAATATTGAACAATATATTTCCAGTATCTGAAGTTTGATTTTTTCATTAATCTTCAATCGTGTTTTAAGTGTAGTACTCAATAAATATGGATGAATCAGCGTATCAAATTTAATTTTTAGAATATTCTCGCATATTACAAATTCTTCACATAAAACTGTTAATAGTTTTAACACATTTTCAAATAGTGGATTTATATGTAATATCGAATTAATAATCAATATTTGCATCCCTGGATAAAACCAAATACATATAAGTAACTCACGAACATTGTCTTTAGACATTTCCTTTTTGCAGTTAATGAAATCTAGTAATTCTTGAATAGAAGTTGCACTATTATTTTCTAATAATCCATCAAGAAGATGAATTAGAAATTTGAAGGTTGATAGATCGATTTTTTCAAGATCAAAATCATCAATGGTATAATTTGTTGGTTTTAAATTTATTTTATTAATAGCCTGCGTCATAAATCTAGGGTATGGTTTTTTATTAAAATTTCCATAAAACATTTATTCATATTTCTTTTTCATAGGGAATTTAAAATATACAGAAAACAGTAATGCTAGTATAAAAACACCAACCAATATGAGTGGCCCCTTTGTATCATCGTATGGGCCCGGATCTTTTTGACGTATGCGTTCAGCACGCCAAAAGAATAAATGCAATGCATACAATGCAAATATAATCGCTACCCCAATCAGTAAGGAACCACACAGATTAGCCTTATAGTTTTCAAGTCCTAACATTGCAGTTCCGATTCCACCTAAGAAAATTGCAAATTGAACCCAACTCAAAAAAGTTCGTTCATTAGCAAAAAACACTTTAGGTTCGACTCGTACAGGAATACTTATACGAAAGTCTGTATTATTATTATTGTTGGTTCTATTGTCGAATTTTAAATTATTTGATTCAATTGAATTCGTCAATTCAATCGATTTACAACAATCTGTTTGAGAGCTAACAAGGATCAGCTCATCATTATTAAACTTCTTCCGGTTTCTAATATTTATAAATGGATCCTTTCTAATATCAGTTTTCATTTGTGGTAACCAATATGGGATATCTTTAACATTATATAAAATAGCAATACCGTGAAGAAATTTGCTAAATTTATAAACATGTTCAACATATGTAGAGTTTAAAATATCTTCTAACCATGTGGGTTTACTTTCGTCGACTCCTTGAGTTTTAACTTCTAGTATTGCATGTGGAAACCGAACAATTTCTGTATCTTCGAGTTGTCTGAATGGCCACTCACACATAATATCAGTTCGGCGCCAGTTTTCACCATTCTCTTTAATCATACAAAGATTTGTGTCAAGACTGAGGCGAATTGTACTATCGTTAGGAATTTGAAAAGAAATACGTTTATAAAATGTCCTAATTTTAGGCTTTAAAGTGTGTTTGACAATGGCACCTTGAATTTCATCATATAAATATTTGATTTCCTTTCCATTTAGTCTGCTTACATGTTTCCAAACATCTTTACCTTGAATAAAATCATTAACATATTTTTCTTCTATTTTAAAGCGTAATTTTTTAGATTCCTCTCCAGTCCATCCCTCACAGTGCTTTTTCCTCTCAATAAATACGGTATCGGTCTCCTTACTGATTGTTCCATACCATCGAATTCGTATGGCTTCTGCTCCTTCGTTCTTTTTGAGTCGACCATCGTAAATGTAAAAATCATCATTATCAAAATACACTGACGTAACGCAGGTATCATGGGTTTTATGGTTCCATGCAGAGTACGGAGTCTGTCCTTTTTCATTTGTAAAAACATATAATGGCAGATTCTTAACAATACTTAATTTTAATGCCATCAAGTTATCAATATGAACCCAGTACTTATTTGTCTTGCGAACAAACACCAAGTTGGTATTCGATGTAGTATTGATATCCATTTCTTTTAGTTTGAGACGAGACCCTGAGTAAATCAACTCATCCAAATGATTAATTGCTTCTAGCTTCTCTTTTAATATTGTTTTGTATTTTGTTTCTAATTTAATACTACTTTTTTTATCATGGTGTTTAAGTATCTTTTTAAACCCAATAATTGTTATTCGTATGAACTCTGCAAATTCCTTCATGTCTTCTTGAAGCAAGTCAAAGATTTCTTTATTTCTCCCTTCGGAATTTTTTTCATAACTTATTAATGCTTCACGAAACTTGTTTGTTTGGTCTTTGATGAAACTAAATATTTTTTCAAATTCACTATGAATATAATCTTCAACCCCATCATGGCCATTGTTTATTGCGGTTATAATTGCATCGTATTCTAGATATTTATCTTGGTATTTTTTATTTTTATTATTTGCTAAATATTCGGAAAATCCCATCAATTGTTGTGTGTACAATAGTTATGTTTTAAGATAAAGCTGAAACAGATGTGACATCGATTTTCTACTATAAAGCAAATTATCTGTTGATTTAAATTACAATAGCTTATATACTGATATACTTATATTATGATGTTTAATTTTGTAATGTACTTAATAATATTGATGCAATTGCATTAAGAAAATAATGTTCTATTATTTCAATTCTGTTTATTTTGACTTTTTCTATTATCCAAATAAGTTTATTTAGTTGTCCAGATACAGAAGTTATATCTTGAAATTGACAATCCATTTTCCAAAGAAAAAACAATATACCCATAATAGTGTATAATAATACTAGTAATATCGAAAAAATCTGTGCAAGAATACTATTATTAACAATAACCTCGCGGCGCATTAGTTTTATTTGATGTTTAGATATGATCTGAGTATTGTGTTTTCTCAACTTTGTATTATAGATTACATCAAGAATATATACTGGTAATATTAACATATGAATGAAAATCCAATGATAAAAACGGGATGATTGTAAGTTGTATTTAAATTCTAATGATACTGCTAAAAATATAAATCCTAAAATCACCTGGAGTATAAACTGACCAACAATATAAATTCGATTAATACTTGTGAATTGTGTTACAGTAAAATTCTTTGTATTACTAGCTAACGATAAAAACCATAGGAGCAGCAAACAACAAAAAACTGTGTTAATTATTAAAATACAACAATCCATTGTTTTATGTAACATTATAACAGTATTGTTTAGGAGTATATATTTAACAAATCCAATGAATGCAACTATTCCAATAATGATATAAAAGCAAAAATATAAATACTTAGTAAAAAACATGCCTACCCTTACATTTACAAAGTTTTTAATTTATAAATAAAATATCTATATTAATGATAATTTTTTTAAATTATAGTAAACATTCTAAAAAAATAATAAGGTTCATATTTAATATATTATGTAATAAAACATTTATTTAAATGTTTTTTAATCCTTGTTAGCTAATGGACAACTTACATCTTTATTTAGAATATAATTGTTTAGAATCAATAGACTTTATTATGAAAATTCCTCATGAACACGTTAACAATAAAAAGATACATGAATCAAGTGCATTAATATTGGGAAAATTATATCAAAATCAGCATAGGCACGCTGAAGTAATTACGTTACTTAAATGTAAAAAGTTTCAAATGGATGAAATTATTTGCTCTTGTTGTCCAGATCCACAAATATTTATACGTGGAATTTCATATTATGCTTTAAATCCTAACTACAAGATAACGGTTGAAAATAATAAAAACACAAATAATCCATTTTTGATTTATTTACAATGTTTATTTGGATCATGCACTATGAATATAGCCGAAAATTTAATTAATGCTTTGGAAATTCTTCCTGATTTCTACGAAGCAGCATTATTATTAGGAAAAACAACTAGATGTGCAATTAAAACATCAAATCTTTATTTAACTAATCATATCAATATGTTATTATTTGTCCAATTTAATTACAATACAATGCTTGATACAACATTAATAGTTGATCAATCACTATATGCAGCATACTTATTTCTAAATGATGATTTAGATGCAAGCATTAAGCTGTATAATGAGTTGATTTATAAAAAATCAAACTATGATTATATAGAATATTATATTTTAATTATTCTAAGTACTAATGATAATAGTATTATAGCTAAACTTAACTCCATTCTTAATTTGGTGCCTAACACTATATTAATAACAGCTGTATTATTATTTTTAAACAATAAAATTAAAGAAGCTATAAAACTAATGAAAAAACTTATTAAAAATACATTAATTCATAGCCAAATTATAATATACAATCTAATTCTTATAAATGTACTCATTAAAAAAATAGATAGTGAATATGATGATGTGGTTAGCGATATTTCAACCAGTATCAGCAATATCTTGAACTTAATTTTAAATAATGAATTACTATCTCGGTATGCATTTACATTAGGTAGTGTATGCTATAAAATTAAGGAATATGATAAAGCATTGTATCTGTTAAGTCACGTTACAAAAAATGATTTAAAAGATAGCCTTAAATTACGAGGTCGAATTTATTATAAATATAATATGTATGATGCAGCAAAAGAACATTTTGTATTGGCATCAAGCCATGGAAGTGATGATGCATTATTATATTTAGCAGAGTTGTATAAAAAAATGAATAATAGCAAAGCTGCCATTGAAAGCTACAAAAAATATCTTTCAGTTAGTACGCAAGGTAATAATCGTGAACTGGTTGAAGAATATTTAAAACAATATTATGAAGAACAAAAAAAATTAAATAAAAATTATTAAAATAATCTGTATTTAATAATTTACAATGATCATGGATATTTACAAAACTATAAATTTTGTTATTTTCCCCATATGGATATTGAAAAAAAGTTAGAGAATATAGATCTTAGTAGAAAGACCGTGGTTAGAAGTATCGTGTTTGTAGGTCATGTTGATGCTGGAAAATCTACTGTTTGTGGCCGATTATTGGTGGATTTAAATTTAATTGAAGAACGGATTTTAGAAAAATATAAGAAGGAGGCACAGGAGTGTAATCGGGGAAGTTGGTATTTGAGTTGGTGTATGGATCTTAATCCTGAAGAGCGTGAAAAAGGAAAAACACAAGAAATGTCAATGTGTTCTTTTTATCTCCCCACATTTGGAGCTGATAATGTTAAAATAAACATCATTGATTCTCCTGGACACAAATCATTCATTGGAGAAATGATTGAGGGTGCATCGCGAGCTGACATTGGGATTCTAATTGTATCTGCACGCGCAGGTGAATTCGAAGCTGGATTTAAAAAGGGACAAACTAAAGAGCACATACGGTTGCTTCGGGCCGCCAATGTGAACAGAATTGTGGTGTTGGTCAATAAAATGGATGAGTGTAATTGGGATAAGGATATTTATGGAAACATTGTTGCAAAACTTGGCAAATTCATATCGCCTTTATATGGAGATGTTAAGTACATCCCGGTAAGTGGATACACAGGAGACAATATCGTGAATTCTAAAACTCTAGAGTGGTATTCTGAGGAAACATTTGTTAAAACACTGTATAATGTATGTGTTGAACCCCGAAAATGTGAAACTGGAGGTCTAATCTCTATCGTAGTCGAACGTAGCAAAGGAAGTGTCATTTCATATTATGTCAAAATTGAAGAAGGAAGGATTGAAAAGGGAAAGACATACTCTCTAATTAGTGGGAAAGGAGTTAACAGTATCATCGTGGTGGATGTTAAAGACGATGAAGACTGCGATGTTTTTGAAACTCAAATCAATGATGTCTACAAAATTGTAGTGAGCGGATACAAGGATGAAATTAGTACTGGAAGTCTAATTGTCAGTAGGGAGTTTGAGGATCGTTTTTGTGCTGTCAAGAAGATAACTACTATTCTTGGGATATACAAGGAAGTCGCAAAATGCATATCAATTGGATACAAAGGAATAATGCATATAAATGGTGTCCAAACAGAGTGTCGGATTCATAGCATGTACACATCAGAAAAGAAAAAAATAAAAGTTGCAAAGAAAGGTGAAAAGACGATAGTAGTGTTTGATCTTGAAGATGAAGTGATTTTACCTACTAATCCATCGAAAGAAGGCCGCATAAAATTTAGCTTACGTGACGAGGACATCACGGTTGGTGTGGGTGAAATTATAAAGGAAATGACATACAAAAGATATACCACAGTTTATTAGAGTTTTTGGTCTATGCTTTGGGACATACGTTCTTTCATGTACCGAACGTACTTCCATCCCGATAAAGCCCTGAACCCTTGCGAGCTGGATTAAAAGGGAAAACTAAAGAGCACATACGGTTGCTTCGGGCCGCCAATGTGAACAGAATTGTGGTGTTGGTCAATAAAATGGATGAGTGTAATTGGGATAAGGATATTTATGGAAACATTGTTGCAAAACTTGGCAAATTCATATCGCCTTTATATGGAGATGTTAAGTACATCCCGGTAAGTGGATACACAGGAGACAATATCGTGAATTCTAAAACTCTAGAGTGGTATTCTGAGGAAACATTTGTTAAAACACTGTATAATGTATGTGTTGAACCCCGAAAATGTGAAACTGGAGGTCTAATCTCTATCGTAGTCGAACGTAGCAAAGGAAGTGTCATTTCATATTATGTCAAAATTGAAGAAGGAAGGATTGAAAAGGGAAAGACATACTCTCTAATTAGTGGGAAAGGAGTTAACAGTATCATCGTGGTGGATGTTAAAGACGATGAAGACTGCGATGTTTTTGAAACTCAAATCAATGATGTCTACAAAATTGTGGTGAGCGGATACAAGGATGAAATTAGTACTGGAAGTCTAATTGTCAGTAGGGAGTTTGAGGATCGTTTTTGTGCTGTCAAGAAGATAACTACTATTCTTGGGATATACAAGGAAGTCGCAAAATGCATATCAATTGGATACAAAGGAATAATGCATATAAATGGTGTTCAAACAGAGTGTCGGATTCATAGCATGTACACATCAGAAAAGAAAAAAATAAAAGTTGCAAAGAAAGGTGAAAAGACGATAGTGGTGTTTGACCTTGAAGATGAAGTGATTTTACCTACTAATCCATCGAAAGAAGGCCGCATAAAATTTAGCTTACGTGACGAGGACATCACGGTTGGTGTGGGTGAAATTATAAAGGGAAAATGACATTACAAAAGATATACCCACAGTTTATTTAGAGTTTTTTGGGTCTTAATGCTTTTGGGACATACCGTTCCTTTCTATGGTACTCTGAGCGTACCTTCACTATCCTCAGATGACAAGCACATGAGACGCAGTAGGAATCGATATTGAAGAACGAAAGGACATCTGGGCTTTGATAAATTGTGGCAAGGTTAAATCATCCATGACGCAGCCTCAATAATAAGATGGTAGTTGCTCTTGAACGGCCTGTCTTTGGAACAGTCGCCGCACTGGACCATGGACATATTACCCCCCCCCATGGTTTGGCCTTCCGGGCTCTTCTTTACGGGCACCGGATTGGTGCAATTTTTAAAAAAAAGCGAATAAAAACAAAAATTTGAGTTTGAGGATCGTTTTTGTGCTGTCAGAAGATAACTACTATTCTTGGGATATACAAGGAAGTCGCAAAATGCATATCAATTGGATACAAAGGAATAATGCATATAAATGGTGTTCAAACAGAGTGTCGGATTCATAGCATGTACACATCAGAAAAGAAAAAAATAAAAGTTGCAAAGAAAGGTGAAAAGACGATAGTGGTGTTTGACCTTGAAGATGAAGTGATTTTACCTACTAATCCATCGAAAGAAGGCCGCATAAAATTTAGCTTACGTGACGAGGACATCACGGTTGGTGTGGGTGAAATTATAAAGGGAAAATGACATTACAAAAGATATACCAACAGTTTATTTAGAGTTTTTTGGGTCTTAATGCTTTTGGGACATACCGTTCCTTTCTATGGTACTCTGAGCGTACCTTCACTATCCTCAGATGACAAGCACATGAGACGCAGTAGGAATCGATATTGAAGAACGAAGGGACATCTGGCTTTTGATAAATTGTGGCAAGGTTAAGATCATCCATTGACGCAGCCTCAATAATAGGATTGTTAGTTGCTCTTGAAACGGCCTTGTCTTTTGGAGCAAGTCGCCCGCACTGGACACATTGAACATTATTTACACTCCCGCGATTTGTTTTGGCTCTTCCATGGTTCTTTCGTTTAACTGGCATCTGTATTGGGTGCAAATTTTTTAAAAATAAATAGCTAGATTAGAAAACAATAGATTTATTCATGTAACAGGTATAAGACAAGTGTCAAAACATCAGTTTCGCTTTGCACAGTATCATTTCATATATTTTGGGGATTTCAAATTCTATTCTAGTCCTCAGACTTTTTGGTGCTGGTGGCTCTTCGATGTCTAAGCTACTTAATATTTCATTTTTGATGAGGTCCAAATTGTTCCTGTTTTTGTAGGGTGTCTTGCAGATGACGTTTTCAGGGTAGTATGCTGCCTCTTTTTCATTCATAAATTTGACATAGACATCGTATTTCTTCATGATCCGTTGGATATGTTTTCCTGACGTCCCAATGATTTTCTTATGATGTTTAAAATCAACCGAAAACAAGAGGTATGTCGGATATTCTAAATTGATCAGTCGCAATGTTTCGACCATCCACGGATGTTTGATGACAAATGGTAAACTTGAATGTGAGTTAGCTCGTCACATGGATTGAATTGACGGTAGTCATCCGAAAATCTATGATCTTCAATTTCTTCCGCTAAACTTTTTTGGAAATTCCCCCCAGAACAATAAAATATTCCTCCCGGATTTTTTGGTTTTCCGGGTCTTTGTTAACGGCATTTGTTGGGTAAATTTTAAAAAAATGGTAGTTAAAAACAAAGTTTTTTCGTTACAGTTAGACAAGTTCAAACTTCGTTCGTTTGCCAAAATCTTTCATATTTTGGGATTTAAATTTATTTTGTCCTCAACTTTTGGGGGGGTGCTTTTCGATTTTAAGGTACTAAAATTTCTTTTGGAGAGGTCCAATTGTTCCTGTTTTGTAGGTGTCTTGCAGGATGACTGTTTCAGGGTAGTAAGGTGCTCTTTTTCATTCAAAAATTGACATAGACATCGTATTTCTTCATGATCCGTGGATATGTTTCCTGGACGTCCCAATGATTTTCTTATGATGTTTAAAATCAACCGAAAACAAGAGGTATGTCGGATATTCTAAATTGATCAGTCGCAATGTTTCGACCATCCCACGGATGTTTTGATGACAGATGGTAAACTTGAATTGTGAGTTAGCCTCGTCACATTGGATTTGAATTTGACAGTTAGTCATCCGGATAATCTTATTGATCTTTCCAAATTTCTTCCCGCTTAAAAATCCACTCGTTTCGCTATTTACGGAAAAACATACCTCAAGATCCGACACGTTCTCTAGCACAACTTCTTTAAGATCATCCACACGACCTGTGAACGATGTTGTCTTATTCTCCATCCGACACACCACAAGAGGATTGGTACAACTGACTCCACACTCACTTCCTCCACCACCGGTACGGATGCTAAGGGTATAAATCTTGTAATAATGCTCGTAGAACTTATGTTGTATTATTTTCTGGTGGGCCAGCTTGCCCGCAGCTAGAGTTACTTTGTTGTGTCCGATAATCAAATACGAGTTGTATGTATCCAAGGCAGCAACAAGTTCCGAGTAGTACAGTCTGATTGTTTGGAACAATCTAACTGGACTGACATCGAAAACATCATAATACTCCAGGGATCGAATACTCACAGGTTCGTAACGGATTCGTTCTGTTGACAAACACGAGATGGCAATCCCGTTCATCTTGAGATAAATGTGTTTTAAATTTTCCTCATGGATGTTCACCTCAATACCCTTAGATGTCTTCAACAATCTCATGTTGAAATTCTTCAGCACACTGCACATTCTCAAGGTATTGTACACTAGATTGATGGGGGTCTCTACAGACTTGGGTATTGTAAAAAACAGACAGTTCGTTTCTGACAAAGAAACTCCCTCTTTGTAGAAATCTATGTTGAGTCTTTTGGGTGTTTTCCCGAACGTATTCACCAGCTCGGCTCTACACTCAGAATTTTTTTGGAAAAAAATCCCTCAAGTTTCCGAACCGTTTTTAGGACCAATTTTTTTAGTTAATTCCCCAGGACCGTGGACCGAAGTTTTTTTTTTTTCCTTTCCGGCCCACCACAAGAGGATTGGTACAACTGACTCCACACTCACTTCCTCCACCACCGGTACGGATGCTAAGGGTATAAATCTTGTAATAATGCTCGTAGAACTTATGTTGTATTATTTTCTGGTGGGCCAGCTTGCCCGCAGCTAGAGTTACTTTGTTGTGTCCGATAATCAAATACGAGTTGTATGTATCCAAGGCAGCAACAAGTTCCGAGTAGTACAGTCTGATTGTTTGGAACAATCTAACTGGACTGACATCGAAAACATCATAATACTCCAGGGATCGAATACTCACAGGTTCGTAACGGATTCGTTCTGTTGACAAACACGAGATGGCAATCCCGTTCATCTTGAGATAAATGTGTTTTAAATTTTCCTCATGGATGTTCACCTCAATACCCTTAGATGTCTTCAACAATCTCATGTTGAAATTCTTCAGCACACTGCACATTCTCAAGGTATTGTACACTAGATTGATGGGGGTCTCTACAGACTTGGGTATTGTAAAAAACAGACAGTTCGTTTCTGACAAAGAAACTCCCTCTTTGTAGAAATCTATGTTGAGTCTTTTGGGTGTTTTCCCGAACGTATTCACCAGCTCGTGCTCTAACACTCTAGAATCTATCCTGCTCTGAGTGTTGTTTCCTTCGAAAAACAGAGACATACCTTTTTTATGAAATAACAAACTTTTTTCAGGCATTGCTCCGATATTGGGTGCAAATAAAAATTTATAATAGAGTTGTTTTAACCGGGTTTTGATTTTGTTTTATTGATGTTTTAGACAACGGGTAGTTAGTGCCTATTGAAGAAAAGAAAAACGATGTCGACAGGATTCGAACCTGTGCAGGACAAGCCTAGCAGCTTTCTAGACTGCCGCCATAACCACTCGGCCACGACACCTTAATTTTTCCAATGGCATCACTATACTTTGATTAGAGAAATGATAAAGAAATCAAAATCTAGCAGCTAAGGTGGTTATTATTATTAAATTATTGATTAATATAAAACACTCATTATTTAATAATAATGACCATTTTAGTTGCTGGATTTTGATTGTAATAAAAAATTGATTATTAATTTTCTCCTTAATAATGATAAATTTGTTTCTCAATATTATTATTAATTGTACAACTATAACTATTCCATCAAATGATTATATAGTAGTACATCAGAATAGTGAGATTTGTTTTACAACCAACATCAATATCAAAAAAGAATGTATTCTCAAAGCAGGATCTTTACTGTTCTATAATAATGGAACTTATAAAACAAAAAAAGATATAGTTCTTCAAAGTGGTCAAATATTAAATAGTTATTTATTTAAACCTCAATTTGATATTATATTTAATTTAAATGATTATCCATGTCTTATAGATGGCATAAATATTCAGGGATATCAAGGAAAATTGTCATCAGATAATATTCAAATTAAAATAATGAAATCTGAAGATTTAATATTGTCTAATAAATCAGATATTGATAATGTATCTAAAAAATTTATGTTGGAAGTTATTAAATATAAAAATCAAAATCATATAGTAAATTTCGAAATATTACATCCTGAAACGGTTGAAATTTTATTAGATAGAAATATAAAAACAAAAAAAAATAAAAATCAAATAAAATTATCAATTCCAACAAGTAGAGTTCCAGTTTGCCCTATTATATATGATTCCAACTATAAGTTATATTGTTTTACTCAATTTATAGTTAAAAATTCTATTGAAGTAGAATATGTTTCAAATCAAATTTTATTGCCAGGTAAATATATAACAACAATGAATTGTCATGGATTTATTGATTTTCCAAAAGTTAAAAATACATTGTGCAAAATTAAATCAGATTTTGGAATATGCATACTAGATTTTAAATTAAATAAACTACAAACAACAGAAAACAGTCGCTGGAAATGTAATAATGCTGATGAAGTTGAAATTCATAATGATTTCATAATAAAGGAATATTATGATGGAGATACAGATAGTGTTAGCTTATGGATATATACTATTGTAGTATTAGTAACAATTGTTATTATTGGATATTTACTTGTAAAATTAATTAATTATCTTAGATACAAGTCTATAAATACTGAAGAAAATTAGTTTAAATACTTTATTTTAGTCAAATACTATATATAATGTATTTTATCAATAAAGATTTTAATATTAGATAAATAAAAATAATTTATATATAATTATTATTCCTTGCTTTATAATATATAATATGTTTAATAACAAATATTAATTATAAGTGATTTTTATTTATTTAATACCAAAAATTTATTTCTAATTTTATTGATAACTATATATTGAAAGAACATGGTCTTGATATAGAAATTGAAGCTAGGATAGGATATATTACATTTTCCATAACAGGAAATCGTATAAAATATGATACATTACACCCAGTTGTTTTCACAAGACTTCCACTTTTTATTCAATTTAATAGTAATATTGGAGAAGAACATTATAAATATTTCAAGACTAAAATTGGAAAATTGACGAATGACTCAGTGGAAATAACAGATACAATTTTTATAGGAAATAATTACAGAAAAATTGTGTCTACAGATGGAAAAGCAATATTTCAAAAAAAAATACGACGAACTAATCTTGACATATACTTTCCAAATTCACTTTTTGATATCCGAATTTCAGTATCTACAGAAACAAATGTCCAAATGCCACCATCATCATTTATAGAAAAAATCATCCGAGAACGAAAAAGGACACAATATATGATGGAAAACTGTACATTTGATTTCACCCAAGTCAAAGATAATATGGGAAATAAAACATCATATGAAGTCGAAATCGAATTAACCAAAACAGATGTAACTGGAACAGAGTTTTTTAATAAAATTTATTTATTATTAAATAAATAAAATTAATTAATATTATTCATAATCTCAGATATTATTTTATATGTAGAATATAAATTCTTTTCAAAATTTTTGTAGTGAACCCTATGCAAATTATTATAAAGACACCAACCAAACTAGTTATTAGAAATTTTGAGAATTCAATGACAATTGCACAATTGAGAGAAGAAATCAGAAATAACATGGGACTATGCATTTCCCATATGTTCAATTCTATTGATAATATAAAAAATGTGTTTAGAAATGGACAAATAGTGTATGCAACACCCGAACTACTAGGAGGTGGAAACATGACTGAAGGAAACAAACTTATTTCACTCAATTCTTTGAAGACGAAAATTTGCCGAAAATGTTATGCGCATAATGCTTTAAAAGCAACTCGATGCAGAAAACGAGCTTGTGGCCACAGCAATCAACTCCGAATGAAAAAAACAGTTAAAAAGAAGTAGTAAATTAATAATTTATTACACAATAGCATTGTGGTATAAATATTGGACAAAAGATACAAAGGATCATTCAATAATTAGGAAAATTAATTAATATGATTCATTATCTGGTTATTTATCAAAACGTAATTTTATGATTTCATTTACTTGTTGGTCTTTAATTGTAAGCTCTCTGTTATAGAAATGATGTTTGCAACAAATCTTCACAAATAGTATTTCTGAAGGAAATGATATGGGTATCATTTTAACTTCCTTTGTTAAAATGAACTCATTATTTTTGTATGTGAATGAAAACAGCTGAAGTTCTTTTTTGGAAAAGTCCATTTTATCAAATGGCAAACTAATCACCTCAAATTTAGGTTTTGATTCATTACATTTTGAAGATAGGGACTTTATTAAAATATTATCTCTGTAGAAATCATCTTCACTACAATTAATTATAATAGCTGGAGTAGACATATACTAATCAAGGGTTATTAAAATCTGTGATTTTAGTAATTAAATGTTGTTCTAATGATTTCAATACATCTTTACGTCTTTGGATTTGATTTGATAAATAATTAGTATTGTTTGCATGATTAATATTGTGTTCTAAGTTAGAAAATGCATTTTCTCCATTAACATTGTTTGTAACTGCCTCTCTTCTAGAAAAAATTATTTTATTTATTAATCTGCTTTCTTCTTCAAGAATCTCGGGTCTGAAATGTTCCATATTGAGGATAATAGAAATATCTAATTTTATCTTTGTTTATATATTTTAAATATTACAAACAGTTTTCTACAAATAGGGCAATTTTCATTTTTTTCTGTCCATTCTTCAAGACAATGTAAATGAAATGTATGACTACATTCTCCTGTAACAGGAACACAATTGATGGGATGATTACATTTATTGCATGCTGTGTTGAATTCTTGTTGACATATTAGACAGCAATCTTCTCTAATATCCCACTTCCAAGCATATCTAGGATAAACTTTAGTTATTTTAATTTCATTCATTTGTAACTGTTTTTTGGTCATTATTATAGGGTTTTACTATTTTCACTTAATAAACGAATTTCTAATAACAACTTGATTACTGATATTTGAAGCAATCAAAAGTAATCACTATCAATTAATTTTACAATATGATTTAATTATTAATAGTTAAAACACATTATAATATCATAAATATTAATAATATAATATAAACACACAAGTTGTTTTACAAAATTTGTTCAAAACAATCATACTTAATATATTTTTAAGTAAAAAATTAATTTAATTAAAATAAAAAGGATTAATCCTTTTTTATTTATAATAAACTTATCCCTAGTATGATTATTAAACTAATAAGATTTATTAGCTTTGTTTATTCAGATAAAAATGTTAACCAAACAGTAAAACAAAAATTTTTAAATACAAAAGGAACTATAATAGATTTGAATTTAACAAAAAATAATGATATTGCCTATGAAACAATAAATATTCCTTATAAAACAATATTAGATAATGTAGAAATAATATATGGAGATAACCTTGAAGCTTTATTTAAAATCCATGATCATTTAGGAAGTAAAATAATCTATTCTAATATTAATAAAAATAGTTCTTATATAGTTTTATTATCAGAATTTACTAAATTTATTATTCCACAGGAAAATGATGATTTGTTTAATTTAAAAGAAAAATTGATTGATAAATTATATGAAAGTGATAATTTATTTTTTTCTATAACAGAAGCAGTATGGTTTGTAAATAAAATAAACAGTTTAAATAAATTTTTATTAACAAAAATAATTCAAGATGAAAAAATAATTAATATTGAGGCAGCTCAAAATGAATTAATAAAAATATTAAATGAAATGAACATTCATATAGACTTAGATAAATTGCCTATAATTAAACAAGAATCAATAGACAAAATAATTTATAAGGTATTCTATTTAACATTTAAGAATAAAAATAATGGTAAAAAATATAAAACACCCATTTATAAAATAAGAATTAAAAATAATTGTATTTCAGATATTAATATGATATTTAAAAAAAAAGAAATATTACCATCAATTATATTAATAGGAAGTATTACATTATTAATTATTATTGGTATAATTATTATATATATATATAAAAATAAATCGGTTAAGCCTCGAAGTATTTATACCATAAAGAATATATCATAATTAATTATATATCGAATAACTAGTAAATAAATTAATAACGATTATATAACTATTTTGTGTTATATTAGTTACTTAGTACCTTATATAAGATATTTTGAAAAATTTACCCATGCCAAAATCTTATAACTATGATATTGTTGATACTGACACAACGGTAAAAGCATGTATAACTAATGCCCAAGTATCGTTCAAAAAGACAAGAGAGACATGTAGTACACTTCGGGGAAGATATGTGGTGGATGCAATCAATTATTTGTATAATGTTATTGGAAAGGTGGAGTGTGTTCCTATGAGACGATATGCAAGGGGATGTGGAAATACATCTCAAGCATCAGCATTTATGAATGGAAATTGGCCTGCAACAAAAGGACGATGGCCCGAGAAAAGCTGCCGATATATAATTAAATTACTCAGTAATATCAAAAACAATGCTGTGGTTAAAGGACTGAAGGTTGAGGATCTTGTTATCCAGATGATTTCAGTGAATAAAGCTCCTAAAATATTTGGACGATGTTTCAGGGCCCATGGACGAGTTAATGCATTTAACAAAAGTCCATGTCATATCCAAGTAGTCTGTGGCACTGAAACAACAGATATTTCACCTCAAGATGATCTTGTACAAATAGAATAAACATGGTGTTAATCACATAATTTATTAATCTTTTCTCAAATCAATCACTTCTTTTTCAACCTTGTTGTTATCTAATATGTAGATTTCTAAATAGTCACCTGTTTTTACATCTGCTTCAGCTGCAGAATGAAATGCTTTTTTGACCAAGCACAGAGCATCTTCGAATGATATCTGACTATAGTTTTCAAAGTTTTTTCCACTGATCCAACTATCAAATAATGGCTGAATCATAGCAGTACCCGATCCATTAACTCGACATTCTGTTTTCTGATATGATCCAATGCAATCATATGAATAAATTGTAGCTTCGCCATTTTCAAATCCACAGAGACAACAATATGAATATAATGGGAAAAAGCGATTATTATATAATACATGATGAAGAAATTCTGCCAGTGCACTTAGAGTCATTTTATCATATTGTTCATATTGTTTTAATTGATAATCAAGAGTTAATGCAATTTTATGGCTATCTGCATGAAACCCTACTGTTGTTAAGAAGTAATCTTTGATTTTATGGATCTTAGTGCTATACCGAGTATATATATTGTACTGTGCATTAAGTCTTGTATCTGCAGCAATGATCAATTTATTTTGTAATTTGATTGTAAGAGTTGTTCCTCCATTATCTTCATAAAGATCCTCTTCCTTAACTTCATTTTCTGTATTTTGTTCACAAACTGTTGGGAAATGTTTTATTACATTAGTTACATCTTGAATAACTTTGGAAAATGAAATAGTTAATCTTGGCCTTTCTATTATACATGGAGTATCAAATTCCAATGGATCTTCCTCCACAAATTTCTTTGTAAAATTAATATCAATATTTTTAAAAAATGTATTTGTTCCAAACATAAGGGGAAAAAATACTATTATCTAATTATTTAAGTAATTTATTTAATTCAGTTATAAGACTTTTTTTGTCAATTGCAATTCTATGTTGTGTCTCAATAACATATATAACTAATGTATTGGTTTTCTTTTCATTTATCCCTGTAAACACAATAAGTTTATATTTAGCTTTTTTTGCATATTCAACCTGTTTATTAAATATCATTTTAGTTCCATACATTATTTCACAAGAATAATATTTAATAATTTCATTGAAAATTTCCATTCTTTCTTCTATTAACAATCCATACAAAGATCCAACTAAAACATCTTTATTCCCTAACGAACAATAATTTTGAGAATATTCTTGTTTTTTCAAAATTATTGTAAGCAATCGACTGATTCCTATGGAAAATCCAACACAAGGGGTATTAAAATTGCTGATGGTGTGACATAAATTATCATATCGACCTCCACCTGTGACTGATCCAATTTCTTCATGACATAGAGTTCCTTCAAAAATTAGTCCAGTATAATATGATAACCCTCGTACTAATGTTAAGTCAAATGTTACATAAACATTCATTATACTAAGATACCTAAACAATACTTTTAATTCTTCGCATGCTGTTTTAAAATTGTTCATAGAGTCAATTGTTATTATTTTATCGTATATAGATTCTAAATACTCAATTATTGCCTCATTATTGCGAGTACGGGTAATTGTAAATAAATTAAATAACATATTTATTTGGTCATCCGTTAAACCTTTTGCTTTAAATTCAATTTTTAATTCTATTAGTGTTAACTTGTCAAATTTGTCAATAGTACTACAAATTGTTGCCTTTTTATCACCAAAAATACCGCACGTTTCAAGGTAACCATCAACTATTCGTCGATCATTAATTTTTATCATAAAGCCTTTTAAACTAAATGTAGTAGTGATAGTCTCCAAAATACTATGTACCATTTTTAGAATTTCACTATCATATACCATTGGTAGATTTGTTCCAGTGATGTCAAAGTCAGCTTGTGTGAATTCTCGAAGCCGACCCTTTGATGGATTGTCTCGACGATATACCTCACCAATTTGCCACTTTCTCATTCGACTAATTTTATGTTTGGAAATAAACCGTGCAAATGGCACTGTCAAATCATATCTTAAACTACAAATATCGCCTTTTTGGTCTTCTAAATTAAATATTAATTTTTCATCCTCACCATATTGGTTGTATAGATTTTCTCGAAGTTCAAAAACAGGAGTAAATATTGGTTGACCTCCATGCCGTTCAAATATAGTTTTACATATATCTATTAGTTGATTTTTAATTCGCATTTCATGAGGAGATAAATCGATTGTTCCTTTGGGGCATCGTAAATTATCATATTTTTTCATGAGGGTTAGTAAAAATACTAATATTGCTTATATCGTATTTGAAATTGGCCAAGTTATTTCAATTCTAGTTAATATTTATGGCCCAAATTTTTACCCTTATATATGAACTATTTTATTTGGCTGGGTACTAGCTTATCTACTTTTATTTCATTGGAACTAGATAAAAATGTTACATACCTTGTTCAAACTCCACTTGAAGGAAAACCTGCAAAAATAATTAAAAACAATTGCATATGTTTAACTAACCTTAATATTACTAAAAATACATCATACAACACACTGTGTGATATATATAAAGAATTAGAATTAAACAAAACATATACTATTTCTTGTTTTTCTGAGAACAATATTATAGATATTTGGGTAATTTTATCATTTTTTCTTAAAGGTCTTTTATCAAAAGAAAAGTTAAAAATAAATGATACTATTTACATTTCATTAAAAGACTATTTATCTAAAGAGGTGTATAACAAAGCATCAGCGGCTGGAACAAATCTTAGATTGAATACATTTATTATTCCTGAATCATTATCTGCAGTATTATATTTAGTAGAGTTTTACAAACGAAATACATTTACAGTTATTAATTTTCGAGGAACCCAAATAACTAAAACAAAATATAATATTATTTATCCCGATAAGAAAGAAAAGAATGAAGAAAAGAAGATAGATAAAATTAAAATCCAATTAATAGATAGAATATATTTTAAAAATGGAATTAGTGATATTGATGTTGAAAATGCAATAACAGATGCAATAAAAAAAGCTATTATATCTCGAATAAAGGATAACATTATATTTTTTCCAGATAATAATATTGTAGATCCACAAGATACACCATGGTGTAATATTTCTAATTTTAAGGAACGAGTTATAATAAATTTAAATTTTGGATTTTCAACTTATGAACATCTTGGTATTGACATATACAGAAAAAATGAAAAAATTCAAACTATACCTTCAATTAAAATAGATTTAAATGAATTAAAAGCAAGTTTATCAAAAGAATGTATGATTGAAATGAATAGTGATGAATTTTATATTAGTAGTTACAATGACATTCTTAAATATAATAATATATACTATAAGCTTATTAAAGTTGATTATAAAGCAATTTATAGTGGAAATTTGAATTTTACCAATTTTATTGTCGATGATCCACTTGTAACTCACAAGCAGTACAATATTTCATCAGGAACCTTTGTTATTATCAACAACAAATTAGAATTAATAGATCAAACAGTTAAATTGGCTGATGACATTGATAAAGAATTACTCAAGTTTAATTATATTGATAAAACAAATTCATTGTATCAAGATTTTATCACATTCCATCACCAATACAAACACATTCTTTCAGAAAAATATGATATAGCACATGATTATCTTCAACGATATAAAGCAGTAGAAAATCAAGAATTAGATATTAAAGGTAAATTCATATTACATGAATCAAATTTAAAAATGTTAATAAAAACAATTGATACAGCTCGAGAACTGGGACTTGATGTTAATGAAGATGAAAATTTTCTAGCCACAATAAAGAATGATGTTCAAGCAGCTGAATTAATAAGAAATAAGATTAAAGAGATAAAAATAAAAATAATGCGAAAGAAAATAGAAAATGAGAAAGCTTTAATTAAAGAAAATAATAAAGAAAATAAAATGACTGATACAAATAGTAATGTGCCTGATAAACCTTTAGAAAAAACGAAACAAATTTTTGAAAAACTTCAGCACAATCTTGAAAATAAGAAAAATAAATTTAATAATTTGGAAGTAGAAAGTGTTGATTCAATTGAGAAAGAAAAAGTTGATAAATTATTTGAACAAGCTATTAAAGAAGAAAAAGAAGCTAATCAACAAAATGGTTCCAACAAAAATAAGGAAATTTTATAATAAATAAAATCAATTTATTTTCAATTCGTTTCTAATTGTAAATATTTAACCCTAGATGCAAATGAAACAATCTAAAAATTCAAAAATAGAGATATCTACGTCAGGATATGATTCAAATAAAGCAATAGATATTTACAAAAAGGAAATGGCTAAACATCTAAAAAAATTTTCAAGTGCACTAGAATGGTCTGATTTTATAGGACTACTCGCTACATTAGAAGAAATTATTAAAGAACATAATGTCAAGAGTAATAAAGAATATTTAGTTGAAATACCATCTTTAATTAAACGATTAAGTCAATGTCTTAACCCTATTCTTCCAAACGGAGTTCATTCTAAAGCAGTTGATGTATATGAAACTATATTATTATATTGTAGCGATGAATTGTTCAGTCTAAGTATGCAAAACGAATGCTTGGACAATAATATACCTGAAGAATTAACTGCTGTAGTTATAAATGAGATGTACAATTCAAAAATATTTATAGATGAATATTTTAATCCAGTTAACACTGTAATAGATACTAATATAATTAATTATTCACCACATAATAGATTTCTTGTAGAAAATTTTAACTTATTATTTATGCCACTGTTTACTTTTGGTTCTAATGTTCGTATTCTTGTTATGGCACAATATATTCATCTAATTAAAAATCGAATTGTTCCACTTGTCTTGGAGGAAGTTGATGTAAAAATTGTTTTGCGTGGTCTTCTACCATTGCTAGAATCTGAATCATCCGATTACTTTGAAAATGTTTATGGAATATTAATGACTATTTATGGAATTTATCAATTTAAATATAATAGACAGAAGTCAATTTATTATATGCTATATTCATTGTTTATAGAACATGAAGCATTACGATTACCAATATTTAATTTTATCAACAAACACAAACAAAATGATATGACATTTGCAACCATTAAAGATTATGAAATGCTTACAAAGGCATACTCACTAGGATTGTTATCTGATTCATTATTTGTAATTCGTAATGCACTAGATCATACAAATTCTCATTTTCCCTTAATGATCAATGTTGAAAAGGAAATTATCATGAAACATGAAGCACCAAAGCCAAATGAAGAAGATACAAATGAGGAAATAATTGAAAATTCAAACTATAGTACACCTAATCTTAATACTATACAATTCAAAAAGGAATATATTATAACACATGAAAAAAATAGTCTATGTACAAATGACGAAATAGAAGCAATATTGCACCAATTTAATAATAATTTAAAATGTAATGTAATAACAATTTTACTGAAGAAAGAAAATTCTCTTATTAAAAAAGTTTTAAAATGGTTCAACAATGAAGAAAAGAGTAATTTTGATGAGATTATTTCTACGATGGAAATTGTTTTAAATGATAATACCGTACCTGTAAAAAGCAGATTAGATATTTTTTATAGATCTATAAATATTTTAAATGATCGGCTTATTGATACATCTGACCATTATACATTTTTATCTCAAATTATTAACCGCTATTATATAACTAGTATTAAACAACTAAAAGTATTAGTTGAAAATGAAATGAATGAAAAAAATATACCTTTTGAAGAACTTGAAGGTATCTCAGCATTAAAAGCTGTCAAAATCTTTTTTAATAACGGAATGGAAATAATAAGTAAAATGATTTTTGAATTATTATGTACATTGTTAATGAAAGAACCTAATATTGACGAAGCTAGCAATGTTATTGAACTGTTGATTTTCATGCTTACAATATTTACAAATGTTGATAGTTCATTTTACAAAATCCATCTATTTTTATTTACTATTGTAATTGTTAAAAGTAGAACTACCATTAAGACTGAACTATTACACAAATATATGAATATATTTTATAAAAAGATTAAAGATGAGCGGCTTGATATTCAGTTTGATCAACTTACTGTTGATAGTTTAGAATTTAGCATAGATGAGTTCTATAAACAATCAAATTCATTGAATACTGACGAAGATTATATAACAAAACAGCTTATTACCATTTTGTCAAATGAAATTTATAATATTCAAAATTATAAATTAGCACGTAAGATTATTAATATTATTGGAGTTAACAATGTATATAGTGAAGTTATTGAAATAAATGATGCATTTAATGATGATGATCTTTTATTCATATGTAAATATCATCAAGTGTTTGGCATTCCGTGCTATAGCAATGAGTTTATTCAAGCATTAGAACATTATGCTAAACAAGATTATACTAAAAAATACATCATTGAAAATATATTATTTAAAATTAAACCAAATTTCAAAAGTGAACTCAGTATTATTTTATTTCAAAAATATAGAGAAAATCATGATCACACATATATTATACTTTATGATATGGAAGATATGCTAATTAAAACACTTCTTAATAACAACGTTAACATGGATATTATTAACTTGTTAGAATACACAGCAAGTAAAGAACTATTTATTAAAGCTTTTGTTATTTATCTGTACTATTTGATAGAAAAAAACATCAATAGCAACATTTATTATACACTAATTCAAGGGATAATTAATCCAAATAAAATAATTCTGTATCTCTTACACGATTTATCAGCATCTGAATGCAATCAAGATATAATTCGAATACGCATTTTAAGAGATATTTTAACAAATATAAATGTAATTAAAATTATTCGTCGAGGAACAAATAAATTATTTGGAAGCGATCCATTAAACATTGAAATGGTTATCAATGAAGTATTATCAAACAAGCTTCAATTAAATGACAAATCATTTATTAAATTATGTCTGAACTGCTATTACATCTTGAACATTAATAGTATTAATATTAAACCAATAGGGGTAGACATTGAAACAATTGTTACTAATTATAAGTCAGATATGGATATAGTTAAATATACATTACATTTGTTAAGCATTGATTATATTCTTTTTAATTTGATGGAGTATTATAAAATGATTTTGAAGGAAATTAATGTCAAGGATATTTTTAATGAAGCACAAAAAATCGAATTTTACAATAAGCTTATTAATATGGGAGAAGAGATTATTCGAATTAATCCAAATATTAACCATACATTTCATATAATTAACATTTGGAAAGAGTCATATAAATATTTATATACTAGTACGTTTGTTCGAAAAATAGATCTGAATCTATTTTATTGCAAAGTTATGTTGTATTTTATAAGTCAAACAATATTAAAAACAGAAATTCAATTAAAAGGAAGAACACTATTATTCAATCAGGACTTTACTAAATTCAATTATGCATCCAATGATGTGGTAAATATTAGTGACGATAATACCAATTTAGCTTCTGATGGTTATTCTATCCAAATAAGCTGTAAAGATGATTTCAAAAATATTGTTGATTTCAATTTAATTATGTTCAAAAACAATCCAACACAGTTTGTCAATGCTCTTCCAAATGATTTGAGTGTTTTACCGTTATTTTATAACTTCAATGATCAATTCAAAGGACGATTATTCAAGGAATTCTATAATCGATATAACTCATATTTATTATTTATGTTATATACCGACTTTGATATCATACAAATGAACAGAAAGTATAGTCAATCACTCAGTGAAATGGATATGTTGGCATTGTTACGATTATCCATGAAAAGTGTAGATAGTGTTACAATGAATACTATAATTAATGGTATGAACTTAATATTTAATGAAAATATATTTAAAATGTTTTATTGTGTTATATTAAATAATATGAAATATGGTAAAAGATTTCGTGACTATTTATTTTTAAAATTAACAAAAGATAAAAAGATTTACAATTTAATATGTGAAGTTGTACTAATTTCGAATGAAATTGACGAGGATAGAAAACAAATTGAATTTAAAGATGTGCTAATCGATATGATCAATCACTTGTATGAATATCATAAAAAATCTGTTACTTATCATATCCATCGAAAAAATCTTGCTATATTATTTACAGATTCTATTATTGATAACTTTTTTAGTGATGCATTTTTTTCTCATAGTTTATTAAAAAAAAAACTAATTTTCAAAACACTAGGGCCATATATAAACTACAACCAAGTGTTATCAAGCATTTTTAGTAACTTAGAAACATCATTTTTCAGTAAAAATATTACACAAAAAATCTTTAATATCCGAAGATTAGCGTTTATATTTTATACAAATAGTAATTATATTGAAACACGATGGGTTGATAAAATATTGACATGTATTTATGAATTAATAAATTCTAAAATTGAATTAAAAATAGAGATATATAAATTGTTGCGAATATTACTATTTAAAGTGAATGAACGAAATATTAATATAATTCATCCAGTAATATTTGAAGACATGCTATTAAGCATAAGGTTGAATGATTTCAAATTAAATTATGCAATACTTAGTTGTATCGATATTAGTGAGTTTATGGAGTTGCAAGAAATGATATATGAAGTGGTATTAGATGAATTTAATAAAATTTATAAAAATGATACAAAAACATTAAATCTCAGTGTTGAAGATAATCCAAAGGAATATGGACCATTATTTGATTACAAAGCTAATACTATGGCAAATTTAAATGAATATGCATGTAAAGCATTAATATACTATGAAAAAAATCGCAAAACACTAAAAAGAAAATCTATTAAAACCCTCGAAAATTTACTTTTAACACAATTTGAAGAGACTGAATATAAATAATAGTTAACAAATATTTTAAAAATTTTTGCTAGCCCCCATTATTGTGATATGAGTAAAGGAACGCCTAGCATGGGAAAACGAATTAAGAGAAATCATTTATTGTGTCCTCGATGTGGACATATGTCGTATCACAAACAAAAGAAAATGTGCGCTGCATGTAGCTTCCCTAATCCTAAGAAAAGAAATAGAGGATCCCTTAAAGCTAAACGGCGAGAACATGGTAAAAAGAAATATATTAAACGAGTTATCAAAAAAAGCTTGACTGGATTTAAACAACATTTGTTAATTGAAATGATGAGAGAATAAACTAGTTTGTTTATAACTTACCATATTGCTACCTATTTAATAAATAATTCATACTATTTTTATACTCATTATTTTAAATACCCTCTATTTAATGGTTACTATTTGTTTGTTAAAATTTTGCTTATGTAATGAACAAATATTTATATCACGTCCTAAGCAATCACCATATATAACAACTAATAATATAGATGATCCAGAAGAGTTTCGATATTTACGATATAGTTCTCCAATTAAATTCAGTTTCGAACCATACCACTCATTAGTAAAAGAAGCAGATTATTTTAATACATTCAATGATAAAAATGTGTTTTTAAACCACTGTGTTAATTCTCAAAATTCGTTGCCACTTTATCAGCAGATTTCTGCTTTAATTAAGTGTCCAGAAATTCAATTTTCTATTCCCAGTACACAACATATATTAACTATATTTTGCAAACCAATTGAATATTTAAAAATAAAGAAAAATAACAAGATTATTACTTTAGGTCATTATAACAAATTTATTTATAAAAATAATGAACTTATTTTCATGTATAGAAACGGAGATACAACCCCGTTCCAAAAGAAATATTCTGGAACTGTAGTATTTTATCCTCATGATGAAAGACAATACAAAAGAATTTTTTTAGAAAAACAAGGACACTTTAAATTTAGAATTGGGCTACCTGAATTACTTGTGGAAAATAAACAAATAGGATTAGTGTCTTTAACACGCAATATGGCTGAGCGTATCAAAAATAATTGGAGCACAACTTTTCAATCAAATGTGCCTTTACAAAGTCAAAAAGAACATATAAACAAAGTATCCCAACTTAAAAAAACAACTATCACCAAATCTCAAAATTTAGTCAATAATTTCATTAACAAACATAAAATTTTCTCAAATAATAATTTTAACAATCACAAATTCTCCACAATTAAAAATAAAGATTTAATGAAATTTTTTGATAACAGTATAAAAACTCTTATTAGGAATACAAATATTAATAATGTACCATCCAGTTCTCATACAAAACAAAAAAAAGCATCAAAAAAAAAATCTAATGATAATTTAAACACAACTTCATCCTCTGAAAATTTTAATTATAGCAATAGTATTCTTTAAATTCAAAATGTTATATTATTAGATAAAATTTTTATTTATTTATGGTTCATGTATTTTTATGATAGTTCATTTATATTGAGTTTGCGACTAAACCTTGCCTATACGTATCAATCTTTTTCTTAAGCATTATTAATTCTGTTTGAATAATTTCCATTTCTTTTTTTATTTTATTTATGCTATGAGCATTTATCTGACGTTGTAATTTATATAACTTAGACAACAGTAATTTGTTGTGAAGTTTAAGCTTATAATTTTTAGTTTTGGCATGCTTAAAATGTATTATAGATGGATCTATCGAGTTTTTGTCAGAATGATTTAAAAGTATAGTTTTCAATTTATTATATAGTATATCAAAATCTAATTTAAATTGACGTGTAAAAAATACTTGAAACATTTTAATATTAGATGTTGATTTTTCCATATATATTGAATTAATACTTTTTACAATACTCTTTTTAAAGTTTAATAATTCATTCCAAATGTTAATTTGAGAAGAATTAATAGGTAGTGCACAGACTTGAGCCTTATTCTTTTTTGATTGACTGATATAAGATGCAGGTGTTATAATTTCTCTTATTTTATTGTAAATTTTAGTTGTGGCAGTTTCTAATACACTTACATTACATTCATATGTATCTTCCATGAAATCGATATTTTTAAACATATTTTTTTCGAAGTCATCTCTTAAGTTATTTTCTAACTCCGACAAATTAGCAGTCGTAGTATTCATTAACGTTACTTTGTTCTTGGTATTCATGAAAAGGGTTAATCACTATTTGTTTACATTGTTAGATAAATATAAATATATACAGAAATTGTTTTCACAACTATTATATTATTGTAATGCGTGTTAATATCATGCATGTATCAACCAATTACTTTCTTAAATAGTATTTTTATTTAGGGGTCATATACCCTTTTAACTTATACTTTTTAGATTTTTCATTCCCAATTTCTGGAAAGTTAATTGTTCCTTCGATATAAATAGGTGGATTAGCTTGATTTTCTGGAAGCTGTTTCATTACGTTAATTATGTCACCAACTTTATTTTCATTCGCCGGTTTATCATTAGCACTATTTTTATCAATGTCTTTTTTTATGTCATTAGATGAAGGCATCTGGATTTTTATATATGGAAAGTCTTTTGTTATTAATACCTTACTTTTATTTTCTGACTTATTTGCACTAGGTTCATCTGACTTAGGTGACTGTACATTTTTATTATCATCACTATTAGTACTATTTTTATCATCATTATATTTCTTTTTAATTTCTTCTTTTTTCATTTTGAATTTATCCTTTGTATCATTATATTTTTTTTTCAATTCAGCTTTCTTATCCTTGTTATCTTCTCGGGATAAATCAATATCAATTTTAGATAAAGCAACAATTTCATTTAACTTGTTTTTTTTGAGTTCAATTGCTTTTTCAATTTGTTTTTTATCCTTAGATGAAGATTTGTTTTTACTCTCATTTTTCAACTGTGCAATTTGGTCTTCAAGTTTTGCCTTTTCCATTCCAACCTTTTGTTTAATTTTTTTCACTTTATCCTCTGTTTTGTCCTTAATTTCATTTTCTAATTGTTTTTCTTGAGATTTTCGTTGCTCTTCAATTACTTTATTTTTTTCATTTTCGTTATTTGCTAATTTCATTTTCATTTTCATTTCTTCATTTTTTATTTCAATTTCAATCTTAGTAATTTTATCCCGCGCATCATTTTTTAAATTTTCTTCTTCTTTTTTAATTTCATACTCATGCTTGTTTCCATTAGGATCATCCTTCTTTATCTGATCTTTTTTGTTTTTTAATTCCTTTAATTTTTTTTCTAACTCATCATTAATTTTATTTTTTTGTTCTTCCAATGTCTTTTTTTTCTCTTCAAACTCAATCTTTAAATTATTTTTTTCATTTTTAGATTGAATGTCAGATTTTTCATTTTTATTGTCTGTATCATTATTTGTCTGTTTTGAATCATATTTTTTATTATTTTGTGATTCGGAAGTTTTAGCCGTATCAGACTTTTGTATTTTGTTCAAATCATTCATATCAATAATTGTAATTGTTTTTAATTTAACAACGGTTTTTTTTTTATCTGGTCCAACATATAGAGTTGTATATGTAGATTCTCCTCTATCAAAAATATTTTTATCATTTATTGCTTTGGTTTTTAAATTAATTTCATTTGATTTTGCCGTTATTACAGGATTATTATTGGGGTTTTCAATCGATTTATTTGTTTCTATTATCATAGGCATCTTCTGGTTTACAAAAGGATCATCAGCACACTTAATTATTTCTATAAATGTACAGGACAATAAGTTATAAAGCATTCAAAATAATATTGTTACAAATTTATCCAAAATCTGTAACCATGTTTTAATATTTAAACAAACAATATTGGCAATATAAGTTTATTCACCAGTTTTGGCTTTGATTATTTCACATTTTAATGATTCACATGGATTAGCACAATTACCCTCCAGTTCACAAGGACAATCGGGCTTACACTCGATTCCTTGTTCCTCATCATTAGTTGTTTCGACAACAATTGTTTTGTTAACGGTAACATATTTGGTATCATCATTAGAATCATCTTCTATCTTATCGTTAGTTTTTTTACTAAAGCTATCACACTTTGTAGTATTTTCAATGGTACAAGGATTTGTAGATTTATTCACAGTCATTGTTTTAACAACTTCAACAGTTGCTGTTTTGGTATTACAAATCGTCTCAGTTTTAGTTTTCACATCATGAATCACTGCCGTTTCTGTCTTTGTATTACATATAGTTGCTGTCTTTGTTTTAAACACGGGTTGTATTTCAGTAGCAGTTAGTGTTTTAACCACAGTTGAAATTGAAATTGGAGGTTCTTCTGACGGAGTGGTAATACAGGTTTTTTTTGGTGACAAACATGTGCCTTTGTTTTTAACCGGAGTATTGGAAATGTATTTTTCTGAACTGCATGCATTCAAGCCACCATATGTATTATTGTGTGATGGTTTTGATTGTAATTTGTTTTTTTTACATTCATTTTTTAAATCATATTTTAGTTTAATAGATTTAAACTGAATTTGCTTATCATTTGAAGAGGAATTGATGTTTGTCGATGTGTCAATTGGTTGAGAAACAATGGTTGAAGGAGTAGAATTGTTAACTATTAGGGAATTAGGATTGATTGACGCAGTACTATTATTTATAGATGAATTAGAACCATTAGAAATATTAGATATAACATTATTACTGGTAGATACGTTGGGACTGTTAGAATTATTAGAAATAGCATTAGTATTTGATGATAGATTTGAAGTATTAGAATTAGAAGAAACAACATTATTGGCTGTTGCTAAGTTTGAATTATTCGAAATATTGGTATTAGATGTAGCATTATTTGTAGATGCTGTACTAGCATTGGCTGATATAGACACCGGGTTATTTATATTATTAATAGAATTAGAAAGTGAGCTAGTATTAGATTGAGACATTGAATTTACTGCTTGAATTGTTGATGGAATAGTATCTCGAGATACTAATGATGCAGATACTGCTATTTTATCCATTAAGTTTTTTTGCTTTTGACAATCAGATTCTTGAACTGTAGGATCTTGAATTAACCCAGTAACCGTTAATTTTCCTTTATCTGGAATCTCATGTTTTGCACATCCACAATCCTTTCCCTTAAATGCACCTGATTTATCAACCAGTTGTCCATTGACCATTTGAAACTCAATATTCCCACTATAATCTTTATAAACATCTCCATCTTTATATACAAGAATATCACTAATTTGTTTACAATCTTTACAATCATGTAAAATGGATGATACTTTGGATGGATCGACATTACTAATACTTTTTATAGTGCTTGGTGGGATAATAGATGAAATACTTGCTGATGATATACCATTTATCTGCGATGATATAGTGCCTGCAGCTATCTTTCCAGAACTTGAACTTATAGATAAAGAATTTACTTTTGAATTATTTGTTGACATACTGTTAGCTTTTACAGTAGAATTTTGATTATTCACACTACGTGTAATAGAACTACTTGGGGTGATTGATCGTTGATTGTTATTATTGATCATTGATGTATTACTACCTGTGCTTGTTATTTTAGATACGGAGTTATCATTTTTATTGTTGTTCATTATCGCACCAGCCGCTATTCCACCAGCTGCACCGGCAACTCCTGCTGCAATAGCAGTAGTAGCAGTATTATCTTTAGATATTGTTTTATCAGGTTCTTTATCACTTTGTTTTTTATTATTTTCTTCTTGTTTTTTACCATTAGTTTGATTTGTAGTATCATTTTGATTTTTATTGCCATTTTGTTTCTTATTGTCCTGCTCTTTGGTAGATTGAGATTTGTTCTCGTTAGATTTCTTGCTTTCTTCTTGTTTTTTATCGTTATCATTCTTTTTTGTATCATTATTTTTTTTATCTGGATCTGATTTCTTGGTACTATTCGTTTTTTTTGCTCCATCTTGTTTTTTATTATTATCTGATGTTTTTGAAGCATCTAACTTTTTATTACCATCTGCCTTTTTTATGTCATTTTGTTTTTTGGTGTTATCTTGTTTTTTATTAGGAGTTTGCTTTGCATCCTGTTTTTTATTATTATCTGTTTTTTTTGTAGCATTATTAGCACTTTGTTTTGAATTATTTTGCTTTTTAGTATTATCAGCCTTATTTGGTGTAGTTGATTTTTGTGTAGATTCTAATTTCTTATTATTAGTTTGTGATTTATTTGCGGTTGGTTTTTTATTATTATCTTGCTTAGTACTAGTTTCAGCCTTTTTAGCAGTACTTCCGGTACTTTGCTTAGCAGTATTTGAAGTTCCTGATGCAGATGATACATAAGATTTTTGAAAGTTAAATCCTAAATCTGTTGCTCGTTCTATTATCTGGGATAACAATGATTTAGAAGAATCTATTTCTTTTAAATTTATTACAAACTCCTTTTTTTTATTAAACTCTTTTTCACTTGTTATACTCAACAATATATTGATATGTAAGACAAGTAATGGTACAATTTTCATTATGTTATTTATAGCATTAGAATAAAAATAAATCAACTCAAAACAACATGTTTTAAATCAATAATATATCAGACTATTAATATGTATTTATACGACCCCTTTCAACATTGATTAACCGAAATGGATGATAAAATAACAATTCAGTTTGTTGGTAAGCGGAAGATACCGATTATGCATCGGTATTTGTTTACTATTTGTACACTTGGGATATATTACGTTATTTGCAAAATAAGTAAACGTATGAAAATATGGTTAACATACACAAGTAGTACATTATATAATGCTACACACATTTTGATTATTGATCAATTTCGTAATTCTAAATTATATGCAATAAAACACATTACATTAAATCCTAACCATCAATACCAGTTGGCTAATTCTATCATATTAAAGTGTTTTTTTAATACAAAATCAAACAGTATAAAAATATTAGATACTCCTTATGGAAGATTTATATTTGATATACAACTAGACAAATTTGTTATTCCAGATTATCCTATGCAGATAATAATGCAAATGTCACATTTGGAAAAAACAATTATTTTTGGAAATAACGTTATGGATGAAATTAAAGCTAGGAATTTAATTGAAATTATTCAAAAAAATTTTTTTAAGTTGACATTTTTATGGGAAATATTTGCTATATATATTTGGTTTATTCTTAAATTTTGGCGTTATGTAGCTTTTGTAGGTAGTCTGTATCTTTTTCTTTTTATAAAAAATATTTATGATGAATATATTATAAATACAAAAATTCTTTTGCAAAAACTCAAAGACGATCAAATAAAAATATATAAGCAATATGGATCTGAAGATATTCAGAAATTTGAATACCAACAAATTAGTTATAAATACATTTATCCAGGAGATATTATTTTAATCGAAAAAAATAGTGAGATAAAATGTGATATTGAAATTGTAGATGGAAATGTTATTGTTGATGAATCATTTTTAACTGGTGAATCTATTCCAATCTGTAAAAAACGTGGAGAAATTTTATACGCGGGAACAAAAGTAATCCATAGTTCTTCTGAAAAAACAGAAACAGTATTGTATGATGCTTCATTAACTAAATTAATTCGAATAAAAAATTTATTGCACAATACTAGAAAATATATTCCTAATACAACACCATTTAAAAACAATAAAACCATTCCAACTAATTTTCAAACTGAAAAAGCAAAAGGAATTGTTTTAAAAACAGGAAAAAAAACCAAACAAGCGCAATTAATTAGAAACATGATTATTAAATCCCAATTAAATGATAAATTCAGAATAAAATGTTTTAGTATTATGATTTTACTTTGGATATTTAGTATAGCACTCTGTATTGGAATATATTTTTATTTAAAAAGATATATTTCATATGTGAAAGCCATTGTATATAGTATTGATTTGTTTATGAGCATTATTTCCCCAGATTCAATCATTACAATGGAATTTAAAATATTAAAATCTATTTCAGAATTAAAAAAACATGGCATAACTTGTAATGATAAAAATTGTATTATGGTTGCAGGTAATGTGGATCTTTGTATATTTGATAAGACTGGAACATTAACAGAAACAGAACTTGAGGTACAATACATTGATTTAATATTATTTCGTGTCACTACGTTTGATGAGTTAGCCTCTCACCACCTTTTGCGCTTGTGTATGGCTACTTGTCACAACATTGTTGAATTGGATGGAGAATATAATGGGGACATATTGGACATGAAAATGTTCCTGTTTTCCAAGTCAGTTATTTATTATAATAAAAAGCAGAGAATCGTCAAAATGCTTCCATCCAATTGTATGGACGAGACATTGGTGAAGATAAAAGAATATAAAGAAAACAATACATACCCAAACAAAGAAAATGACAACTATGATTTATTTATTAGTAAAAAAGAAGATATGTGCAAAATTACTTTTAATCAATCTTATGAACTCAAGTTAGATGAATATGCAATAGTGCAAATATACGAATTCAATTCGATACTGAAATATATGTCTGTGGTTGTTAGAAGCAAGACTACTGGAAAATATTTTGTTTTTTGTAAAGGTGCTGCCGAGATATTAAGAGATAAGTTAAAGAGTATTCCTGAAGAGTATATGAATAAGAATCATGACTTTGGAATTAAAGGACATAGAGTAATAGTCATGTGCTACAAAGAGATAGACTCATTTGATCCTAACATGCAGAGAGAGTCCATTGAATACGATTTAGTTTTTCTAGGATTTTTGGTGTTAGCAAACAACTTAAAGAAAGAAACCCGGGAAGTGATAACGGCACTTAACCAAGCAAATATAAAGAGCAAAATGTGTACGGGGGATTCTATATTAACAGCCATTTCTGTTGCTAGAGAGTCACACATGGTTTCCTTTGACCAGCCGATTATTTTTCCAACAATTATAAAAGATAAATTTCCCGATATGAAGGAGCCTAAGAAATTTTTTGATGATAAACAATCAACTTTTGATATCGAATGGATATGTGTTGGAGAAGACGAATACTTTTTTGACAAAACGAAATTCAAGCTCTATTCAGAATTCAATAATTATAACGAAATCGACTTTGCAATTGCCATAGAAATGAATGAGTATATCGAATTAATGAAAATTGACTATTACAAAAAACTTATTCTTGAAAAAGGTGTTGTATTTGCCCGATTCACTCCAGATCTTAAAAAGAATTTGGTTGAACAATATACTTCGCACACCACATTATTTTGTGGTGATGGTGCCAATGATATTGGTGCACTTTCGTCGGCAAATATTGGATTATTACTTTCATCCAATACAAACGGAGGCTTCACATCATTTTCCTCGTTGCATTTGAAGGCCATTATTAGTTTGATTAATGAGGGAAAGGGTTCACTAGCAGTGGGAATTTCACAATTTAAATTTGTTCTGTATTCACAGATATTAACTGGAATACAAATGTTAGCTCTACTTTTGCGTCAGCATTTTCCAAGCGATTCATGTAGCCTTATCATTGATCTCGTAAGTTGCTATGGATTAGGAAATGCATTAATATTTTTTAACAATCCCCACAGACTTACAAAACAACGTCCTCAAATCAATCTTTATACCCAGGTTATATACATGATAATGGAACTACTATTAATATTAGGTGTTTTATGTGCAGGGACATTATACTTTTTAACACCTAACATGGTGCTGAGAAAATTATCTGAATGTCAAAACCCCAGTGAAAATCATATTATCGAGAATGGGAATCTCGCATCAGTAACATTTTTTATTATTATTTGGTTGATCATTTTAAAAGCATTCTATTTTGCAAACTATGGAGAATTCAAAGAATCGAAGTATTCTAACAAGAACTTCATAATTGCAATTGTATGTTTGAGTATATCCCTAGTAATTATCTTTGGACTTAACATAACGAAAAACTCGATAGTATGCAGATATTTGGATATAACCAGCCTAACTCCATATGAAATATTAGTATTTTGTGGAATGCTTACTGCAACAACAGGAATAACCATGTTTTTTAATTATTCTGTAAATAATATTGACAGTACTAATTACATTGTATAATTTTTACCCTATGGGATTTATCAAATTACATAATTACATTAAAAATCGTCAGTTTCCCATTATAATTCAAAATAAAGTGGAAGCTGATAAATTAGGAATGTGTATATATTATTTAAATAGATTAGAAATGCCATATCAACTAATTGATATTAGTAAAGTAAAAGAGCAACACAAACCTCTTATACCACAACTATATATAATACACAATATTGATGATTACAATATTTTAGAAAAATTATCATACACAAATAATATAATCATTGTTAGTGAGGTATTTATTAATATAAAGACTCAGAATATATTTATGGTTTCACTGCCATCTAATTTTGGGCATATTAATAAGTGGCCAATATATCATGTTACAACTAAAGTTTTAACCGAAGTCAACAAATTAACAATAACCATGTTGTTAAAAAAAATTTTTAACAAAAAATGTACAGGTATCGAAATAGCAATTGATAATAACAAATTTATAATTGTTCTAAAAAATATAATAAATTCAAAAATGTATGGAAATTTATTTTTTATAAAATCTAATAATATATCTGATGAATATCACATATGTAAAACATTTAGTGTTAATAATGTTTTAGAAGGTATATATAACTGTTTTTTAGATTTTATAACTGTACAAACTTTTGTTGAATTTTATGACATTCTATCTCTTATTGATTTAAATATTAATCAATTAATAACATTTTGCATTTATTCAAAGATAAACAGTAATGTAATAAAACATAATGGATCACTTATAAGAATTAAATATTCATTACATTATGATACTAATGAATAAACTTGACATTAATCAATCTGCCCATTGAAAGAATTTACACTTTTTATATGCCTTATTACATAAATAAAACTTTCTACCTTTATTATTAGTTGTATTGGATATTTTTTGTATAACAGGTTTGCCACAATCACACGTTATGGTATCGATAATTTCGTTATATCTAACTTTTTTTAGAGGTATTTTATCTCCTTCCCATATAAAAAAATCACATTTTATTGGAAAAGTATAGCAACTATAAAACCATTTTCCTCGGTTTTTTCCCTTACTAACTTGTTTTTTTACTGCTATTTGTTTACACCAACATAAAATATTAGAAGATGTGTTATTAGTATGATTAGAGGCAAGTGAATATTTATTGTATAATTCCGAAGTGTTTTTTTCAACATTTGTTTGCATAATGTTAACAAAATCATTTATATTCTGTTGGAATTTGTCAAACATTCGTGAGTAAATATCTATCTGTTCTTTTACTAATGCATTTTTTGATAATTGACCACTACAGATACGTTTAAGATTTTTTTCCATATCTTTTCTAATAGAGCATTCAGACAATGGTAATTTTAATTGACTATAAGCATTAATCAAATTAATTCCTACAATTGTAGGCTTAATATATTGTTTCTGTTTAATAGCATAGTTTCGAATTTGTATTTTCTCTATATGCTCATGAATAGTTGCATCTGTTCCAATTTCATTTTTGTCCATTAAGGTAATTAAATCTTTTTCTGTTAAATATTCGGGACATGTTGTCATGCCATCTTTTATCTCTATATTTGCCTCTTGAATAATATCATTAACATTATATTTTCCAATAATTTCCTTAGCATTCCATTTATCATAAAAATAAATGTTTAAGTAATTTCTTTCTAAAATAATTAAACCTTTACAGTAAAAATTTTCTTGTTCAATATTTTTTTGTAGTATATTTGAAGATTTCGGAACAAATGGACGTATTTTGATAACAACTTCAACACCTTTAGCATTATCATCACAACATGCTATAAATCGTCTTGCTATAAAATCATATATATTTCGTTCTTTGTCTTTAAGCATATTTCCATCTTTTAAAGGATATATTGGACTATGTGCCTGATCATTGTTATTACCATTACGTGGGAATTTCAATGTAAATTTTTCTAAATACTCTTTAAATTGAATATCATTTCGCAGTTTACCAATAATTCTGTTGAAATCAAAATTATTTGGAAACACATCAGTTTCTGTTCGTGGATAACTTATATATCCTTGATTATATAACTTTTCGGCTATGTCCATTATTTCATGACTGCTTATTTTGTAATATGTCGTACATACTTTTTGTAATTCTACTGTTCTTAACGGTAATGGTTTATATTTTACTTTGTTTTCTCTTATAATATCAGTTACTATAAAGTTTGCACTTATTAATTGATTATAAAAATGTAAAACACAGTTTTTATCAAAGATATGCCCGCGATTCCATAAAAATTCATTAGATTTATTTATAATACTTATTGAATAAAATTTTTCTGGAATAAAAGAAACAATTTCTTTGTGTCTTTGAACGACAAAATTTAATGTTGGTATTTGACATTGGCCATATGAAATAATTTTATGAGCAGATAAAGAATTGAAAAACAGTGTTTTTTGAAGCGCAAGTGTTTGCAATACTGTAAAGGCACATCCAATACGTAAATCAATTTCTTGACGTGCTTCAACTGCATCAGATTCATACTGATTAATATCACACAAGTTATCAAAGGCTTTTAAGATTTCATGTTTAGATATAGCAGAAAATCGAGCTCGTTTAACTTTTTTGTTGTAAACTAATGATTGAATTTGCTTTCCAATATTTTCACCTTCTCGATCACAATCAGTCCATATTACTACTAGATCTGCATTAATTGATAGATCTTCTATATTTTTTTTAATATATATTGATTCAGTTTTTAGTTTTTTGTATATTTTAGCAGTAAATAAATTCTTGGGATTTATGGTATTCCAGTTCTTATATTCTGATGGAAATTCAAATTCATACATATGTCCCAGTACACTAGTAAATATCATGTTGTTATTTTTAAAAATAAATTTGTGATTTAAACAATACTTGTTTTGACCTTTATATACTTCTACAGAATTACTAAAGATATTAGCAATGGATTTAGCTACAGAAGGTTTTTCTGCAACATTCACTATTAACATTTGAAAGGGATAAATAGTAAAAAGAATTAACCCTATGTTTAATGTAATTTTGCCAACATACAATGAAGCACAAAATATTTTTGTTTTAATAAAGATGCTAGAAGATATTTTTACTCAGCTCAAATATAATTATTTGATAATTATAGTTGATGATAATTCTGAAGATGGAACAAGAGATATAATTAAAAATATACATATTCCTAACATTAAGTTAATTGAACGTCCTACAAAATTAGGATTAGGTAGTGCATACAAGTCAGCATTGGTACATTGTATTTATGAATTTACTATTATTTTAGATGCAGATTTACAACACAATCCATTTGATATAATAGGAATGCATAAATATTGTAATCAATATGATATAATAGCTTCTACACGATATAATAAAATAACAATTGAATTAACAAACAATAAATATATTGATATACAGGGAAAAGTATATAATTGGTCATTAATACGAAAGTTTATTTCTTCATTCTCTAATACTCTTGTTCAATTTGTACTTGATTTAAAAACTAGTGATGTAACTTCAAGTTTTAGAATTTATAAAACAATGGTTTTGCAAGATTTAATATCTCAAGTAAGAAATAATGGATTTGGATTTCAAATGGAAATTATTGCACGAGCAGAGCATAACAATTATACAATCAAAGAGTATCCAATTACATTTTATAGTCGAATTCATGGTAGCTCTAAATTATGTTTAACTGAAATTTTCCAATTTATTGTTATTTTATTACAGCTATATTTTGTTATCTAATTTATAAATAAATATAGAATAAATCCGTTAAATTCATTGTTGGTGTATAATTAATTAAATATGTAATTAATCTAGTGTTTAATTCATAGTTCATTTTATAAATTATTTCATCTAATAAAAATCGGAATTCACGTATAGATTTAACAATTCGAATATTATTTTTTTGTACTAAATATCTCATTAAACTCAAATTTGTTGGTTCATCATAAAATTCTTTCAACTTTTGTAAAATTATTTTATATGGCTCATTAGCTAATATATATTTGTTGTTTACTTCAATTATAATATTTTCTGTCGGAGCAAGAATAAATTTGAAACTAGAATTATACAGTATGCATGAATGTGATTGAAAAGAGTTAATTATGCATGTATCATCAAAAAACAATAGATTTTTTTTTATTTTATATGTATTTGTAACATATCTCATTATATCAATAGCTTGTGCTAATAAATTATATTTTTTGCTTTTAAACTTATTTGTCAAAAATAATAACCATAATGAAGCCAACTCATATACACCTTGCTTATATAATTTTTTAACCAACGTTAACATAATTTTTTCATTCCACATATATTTTTGAATGAGTTTTAAAAATATTTTGTCAGAAATAGTATTTATTTCAATATATGTACAAATTTTATAAGTTAACTCACTAAAATTATCCATAATGGTAAAATATTTTGAAAATTAATTTATTAAACAAATATCACAAATCAGTGAACCTTCGTCTGTTACTACAAATAATTCTTCTCCAGAATCGGACAATTGCATATTAATAATGTTTTGATTAATATTTATAACTTTAATGGCATTCGAGCGCATATCTAAAAAATTGATTGTTGAATTTATTGTCTTGTTCATTGTTGTAAAAGCAACAAAATGTTTTGAATATGTTGCACAAGAAATATTACTTGATGGGGCAGTATATAGAGTAGTTGCGGTTTGCATATGTAAATTAAATTTTTGCAATACCTCATTCCCAAAGCCAATAATATGATCTGAACCATATAATGTGACATGATTTGTACAATTGAAGTCATAAATATTATGCGTAGGATTTTCTATGTTTATATTCAAAACACCTAATTTACCAGCTGTATCGCTTGATATAATATATGAATTATATGAAAATTGTGTGATATTGTTTTTATGTACTGAATATGTCATAGTGGAATCTTTATCTGTGACTATAATATCATTTTCACAAACGCAAGCAAATTGATTATCACTAATATATTCTGCAAAAGTAATACGTCTGTTAGAAATTTGAATTTTTCTGTCTAAATAAATCGATCCATCATCCGAAGCACTCAAAATCTTTTTATAATTTGGATTTGATGTCTGAATACATTTTACAACAGTAACAGATTGTGTGTGTCTATTAGTTGTATTTATTGATCTTGACAGAGGGTCAATAATAGCAATATCTCCATTTGTTGTTCCAGCAACTATATATGATGCACACGCATCTAAAGAAGTTGTTGAATGTATTTCTAAATCAAACATAAATTTGTTTCTGAAATATGCATGTAATCTATTATTATCAGTGATAAATGCCAAATTATTATTAGACCAAGCAGAAAATAAGGATTTCCCGTGATGCTTTAATAGATTAATTTCTATCGAAATACTATCCACATCGCATTGATGATTTATTTCTTGTTCAATTGATTTTTTAAAACTGCTTTTACATTCTAAAAAAACATTGTTCTTATAATGTAAGTCACCATAAATATATTTTAATCCGCATTCTACTAAATATTCTAAAGTATAATTTCCAATTTTATTATTTGACAATAAATCTTGGTTTTTAATAATTTCTTCTTTGCACTTTTTAATTAATTTCTTGATATCATTTTCTGTCAAATCAAACATCCTTTGAATAGGGTTTTTAAAAATTTATAAAATACATCCTTCTTTAATTACTCACATAGCATAATTTTTGTATCCCTATATGTCAAATTCAAGTAATGATAGTGATTATAATACAAATATTAAAAAAAAAACTAAAAAACAGACCAAAGTAGTAAAAAACAATATCAAAACATCAGAGTACAAACAATATCGGAATGATATTTATACCCTGTTATATTATTCCAACAAACCATTTATTATAACTGAGTTAAATTTACAATTTAAACATATCCCTAAATCACAAATTGAACAGATTCTACTTGATTTGAGTCAAAAACAATTAATAACCACTAAACTTAATGGAAAAACAAAATTATATTTTTTAAATCAAGAAGTACTTAATTATTATCACGAGCAATTTGATCAAATGGAAACAAATAAAGTTAATGAATCGAATATTACTTTAAAAAAAACGCTAAGTCAATTAAAAGAAACTCATACAAATATAATGGCGAAACTAGCAGAATATAAGCTCCAAAATGAACAACTACATGAAAAATTACTTAAAGCAAAAAATGAGCTTAACAATGAAGAATTAATTAAATGCATTTCTGATTTTAAAAACTTTATAAATGATAATGAAAAATATAGTAATATTATAACTATTGATAAAAATTTATTTGAGACTAAACAATTAAAAGTTATCGAATATAAAAAAATCGAAAAACAACGACAAATAATGTTTAAATCTATTGTAAATACAGTATGCGAGAATTTTGAACTTAAAATGAAAGAGTTATTAGATGAAGCTGGTATTGACCAATAGCACAATTCTATCAGTCTGAAAATAATTTTTTTAACTTTTCATTTGATTGTATTTGTGGACTACATTGAATATAATATTTTTTTTTAAATATGGCAATTCCAGTCTCATTTTGATCACAGATGTTAAATATCAAATCTATTGTCACGGGATTAGTGTAATCCAACATACTGAATAAATTATGCTTTATTAAAATATTCTGGACAGCACTATTATAGTGTGAAATATCAGTAAAAATCATTATAACATCATTATACAGTTGTGGAAATTTGAGATATGGAATTAGCTCATGCAAACGACATAATATTTTATTATTGTACTGAGATCCGTCCATAAGTAAAATCATGTGAATTTCATGCATAATTTGTTTTAATGACACAATATTTTGTGAAGACTGTTGTTTTAAATCACTAAGAGCATTTTCTAATGCAATAGCAGCTGGATTGGCTAAAATATCAAGTGTTTCTTTAGGAATTGGTTTAAATTCTTTATTATCTTCTGTTGGTATTTCATTAGACAAAAGTTTGTTATTTAATTCTGTATTCTCTGTCATAGGGATACAAAATATATTTTTATTTTTATATAATTTTTTTTACAGATAATTTAATGATTAAATTATTATTAATAAATATTGATGTACAAGAATAATTTTCTTTATATTTCGTTACCCTCACATATGAATGCTATTGTTCGACTTAAAAATGAATATAAAAATTTACAAAAAAAACTACCATATGGATGTTATATTAATCCAGTAAACAATAATTGGTATGTATGGGAAGGTCAAATTAGTATAAAGCATAACTATTTTAAAATTAGATTGGAATTTAATGCTGAATATCCGATTAATCCTCCTACTGTTAAATTTATATCAAAGGTGTATAATCCTAATGTTTATTCAACTGGAGATGTGTGTCTAGATATTGTAAATAATAAGTGGCAAGCATCTCTTACTATTTTAGATATTATTTTTGGATTAATTCAATTGCTGGAATATCCAAATCCTAGCAATGCTGCTAATATAATTGCTGGTGAATTATTTTTAAATAACAAAATGGAATATTTAAAACAGGTTGAACAGACAAATATATTTAATAAAAAATATAATATAGTAGGATATGTTGATAAAAATAAAAAATATAAATAATTTATTTACCAATTATTTATATCAATATGTGAGTTGTATTGCTGTAACATTTTTTCAGTCTCCACTGTATTAAATCCTTTAATTTCTGTTAATGGAACTAAATCAGTAGTAATGTAAGCTAATAATGGTACAGCTCTTAATTCATATAATTTAATAATTTCATTGAGAAGATCATTTGGCTGCGAAATATCTAAAGGAACAATTACAATTTCTTTATCTATCTGGTATTCTTCGAGGAATTTTTGCAATTGCTTGCATGGAGGACAAGTAGGTGAAGAAACTTTAATAATAACTGCACTAACACTTAATTGTTTTAGTTCTGCCTTAGTGGTTACTTTAATAAACTTAATACCTTTTCTCGCTGTTGTTTCTTTAACCATAAAGAGGGCTTTTAAAAAATATTTTTGATATAGAAATAAATACTAAAATAATATTTATTAATTTTTACTATCAATATACATGTGCTATACCAATATGATATTTTTTACAAAATTTATCCCTCATGGAAGAAATCGTAAGTAGTGATTTGGATATTAAAACATGTATTAAAACATGTTTAAAAACCGCCTATAGTCATGTTAAAGCTTTAACAGGTTCAAAAGAAATTGTAAAGAATTTGATTAGAAATGAAGAAATTAGTCTCGTAATTCTTTCAAAAGACTTAATTCAACAATATCAAGATCTTATCTTAATGTTGTGTAAAAATAATAATGTACCAGTTATCTTTTTAGAAAACAAAAGTGAATTGGCTGAGGTATTTCCTATAAAAGTTAAAAAGGCTAGTGCTATTGCAATTAAGAATTTTATTGGAGAAAGCCGAGAAAAAGCATTTCTAATGAATGTTATTAATAATTAAATATTTTAAACAATTGTTTTTTTGATATTAAATTCCATAGTCCCATGGAGATTTCTCTTTTAATCCAACGTTAGGAATTCGTTTTTCAATTCGTCTCATTGCCTCGTGAATATGAATTGGTAATAATGCACCTTTGTGATCAAATTCTTTTTGAACAGCTAATGCTTCAATAACTAGTTCTCCAATGAATACTTTTGCTAATCCACTTATACCGATAACTATATTTGGATTAACTGCTTGACCAATTACATTATTTATGTATTTTTTGATAGCACCTTTTACAAAATTACTACGTCGAAATGTTTCGTATCTTTGCTGTTCACTTGAATCCATATCGTTTACTGATTTCTGAAAATATGTATCCGTCTTTTGTTGGTATATAAAATTATCAGTACTAAAATTACTTTGTTCATCACTTGAAACATAATTCCAATCATTAGACCCGAATTTGTTATCATCTTCCTCATTGTCTTTATTGTCCATTATTAAAAGGATATAAAAATTTACACTTCTATGTTTCATTAAATATTTTTTGTACTAACTAATCGAGTTTTATATTATAATATATATTTTTATATTTTTGAAATAATTGGAGGCTGTTGAACTTTATAGTAATAATCCTTTCAAAGATATCTTGATTTAAAGCACAAGATATATAAATATTTTTCTTGGATGTTTTTATTTCTATAACAAAGTTAGAAACTGGATATATGGCGATAATATCATTATTATAAATAACATGACTATTTTTTAGCCAATAGTTATGAGATAATATATATATCACATCATGATTTTTTTTATGAATATTTTTAACTAAATCATCTAAATAATACATGGTAATGATTAACATAAATAAATTTTTAAATCTTTATTCTCCTTTAAACTTATTATTTTTTATGGGAAAGGTTACGAAAAAAACTTATATTTTGTTATTACTTCCAATAACTACAATTTTCTTCTTATGCTTGTTATTGTTAATTGATAAAAAGAAAATTCATAAACCTAAGGATATTATAAAAGATACTTTGCCAGATACTACATTATTAGATGTGCGGATAAATAAACTTAAAGATCTACTTAATAATGTATCATCAGAAGTAAATACATTGCCATCACTTATTATAGATAGTAGTGAGGATAACATTGATGTGAATATCAGCCAACAATCAACATTCACTACTACTGATAACGAAAAAGTAATAACATCTACATTCACTCAAACAGGTGCAACTCCATGGAATACTATTAAAATTATATTAAATAAAAATTTAAATCCAAATGCTATTTTAAATAGCATGACAATAGATAATTTTAAGCATACATTTAGTAATATGATTGAAAGTTTACTTATAATCAATATTGATGTAGCAAAACATATTAAACTTAGTGATAATTTACAAAATTTATTCAAAAATTCATCAGAACCGTCAAGTATTAATAAAGTTAAATTAAATAATGATCTTAAATTATTAAAGCAAGATATTCAATTATTATTGGAAAAAAAAAAGACAAACCCAGATATAAATATAACATTAACTATCACATTAACCACATTGCAATTGTTATTGTCTGTATTGTTAAACTTACCATTAAATGCTCAGGAATTGCCTATTAGTAGTAATTCAATTTCGTTTAACCATGGAATTAATATAGTTATTTATAATAGCATTCCAAGTAAATAAATATTAAAAATTTTTAAAGTCCATGACCAGTATTGATACAAGAATTGATAGTATTATAGAAAAATTACAAGAAATAGAGGATGATATTGACCAATTATTATCATTATCTAATGAATTAGCATATTTAGATGTAAACATTAATAAGTTTCAATATTGTTAAATATAAATTTTATTTTAATACCTATATTTACATGTCAAAAAAGCAAAATACAGAAACTCATAAAAAATTGAATGAAGGAGTCACAATATTTACACCAATAGAAGTTGAAAAAAAGGAAAAGAAAAATATATTTGCAACAAAGGATGTATCAGCCACGATGTCAAAAAATACATCACAGTTTTTAAACAAAAATTATGTTACCCATGATACTTTAGTTGATACTAAGCCAGTTCATAAAATAATGAATATTTTAACAAAAGAATTAATAACTATATATAATGTTAAAATGTTATATAAAGATGAGGAAATACTAGAATTAGCGGTTGGTACATTAGTTGTAAAACAAAAATGTTTTTTTTTTATTAGGCCTGGATTAGTAAATCCTCTATTATACTTTAATATAAATACTGTTCATTTTATATTAGATAACGAAAGTATTGAATTTGAATATAATGGTATTATCTACTTTATAAAAAATGTGGAATCTATTAAAACATTATTCACAATACTTAATGACATACAAAACAATTAGTAATAATCACTAATTCCATTAATAAAAATTAAAATTTTTACAATTGTTTTTTATTTCATATCAAAAAAAATTTGTCCTCTAAATTAGTAGTTATGACAATTTTTAATACAAGCAATTGTTTAGAAATTATTTATCAAAAAAGTGAATTTTTAGAAAAATTTCAGAAAAACAACATTATTTTTGTATATGGAGCTACTGGATGTGGAAAAACAACACAAATCCCACAGTTTATATATGAATACTATAAACTTCAAAACATACCATTTTTCATTGGAATGACTCAACCTAAACGTCTTTCAACCATCTTTTCAGCTTCTAGAATAGGTAGTGAAATAGCCAAAAAATTAAAGAAAGCAATAAAAAACGAAGTTGGATATACAATATTGTGTGAATCAACAGTTACACCAATGACCAAAATTAAAGTTATGACTGAAGGTATTCTGTTAAAAGAAATTTATTATGATTTTTTATTAACAAAATATAATGTTATTATTTTGGATGAAGTGCATGAAAGAAGTATTAATATAGATTTGTGTATAATGTATTTATCTGTAATTGTTCGAAAAAGAAGTGATCTAAAAATTATATTAATGAGTGCTACTATTGAATATACACGGTATTTAACAATATTCAATGACGTGCAGATCGATAGCTTTATAGTTAATACACAAAGTCATACTGTTAGTATATTTTATGAAGAAAAAACTCCTATACAATATCTTGATGTAATTCATAAAAAACTATTAAAGTTAATTCCTTTCAAAAAATCAATTCTTGTATTTTTACCATCTAAAAAAGATATTTATATTCTCAAATCTATGTTACATGATGTTTTTATGGATATAATTCCTTTACATTCTAATTTATCACAAAAAGACCAAAATAAAATTTATCAAAAAGCACCAAAAGTTATTTTAGCAACAAATATAGCTGAAACATCTATTACTATACCTGACGTTTACTACGTTATTGACTCAGGTTTAATGAAAAATAAGTATCACACATTAGATTGGATTGAATATAAACAAGAATTTATTACTAAAGCTAATGCCATCCAGCGACTAGGAAGAGTTGGAAGAACAGGTCCAGGAGTATGTTTTAGAATATATTCTGGTGTAACATATGAACATTTTCAAGATGAAATAAAACCAGAATCATCTAGAGGAGGCATTGAACAAATTTTATTTACTTTGCATTTATATAATCCAAATCTTCGAAAAATTGAGAAATCTAAAAAAATAAAAGAATATTTTAACATAAATTGTGATGTTAATATAAGTACTATTTATGACAAATTACATTCATTAAAATTAATCCATATTCAAAATAATAAAATTCATATTTTATGTCCTCAAATAATTGATTATCCAGTACATCCTTTTTTAGGCTTGCAAATATATCAATGCTTACAACATGTTAGTAATAAAGATGCTAAAATAATGTTGATAATTTGCTTTGTCATGTTGCATTTTAATATAGAACATAAATGTAATACAAAATCTATTGACAATGATTTCATTCATTTAATAACATTATATAATCAATTTATCTCTTGTTCAGATAAAGAACAGTGGTGTAATATAATGGGAATCAGTTATAACATTTTGATGAGTATTGATAAAATAATTAAGCGCATCATAGATTATAATTATATTGAATTTAAAATTACTCCAGAAGCATTAGATATAGTTATAGAGTATTTAGTGTATTTATATCAAACCAATATAGCTGTAAAATATGATGAAACATACATTTGTGGAAATAATGAATTAATTTTAGACAAAGATTTTAAACCCAAATATGTCATATTTGGATATATTCAATGCGTAGGAAATAATAAATTCATGCGTAATATATTACCTTTATCTGAAAAATATATAAAAAAATAAATGTTTCAATTTCTTTTTACTAACTCCATATTCAATTTGAAGTCATCAATAATAACTTCTCCTATTGATTTTATGATTTCCTTTCCGGCTTCTAGACTATTGCATGTTATATAAGCCACTCCTTTAGATATATTTTTATTAATTTTATCTGTCACCAAATATACTTGCAGTGGGATGCTGTTGTTTTTACTTCTCAGTATGTCAAAAAGTTGATGTTGTGTTATACGCAACGGAATGTTTGTTATTTTAATACTGTAGTCATTTTGTATCCTTGTGTGAAAGTCAAAACTAAATTCGGGAACAAACTTTTCTGTGGATATTTCACTTTCAATCTTAACCGTGGTATAAATGTTGAATGGAAGAATTTCAAATGAAATAGGTGGCAAATTTTTATTGTATTGTTGAGCTGCGTTTCCAAAAGGTACTATTTTAGATCGTATTGCAATATTTTCTGTATTTGGCATAAACTGAACAGTTCTCATTAAGTTTAACGGTAAATTTTTGGCCTTTAATGTTAGTGTTAGACTATGTAATCACAGATGAAGTCTTTCGATTTGTTCTGAGTCATTTAAATAGAAAGGTTACTTATAATATAGCTGAAAGTACAAGTTTAAAAATAGATGATATGGTGTATTTTGGAGACCAGCTATTATTGAAATTGATAGAATTATTTCAATCTACCGGAAGGCTTAATACTATTAAAACTGCCCTCAAAAATATAGATTCTCAATCTACAGAAGATACACTATTGAATCTTATCAGATATTACAATATGCTAAAAGAGACTATTAGTAAAGGAACATTGAAGTATTATGATGTTCTGGTGCAAAAAAAACAATATGTCAATATTGATTTCGAATTTTATCTGTTAGAGATAATAGCTGGAACAATACAAAGTATAGAACCACTTGAAGGAATGGATAATATTTTTATTGAGAATGTTAATATAGGTACAGAAACACGAATTATATGCAGTGGGCTAAGAAAATATTATCATGAAAATGACTTAAATGCAAAAACTGCACTTTTCATTATTAATTTGAAGCCAGGGAAATTTAAAAATGCCTTGTCCCACGGAATGATTTGCTGTGGATCATTTGAAGAAATAGTCGAGGTTATTTTTGTCGATACTGAGGATAGTGGATCCCGCCTTTCACTAGGTGATACACACATTTACTTCTCTCATATTCAGCGGGGTGAAATTCCTTTGTTAAAAAAAGAAAAATACTTGAATGCATTGCAACAATTTACAATACGAGAAGGATATCTTTATTTTAAAAATACTAAATGTTTTATTAATGGAAAGGAAGTTAAATTAAACAGAATTATAACGGGAACTATGAAATGACTATTATCATAGTTACAAATAAATGGCAAATTATATTTGATTACAAACAATTTTAATCATATTATCAATTCAATAAAATAAGTAGATAATTAATTTATTTTATAACTATATAAATAATTAATTGTTTAGAGAGAATTATCGTGAATATCAATAAATATTCCAGTACTCTTTATGACATCAATAAGTGGAACAAATGATTGATTTGGTACAGACGAATTTAATACATAAGAGATTTCTTTACATAGTTCTTCTGAATCTCTAAGATTGAAAGTTCCACCATAAAGTTCAATGTCTACATTATTAATTTCCTCATCAATCCATTTTCTAGTAGCTGGCCACACCATTTCATCAAATCGTTTAACAATTGCTTTATGAATAAGTTCTGGATCTTCCTTAGAAAAGAATTTTACTGGATCACGTCTTACACGAATATTTTTAGCAACATCCTTATTTCTTACTCTTAATCTAAGTTTTAAAATATTGAATTTCTCTTGGAAAAATGAAATAAGATCGTTTTCAACCCAAACATTATTAGGATGTTCTGTCGGAAGCTTAAATGGTGAAAAAAGTGGCAAATTTAATGCAAATCTATTAAAAATGTTGAAAGCATATATTCCTTCTATTATTATTATGTCTGGATGTGGGTTATCAATAACCTCATCTATTCGGGTTTTAGCTACCAGATCACGTTTGCTAATAGTTATTTTGTCATCGCCATTTGCAAACCCAGTCATTGTTTTAATAAATGTTTCCCAATTAAATGCACTTGGATTATCAAAATCATAAAAATTTAATTTAGTCTCATCGGTCCATTTAATAGTATTGTAATATTGATCAAGAGAAAAGCAATATATGTTATTGTTTCCTTTAAGTATGTTGTAAAGATAATTTCCTATTGTACTTTTTCCACTACAAGTACAACCTTGGATCAAAATCAAATCAATTTTATTATTTCTTGGAAATTTTGCCTTTAAAAACATACGAACCATATCGGTTGGAAATTCTTGTTCCCCACCTAGTATCAAACTCATATTAACGATAGTAAAATTTTATTCTTTAAATAGTTAATTTATAAAAAATTAATGATATACTAATTAAAAATAAAAAGAAAATATATAGTAATTAAGTCTCAAAGAATATGTAATTTATTTTTGCAATATAACTGTTTTAATGATATATTATAAAAATATCTAAATTTAATTATAAAACAATATTAAGACACAATATTCATGTTTAATAGATGCTGTAGTGAGTTTAAAACATATTATTATCAAAAAATACTGCTTTATAATTAATATTAAATAAATTGAAATTTTTCCATAAAAATATTATATGCTGATTAAATTTTTAATTTAAAACAATATTTTACCATATAATTAGTTTTTACATATTATTTTCAATAAAATGCAAAATATATATTGAATCATTATTGCATTTTACACAAATAATCTATTCAATAAAAATATATTTAAAATATTTGAAAATGTTAGATTTAAATATTAATGATCGAATAATAACTTTGACAAAATTAACTCTGTCGTTTTGACTTTTATTTTCCGTTTTTTTAATGATCTTACTAATATCATAATTGAAAAAACATTAATAATGTTAGAATTATTAGGATTTTGTTTAATGAACAATAGTAATTTTTCAATAAAATTTATTTTTTAGGAAAATATGGAGATTATAGAAGAAACACGGTGCAGATTTAAAAGATTGTTATTTAATAGTTTTATATTATCTAGATCAACATTAAGTTTATTTTCATTATACTGTTCATAAATATATAGGAAAAGATATTGTAATTGTTACTAACTAAATTTATTTAAACTTATAGTTTAAAAATACACAAAAGATTTTCAATATGTAGTTTGATTAGTGTAAATAATATTATTTTAATTTCATATATATTTTATTTTTAATAATATATAAATTTTCTAATTTATTTTATTGAATAATTTCATCAATTATTACTTTTTCATTTAATTCATTATTTTGCAGTCCTAATACTTTTTCTGCTATTTTAATTTCAATAGTTGTTGTATGTTGTCCTGCTATGCAAATAAAGTCATTTGCTACCATACCGGATCCCACAATATTTTTTCCTTGATTTACAGTTGACGCAATAGCATTAACTTTTAGTAGTTGTGATATTTCTTTTAGTTCTAATTCATTAATATTCGGATGTGTTATCAATCCTATATTGTTTAATACTCCATATGACCCAACCAAAGATTCATTTCCCAAAGTTAGTTTATAAACCGGAACTTTTAAAACATCTTCAAGTATTGAAATATTTTCTTGTGAAAAATTTGGATTAACAATACATACATGATCATTACATAAAATATTATTTCCTAATGCATTTAATTTATCATAAATTCGTACGACACGAATATCTTGTGGTAATGAATTCCTAATGTGCATTAATTCCTGATCGTTTGTTGAATGAGAACATAGTAAACCATTTTTGTTTCCTACACACAAACTTCCTACCGTTTTAATATTTGCCAGTGTTGTTTCTACAACTGGAAAATCAAATCGTTCACGCAAAAAATCAATAACATTTGAACATTCTGATCTTCCTACAATTGCATACTTGTTAGTTAAGCATATAAAAGCGCCTAATTCGTTATTTCCTTCAAAATCAAGTTTAAAATGCATTAAGTTTAAGGGAAAAATTTTTTATTTTCGACCTTATTTTTATGCAATACATTGAAACTATACTTCAATATATCGATCCTAAATTAGATTATCATGGGCAAGTATTATGTAATAGAATGATGATTGGGATTTTTGCAATCGGATTTGGAATATCATTTCTATTTGGAATTTTTATAAACAATTTAAAATACACACTATATGGAGGTATTGTAACCACAATTATAAATGGAATATTAACAATACCAAGTTGGCCATATTTCCGAAAAAATCCATTAAAATATAAAAAAATAATTAAAATAAAACAAGATTGAGTTTATTAATTAAAATACTTTTTTGAATTTTTTCTTTTGTGGTTTTATCGTTTGTTGTATTATTGTGTGCTTGGCCCAACCAATCACAATTGTTATTATTGGAAATAATATCAATAGATTGACTAATCGTTTAATTGATTTCGCCTCTTTAATATTTTTTTCTTGTTGTGTATTATAATAATAATTTTCTGTTCTCAGAGTATCCATCGCATTAGACAAAGCTTGTAATGTATTTCTAAGAGTTTCGATACCTTTATCTTGTTCTGGAACTTTAGTAGACGTAAAACAGTTAAGATTAAACAATTGGATTTTAGGACTTGTATTAGCTATCATAATAGTATATGTTCCCTTTTTAGTAAATTTTCGTTTAAAAGGTTGATCATCAATTTTATCTCCCAGTGTATCAAATTCAGACTGACTACTTGATAGAGAATTTGTATTCACATCATTTTTTGGTTCTTCGATAGAGATTCTTAAACCTTTTTCTTTTGTTATATTCCGAACTGTCAATGAAAACTGTTCATTATCCTCATTAACTATGTTAATAAACACAAGTGTTTCATTAGGATAAATTTCTTTATCGGCATTATAGAAATTTGCATACAAGCCACATATGTACGTTGAAATAAACAAAAACACAAACATATTTCTAGAAGGGATAAAACCAAATAGTTTTATTACATTAGTATGGTAAATCAAAATTATTGTTAGTATGTGATTAAATATAATAATTCAACCCTGATATTAACTATGACTAGTATCAATCGTTCTAATTTCAATAGATATTACAATAAATTAACGGAATATGATAGAATCGACGCTAATCCATTTAATACAACATATCTTCAAGTTCAAAATGAAAAAAAATCTATCAAAAAAGCAGCTAAATCCATTTTAAAATTAGATATATTGAAAAAAAAAATAATCAAAGCAACTACAAATAATTATACTGAAAAAAAAAGTAACATATTAGAAAATATTCAAATTTTGTCTCCATATGAATTAATTAAAACAACAATTGATTATTTTGTATTTCCCCATACACAGTTTAGTACGTATAATACTGTTTCCTCATTATATAACTATTTTCCAAATGCTCTTAAAGCAGCATTTGAAAATTACATCTCATTTCATAAATTTTTTTATGTAAAACTCAATGATGGCTTGATTAAATTTGAGACAAAAATCTGTATGACAGAAAATTTAGTATCATTATTAAAAGAACATGATATTTTATTTATAAAAGAAAATCAAGATATTATTATTACTGATACTCCTAATATGGTTATGGATATGCTTGGTAACATTAAAATTTCGAACTTAGCAACACTTCCAATCATTGTTAGTAGAGAACAATTTGAGTATTCTATTTATCGAATATTTCGACTTAATCCACCAAAAATTATAAAAATTAATAGCAAACAAAATTATTATTACAAAACTACAGGAATAGTATATCTTGAAGATTTATCATTATCCCCTGATACAATAATAAATTCATAAAAATGACCGATATATGAGCAAGACATTCAAAGAAAAAAAATTGCACCAAAGTCAAATACAAAAATTAAAAAACTTGGAAGAGAAAAAAATCAAACAAGCACATGAAAATGAACTGAAAGAAGCAGCCATGTGGCATGATCCTATGCCTATTACTAAAGCAAATACAAAAAAAGAAAGAGATGTAGAAAAACAGAAAAAAAAAGATGAGTTAAAAAGAAAATATGAAGAAGAATATAATTTGCTGTAATATTATTTATTTTTATTTGTCACTTTTAGTATCGCTTCTTTGTTGACAATTTCTAATGTATCTTTCTCTAGCTTGACATATGAAGTTACAGAGGATTCGATTTGTATTTGGAACGGTTTTCCTTTATATTTTAAGTGTAATACATTATTTTCTACTTGAAGATCTGTCAAGTCAATTTTATTAATATTATTAATGAGCAAGATAAATTTATTAGAGTTTATCATTCACAATATACGAGGGTTAAATATCTACAGTTCATTAAAATAATTACGTATATATCTTCCTATATCTGTTTCTTCTGGTTTATTTTCAGTAGCATCTAGGAGCATATGGGCTTTAGTTAATTTGTCTACTAAACTTGTTAACTCATTCATTTTCTTTTTAAATTCTAAAGATATTGGCGCTGTAAATATTAACTTATGCTCTGCATAGTTAATTTTCGTTATCTGATTAGCTTTGTATATTATGAATTCTTTAATTTTTTTTACTACAAACTCGTTATACCATGATACATACCTTTCAAGAGTATCTTTCCGTGTAAAACATAAATCCAGATACTGTTCATTCATTTTGTCAATGTCATTTAAAATATCATTATTATTATTTAAATTATTGTCAATAATTGTATATTTTTCCAATAGTTTTCTAAATTTAATAAAAAAAACACTATGAGTATCTATGCTTGATATGGCATGGATTTGCTGCAATATTGTTTCTATTGATTCGATATTTATTTTAAAATCTACTACTTCAACTGGACATACTTCTTTTTGGGATTGACTGGTTTTTTTTTTCTTTGGTGCCATTGTTTTATTTACTTTCATACGTGAGAAAAATTTTCGTCCTAAATATGTTAATAAATGAAAAAAAGAAGTATTTATCGACAAAAAAAACAATATTCTTTGATTGTAATGTTAAAAAAACAAAAATGACACAAAGCAATGAAGCTATTCGTTATTTAGTCGATCTGGATATAAATATGATTAATTCTGATTCAGTAGAAAGTATCTTTACAGAAATTGCAGGAAAAGAAACTGAAATTTGCCAAAATGAAGCTATTATTTCAGTTTTACAAAAATGTTTTCAATATGCAACTTCATCTCGTTGTATCCATTTTCTTAATACATTTAATTTAAAAAAAGACATTTATGTTTATTCATGTTCAAAAATAATAGAATCCATTTTATTAAGACTATTTGATTATTTGTATATATCTCTAGACAAAGATAAAAATAATGATATAATATTGACAAAAGTACTTACAATGTTTAATCAAATTAGATTTGACCATATGATAAATAATGAGAACAGTACTCATATTTTTAGAAAGCTACTTTGCTTATTTACTGGGAAGCTTATTATTAAAAATGATATATTAATATATACCCATCCAAATACTAAATATATAAAAGAGATAAAACAAAAATTTAATCGAGAATATAAATTTAGACATGAATCTCGAGTTCATGTATATATAACTCTTGGATGGTATTTACAAATAACACAAAGTCAAACATTTATAACAAAGGCAATAAATTGGTTACGAAACAACAATGGTGATTTACTGAATTTTATTAAAGGAAAAGGTATGTTCTATGAAACAATTCTTGATTTAGCCAATGAAGATAATAATAACAAATTGGTGATGATTTTTAAAGATAATTTGAAATTAATATCTGAACAAGAAGATTATCTGATTCAAAAGATATTAAAAAAAGCCAAAATAAATTTATTTAAAATTTTATTTAATGAAATATATACTAATCCTGAGGATGTTAGTTCTGATATTTTTAATAGCAACTTATTTATAGCAGGATTGATGAATTTACAAAATGTTAATGAGTGGGATATCATTGATCAAATTATTTCTAAATATGTGCAAGGAAAGAGTGTATTTTATGTATTTTTGCTAAATAAATTCAATAATATTGGAGAAATTGATTCTAAATATGTCAGTTTTATATGTAATCAAATGGCATCAACTATATGTAACGACAAAATTAGTAGTGCTCAAACTATAACATTAGATTTTGAAAAATATTTTAATAAGAAATGGATTAATACAAAGTCAGGAAAAAATCTTTTAATGGGATATCTTAATGCAAATATATCAAATAGCCAAAAAGCACTATTTTTTAATACAAATATAAAATATTTCTATAATATTAAACATTGGAAAGATCAACGAAGATTTATGACACAATTATTAAAAATTGCTGATAATAAAACAAAATACAAAATTATACATATTTTGAAAACTATATTATAAATAAATAATAATTATTTTTACCCTAACATGTCTGCAATAACATCTTTCGATTCATTTCTTAGAGCTTTTGCATTAGTCATTACCATTGTCACTATATTAAAAAGAAAGTTCCCAACACTATTTGTTAAAAAAAATGGTGAGTCTGCGTTCTATCACAAAATAGTTATAATAGGAGAAAGATTAAGTTTGTATGTAGCATTTATTTTAATATATAATGGAATAATAAAATTTGTGAATTTTTTTTAATAATTATTAAATTATTGTGCAATTGGTTCTTTTTTCAATAATTCTACATATTTATCCTTTATTTTACGATATTTTTCTATTATCTCATTAAGTTTAGCTATTTTTAATAATAATTCATTATTTTCTTTTTTTTTTGCATGATACAAATGTTCAATATTTTTTACATTATCTAATATTTTACTAACACTAGTTTCAGCATGAGATAGTGTTGGAATATGAATTTGAATTATCTCTAAAACAGTATGCAATTTTGTATTTAAGTGATCATAATTTTTAGTAATTTTATCTAGATTTACTGTTGTTTCTTTAAGTTGAATGTTCAATATTTCAACTTGATGTTGCAAATCTTCAATTATTTTCTGATCTATCTTAATTTTATTATTATTTAAATTATCTAATTCACTTTTTAACTTATCATATTTTTCTTTTAATTGATTATAATGATATTGAATATCATTTAGCATTGCAACATTATGGAATGGAACTGATGATGTCATATTTATTGGTTTATCATTTAGTAAAGACAAATTACATTCATTCTGTACATTTAATTTAATCTTTTTATCAATATCTTTATTAATCTCCATATTATAAGTCATAGCATTGAGTTGATTTTTAAGTGAAAAAATTTCATTTGCCTGATCCATAATTTTGGCCTCAAATAATTCTTTGTTAATATGGTCACATTCAAGCTCATTTATTTTATTCACATATTTCTCTATTACTTTAATTAAATCAGGATATGTATAGTCATTATATTCTAATTCAATAGATTTAGGAACATATAATTTTGATTTAAATTCAATCAATCGATATTCTTCAATTTTTTTTCGGAGTAATTTATTCTCATTTAATAATTTACTAAAATTTTGTTCCATTACACTAACTAAATAGGGTGTAAATTTTAAAAATAATTTATTTATAATTTAAATAAAATGGTTCTTCTTTTATGGTCTCATTTTGAAGTTCATCAATTATTGTCTCTGCATGTTTAATTGTGATAGTATTTAAAAAACATATGCCTTTACATAGTCCAGTCATATCATCAATTAATACATTGATGTTCTTTATCGGTACAGTTATATTTCTAAATGCAAGTAATTCACGAATATCTTCTTCAGTTGTATAACAACATACTCGGGTCATAATCACTGTTGTTGATGGTATGTTTTTAACCGTAATATTTGGCATGTTCATAATTTTTATTTTTAATTGATTAATATATCGTTCTGATTCATTCATTTTTTTATTATTGTCAATATGTTTATTCTTTTGAACAAAATTTTTATTTTTAGAAAAAATATATTCTATTAAATCATTATTTGTATGACTTAAGTGGTGATGTATTTTGGTTTTTCTAATATAGCGAATCTTTCGATAACTAGGTAACATTTCTCGATCTCTACTACCAAAATCAGATTTGAGAGCGTAATAATCGTAACTACTTCGATTACGGTATTTATGATTATTTTTTTTCCGACTGTTATATTTCATTTGGGGCTAGATTATTTTTTTTATAAGTTGCTTTTTGATAAAAACAAATAATCTACTTACTATTCCCATAACTAGCATGTTAAATTGAATAAAAAATCCATTTGCTAAATCCTGACCACATAAAACATAAATATTCTCACACATGTATAACATTTTGGTCCGATTACGGTTGTGAAACTTAAGAAGTAGTCTATATAAATGACGCATGTTTTTCATAACACAATGGTGTTTAAATTGCTTTTTATGCCTTTCAATCACCATATTGAACAATTTGATCTCATTTGTTACAATTTGCTTATTCTCCATATAGATATAGCAAAATAGGGTTAAAACTTGAATGTACATAAAAACAAACGTCCACGCTATTAAAACACTAAAAATTTCATATATATCGGTATTTATGATAATTTATTCTATTAATTTATCTTCTCATATCTCTTCTATCACCTCTGGAAAAATCACAGCGTAATGGTTCATTATTCTGTAAAGATCCCAATTCTTCCAATATTTTTCGTGCTTCTTTTGCTTCATCGACTGTATCGAAGTTGATAAATCCGAATCCTTTACATCGCCTTGAATCTCTATCTTCAATGGCTCGTGCTTCAAACTTAAATTTAATATGATTTGATAAAATATCTTTAAATTGTTCTGACGTCATCTCACTACCCAATCCGAACACAATAAGTCCATTTGATGGATCAGAATTATTTCTTGGAAAATCTTCCCTTCGATCATCCCTTCTCAAATAACCTCGTCGATCATAAAAATCACGACGACGATCATCATATTCTCTTCTCATATCTCGTCTACTATACCGACGGTCATCACGATAGTCATACCCGCGATAATAGTCTCCACGACGGTCATCCCATCGACTATATCGACGGTCATATCCACCTCGTCTGTCATAACTGTTCCAACGATTGTAGCGTCTATCATCTCGCCTTTCATAATCTGATCGTTTGTCTTCTGCTTTAACATCTTCTATCTTACTTTCCATGGATTTCTCATTTTCGGCAGCACTTCCAGGATTAATTGGGGTTGTATTTTTTAAAGCTGATGTATCTTCTATTTCTTTTGGTTCGCTCTTTTGTTCTTTGTCTGTTATTTCGGTAGATTGAATTTTTTTACTAGGCATGTGGGGTAAAATTTTGTGAAAAATAAACTTTAATTATGTAAAATCAAAAAGTAAATATTGATTAGTGTAATTTACTGTTTACATTGTAATATACTATCTTGGACCTTTGTTACTAATAAAATTCGAATAGCATTAACACGTTCCGTTTTATTTGTTGCAACTTCAATAGCCTTTTCATAAAATTTATTCTGCTTAGTAGCATTTCCAAGTAAAATATACGTTTTATAATATAAAGGATCTAATGCGATAGCTTTTTCTAGTAATATTGTATGCACACTGTTAAAATACTCCAAAACTAAAAATGCCGAGACATATAATCTGCAATCCTGAAGTTCAAGATTATGAATATCTCTATTTTTTCCTTTGAATATAAATATATCAAGCATTTGATTAAGTCGTACATCATTTTTGGTTAATATATAGAATTCAAACATAACACATATAAAATTAATATTCGTAACTTTCGTAATCCAATTATCTATATATTTTTCAGCATCACTAAGCTTATTTTGTTTAATCAAATTTATAACAATATCTTGAATTACAACTTCTTCATGATTATTCAATTCTTCCAAATATTTTATCTGTTCATTCAACAGTCCATATTTATCTAAAATTTTACTAATTAATAAAAAATATTTCTTACTAGATATATTTAATATCCGTTTAAAATTCTTTAAATTAAAATTTTTAAACTCATTATTAGTAAAACATGATTTAATTTTGGGGTTATAATCTACATATAAAACTGTATTTTCAAATATTTCATATTGATAATATAAAATGATTAAGTCCATAAGCTCATAGTTATCAATAGACCGACAACTTTTAAAAATATTTTTAGCCCAAGTAAAATTTCCCTTAATGCACTCATCAATTCCTAATTTAAATGCAGAATTGAATTCTGATATATCGACATCCAATAATCTATAAAAGTCAGCAGTTTTTATAGGGCTTACAGTATTTTGATATCTTTCAAAATACTCACTATTGACACAGTCTTCCAATATTTCACCAGCAGTTTTGTTAAATGACTTCATAACTTCTTTATCAATTTCATCGCTATCTAGCATGGTAAGAAATAGAATAGCACGAAACTTATTGATTTTATTAGTTTCATTATTAAAAATATCATATATATTGTTTATTATCGCATTCACAGGATATGTTTTTAATATTATATCATACTGACCACGATGCCATAATTGTTTAATGCTTAATGGTTTATCTTTATTCTTATTTAAGTTACTATCTGAATACACTTGCTTAGATAGTCGAGAATTATTAATATATTTATAGATACCAAATGCTATTGCACATACAGTAATACAAGTAAATGTCACAAGCCTCCCTTCATTACTATTTCTCTTAACCATAAAAATTTTGCGGCTAAATTTTCGTTTTTTAAACGGTCATAAAATATAATTCAAAAAAAATAATTAATATATTTTATTTAATTAATTAATGAAATATAATGTTGAAATCTTTCACTAAAATATGAATCTTATTCCTCTTTGTTTTCTTTCTCTACATTCTTTGTCAATTTCTTGTCAATTACTTTACTATTTTCCTCTGTGATAGTATCCTTAACACTTGTCAAAATATTAATTGGACTGCATGAATTGTCACAGTTTGCAAATTTACCCTCTGTTGTGTTCAAGAAATCAGAGCATGGCTTGCAATTCAATTCTTGCGGTTTCTTTTGGTTTGGCTTTCCTTCAGAAGTAATCATATCACCAGCTCTGTTAATATCCATCTTGTAATTTGGTTTTTTAAAGAATGTTCGGCATATCTTTGGTGGTTTCATTATAACATATTTAGGGGATTCATGAACAATCATCATTAATAAAGTGTCTCCCTCCCAGATTTCAGCTTCATTATTGTGAACATCTACTGAAAATCTAGGATCTTTAATCCATTGTTGTAATGCAGTCTTAGTATAACATGCTTTGCTTTTTTGATCTAATTTGGTTACGCATGGCTTGGATGAACTTGAACTGCTGCTAGGTTTAGACATCTCCTTACTAATCAATTGCTTAATAGAAGTTGCTATCTTACTAAGTTTTTCTTTTTTCTCAGCTATACACTTTTGAATTTCAACAGGATCAAAGGTTACTAATGGTCGTCCTTCTATAGTTTGATTATTTTGTTCTGTCTTGGCTCTGCATTCTGCAGCTGCTTTTTCTCGAGCATCTTGCGCTTCTCGTTGTAATTCTTTAAGATGTTCAACTAATCCAGTAGGATCAACAGCTGCTGTGTTTAATAACTGTTGCTGTGGTGCAGTACCAACTTGTGCATTCACAACATTAGCTGCTAAAACTATGGACAATTTGCCAAAAATATTTAATGAATTCATGTTTTATAAACACAATGTGTTTTTAAAGTTTGATTTAAAACATTGTTTCATCATGGTCCAATAAGTTTCAAAATGATTATAGCTTTAATAATTATATTTATCATTGATATAGAAATAAAAAATATATAATTAGATATTCACTAAATACCTTAGTACCAATCTATTCAGAGTCCAAAACAATTTGATACTCAAATCTAATTTAAAAATCAAATATAATAAATAAATTATTTATTAATTGCAACCTGTTGGTTTCACGCATTTTTTGATTGTTGGTTCAACGTGTGGGCTTGTTGGGACTTCAATACCACCGATATTCATTGTAGGTGTTTTAACTTCAGGTTTAAAGCAATCACCATATCCACAAAATGAACCGCTTGTCAATGGTTTAGATGTAAGGGGAGTTTGTAAAGAAGATGGTGTTGATTTTCCAGACAAATCAAATGTTGGAATATTATTATTGACTGTATTATTTCCACCTAATAAGTTTCCTAACATATTGTTATTATCACTAGGTAAATTCATAGTGACTCCATTATTATTTAATGGAGTATTGTTAGTAGTTCCAAAAGCTTGGTTATTGTTTGTAGTATTATCTAATCCTTTTCCATCGCACTCTAAAGATTCTACAATTTTACAATTTTTATTTCCACCTATTCCTGGAATTATTATATTTGATCCTGTATTACTATTATTATTATTTCCAATAATTGATGAGATCTCTGGGCAATTTTGAGGTCCATTAAGTCCCTGCAAAAGTTTAATTATTTCATCTTTAATTTTATTAATATTAGGCTGTGTATTATTTATTCCATTCATTACTCCAGTTCCATCCATTTGGTTTTGGTTTAAATCTGCAGATCTGATTAATGAAAAGAACAGTATACTTTTATTGATAATAAACATATTGTAATAAATAATGCTTATTTTATAAAACTTATGAAACAGATGTTTCACTTTAAGTGTAGATTCTGTTGTTTAGTCATAATTACATGTTTCACATTGAAGATAATGATGTTTATTCAAAACATCAGTTCATCTAATAGACCATTCATAATATTTGCATTTGTCTTAGGTCTTTTGTTGGGTTTGTTTTCTTCATAAACACAGAGATCATTTTCTTGGAGGATTTTCAAACAATGCAAATCCTCCATAATAACAAGTGGTTTATTAGACTTGAAACTAAAGTAATCTCGTTCTTCGTTAGTATCACAATGGATTTCAGAATTTTTATCTGTAAAAACAAGCATAAATGGCAGGTATAATTTTTCTTCGGAGCCAATATGAGATGATTGCTTATTTTTAGTTGTAATATATTTGAATGTATTTTTCATTAGAGTTAACTTTTCTCGTTCTTCAATTTTCTTTGTAATATCATTTTTTTCCTCTATAAATATATATGTTTTTCCGCGCTTATCAATTAAATTAACAGCCCTAAGGACATTAAGCACGTCATATATTCGTCGAATTTTGGTTTCACTATTTTTTGTATCAATTTCTTTTACAATTTGGTTATAAGTGCATTCTTTTTTTTCTTTTATTATATCGTATACTGCTCGAGTTATATATTTCATGCCTTCCCGTTTATTTTCAGAGTTCAAGATGTCAGATTCAAGCATATTTAGAATGGGGTTATTATAGATTGAATTTTTATTAAAAATATTAACATCTCAAATTTTGGTAAATTTTAGATGTTTATAAATAAAAGTAATAGTTTGTAGACAGATAATATAATTAAAATTTTATGAACGCGTTTGATTTTTATTTAATATAAATGATGAAGTATGCTCAAATGATATCAAAAATTCTATTGATTATTTATAAACCAAAATATTATGATTTTTATGTTATATAGTTTATTTTTAAATTTTATATTTTCAACAAATACATTAGACTTAGAATATAGACATGTTACAGTAGATGATGTGAAAAATAATTCTTTTATAAAACTTTTAGGACAATTAACTACTACCAATCCAATTACTCAATCCAAATTAGTAGATATATTTAATGAAAAATTTAATTCTGGGTTATATACTCAGTTTGGCTGTTTTTGTAATGGTAAATCAGTGGGATTATTAACTGTATTATATGATACCAAATATGCACGAGGAGTTCCGGCAGCTTTCATTGAAGATGTTGTGGTTGATTCACAATATAGAGGAAAAGGAATATGCAAACGATTATTAGAATTAGCCGAAGAAGATGCTAGATCAAAACAAGCTTATAAAATGATATTATCTTGTACTGAAGATTTAGGGAAATCAACATCTCCATATATTAAAGCTGGATATAGTAAAGATGGATTATGTCTTAGAAAAAATTTAATATATGATAATTCAAAATATATTTCCAATCCAATAATATCAAATCCACCTATCACAATAGAATTAATGACGTCCTAATATACTAATAAATATTTGTATCAATATTAAATTAAAAATTTTTAATTGTGTAACTTTGTACGCCCAAAGTACCCATGAGTTACGTAATTTTTATTTTTGGGGCGGCTGGTGTCGGAAAAACAACATTTTGCAAAAATTTTAAAGAACTATATTCAAGAAAACGAGTAAATTTAATTAACTTAGATCCAGGATCACTGTCTGAGGATATTCAATATGACATAAGTATTACTGATTATATTTCAATTGATGACATAATGATGGAACTAGATTTGGGCCCAAATGGTGGGATGTTTGAATGTTTAAGTTACTTAAATGAAATTTTCTTTCCAAGCGAAAAAGACACAGAAAATAATGCATCAATTTTTAAAGAAGATTCAATAATATTATTTGATTGTCCTGGTCAAATAGAACTGTTTTTGCATAGCGATATTCTTCCACAATTTATTAATAAATTTAAAAATGCAGATGAGTGTTCAGTAGCTATTGCATTTATAACAGATATTTCTTCATTATATAACTATAACAAAATGTTTTTCAATATGTTGATTATAAGTCTAGCAGTTAATAGATTTTCTTTACCAGTTATTAATTTAATAAATAAGCTTGATCTAATGGAGAAATTTGATGAATCACCAGTAAAGTACGATATAGATTCTGGAATGATTACCATTGAAGATGTATTTACTACAAAACCAAGTATGTTCGATACAACTCTTAAAGAGTTTATAGAAATGTATGGTCTTTCACAGTTTATACCTATTAATTGGGAGGACGATGAGCATACAGAGTTCTTATTTTTAAATATTGAACGCGTTTTAAATATGTACGATTAATAAAAATCAAATGTAATTTAAATCTTACTAAACTTCAAAATCGAAAACGTAGTTCCGAATATAAAAAAAATCAAAAATACAAAAAAATATGTTTTTCGTATACTAGTAGTTTTTTTAAGTTTAATGAGGTATTTTTCATTAAATTGTAATGCCTCTGAATATTGCAATATTTTATTTTTAACGCCTTTAAGTTTTTCATTTTGAGCATTCAAATCTGTTTTAATAATATCACAATGTTGTAATGCAATATTAAATAGTTCGTTTTCCATATGGTTTGTTTATGTCTGTTTCAAATCGAATTTTTATTTACAATAGATTGTTTAAGCATGAAAGACATTGGAATTTCTAACTTACCAAATCTCCATCATAAATTCCATCTTGAAAAAGGAATTCAGTTTAATATAGTAGTTATTGGATCTCATGGATTAGGAAAAACCACTTTTGTTAACAATTTTTTTGGCCAAGAAGTTCTTTCGTCTGAATTAAAAACAGAAGTAATACAAGATGCCTGCTCAATTGAAGTTAACGAATTTGAAGTTAATGAAGAAAATTATAAAATCAAAATGGATATTATTGAGATTGATGGAATTGGAGATAATATAGATAATTCTAAATGTTATAAACCTGTTATTGAATTATTTATTGAACGATTTAATGAATATCAAGAACAATCAGAAAAATATATCAAACAATCTATCAATGATAACAGAATTCATTTATGTTTATATTTTTTAGAACCAATTTGTTATGTAAAAGATAGTGATCTAATAGCATTTCAAACCATTTCTGATTATTGTAACGTTATTCCAATTGTATCTAAGGCTGATTTGCTTACTGCAGATGAGATAGAAGTTCTAAGAGATCTAATTCAAACAAAATATAAGGAAGATCCCTTTTTTATTAGTTGTACGACAGATTGTAATCTTAAAAAAGAATACAACTGGGGAATCTTAGATATCAGTAATTTTGATATGTTCAAATTAAGAGAGTACATACTAGAAAAACAAACTATATATTTAATTGATGAAACAGAATACCTATACGATCAATACCGTATTTCACAACTAGCAAATGAAACTCCTGAAGGCCAAGAATTCTTGAAAAAATTGGCACATAAACAACAAACTCTTGATCAAATTAAAGAAAGAATTAAAAAAAAACAAGAAATGTTATAAATAAATTCATAAAAATATAATTTTATTTTATAAGTAAACTGATATCTTCGGATGAATATTTTTTAATTCCATTCTTGGTTAAGATAGAAATTTCTACATTAGTTTCTGTCATTTTGTCTGCCATACTGTGTTTTAAAATATCGACTAATTTTTTTAATGTAAGTTGTTCTAAATCCATGACTTCTAATTGTTGTTCTATTATTTCGCTAGCGGCACCTATTGCTTTTGCACGGTATTCTGTATATGATCCTGATGGGTCCATGCAAAACAATCGTGGCGTATTTTTTTCATAAACGCCTAAAACAAGCGAAATACCAAATGGTCGGCTATATATTTTATTAAAATCATCTTTTTCTCCAAATTTAAGTGCTAAATTGCAAATCCTGTCCAATAATAGTTTAGATGGAATTAATGTACCATAAAATGCTTCATGTTCTAAACAAAATGTTCTACATTTTTTGATTATATTTAATGCATCGGCTGTTATTCCTGCAAATGCAGTAATAACATTATCATAAATTTTGAAATGCTTTTTAACACTGCTGGGTTTTTGCAATATATTGGCTAATTTTTTTTCTGATACCATCACAATAGAGTTATTTACAATTGCAGCTATGGTTGTAGTTCCAAGTGTCATTGCTTTCATGGCATATTCTATTTGCTTTATCCGACCATCTTCCGTGTAAATATCAGTCTGTGTATCGTAGCTCATATATAAGGGTCAAAACAAACAATGTTTTACAACAAATATTCAATACGTTGTAAAATAGTAGGATGAAAGCTTTTTAACGAATTATACAGTATTGTATACTGTATTGGTTCATTGCTTTGAATTGTAATTTTGAATAATACTTTTGCTAAATTGACTCCAAATCCATGACGTTTTGCTATTAAGTCAGCATTGATTTCAGCTTTTTGGCTAGTAAGCTTATGAATTATCATTAAATATCTATTTAAATACATCTCATATACAATTAATAGAAACAGAAAAGTAGGAATTTTAGATATATTGGAATCAACATATTTATCAGTTACTGAATTCCACAGCCACATAATCAGGCAAAATTCAATACCTTTAATAATATACAATGCTATTATTTTTTTATGTAAAGAATAGTCCTGTGAATGACCTAATTCGTGCATTAATATAGCTTCAAATTCATCATCTGTAAAATGATTATGCTTTCCATAAATTTCAATGTGTTCATACTTTCCCCATCCGACCAATGCTGCATTTATAGAGTCAGATTTATGTTTTTCTTGGAATATTTTGTTTTCTAGAGAACGCTCCTTTAGATAATCTCGGATTGTGCGAGAATAGTTTTTAATATTCACTCTTTCAAATTCATCTAAGTTAATATCGTCTTGAGTGAAAAATTCTGCAGTTGCTTTTATATAAAATGTGATATAATACGCTATAATAAGCTTTGAACCGAATATTTTAAATAAATTACATATTAAAATTAAAAAAAAAGGAAGTATAGCTCCAAATAAACATAAATAAAATAATATTTCGAAAACAATATTGTTGAATTGTGATCCAAATAACTTAACGATAGCATTTCCATATGCTGTACAAACACATAAGATTACAAATATAACTAAAAAATTAATTTCTACACGTTTACAATTTAAACTTTTTGTTTTCAGAATATTCATTTTCGCTTTGCAAATATTAATAATATATGATCTTGATTTTTTATTTGAAAATAAATAAAAGTACATTGTAAGACCAATCCATATAACTGCTTCATATCCAAGACGATTAAACTTTGTATTGAGTGCTTTTATCTGACTATTAATAAATTCAGACTTATTATCAATTAATTTTTCTATTTTATCTCCGAAATAGTGAATATCATCTTCTATCTTTAATTTTATATATAAAAATGCACTTTGAATCATTTCGTTTAGAACAACGAATAAGCTTATGAAACAAAACCCAAAAACGATGGTAATAAAAGACAATTTCATGAATTTTACCAAATTAGATTATTTTATTGATAACTATGAAACATAATTAAGGTGAAACTGATTTTATATCTTATCACACCTGATTATATCGGTAAATGCATCCAGTCCCCCTATAAATTGTCCATCTACAAATATCATTGGATATGTAGGCCAATTGTTGATTTTTTTAAGATATTCTCTCATTATATTATCTTGGAAAATATCAAAAGCTATTATATCTATTATATTCTTTTTATTGCATAATTCGACTAATTGCTTAGAATATTTGCAATATGGTTTTTCTATTGTTCCTTTCATGAAAAATACATACTTTGATTGACAAACAAACTCTTTGCAAAATTTAAAATAGTAATTATATAACACATTTTCAACTGTATCTTTAGGTGTAATTTTTGTATTTTTAAATATAAGTAATGGAAATGAATTTACATTAAATGTTTTACTAACAAGTTCTTCAAGTTTTTTAGAAATACTTAAATCGACATATGCTACGTCATCAAATTCCTTTATTTGCGTATTTAAATTTTCAAATGCAGTATTGTTTTCTTTGTAAAAAAGGACAAAATCATTTGTATCACATATATTTTTCAAATCCATTTTTGGTACTGGGAGTAAAATATTTTATTAAAATTATTTAGTACTGGATACGATTTTATTGAGAGAATCATCATTAATGTAGAGTACATTTATTGCCGATCCATTATTTAGCTGAATACATTCTTGACGACTCATTAGTGTTATTCCAATTCCAAATAATACACCAACAGTGTTATCATATAGTGAAATATAAACTATTTCATCTTTGTCAAAATTTGGTTGCTTATCAATATATTTAGTTATACCAGGAGCCATAACGTACGCACCTCTTTCTAAAGCTTTCTGTGCGCCTTTATCAACAAAAATAGTGTTATGCTTGTTTGGGTGGAACATATGACATATGGTATTATAATATGTAAAAAATTGGATTTGCAAATCATCAGTAGTCACAACTTTTGCTTTGTTGGTTAACAAAAAACATTTGTATACAGTTTTTAGATTCATATAATCACATCCATCTTTGATATATGTTTTAATGTCTTTTTTACTAAGTATGTGAAACTGTTTAATTTCAGCTTTAGTAAAATATGTCATATGGTTAAAATATTTTGCCCATATATGGAAGATATTGAAGGTCCAACATCTAATTTTTTCAATGATGAGGGAAGATTACTTCAGGCAGAATATGCTGTTAAAAATGTATCACATGGAGGAACGATAATTGGAATTGTTTGTGATGGTGGTGTGGTGTTATTTGGGTTAAACCAAACAAAAAGCTTAACGAAAGAAAAGATTTATAAATTAAATAACAAAACATTTTGTGCAGTTTCTGGATTATTTGGGGATGCAAATATGCTTATTCAATATGGTCGTAGGATTAGTATTAAAATAGAAAATGAAATTCTTGAATCTCCTAAACCATATGTGCTATGTAAGTACATTGGACAAAAAAAACAATTATATACAAATATAACAGATACACGACCATTTGGAGTTAGCATACTATATTGTGGATATGATGATAAGTATGTTTTGTATTCAACAGATCCATCCGGAACAGTTAATCAATGGAAAGCCTTTGCCATAGGGTTGAACGAAAATTTAATCAATCAGAATTTGAAGAATGAATATGATGAAACACTGCCATTTGATAAGGGTGTAGAATTTCTTTTAAAAATATTAATCAAGTCAAGAGAATGTTCTTTAGATATGATTGAAAAAATGGAGATATTATGCTATAATAAGGATCAACAAAGATTTTTAACCCTTGATGAAATTAAACAAATATTTGAAAGTATACAGTCAAACATCTCATAATTATTAAATGATTTTATTATATGTTTTTTTTTGATATTATCTTTTTTAAATTTCTGTATGTTAATAAAATAACTATTTCCCTCCAGCAAAATAAAACAAAAAATTGAATCCTTTATGAGAAATTTTAAAACATTTAAAATGCCATATATTTTTATAGATGATTCTAAAGGTAAATATCAGCCTATATATAAAGAGTATGTTAAATCGATCCCAAGATTAAATTTGGAAAGCGGAATTCTTAATTGTCCATTTTTGCCGGAAAAAAATGAAAAAAAAGTAAAACCTAAAATATCAAAAAAAAATAGAAATAAAATAAAGTATTGTGAAATCTGTTGTGTTAAATATGATAATTATTTTAAACACATAACTACCCCTCTCCATCTTTCTTTTAGTACTGATGAAACCAATTACAAAGATATCGATAAATTTATAGCTAGCATGGAGAATATCGTTTTTTCTCATGAAATAATTTATCAATCACCATGCAAAGGAATAGATGATGTATATAAAATTAACTATCAGTTTACATCTCTGAATAGTATTATTCAGCTAAGCAACCCCAGCACTAATTGTGAAATTATAAGTAACAACGATCCAAATATACTAATTGCAGAATTACTTAAAAGAAATTATGATTTAAATAAATATTAAATTTTAATAGTTTAACTACTTATTACTTATTTAAAATTTGAAATTGAATATACCCCTAGACATATGCGACGTCTTAAGTACCATGAACAAAAGTTACTTAAGAAAGTTAATCTCGAAGAATGGAAAAAAACTAATACAACTAAAGAGCAGTTAGTAACAACAAAATATGTACTAACAGACAGAAATACATATCATATCTATAATAAAATTGTTGGAAAAATAAGAAAGTTGACGGAAAGTTTAGCTGCTCTTGATAACACTAATATTGCTAAAAAGTACTTATCTAATAAATTACTTAATAAACTATTTGATGCAGGGATAATTTCTGAAAAAAAATTATCTGAATGCATTAAAATCACTGTATCAGATTTTTGCAAACGGAGACTACCTATTGTTATAGCTCACAGAAAATTTGTGGAAAATTATCAAGATGCAGATAAGTTTGTTCAGCATGGACATTTTAAAATTGGAACAAATATATGCCGTGATTCTAATGTTTTAATTCCAAGGGCTATGGAAGAATTTATAAAATGGAGTGATAATAGTAAAATTAAAAAAAAGTTAGATGAAGTTTATGGCGATTATGATGATTTTAAATACAATTAGTCATTTCATTATGAAATAAATTTTAATACCCATAATGATAAAAAATGAAGAAAATACAAATAATAAACTACTAATTAATAACGATAAACATACAGCAATACAAACAATAGAGAATAATACATCTAATTTGGCAAGCATTGATCTAATGGAATATTCAAGCTATGTTGATAAAAATGTAGTTGTAATATTAAAAGATAATAGTTTATTGTACGGAACGTTAAAATCTTATGATCAATATAATAATATTTCATTAAATTATGCTGTTCAACGAATATTTCACAATAACCAATATGCTGAAAAAATGATTGGGTTTATCGTTATTAGAGGCGATACCATTGTTATGATTTCCTTAGCACGCTATGATTTATCAGGTTTAGAAAAAACGGAATTTTTTAAATTAAAAGAAGAGTTAGAAATATATTTATTGAGTAAATCTAATAATTGAAATATTTATTTTAATTTTTTTATAGTTAATTCTTGAAGTTCAGCAGAATCTTTCTCAGCTTTGGCTTTATAAATGATATCATCAATATTTTCTAATCCTATATTTTTAATATCAATATACTTTAGGATAGCTTGTTTATTTTTTTCAATTACTTCACTAAATATATTTTTATCTTCCTTTTGTAAAAACTGAGTTATACCATCAGCTAATGCATAATTATCAATAATTTTCATGGGAAAAAATGTTAACATTTGATGGAAAATATCTATAAAAGTGGTTTGATTGACTTGAGGTACTGTTACATTATTTTCTTTTATCTTATATTTCTTGATTATTTTCAAATAATCCTTTATATTAGCAATTTTACCATCTACATAAATATCTAGATCATATTTATTAATTAGATATTTTTTGGAACATTCAATACGAATACGTAGTAAAGGTAATAGATCTGTATTTTCCTGTTTTATTGTAACTAACATTTTTTGGATAATCTCATAAATTTTTTTATTATAATCTTCGTCATCTACTTTTAATGTATCAATATATAATGGTCGAACAGTTTCTAGTTTTTTTCTAGTTATATGCGCTTGATTATTAGCAATCTCTAGGAAATATACAAATTTATCACCTTTTTCATACTCGCACAAACTTGTTCGAACAGATGATCCTACTTGGATAATGTTAAAATGATTTGTTGTAAATTTTTCGCTGGAATGTTCATGGCCAAAAATAACGAGATCAAACCATGAAGGAATACATTCTTCAGGAAAATATTCATTTTCATGAAATGTTCTGTTTTGATGTACCATTAAAATGTTATAATATTCTCCTTCTAATTTTTTGTATATAATCTTATTCGAACTTACTAATTTAAATAACTTTCTATCTTTGATGTATCCAAATCCATAAATTGCAATATTAATACCATTAGATTGAAGAATAATAGGTTCAATTTCAATATGGTCAAGTGTTTTAAATTTTCCAAAATAATGGATAAATCCCGATGCATGCAGTATATCCATTGGAGAAATAGCATTAAAACCACTTGGATCATCATGATTACCATGAATCGCAAGAATTGGAATGATTGAAGATGTTGAAATATTTTGAAAACGAATATTTTTATTATTACTGGTTATGTATTTTTTAAGTAACTGGAAGGTTTTATAATATGTATTTTGACTGGGTTTATTATAATGGAATAAATCACCTGCTTGTAATACCAAATCGACATTTTCTGATTTTATATAGTGTAATATCTCTTCAAACGTATTAAAACTATCATTACCTCTGATTCTATCTGTTTCATTAAATCCCAAATGATTATCAGACGTTATTAATATTTTCATACTGTACCCTTTATGGTAAAAAAAATTGATTTTCACAAAACACTTATTGCATGGATATTAGGATTATTTATATCTCCTATCATTATATTTTTTTTATGTATTAAAATCAAAGTTGAAGCATTTAATGCATGTTTTATTGCATTGATTATATTTGTTTCTATTTTGTTAAAAATTGCATTGATGACAACGAAACGATATACAAAGAAAAAAAAACTGCTGAAAGAGTTTAATAAAAAAAAATAGCATTATTTATTTGTGAAATAAAAGCCAGGAAGAACCTTAATTGGCTTAGATTTCCTTTTTCCAAGCAAAGATTGAATTGTACTCCAGCTATGTCTTACAATAGAATTGAATCCTGAAATTGGATGAAAGTTATCTTTAAGCCACTGTTGTGTGTGGGGATCTGATGGATATCCACTTCCACAATGATTAAAATCCTTTATAGAAGTGAGTTCTTCGTATCCGAGTTTCGTATAATAATTTTCATGTATATTATTTGTTTTACTGCTAAAAAATGCATCTCTATTAATTTTGGCTACTATACTTGCAGCAGAAACAACTTCATATGTTGCATCAGCCTTAGCTTCGATTGTAAAATTAAACTTAAAGTGTGCTTGTAAGTTTTTTTTGTAATTGCTAACATTACCTAATGCGTCTATATATACCATTTTAACGTTTTTACATTTGTTATTAATTTCATTTAGAAGCTGTACTACTGCCTCTTCTGCAATTTGATTTAAATTTTTTATATGATTATTCATATATGTAGTGATATATATTGGCTCAATAACAATATATGCAAAATTCATCATTGTAGCATAGTAATTTTTTCTTGTATTTGGGGTTAATTGTTTAGAATCTATAAATTGTCTTATACGGCGTTCATTAGTATGCATCACATATGCTGCATACACCATTGGACCAATAACTGGCCCTCGGCCTGCTTCATCGATTCCTACTATAACTTCTTCAGTTGTTTCCTTAATATTGGAAAATAATTTCATAATATATTGGGATGAATTTGCAAAAAATCGAAAGTAGATTAATTATTAGTTTATTCACCACATGATCCCCCTAATTTTCGTGGATCAGCTGCTGTTGTAATTTCAGTATGGTTTTTGGTCCTTTTTACATTAATTCCATTAACAGATGTGAATGTATGCATTTCATCGCTTTTTACAATTTGATGACCTAATAATTTAAGTTTTTCTAATAATAAATAAGGAAAAGCATCTTCAACAAATGTTGTTTTAGGTAGCAATTGATCGTGAATTCGTGGCATAGTGATTGCTTTATATAAATCACCCGTTAAATAATAAAATAATATTGTTGAAACAATTGATGTTGGTATTTTTGTTCCACCAGCTGCACCTAAAAGAAGTAACTCTTCAGATTCATTATCCTTCTTAATTAGAATTGTCGGACATGCAGATGAAAATGGTCTTTTACCAGGTTCTATTCGATTCGATTTAGATAATGATAGATTATATGCATTTAATATTCCAGGAACATAAAAATCATCCATATGATTATTGAAAATAATTCCTGTTTCTGGATCCATTACTTTGGAGCCAAACTCAAGATTAATTGTTGTTGTGATTAATACTGCCATACCGTCTGAGTCAATTACATTTAAATGTGTTGTTCCATGATCTTCTGCAAAATCAACAATATTTCCAAATAGTTTACGATCTAATGGTTTTTCAAAATCAATTTCATTAAATAGTAAGTGTGCTAATGCCTTAGAAGTTAATTCTATAATCTTCTCATCAGACTTATCAACAAATGTAGGATCTCCAGCTTCCCCTCTTTTAAGAAACATAAATTTAAATATTTCTATAAGTATATGAAAGATATAAAATTCATTCTGATGACTTAATGTTTGGTATATGTTTTTAAAATCAAATTGGTCGAGAATATTTAGTGCCATTAAAACGAATGCTCCAGATGTTGGAAGATTAGTTGTAATAACATCATATTCTTTAAAATTTCCTCTTAGAACATCTCGTTCCTTAACATTATAATCTTCTAAATCTGATTTTGTTAAAAAACCATTATTATTCTTGCTAAATTCTACAATTTTCTCTGCAATTTGTCCCGTATATAATGAATCTGGATTTTGTGCAATTTCTTGCAATGTTCGACCTAAATTCTTTCGAATAATAATGTCTCCTTCTTGTATAATATTACCATGTTTCATAAAAATTTCTCTAAACCCGGGATCATTTTTAATAAAATTTTCGTTACTTTTTAATTTGTTAATAAGCAAAATATTAGCTTTAAATCCAGTGTTACATAGTTCAATACATTCATCAAACAGTTGATTCCATGGAAGCTTTCCGAACTCTTTATGAACTGTATACAATCCTTTTAATTCACCAGGAGTACATGTTGATCGTATTCCAAGTTTTAAATCTTCTGGATGATTTATAAAATATTCTTCTTTAGCTAATTTAGGAGCTGTTTCTCTAAAATCATAAAATTTATATGTATCATTTTTTTTTAACATTAGAAAGCCACCTCCACCAATTCCAGCAGAAAATGAATTGACAATTCCTATGGCAATTGTTGCAGATATTGCAGCATCAACAGCATTTCCACCTTTCTCTAGTATTTTTTGCCCAATCTGACTTGCAATTACACATTCTGTATTTACAGCGTGTTTTTCAAATTTATTTACAGTCTCTTTAAGCAACATAGGGGTGTGATATTTAATAATACTATTAAATATACTATTTTCAAAAGGGGTAACTAATAAAATAAAGCTAAATACAAACACACACATTACATACATTGCAATAAAAACAATTTTAATTTACTCTAAACATTAAAAAACAAATAATAAACAAAAAGTCTAGGTATAAAAATGTAATATTAAAACCTACTTATTAATGCTTGTAGATGCTAATTTATTGGATAGTTCAATTTCAATATCAGGATATTGGATATAATCAAATGCTAAATCGTAAAACACAGGCATTAACGGCATGTTTCGTTTTTCTTGATTTACTGTAGTAAATTTTTTAAAATACATCATAATAATATCTTGTATGTTGTTTTTTGTTATTTTAATATTTTTAATACTAGTAGTAATTTGTTCTTTGTATATTGCAAAGTTAGATGGTATAAATAACTCTTTAATTTTGCCATTAGTTTCTATATACCCACTTAAATTCTTATAAAATTTATTCAGTTCACTATTTGTATTTGTGAGATATGCAGAAGTGATAAAATTATCGTCCAAGAATGTAGTAAATTTTTGTGTTTCAATAGAAAATATTTTTATTTTATTAAAAATATTGACAAAATCACCTTGATTTATAGCAGTACATGCATTGTTGTAATTTTTATCAAAATTGTGACAATACCTAGCCATTTGTGTATTAAATCTAAAATCATTATTATTGAATACGCCTTGTAAAAAGTTATCTATTTGTGCTTGATTGTCGAACATCAATTGGATATCATTCCACATATATTTGATTCGATATTTATTAATATCAAATATATCATCATTTAATGTTATTAAATCCATATTAAAGGAATAGTAAAAGTTTTGATTAAGTACATTCCAAGTATGCGCAAAATTTTCTTCTATAAATTCATTTTTAAATTTAGAAATAAACAATAAAATGTATTTTTTATAAGCTTTAACAAATTCATTATCATCTTGTTTATTAAGAATACGTATATTTTTTCTTAAGTATCTTATTGAATTTGTTTTGAACCATTTAGCCAAATTTACCTCAAGTTTGTAAATCAATGTGGTTGTTTTGTTTTGATTGCATTTTTTGATCATTTTGTTATTAAATTGTTTGTATTCATTATAATTTTGCTTTAAATACCCATGATTATTTCTATTTATTTCAATTATTTTTAAATAATCCATTAAGGATAAAAAACAAAAACTTTATTATTTTTTTTCTATGAGTAAAAATCCATTCAAAAAATAATAAACAACCCATACCATCAAAAATTGACAATCCATTCTGTCTATTATACCTCCATGTCCTGGAATGCAATTTCCAAAATTTTTTTTATTAAAATATCGTTTTGTAACAGAAGCTATAAATCCTCCTAATGGTGTTATAAGTGATGCAAAAACAATAAATACTAAATTGTGGTAAAACTGGACAGTATCTATTGTTGAGCAGATACCCCAATTATTTATTAGTATCTTATAACAGTTAATAGGCATTATTTCATGGTATAAGTGTGTATGTAAAAAGGCCCATCCAATAGCATATGTAAAAATAGCACCACCTATAAATCCTTCCCATGTTTTATTAGGACTAAAGGTAAATAATGGTGTTCTTCCAAATGTTTTTCCACTAAAATAAGCGCCTGAATCGTTAGCAGCAACCAATAATGCTGGATAAATACAATAGAATTTTCCTTGTTGAAGATTAATTATTGATATTCTGAATGTAGAACTTAGAAATATTGCACCTAAGTGACTAAACATTAGGATAAGTAATTGATTTTTGAATGTTGATTTCTTAAGTAATATCACATATACTAATATACCTAAGATATATAAATAAAATCCAATACTATTGTAGTGAATTAATTGACAATAATTGGTAAAATTAAATACACCACTTATGAATGCTGGAAATATCATTATAATATATGCCATGATTGTTGTGTAGTAAATTAATATTTTAGTACCAAAACTATTTATTATATCATCCATTATATTTAATAGTTCTAACAGAGATTTTGTACATATCAACAAAAAAAATACAATCAAATACCTATTTGGTAGTTTACATATGAGATATAATAATCCCAACATAATACTTCCAAAAATTACTCTGAAAACATATTTTGGAAATTGTAAGCTTATCCTGATAATCATTATAAAAGGGACATATACTATTGATTATTAAAGGAGAATGATATCTATTGTTTAAAATCTTAGTATTAAAAATAAAAATTCTCAAACTGGGGGTCGAACCCAGGGCCTCCGCGTTATTAGCACGGCGCTCTAACCAACTGAGCTATTCGAGATATTAAATGTAAAAAATATATTCATAATTTAAACACCCATTTATTTATAAATGGAATTAAGCGGTAAAGTAATCTTGATAACTGGTGGATCGAAAGGCTTAGGATATGAGCTGACTAAACTATTATTACACAAAAATGCAATTGTATGGAGCATAAGTCGAACACCTGCAAATATATATCACGATAATTTTAATGAAATTCTTTATGACCTAGAGTTATTAACCAAAAATCAGGTTCCAAATACTATAAAGTGTCTTTTACATATGCAATTTGATATTCTTATAAACAATGTTGGATATAATCCTGGACATACTCTTTTTAAAAACACATCAATCTTAAATATTAATACATGCTTAGCTATAAATATTTACACACATATTTTTTTCACTAGACATATTAAATTTAAAAAAGTTATTTTTGTAGGGTCCATTTTGGGAGTTGTTACCTTGCAAGAAAATATAATGTATTGTGCTTCAAAACATTTTTTGAATAGTTTTCATGAAGGATTACGGAGGGAGGGTATCGATACATACATAATTTATCCAGGAAAAATTAATACAGATTTATTTCATGAAATGCAAGATTTTTTATGTTCAGATAAGCAAGACATTGGATGTAAAATCATCCAAGACATTAAGAATAATAGAACATATCGATATGTTCCGGCAATTTTTAGATTACTACCAATTTTTACTGCAATAATACCAACATTTATAGTAGATTATGTTTTGCAATATTTTTATTCGAAGTATGGTAAAAAAAAACATTAAGTTTAGATTTACATATTATTATCAGTGTTGCTAGATATAGTTTTTTTAGTTGTATTATTTATCATCCAATCATGCAATGAAATCTTGGAGTTCTTATTTTTATTATACTCTTTAATTAATAACATTAATAATAAAACAATTAAAATGGTTATTAGCAATGTAATTATTTTATCTTTCACTCCTATATATGTTTTGGGAATTTTTCTGATTTCTTCATTAGCCATTATAAGGTACTTGTTTGTACTTTCAAAATATCTATCAAGAGAATCTATAAACAAAGATTGCTCTTTTAAGTTTATTTTTATCTCCATAAGTGTATTAGAGATAGAAAGAAGGCTTTTGTTTATTTGTCTATTACTATTTTTTTGCAGATATTCATATTGATTGTCAAATATGTTAAAATGCGTTACATTAGAATCATTTACAATACTATTTAAAATTTCAAAAGAAGAAGTTTTTTTAATATTTTCGGTTATTATTTTTCTATAGATAAGAATTGCATTTTCTAAGCATTTTTCAAAATAGTTGTAAATAGTATCACTAATAATTTTATTCTCTATTTGGATAGTAAATTGTTTGATTTCAGTATGTATATTATTAAAACTAGTTTCTATATGTGCTTTAATGTCTTTTATTTTTTCCTTGTTTTTTACATTTTCTTTGAAGCTTGGCAGATTATCATAATTAATTAAATTTTTATATACTGCTATATTTTTATCTAATTGACTCAAATGTTTTCGTAAATAATCACAATTTAAATAAACTTCACAAGTCATAGTCTGAATATTTCTCAATTGAATAAATTCTAAACTTAAATTTTTAATCATCTATATTTATTATTGATCTATAATATAAAACAAGCAAAATAAACAGTTTATAATTTTATTAAAATGTTATTTAAAAATAGTTTAAAGCTTGTTTGACAACGTACAACTCTAATAACTCCATGTTCTGATTCAATAAAAAATTCTTGTGGTAATTTATCTTGCAATAACACTGAATCTAAAAAAATGATATTTGAATTTTGTGCATATTTAATTGCAAAATATCTTGAAAGATTCAAAATTACTATATTATGAATATTTGCAGCAGAATAGAATCCATTTTGATAAATATATTTCAATATATTGATGGTAGGCCATTCTTCATTGGATATATTGACAGAATGTCGATTTACTATAGATATTTTAATAGTAAAATCAGGAAAGTTTAATTGATTAATATATTGACATAACTGATTATATTTAATTAATATACTAAATTTATCCAAAGGATTTGAAGATAGATCGTTTGTCAATTGATCCAAAAAATCTATAATAGTATGATTTTTATAATAAATTTTACAATATAAAATATTAGACATAATTTCATCACGTCGATTATGCCATTGAAGTAGGTCATCATATAAAATAGAATTTTTATCATCAATCATTATTTGGATTTTTTGATTATACATTTTGATTTTATATTTTTTACCAATATATTGCTTTAAATGTGAACAAATGTCTTTTTCTCGGATTATCATATTTTCTAAAATTTTAGTAAATTTATTAATAATTACTAATATTTCTTGTTTAAGAAATTTTTTAGCATTATATGTCTCATAAGTAACTGTGTCTTTTACAGCAGTTTTAGCTAATATGCCTTTATAGCTTAATTCAAATAACTTTAAAAAAATACCATTTTCAACTTTTATAGTATTAAGAAAATCATTAAATGTTATATCTGATTTATTAGAAGTATTAATATTGTATTTCTTCCATTCTTCTTCTAATGCTGATTTTTTACATTTAAATATTATTCCATTTTCGTTTGCATTTTCAATTTTTCCAACACAATAATCTTTCAAATTTATTTTCATATAATTTTAGGGCTCCATCATACATCTTTCATGAAAAATATACGAAAAGATGCAAAAAAAATGGAACTTTCATTTTATGACAAATTAAATATTATTATGCCTGGAAAAATTCCATATGAAACATTCAAAAGTTATTCAATAGATCGAATTAAATTATTGAGAAAAATTGAAAGTGGTACAGATAATATATTTACTATAGATTCTTTGTATAAAGATTTAATATCTCATTTTGCATTAAGATTAATTGTAATTCAATCTCATGTGACACTTCAGTGGTTTGTAACTATGGAAACAAAGCTTTTCAAATATAGGATTAATCAGATGAATACATATGAATTTAATCATTTCTACAATACAAAATTTATTTCAAAATTTAATAATTTAACCGAGTCTACCATAACAAACAACATTAAAAAAGAAAAGCAAGTCCAAAAAATATCAAAATATGGACAATATGATCCGGATTTTGACATTCCACCAATAAATTATGAATTTATTCATTTTTCTAAATGTTCACATACAATGAGTAAACGAAAAATAAAATTAGTACAAGGATATTTACCATCAACACCTGAGACTCAAAAAGACTTTGTTATTACAGAATTTAATAACTTTATAGAAAGAGAGATGCATAAACTTAGTAATAACAGTATTAGAGATGAACGACTTATTCGATTGCATAGTGATTTATTTATTAGTTGCAAAAATAGTTCTGACAAGGGAACTCCCAATATTTCTATAAAAAACATTTATCAAGAAAATTTTCCATTATGTATGAAATTACTTATTGGAAAATTAGAGCGGGAAAAGCATCTAAAACATAATGATAGGAATCAGTTATCTTTATTTCTAAAGGATTTAGGTGTTCCAATAGAAGATACCATTAGCTATATGCAAAGCCATTTATATCAAATGAAAAATAAAGAGCTCGTATATAATATTCGACATAATTATGGACTGGAAGGAAAAAAAGCAAATTATTCCTGTTTTTCTTGTCAAAAAATTTTCTCTTTATCATCAGAACCAAATTTCACAGGATGTCCATTTATTAATAATATATCCCTTGCAGAACAATTTATCAATATTGAAAGTGCTGATCCATTAACAAATTGCAAAAAATATTTTGTAACACAATTGCAAGATAAAAATATATCATCTAATAATTTATTAATACGATCCCCAGCTGAATTTTATAAAGCATTGTGTAAATTCAAGAAAAATAAATAATATTCATTTAAATTAAGTCCACCAACTCTGAACTTATTAAGTCAGAACATTCAGAATTTAAGTCTAGCATTCCACATAATTTGAAAAGAACATTTGCCATTTCATGGTTTCGTATTTTTTTGATTATATTAACAATTTTTTTGAAAATGTGTTCAATTATAAATTTATATTTTAAAAGGTTTTGGTGTTTGTCAAAAATACATAACTTATTAAGGGTTTCTGGAGTAGGATTTAAATTAAATTCATAAAAATCAATTAATATTTTTAAGTTTTTATGAAGAGAGTCATCAATAGTATTAATATTTATCTTGACTTGTTCATCCATTAAAATAGCTTGTTCAAACACATTTTCCATCACAATTCCATATGTATTGATATCGATTAAATCAATTATTTTAAGCAATTTTCTATCATCTTTTTTCTTCAATAAATGTACTACTTGAGAAAATATATTTTTATTTAAGCATGGACTTCGTATTATATCGAACAAATCATATTTTGTTAAATATTCTGGAATAAATGGCTCATATTTTGGATTGTTTTGTAGAGTTTCAATCAATTTAATTTTTGAATCAAGTAATTTTAATTTTTGAATCATAGCGAAGAAAAAATTCATGAAAATAGCGCTGATATCCCTATTAGATGATGAATCCTTAACATCAGATGGAATTAGATTTTCAATGTTTGAAAGTTGAAATAAAATAAAAAATTTAGACACCACTGAAAAATTATGTTTTAGACAGAGTCGTGCAGCTTCAGCATAGTTTTTAATGAATAAACAAACTAATAATTGGACTTTAGGATTAGTATAATCTTTATAGTGTAACGGCCTGTGACAAAATAGTAATGTAAAAATGTAATCTTCAACAGAACTAATTACGTATCCATCAGATACAATTTTGTTATACATCAAATTATTTAAAAATATCTTGAATCTATCATCTGTGAACGAAATTAGCGATCCTGTTTCAAACGCTTTACCATTAAGGAATTTACTAAAATTCACTCCAAAATTGTCATGAACATGATCAAAAAAGTATGAAAATTCATCTAATAAAATAAGAATATCAGCTTTACGACCAAGACGTAATAAAACATACACTTCTGCGAACAAATATCTTTCTTCGTATATAGAAATATTAAACTCAGTTTTATTAAACTTTAATTTCACAAAATTTATAATTAATTCTTTTTCGTTTTGAATAGTTACTTTATTAGATGTTAACTCAGTCAATTTCATAAATTTCGTTATAAATTTTTCATAAACAAAACTTAAGAATACCAATGGCGAATGTTGATTTTTAAGTAAAACATATATATCATAGAGAAATTCATCTTCAAAAGTAACTTCTGATGGAGAAACAAACAAATATTCCTCAGTTTTTAATCCTAAATTAATATTGAATGTCATTAATTCAATTTCCTTCATTTTTGAGATAATATGTTTTTCCAATTGCTCGTCAAATTCATTCAATATTGTTTTTTTCATGGCATTATATTTATCTATTAATTTATTGTCTAATACACCCTGGATTATATCTTCATAACAAAATTTTTCGGTATTAAAAGTATATAGGCTAATTTTCTGTGGAATGTATGATACAGAATATTTTTTGTATTTATCTTGTTTAATTTTTAGTAATGCTATTGCATGAGTTACATTGTTAATAATAGGTGGTTTATACAAATATGGAACAATTCTTGAACTATTTTTATAAGACATAGGGGGTAATAAAAGTTAAGTAAAATTCATATATTGAAAGAACAATCAAATTATATTTTTATTCATTAGTATCGTCTGAACCTGCGTTTAAAACTGTATTTAAACTGGAAAGAGACTTTGATGTTACTTTTGTCTCTTCTGTATCTATTAATTCGAAAAAACGTTTATTTTTTATTAATTTTAAATTATAATTGTTATTAAGAAGTAACACTTCAACACTAGAGGGTAACAAACTACAGTCAAATTCAGTTAAATTATTAAACTGTAAATCAAGAACGCGGAGTCGTGTAAGATGTGTTAAATTACAAGTAAATTCTTTTATTTTATTGTTGCCTAAATAAAGTTCTTCAATAGTTGGAGATATATAATGTAAAATATCTAAGTTTGAAATTCTGTTATTTGCTATATCAAATTTTTTTAAATTTGTTAGATGTATGATACTTGACGAAATTTCTAAAATATCATTTGACATTAAATATAATTCTACTAAACTTTTATTTTTAATGGAATTATGATTTAACAATAAATTGTACCCTAAATCAAGAACCTTTAAACTGGTAAATTTCTCAATATTTGGAGCTTGTATTAAATTATCAGAAATATCCAGATACTCAATATTTTCTGGAAGTATATCTGGAATTGTTTTTAACTGATTTCGTCTTAATTCAATAGCCTTATATTTTTTAGTAATTTTTAAAAGTTCACCATGACGATTATTGAAAACTAAAGTCTCATCCTCGCTATAATTATTCATAACACAAATAGGGGATGCCAAATAAAAAATAATTTATTTAATGTCTAGTTCTTTCTCGTGGTGGATGAAGAATTAAACCATCACATTTTAATAAACCTTGTGCTGTTTCGCAAGCAGCCTTCAAAACTCTCAATTTCATGGTATACCCTTCAATTACTTTGTTTGTTTTCATATCTCCAGGTTTGCCAGTTAATATATTTAATCCGTAAGTAAAATTGTTTTTTTGATAAGTTGTCTTTAAATTGTATAGAGATGTTATTGCACTTTTAAATGTATTTGAATCAAATCCACAATTCATACATAAGAGTTCAACAAATTCAATTAAAGCTTGAGATAAAATTCTTAATCCTTCACTTTCTACTGTTTTTATTTCATATGATAGCTTTAATAATTCCATTCCAAGCATGGTCTCAGTATTTCCTCCGCCATAGAGAATAAACTCTGAGGTTTTTATTCGTTTTAATACACAGAGAGCGTCATGAAGTGATCTTTCAGCTTCGTCAAGCAATGATTTACTACTTCCATACAGTAGAATGGTTGATGCACCTCGTTTTATTCCAGTAAACTGAGTAAATTGTTTGTTTTTTATATAAACTGTTTCTATTTTTTCACATTTCCCTATAACTTTATCAGAATCATGTGCTTCAAAAAAATGACTTAATACGGGACTTCCTAATGCTTTATTTAATCTTTCTATATTGTCAAAATCAGCATTTTCGATAACATATTTTTTTTTATTCAATAATAAATGCATTGGAAAGTCATAAATGATTTGCCTGTTAATTAAAACATCAAAGTCTAATTTACATATATCGTTGATCTTATTTTCCATTCTTGTTTTTTCTGCTTGTTCTAGAGCTTCAAGTTCAGCAAGTGATGTAACAGATATTTTACTACTAAATATTTTAATTTTATCATAGTCTAAGGAAGAATTAATCAATAACACTTTTGGATTTTCTACAAACGTATTTGAATTGGTTCCAATATTCAATTTTTTATCCAAAATTAATTTGTTGCACCAACAACTCTCTGACAAATCACCTTCAAGTTTAATGATATTAATCAAATTTAAATCACATGCTTCATCAAGATGATTAATTGCATCAATACAAATGTTAGTAAATAATTGAAGATGTGAATTTAAAATTTTACTATTAAGTGTTGTTTTAACAAGATTTTCAATATCAAGCTTTGTTGCTTTAATCTTCTTATAGTTGAGAATTTCAATAATTTTTTCAATTCCAATAGATAATCCTTTAATAATATGGATAGGTTGAACTTTGCTAGCATATGCATGTTTTAAAATTAAAGCTGCAAGAAGAACAATTGATGTCGTTCCATCACCCTCTTCATAATCTTGCATTTTAGATGCATCAATCAATGCACATGCAGACGGAGCGTCAATTTGTAATTTATTCAAAATTGTTGCACCATCATTCGTTATTTCATGATGATTTTCATCTTGCAAAATTTTTACTGCACCTTTTGGTCCCAATGTTGTTTTTAATACATCAAGCAAAACAAGAACACCTGATTTAATAAATTTTTGAGCATCTTCACCAAACATTTCTTCTGCTCTTCCTGCCTTATTGGTAAACAAATCTGCATGAAGAGTATTCATTAGTAAGGGTAAAATTAAATTACATATAAAGAAAATATAAACAATAAACAGTATATGATTTAATTAAACTATTATGCCATTACATGGTTCTATAGTGTAGTGGACAGCACTCTGGACTTTGAATCCAGCAACCCGAGTTCGACTCTCGGTAGAACCATTCTTTTACTGATTTTTCTTAAATATAGTTTTCGGACCGTCAAAATAATCCTTAATTTTAAAACCCATTCGATTATAAAAATAATTAAAGGGCATTTTGATACTTAATTTAGTATTTACAATATTATTCTTAGCATATTTTATACTATTATCACAATAATTCTTCTCCTCATAGTCTGTCTTCCTCTCTAAACATTTATTTTTTATCTTGAATGATTCTTCTGAATCCATTGTAATAAGGGCAATTATTTTGAATATAAAAATAAATAATGTAAATTGCAACTATTTATTTAAATGGGTGTCCCATATATCGCACTCCAATTCTTCGTAATGCATATACTACATCTTGACAATTGATCGTCTTTCTCTTAGCGTGAATACAATATATTTTGCAAGATTCTAATACTGTTGAAAGGTATGCATTTGTAAGGGCATTAACTTCTGGAAAACAACCTCTTCCAACACGTGTTGCCCCTGCTCGTCGTGCAAGACGACGAATGGATGGACCACCAAGTTTATTTGTTGTTGCCACGTGCTTTCTATGACGCTTGGCTGCCCCACTTTTGGCTCCCTTAGCTGATTTTCCTTTTCCGGCAGATGATGGAACTGGTGTCATTAAAGGGGTAAGCTATTTTTCTATTTTTATATGTTTTTTGGGAGGGAAAAAAGAAACAATTAGTATAAATCAGTATTTAAGGGAGGCTAGATTTAAATTTTTACAATATTTTTTATTAAAAATTATAATTTGGATAAATAGCAAATATTCAATAGTAAAATTTATTTGCCCTAAGCTATGGAAGAACTTATTATTTTGCAGACACATATAGATTATATAACTTCACAGGCATCTATGGAGAATAATCTTCCAACTATAGTTATTTTTGGAAAAACTATAAATGGAATTCCTATTCAGGTTAAAGTAAATGATTTTCTTCCATATTTTTATATTGAAACATCCCATCCAGTTAATCCAAATGAATTAGAAGATGCAATTAAATCAACGACTATCAAAAATAATTGTCACGGTGTTGATAAAGTTTTAAAAAAGAATATCTATGGGTATGTAGAAGATACATCAATATATTACAAAGTGTTTTTTAATACTCCTACAATATTTAGACAAGCAAAAGCAATATTTGATAAAGGATTGGTTATTAGTGGTCAAGTTGTAAAATTCAAGACATTTGAAAGTAATTTTCCATTTGTGTTAAGATTTATGCATGATATTAACTTGGCAGGATGTTCATATATAAAAATTAAGGTGTGGGAACCAGAAAATACAGGAAATGGGTTAATCATCAAAACGTCACATAAATTAATTGAAGTGTTACCGTTTGAAGGTAAATATAATAGCATTATTCCCCTTAAGATTCTTTCTTTTGATATTGAATGCTGTGGAGATACTAATGCGTTTCCATCTGCTTTGTTAGATCCAGTGGTTCAGATTGGAAATATAATTCAATCTACAATAGGTAATGAGATTAAGCAGGTAATTTTTTGTTTAAAAGAAGTTGCAGATATACCGGATGCTGTGGTTCATTGGTTTGATAAAGAAGAGGACTTGCTTATGGCATGGAACAAATTTATTCATGACGAAAATCCAGACATTATTCTTGGATATAACATTAAAAATTTTGATTTTCCATACCTGTTAGAAAGAGCAAATATTTTAAAATTGAAAGAATTTGCAAGACTTGGCAGAACTAACAAGATATCTAAATCAATTAAAAAAACACAAAGTTCATCTCAATTTGGTTCTTTAGAATTTAATGATATTACTATTGAAGGTCGAATAATTTTTGACATCTTTCATATATTAAAACGTGATTTTAAACTTAGATCCTATAGTTTAAACTCTGTATCAATGCACTTTCTTAAAGAACAAAAAGAAGCTGTTTCATATTCCTCCATTCATAGTCTTCAAAATGGAAATAAAGAAACACGCCGAAGAATAGCAACTTATTGTTTGAAGGATGCGTATCTTCCATTGCGTATCTTTAAAAAATTGAATATATTTACGAAATACATTGAATTAGCACGAGTTACATATGTTCCTATTGAGTTCTTTTTAACACGAGGAACATCGATTAAAGTTCTTTCACTAATTTATAAAGAAGCTAATGCATATAATTATATCATCCCAGATATGGAGATTAAAGATGGAGATACTAAATATGATGGCGCATTTGTAATAAATCCATTAAAAGGATTTTATAAAGATCCAATTGCAGTACTTGATTTTTGTTCATTGTACCCATCTATCATCATTTCTAACAATTTGTGCTACACAACATTATTGAATGACAAAATGGCTGCTAGATTTAAAGAGTTAGATATTATAAAAACTCCTACAGGGGATTGTTTTGTAAGTTCGCATATCAAGAAAGGTTTATTGCCAATAATATTAGAGAAGCTTTTAAGTAATAGAAAATTGACTAAAAAGTTGTTAAAAGAAACTGAGGATCCAGAATTACAAAAAATTTTGGATGGAAGACAATTAGCATTAAAATTGTGTGCCAATTCAATATATGGATTTACAGGAGCACAAGTTGGACAGCTTCCATGCATATCAATATCTCAAAGTACAACAGCATTTGGAAGAGATATGATTTCTAAAACTAAAAATTTAATCGAAAGTAAATATTCTAAAGCTTCTGGATTTACTCATGATGTTATAGTTATTTATGGAGACACAGATTCAGTCATGCTTAACTTTCAAGAACCAAATATTGAATTAGTATTTAAATTGGCAAAAGAAATTGCTGAAGTTATCACTGCAATATTCATTAAACCTGTTGCTCTTGAATTTGAGAAGGTATATTGCCCATATCTATTAATGAATAAAAAACGGTATGCCGGCCTGTTATTCACAAATCCATATAAATCTGATAAAATTGATACCAAGGGAATTGAAACTGTTAGAAGAGACAATTGTGAACTTGTTAAAATAATAATTGACAATTGTCTAAATAAAATTTTATATGAAAAAGATATTCAGGGAGCGATTGATTTAATAAAAATGAAAATTGATGAGTTGTACAATGATAAAATTGACATAAGTCAGTTAGTGATTTCCAAAGCATATACAAAATCAAACTATAATGTTAAGTCGGCTCATACTGAACTAGTGAACAAATTGCGTGAGAGAGGAGTAGAAATTCGTATTGGTGATCGAATACCATATGTTATAATCTGTGGTGATAAAAAAACTCAGATTTACAATCGGTCGGAAGATCCTATTTATGTACTAGAGAATAACATTCCAATAGATATTGAATATTATATTGAACATCAATTATCAAATCCTATTAAAAGATTATTTGAGCCAATAATGGACAATGTGGAAGATCTTTTTAAAGCACGCACAATTAGTGGGGATAAAGAGTTAACAGGCCCTATGACTAAATTTATACAAAAATTAGATCAGTGTATTGGATGTCAAAAAGTGGGTACTATTATTTGCGATAGTTGCAAAGCTAATTTTTTTATACATCTATCTCGAATTCAAGCGGAATACAACATGAAATTTACACAATTTCATGAATGCTGGACAGAATGTCAACGATGCATGAATAGTGTTATGAATAAAATAATTTGCATTAATCGTGATTGCCCAATATTTTACATACGAACAAAGATTAAAAATGATTTGAATTCAATACAAAAAAAAATGATTAAAGTGTATTCTCTCCAATGGTAAACTACAATTTAATAAACTTATAAATTAATATTAAAATTTTGTTCTTTTATGATATACATCATCTACAACTTTAGAACCATCTGCGATTTTTTTCTGTCGTTCTTTATAGCGATACTTAATTTCTGGTAAATAAGATATTAGCTCATCTTCTGTTATTAATGCATCTAAGGCTTCAAATTGATCTAATATGATTTTAATATTATTTGGAGTAGCTTTATCATATCTTAATTTATATAATTCCCAACCACCTGTCGAAAGTGATCCATCTTCAAAATCAAATATTTCCTTAGTAGAGTTCCAAAAAAATTCGCCAATATTATTATGAAACATTGTATTACTATCATCATCACATATATAATAACCTGCACAAATATTCATATTATTATTAATTTTGAGTTGTTTAAATTGTGATGATTCTCCAAGAGCATAGAGTGTATATATGTACGGAATACTAGTTTTTTTTATCAAGAAATCAATAGTATTAAGTTTTGGAGGTTTCCATTTTATAATTTTTGATCGTTGATATAATACATATGGTTCATCCACAGGTGTGAAAATTAAACCATCGTTCTTATGATTTAGTTTAGAAATATTTTTTAAAATACTTTCACATCCATATGATTTAATCATTTGTTTTGCCACAATACGGAATTTTGTGTATTTATTGCTAATAAATTTAAAATAATTATTATTGCAATAATTATTAACAAAAAATGTACAATAATACAATCGTTTAAGTAATGAATAATCAAGGACTCGTTTATTAGCATACACTAAACAATCAAAAATGGACAATATATATTCATTATTTTCTTCATATAGCTCTCCATCGAATAAAAAATCAACAGAATTTAATTTTTCGAATATTGCATCTGTTTTATAAATTTGATCATTTCTATCTAATATAAATACAGTAGAGTTATTTATATATAAAATAATTCGAATCCCATCTGTTTTTTCACAAACTAAATAATTATTGTTAATAATCCATTTTAGTCTTTCTTTTGTAATATTGACGGGATGATTTCCAATAAAACCTTTAATTTCATCAATTTTAAGGATTCTAGCCAAATTCAATCGGATACTATTTTTTACAAATGGAGAATTTACAGGTGGAGTACCAAACATAGTACTCACAAGTTCAATAGCCATATAAGGGGTTTAAAATTTAAACATAATATTTCTTATTAAGTTAATGAATCTTCCAAAATGATTTATTAGCTATATTATTATATAATAAAAAATGATTATTAGTAATTGTAAAAATAAAATTATTTTACATCCTTATGAAATACACTTCCAAATCATTATTCAAAAACATTAAATATCTTTCTAGTGAAATAAATACAAAACTAAATACTGAAATGTTAGAATACATAACATGTAAAATAGAGGAATTGTTAGTAGAAATTGTCAATGATGCAAAGACTATGGTATATAAACACAAGAGAAAAATAATTCAAGAAGACGATATAATTAAAATTATTAGATATCGGGGCTTGCCTTTTGATATAAAATCTGAAATTAATGATAATAATGAATAAATAGATTACAAGGCCTGGCTAGCTCAATCGGTAGAGCACCAGACTCTTAATCTGGGGGCTACGGGTTCGAGTCCCGTGCTGGGTGTATAATGGCCAATAGGCGTTTTCCCTTTTTGCAAATTAATATTTGATAATTTATAAAGGAGACATTTTATATAATCATGAATTTAGATCATGATTATCATTCTAATTTTATTCAAAGTGAAGCAGTATTTTGTGAATGCAATATTAAACGACATTTTGTACATAATAAAAAATGTATATTAAATCTAAGACCTGAAATATATATGAATCATAAGGTTAATACAATGAATGTGGATATTATTGGCTCCAAATGGGTTTTTAGAATCCTAAAATTACGTTATATTAATAAAATTTAATTAAAATCTTAGATTCATTTTTATATCAAATGTAATAATGAATATAATATTGTCTTCATAAAAATTTCTACCCATGTATGTTAATTTAGGAAGTGCTAAGCAATTACAAAAAGATACTACGTTTAAAATCGATTATAGAAATTTTTTTTATGGAAAAAAGCGAATAGGTATTGGTATTTTAATCATCTCAACCTTTTTTGTTTATAAATTTATCACACTTCTGCTTAATGTGGCTGCACTATATAATTCGAAATTAGTATGTGAATATTTGAAATATTCTACTGATACTCCAAATTGTATATTATTGCACTTTCGTTATGAAAATAAAAAATCACCTTTGAAATTTAAAATAAGTAATTTAAAATTTATGATTAATAATAATATAGTTATTCATTCAGACAACATTGATATTAATGAAGAGGATGAACTTTTATTTAAAATGAAATTTATAATTGACCAATTTCAAATAAACCAATTTGACTGGAAAAATTTTCCAATTATTATTGAAGCAAAAATTATAGCTATATTATTTTATTACATTCCTTTTACATTTATAATCAATTATCAAAATCACATTGAAATATTATCAAAAGCAAAAACACAATACGATTTTTATGAAAATATTGGTCTTAAATGTACCAAAGGTACAACTAATAGACATTTAATATGTTATATAGATGCTCAAAAATTTTTAAATTCAATATTTACAGAAGAAACTAAAATCGATATTAAAATTGATAATGTTGACTTTTTTTTTAAACCTTTGATATTTGAAAAAATTAATCTGCAACAATTTAATTACAATAGTGAAATTAAAAAAAATGAGCATATTGTAATAAATGTATTTATTCCACAAGACAATGATGTTTTTGGACATTTTTTTATCAATACCATTAAAAGAAAAGAACGCTTAATTGTTAGTCATATTATATTTAAAGATATTTTAATTCCTATTAATTTTGACCCAAGTTATCAAGTAAAATCATCAACAATTTTTTTAACTAAAGAACCATTTATAACATTATCTAATATACAACTTGGGACAACATTGTGTTTTTCTATTACCTTTGGAAAAGATTCATTTTTAGATGGAATCTATTTATATATATTTTATTTGTTACGAACATCGACAGTACAATTTTCTGGATCACAAGATGCACAAATATTTGTAATAGGAAAAATAACTTTTGAAATGTCAAAAATAAATATTGAATTAGAAATATTGAATCCAACTGTTATAGGACGTCTTCTACTGTATTCAGAACCTCGTGCTGCATTTGAAATTACCAATTGTGAAGAACATCCAATTTTAGCATTATTCAAAGATATTAAGTTTATTTCAAATTTAACATACTATCATGAATTTTATTTTAGAAATAGAGTAGCTCAAGTATTAGAAACACAAATTGAATTTAGCAGTGAATTTTTATTGGATCATATAATTCAAGAAATAACAAGTGATGAAGAACTTAAAAATCCTACTATAATTACGATTAAAACGAACATTAACTTATTACCCGCAAGTACTATAGATAAACAATGTTTATCTGAAGTTGTAATTATTAGCATTGACAAACGCATTTCATTCAATATAACATCAAATAATATTAATGAAGGTACAATTACTATTTTACCAAATATGATATATTTTGAAATTAAACAAGATTCAATATCAAATAAATTATATGGTGAATTATCAATAGAGTTTATGTTAAAACTTAATATAAAAGCGCTTGACTTAATAGTTGATAATAATATGGGATTTTTAGACACATTATTAGGAATGCATGTACAGTTTGAATTGCCAAATAAAACAATAATTGAATTTAATGATCTTTTTATTAATTCCGAAAATATGCCGTACACAACAACAATTGACATAAGTAATTATAATGATCGATCTTTATGGTTTAATACTGAAACTTCACAAAAATCGTCACTTAACATTATACTTAATCATTTTACTAATACTTTAAAAAATATAACTTCTGAAATGCAGTTTGAGTTTGAAATTAAAACTGACAATAGGATTCATAATGGATTTGATAATTTTTGTAGTACTCTATTTCCTCATACAGATTATAAATGTAGAATATTACAAACTTTTTATATAATCAAACAATCTATTCCATATTCTGCATATTTAAAAAATACTATAATTGATATGTCAGTTCTAGAAATAAAGACTAATGAAGCATGTTTATGTGTTGATTATGTAGATAATTATTTTTTCATAACAATACCAACGCCAATTAAATATAGCATACAATTTAAACATATAAATAGTATAGGAAGTAGTCTTAGTTTTATTCCATCTAATAGGAAAATACGATTAGCGGCAATTTTATTGAATGTTCTTTTGGCAACTTTACTTAAGGATAGTACAACTGTGTATACAAAAATAATTAGATTTTGGAATAATTATGGAGCTAGAATTGATATATACACACCAGAAATCCAAAAACTAATTTTTAAAATTGCACTTGATATTCCACAGGAATTAATAGATTCGAGAACAATTATTTCATTTTATAATTTTATTCCTCTACAATTAGCAATAAATTCAGAAACAACTGGTCATAATCTTATTATTATAAATATAACATCAATACAAGATGATTTAAAAACATATTCTATTATATTACAAATGGAAATCAATTTAAAGGATGAGTTAATGGATCCATTATTAAAATTTAAATTTATAGTAGATTCTGTAGTATGTAAAAAGATTGTATCTAAGGATTCATTTAAATTGACAGATATGTTCAAATTTGCAAAAATATTAATTAGCACAGACTATACTACTTTTGTTTCTACATTAACTGTTGAAAAATTAAAATTAATAATTTATGATTTAACAATAAATCAACATGCATTTACAGATATTTCTGTATTTTCTCCTACAAATATTAAAGACATATCATTGCAATTTAAATATTATATTGACTTAACTCCAATTATTAATAATATTTCTACCATTATTTTAAATTTACTAGGCTCAAATATTGTTCCAAGTACATTTTATTTAAAATATAATATGCATTTTCCAGCAAGTCTTCTAAGATATAGTTATAATCAAAGTTTTTTTTTTAAAATATCAGGATTAAAAGCAAATGGTAATTTTAATTGTAACTTAGTTTACAATAAAAAATTTTATGAATTAAGTAATACTACTTCTAATGATTATTATAATATGTATGGTAATGTCCATACTGCAGTATTACTATCTCAGTCTCAACAAATTATAAAATCAGTTTATTTTGCAGGAAAATTAATAAATGACTGTTATGGAGATAAAATGTGTTATGCAACTCAAAAGCACTTAATTGAATATATTGATATAAACAAATTGCAACTTAAAAATTGTGATGGAATTTTTTATAAAAATCCATATTTTAAATATAATAATCAACAAGTTTACACTTATGTTGATAAGACTCAATTTTTAAATAATTTTAATATTAACATTATATTCAAATCACCAGATGACAAAAATTTTGCATTTGATTTTTCTCAAGTAAAACTTATCGGAGGATTATTACCAAAACTAAATAATATTGTAAATTCTATGTGGAAAAATACACATAGTACTTCAATGCCTTTTTTCCCATTATTCTCAAATACTTTATATAAACTAACAGGAATATGTTTACGATGTCCTATAGATATAATATTAGAATCACCATCAAATTATATTTCAATTGTAATTAAAACAGGCGCTGGATTATGGGGATTCATTGATATTCAAGATGCATTAAACAATTATGATATATGTATAGATGGTTACAATTGTTTCATTCCAGTTCTTATAGGTAATACTATTATACCTGAATCACACCAAATTTGTACATATCTAGACAATCAGTTTAAAAATAAAGCTATGATATTTTGTAATATATCAGGTCAACTATTTTGTTTTGATTTGAATAGAGTAATTGACTTACGCATATATGGAATCAGTATGGCTATGGCTACAAATTGGATTATAAGCAAACAAAAATTAATATCTGTATTTGATATTATATTAAAAACATATACACATTTGTTTCCATCATTTTCATATCCACAAACTATTATGGAACATAATACAAGATATTTACAAAGTATTATGGATTCCTTCCAATCTCATCATCAAACCACAAATTCAATCTATAAATTTCTAAACATGTGTATTTATTTTAATAAGATGGAATGTAGCTATGGTTCACAAATAATTAATAATTTTATTAGTATGTTAAAAAAAATCAAAGCGGTATATAAATAATATATTTTAAACATTAAAAAAGTTTATTTATCAGTTTCATCACTACTTAAATTTTTTGATAATAAATTAAACAATAATTCTTTTTGTTCTTGTTTAGTTTTATAAAGTCTAGTTTCATTATAGTACATATCATTAAATCCAATGATTGTTTTGAAAGAATATCCTTGGTCAATTAAAAATTGTTGTCGCTTTGCAGAATAATGCATTTCCTCTGTGTCTTTAGAAACAAGAGAATAAAAATATGCTTTAAAATTTGGATTATTTCTTTTTTTTGCTCGTAATACTCGTCCAAGTCGCTGTGCCTCTTGTCGTCGACTTCCAAAATGTGATGAAATTTGAATTAAACATGTGGCTTCAGGTAAATCAATTGAAGTATCACCAACTTTTGATAAAAAGAGAGTATTAATTTTGGAATTAATTTGAAATTGTTTTAATATTTTCATTCTTTCAGTTTGTGATGTTGGTCCATATATAAATGGTTTTTTCATTTTGATTGCATATTCTTTTAAGGCAAATACACTGTCACTAAATACTATGATTTTATCTCCTTGTGCTTCATGCTTTTGGATTAAATATTCACACATTTGAATTTTCACTGGATTCATTATAGATAAAAGACGCTTTTTAGATGTATCTTGAATTAAATATTCTCTATAAAATTCAGCAGTCATTTCACACCATACTTCAGAACATTCAACTTTCGCAATATGACCTTTGTCACTTAAGTCTTGCCAGTTCGCTTCATATAATTTCGGTCCAATTAAAAAATTAAGATCTTCGATTTTATCATCCTCACGAACTAATGTGGCTGTTAGTCCAAGTTTACATTGATGTAATACAGAAGATACAACTTTTCTAAACATTTGAGCGGGCACAACATGTACTTCGTCTAATATCATTATTCCCCAATTGTAAGCAAATATTTTATTTATAATTCGTTGAACATCATAACTTCGCTTTCCAGAAAAACTTAACATAGAATAAGTAGTTATTAAAATTCCACCTTGAGAAGTATTTTCAACATTATAGTTTTCAAACCATTCTTTACAATCAGATGTAAAGCGAGATACACTATATGGATTAATTGTAGTGAAAAGTAATATACTTTGTTTCCATTGTTCTACAGATACTGCTGAAGTACATAATATAATTGCAGACTTTTTTATTGTTGATATTGCTGTTATTCCTACTAAAGTTTTTCCACTTCCGCAGGGTAATACGATTATTCCTGATCGGGCACGTCCATTTCCAAGCATTTTGTTCAATGATATCTCTTGATATGATCTTATATGGCAAGATGGCTTTAGATCAATATGCAGATTTTTAATCATATTTACAGACTCATATGCTGTGTAGTCATATTCATCTATAAGGGGATAGTCAATTTCAATACAGCGCTTTTTTATCTTTTCGACGTTTGTAATTTCTATTTTTAATTCATCTATATTTGAATTAACAATAAACGACTTTAATATTTCATCTGAGGTAATAAAATTGAATATCTTATCGTTATATACTTCAATAAAGAATTTTTGAGATTGTCTTACAAGTTTTAATTTTCCGTACGATAGTGTACAATCAGAAATGAGATTTTTCACATTTCTAGGTATAATATTTTTACTAAATTTTTGTAAAGTATTTGTGATTTGATCGGTAGTTAATCCGACAGATGCTGCTGCAAACAATGAATATGGAGTTATTTGGTATTCATGAATATTTTCTGGCCGCGATATTGGTTCTGCAATTGCAATTAAAAAATTCGTTGCTTGTTGAGAATTTTCTCGAAACATTTCTAGTATTATTAATCCATCATAATTAATCCACAGGGGACAATCCTCTGAATTTTCTTTCATAATAATATCTTCCTTTGCAAAACAATATCTTACTGCTTTCTTAGGAAGTAAAGCATTAAGTTCATCTTCCATTGAGGGGCAATTTATTTGATTTTCTTCACCTTTCTGTAAATAAGAATAGAGGATGAAGTAATGGGTATATTTGGTTCAATATATAATATGCATCCGGGATTTTCAATTTTGTCCATATAATTTGGTGCAGACCATATGGTTAAACAACGATCATGATGATCAAACTTATATCCCGTTAAAATCAATTGATGTGATCGTATAATCATATCTAAATTGTTGTGTATTAAAAATGCTTTTAAAATATCAGATCCAAATAAAACTCCGGATCCTCGTTGGTTTGGCTGACATCCTAATTGATTATAAGGATCACTCCAAAATAAGTCATTCAATATGTCTTCATTAGCTATTAACATTGTACGATCTATTCGTTTAATTTTATCTAGTGTAATTTTGTTAGAAATTCCACCATGAACACAAAAATACTTGGAATCTACAACAGCGGCAATACTTAAATAGTTAAAAACATCATTTATCAAATCCCATATATAATCGTCTTGATATAATTGAATAATTTCATCATAAAATCCATATGTTTTATTAATTAGTCTTTGTTCATGATTTCCACGTATCAAGCAAACATTTTTTGGATATAATATTTTATAACAAAATAATAACATAATTACATTTAAAGACTCTGGTCCACGATCAACATAATCTCCCAAAAAAATATATTTGATTTTATCTGGTGTGTCTTTTAATTTAAATAGACAGCATAAATCTGAAAATTGACCATGAATATCTCCCAAAACACATACAGGAGATGTTATGAATGGAACATTTGGTTCATTTATAAAAATTTCAATTACTTTTTTGCAAACTATTTCTATTTCTGCTTCGTCAATTTTTTGTTTGTTGTAAATTTTATCAATTAATTGTTCAAAAAACATTCTGGGGATATTCTAGAATAATATTCTGTAATTGAAATAATTGTCTATGAGTATTATCAAGAATGTTTCATGTTTTAAATATAAAACAAATTGTAGTTTATGAAATGGAGCAAGACAATGAATATTTTGATTATTTATTTAAAATAGTTCTAATTGGAGATAGTGGAGTTGGTAAAACAAATATATTAGCAAAATTATTGTCTAATAAATTTTATGAAAATAGTAAACCAACTATTGGCGTAGAGTTTGGAACTAAATTTTATAAGTTTGAAAATAATAAAATTAAAATTCAGATATGGGATACTGCTGGACAAGAACGATATCACGCCATTATTTCTGCGTATTATCGCGGGGCTATGGGTGCAATAATTGTATATGATATTACAAACAAAGAAACATTTAAAAATGCTATTACAATATGGATGGAAAATATTAAAAATTCCTGTAATCATCATATACCAATTATGTTCTTGGGAAATAAGACTGATTTAAGTAGTCAACGAGAAGTTAAAATGGAAGTTGCTTTAAGTTCTATAGAAAGTTATGATTGTAAATTTTTTGAAACATCTGCACAAAATGGTCAGAATATTGACAGGGCATTTGAAGAATTTATAGAAATAATATACCATAAAGAAAAAGGAAAAAATAGCTTAAAAAATAAATTATATGTTCGAAGAGAAGATCTTAATGGAGAGGAATTAAAATTTATTCAAAAAAAACAAAAGAGTGGATGTTGCTAATTTAACATAATATTGTTTCCAAATAACTCTCTTAATGCCATAGTAGCATAACATGATTTATTTAATGTAAATTCTACAATAATATCATGTTTATTCTTACTAATACGTAAGTCTTGAATTTGTTCTATAATTTTACGTTTTCCACCTTTACAAAATTTACTTGGTAATTTGACCAAATTAAGCTCTTCATTAATATTGAGCACATCAGTTAATTGTATACGTTCATTTATAGCACCATTGAATATATAACTTTGATATGCGTGCAAATATAGCATTTGTGAATCTCTATTTAAATTGAAGAATATACTTTTAGCAGTTTTTCCTTTATGTCTTTGAGATTCAATAAAACGCTGAATTTTTGATTTTCCATTATCATGTGTTTTATTATGAAAAACATCATTATATATCATATCTATAGCTAAATCATATTCTTTATTGATAATTAGTTCACCTATCTTATGATTATTGCAATTCTTTCCAAATCGTTGTGGTCCAAAATAATTTAAAAAAAACTGTTTTGTAGGAATAATATTATCACAATTTACATCACGAATTGTAATTTTAAATCTATTTCCTATGTGATCTCCCATTTTAATTGGACTACCTTTATGTATATCAAAAATGTTAATCATATAATCTTTTTTTTTAAATTGAACAATATCTGATGTCTTTATAAAATTAAATATAGTAGAAAATTTTTTCAAACAATCATCATCATTTTTCAAGTGACAAGCTAAAAGAAACAATTTGGTAAATGACACTTTACAACCTATTTTTTGATATGTAATGGCTTTTTTATCTTTATTTCCTGAAAAACTAAATTCTTTATATGGAATATGTAATAACATCGATAAGATTCCAATGGCATCTGACGTATTTTGATTAATTTTTTTAAGAGTAAAAACAAAATAATCTTCGTCTGTGTTCTCTGTTACGAAATTCCCATTGACAGTTTTTAATTTAATAAATGGAAAATATTTACTTTGCTCATATATATTTTTTCTTTCTTCTTTTGTAGGATTTAATGGTAATGACATAATTCGATTATGACTTATATGAAATTTCGACATGTTTACGATGTCGTCATCTATTAACACTTCATTGGATTTGTAAGTTTCTATGACTATAAAGTCTTCACATTTTCTTTTTATTGTTGAAGTATTTATTTTGATAAAATGCGGAGTATAGTAATGTTTAATTTCTAAATCTTCATACATATTGAAGGAGTACTACAATAAAATTATTTTTGTAAAAATTTAGCATTCTTCTCTGCAATTTTCATTAGTAATATTATACAGTTCATTACCATATCTTAACATTTTTTCTAATATAAAGCATGTGTGAAATAAAATCCCTATTGGACCCGGAGTGGCTACACAAAATTCTTTTCCTAAATGACATTTAGTATGTATTTTATAGTTAAGCAAGTTACATATACTATGATTAGTGCATTTTCCTTTATATATTGAATCATTACATAATCCTATATGATAAACTTTCTGATCCATTAATGGAAATTGCATTTCCTTAAAAATTTTTTTTTCTCCAGGAGCTGAAAATGATATCGTATAAAGATTATTCTTTGCCCCCACTATGCTAGCAATTGCACCTCCAAGAGAATGACCTGTTAAAATGATAGGCTTATTTTTATAATAATTTTTAAAAAAATTAATCATTGTCTCTATTTCTTCAATTAATTCAGATATTTTTTCTTTATTAACATTTTTTTTACAATTAAAAAGAATTTTATCTATATAAAAATCGTCTATTGCAGTTTCACCCATTGAAATTCCAAAAATTATTGGTGTTGTACCTTTAAACGCCATAACTAATCCATATTTAGTATCATATATTTTGCAAATAAATCCAGATTGGCCATGTATTCTGATTAAAGACTTAGAATCTATTTGAGGATTGTATGCTCCACGAGCCATCATAAAAAAATCTAAAATTTGAGACCTAATTTCCTCCGAAATTTGATTTATACTTATATCCTTGGATATATTTTCAAGTAATTCCATTTCAGTCTTAACTGCAAATATAAAAATTTTAAGTACTACATTTAAAAACAACATGTATAAATAATCTAAAACTATTTTATTAAATATTTAAGTTTTTATCAGCATTAATAGATGCTTTTTTGCTAATTCCTATAACCGCAGATGCCTTAGTTTTACTTATAGGTCTGGTTTCTCCACAAATTACAGTATCACCAAGTTGCACTAATCCATTCCAACATGGACTAAGATGAACATTGATTTTAGTATTACGTCTAGCATATCGTTTATATTTTTTATCGTAAAATAAATATTTGATTTCAACCACAACGGTTTTTTCTGCTTTCATCTTAATAACTTCTCCGGTAAAAATTCTTCCACGAACAACTATATCCCCAGTAAATGGACATTTTTTATCAATGTAATTTTCATAAGCTGTTTGTGGAGTTTTAATTCCGAATCCAATATTCTTAACTATTCTATTTTTCAAAATCATTTCAGAACTAGCATAAGGATTGTGTTTAACTCCATCTTGTTTTGGAAATACTTTTTCTTTTATTACAACTTTTGAATTCATGGGTAAATTTTTTATAGAAATCAAAAATAACTGTAAATCAAATGTTAATATACGGTTTGAATAGAAAAACTATACAAGTTAAGTAAATAATCAGATACACTTTGTTTTTAGATTTCACACAAAAAATGTGTTTTGAACCCCCTTAATGTTAAAAAACGAAAAATCACGAAGTAAAGCATTCATATTAAATGATTACAAACGAATAACGAATTCTAAAAACGAACAATATGAATTTAGTGTTGGGCTAATTAATGATAATATTTATACGTGGGAGGTCATTATTATTGGACCTCAGGATACTCCATTTGAAAATGGAATATATAAAGCGGAAATGATTTTTCCAATTAATTATCCTGAAGCACCTCCTACATTTCGTTTTATTACTAAAATGTGGCATCCGAATATTGATAAAAATGGCAATGTTTGTATTAGTATTCTTCATAAATCAGGTAATGATGAATATGGATATGAAGATCTTAACGAGCGTTGGCTTCCAGTAAGAACACCAGAATCTGTCATACTAAGTATTATAAGTTTATTAAATTCACCTAACTGTGAAAGCCCAGCAAATTTAGAAGCATCTCAAAACTATAGAGAAAATATTGATGAATATAATAAGAGAGTAAGGAGATATGCAGAAAAAAGTTTAGAAGAATAATATTTATTATTTCGCATATATCAAATCAAAACCATATAATTTCGAAAACTATATTAATTGTTTTTATTGGTCAAACTACTCCTTTTACAACAAAAATATTTTTTTAAGTATTTATTTATGAATGATACTTTGGATTTGGGGAATGACAGGACTATTACATCATTATTTACAGGAATCAATGCATTAAGCTGTATAAGATTATTAATAGTGATAGAACTACTTATAAAATTTTTTAAAATTGGGAGTATTGTTGGAATCTTATATTATAAAAGAGATGATGTAATTGATCAAAATTTAAAAGTTTTTTTAATAGTATATAGTATTTTAACATTTTTAAAAGGTATTACTTTTTACAAAAAAAATAAATCTTTCTTTCAAATTAGAAGAATTCCAGACTATGAAGAAAATAGTGATATTGCATTAATCAATAATGTTTTAGAAGCAACAATGCTGTTTTGGTATATAATCGGCGTGCATTGGTTACAAGAATGTGAAACGTGTAAGATCACGCAACCGTTGATTTATTACTCTGTTTTAATATGGATAATACTAGGCTTTTTCACTTTTTTAGCACCATTATTGGCTATTATATTATTGTTATTTTTAATGTCATATATAAAACCAAAGCTAACAACGCTTACATATAAATCAATAGCTGAACTTCCTGATGAAAATGATCGTTGTACAATTTGTTATGATGATTACAAAGTTGGTACCGGAATTAAAATTTTACCTTGTAATCATCACTTTCATTCAGAATGTATTGATGAGTGGTTCAATGTAAAAGATTCATGCCCATTATGTAAAAAGAGTATAAACTTACTATATGATTTAATAGATACTGAAGATCCTGAAATTTAAATATCTATATATTAATTTAAATTAAATTGTCTTTAACCTATTAAATTTTTTATCCTTTAAAATACCTCATCATGTTTCAAATGTTAGAAAATATATGTAATCGATCAGAAATACGACGATATATTAAATTATTTTATACAAGTCAAAAGTTTTATATTATTCAAATGATTTACTTTAGCGTTATGTGTCTGTATATATTTTATAAGTCTGTAAATATGTTACATAATAATGCGTCTTCATATTTATTGACTTACTTTGTTATTATAGTTGCGTTCATACTGTTTAAGATGTACTTAGTGCATAAGGGTGTATTGTTTACAATATTTCCAAGCATATTGGTCTGGATATATTTTTCAAATACGGAAGAATATCATGAGCAAATGTCCATTTTTTTCTTAACTCTACATCCACTTCTATGGACATTAATTATTAAAAATATTACAATTAATGGAGAATACATACGCCAAACATTTATTATGCTATTTTTTAACACATTATCATTTATAATGTTTCCTATTGTTTTTGAGATATTGATATTTATTTATATAGTGATAATTTTTGAATCTGCAAATATTATAGCTGGGATATCTATCATCAATGAGGTATTGGAAAAAGAGTTTCGAGATAATTGTATTGAATGCCTTATCTGCAAATGTGAATTTGAAAAAGACGAATGCATGATTATATTGCAATGCAAACACAAGTTTCATGACGATTGTATAAAAACCTGGTTTAAATTTAATAATGCATGTCCTTTATGTAAACAAACATTTTTACAATCAGGTGTTGAAAATTCAATTAGGGAAATTATCAACAAGGAAAAGTGAATAAAATTTTTCCCTAGGATGAAATTAGATTTAAATTTACTTATTACTGCGTACATAAATGAAAAAGCAACTACCAAAATTCTACCTTATAAATCTATTGTTGATGATTTTATATTTCATATTAAGTTTAAATGTGATGCTGATATTAAGCCTATAAATACTGTTTTAAACGAATTGCGTGAAATAGAAATTAATCGAATTAAATATTTCATAAAAGGATATCTGCATTGCAGGCTAGATAAAATACGAACAAACATTTTTATTAATGATAGTTTTTTAAATTTAAAAGAAAAAATATTTAGAAGTAAATATCTGATGTTACTTGAAGATTACAATTTACCAATAGATGAATCCAAATCTACTAGTGATGTTGAATTTGTTGGATTTATAGTTAATAAAAATACTGAAAAAGTTATTTTAGATGGTGAAATTATTGATTTAATCATAGGCGATTTTTATATATCTAATATTGATGATATTTTTGATAAATTACTTAGTAACGTAATAAGTCTTGTTTAATTACTTAAATAATATGATATGAAGAATTATAATTGAAATAAATATTGTAAAATTTACATCTATTTTGGTATTATTTTATTTAATATTTAGAATTAACTCACTTATTCATCATTCATTGCTTTTTCATAAATTAATTCATCATTACTTTCATTATCTACAATGTAGTTATAATTTTTTGTTATTTGGAGAGATGGTGCTGTTTTAATGTTTTTTAAATTCTCATAAATAACACTTATAATAGAATTAAGATCTTCTGGAGTGTTCATGTTACATGAATTCTGTTTTTCAATTTTTAATTTATATGTTGGACAGTAATAGTCCATAAATTCATTATAAGGAATATCTTCATTTAATTTAATACCGCATACAATACCAGTATCGTATGTCCATGCACGAGCTACATTTTCAATAGTATATCCTCCTCCACCAAGAACCATCATTGGGAGATTAAACGATTTTACATATTCAACGCACATTCCATGACCCTTTTCTGTCAAATTAAAACATCCTAATTTATCGCCTGATAAACAATCCGCCCCACATTGCAATACTACTGCAGATGGTTTAAACACATTCATTATTCTTTCAACAATTGGCTTAAATATCATTTCATATGATTCATCATCCATCCCGTCTTTTAATGGAACATTAACAGCATAATTTTCTCCCTTTTCTAATCCTTTGTCATCTAATCTTCCTGTACCTGGAAAAAAATCACCATATTTATGAAATGAAAGTGTCATAACACGATCTGTTAGATAAAATGCTTCTTCAACTCCATCTCCATGATGGACATCAATGTCAATATATAATACGCGATCATGAACCTTTAATAACTCTAGTATACCTAAAACTATATCATTAACATAGCAAAATCCAGATGCCTCTGATCTTTTAGCATGATGTAATCCTCCAGACCAATTGATAACAGTATGGTATTCTTTTGTATTAATTTTTTTAGCGCCTAATATTGATGCTCCTGCAGTGCTACGACAATAATCAAATAATCCAGGAAATATCGGACAATCATCTTTTACATTGAATTTATTGATATCGTTTACCATTTCACTAGTAAGTTCTCCTGAAATGGATGATAAGAAATCAATGTAGTCAGATGAGTGAAAGTCTAATAAATCATTATATGATGCCTTTGTCGGCTTCAATATGTCCATATGATGAAATAGTTCGTAATTAATCATCAGCGAATATGTCATACTTACACGGAGTGGTTTCATTGGATGTGCACGAGCATAATTATAATTTTGAATTTCATCATCATGTAAATACAAAATACTTTTTTTCATACAAGGGTAATCATTAAATAACAATTAATAAAAATATACGAATTTATTAAAGTCAATTTTTGTAGTCAAAAAGCAAGGTTCCTGCAATTCCACCAAGTTTATTCAAATTTTCTCCTAATGGATTATCTATTGGAATTACAAAAATTTTTGCTCGAAATTCCTTAGCTTCATTAATAATTTTTTCAATAACTTTACGTTTTTCAATTGTTTCAGGTCTATATAATTTATCGGTAATAAATAATTTTTCTAATGCACCATAATTAAATACATCATCGAGTTCTTTAAATCCGATAAATGTCATCGAATCACCAGCATCAAAGCGATTAAAAAATTTTTGACATTCTTTTAGTTCTTCGATATATTGAATATTTGAAAATATTTTGATAAAACTAGGATCAATAATAAGTTTTAATAAAACTTTATTGTTTGGTAAGTCTTTATAGTCAGGTGTAAGGTGTAAATATGTTATTTTACAATTAAATTCTTTTTGTGATCGTGTATAATATTCATATCCTATTTTACAGTGACTGGCTATTATTGTCATTTTATAATCTTTAGATTGCGTATTAATACTATTAGTAATCGTTTTAAAATCTTTTTCTTTATTTCCATATGTATTAAGTTTTACACATTTTGATTTTCCTAATGATGAGATAACACAGTTTGTTTCATAAAATATGATGAATAATAACTCTTGTTTTTCATTAACAGCATTTTTAATAGAATTAATATCTTCTGTAGTCCAACACAATTTATCTACACTAAACTTTTGATGCAATGTAATATTTAATGTATGATACGATCCTAGTGAAACATGTTCATTTTCTTTACTTGTTTTTCCTTTTAAAGATAATATTCCATATTCTAAGTCTCCGGAAATTGATTCTACAATTATTTCAAGTCTACATACGATTTTGTGTTGTGATTTACCATCAAAGTTAACTTTTCTTGTAGTTGTTGTTTCAACTTTATCTCCAAGCTGTAAAATATTACTCAATAAATAAACATCATCAGTATTTGTTAGTGATAATACTAATTTTCCAGATTTCGATTTTTCATTAATGCTATTTTTAAAAATATTCATGAGAAAAATTCTAGGAAAAAAAATTATAAATGTTATTAAAAGAAATTTTATTTAATAACTATATAGGTTGACTATAGATAATGAAAAATTATATTATAATCAATATCATAAAGATAGATCCACCAATAATTAAAGAAAATGCAACACAGTCTGCTATGAATTTTAATTTACAGATTCTATGCTTCTTCCATTCACATATATTTGTACTTACTGATATTTGGTGATTTTGTATTGGTTTAGTTATTTGATTATCGATATGTTCATACAGTTTCATCTTCTCATTTTGAATAAAATAAAGAAATGCAACTTCCTTATCATAAATTTCAATAATGGAAGTAATATTAAACTGTATTTCAAACATCAAATTTATATTTGTCAAATTTGTTTTCATTTTGCCAATCATATTAAAAAAATGATCTTTTGAAACTTTATGATTTGTTAAATTCTCAAAATCTTCTTTTATAAATTCCACTTTATTAAATATAGAAAAAAATATAATTGTTTTGAGACGAAATTTATCAATTGGATTTTGAACTAAAAAACTTTTTAAATTGGTTGTGATGTCATGTGCTTGAATTTGAAGTTTGTCAATATCTTGGTATAGTTTTTCTTTTTGATAATGATTTTTATTCTTTAGATTTAAGCCTGTTTTTAATGCAAATTCAGCAAAACCATCAGTAAATGTACTTTTACATCCTTCAATATTTCCAATTATACAAACTTTGTCCAAAGCCTGTGCATATACAAGCATATATAGTCCAAAAAATACATCTATAGTAAAACATATGAACAGTATTAATCGTAATCCAAAAACAAAAATAGATTCATATATAACAGATACAATAAAATATAATGCTAGGAATGTATGACATAAAAAGTATATTGTTAAAAACACAATTGAAAAATTTGGTTTTGAATAGGTTCTATTGAGAGGTAATTTAATTTGAGTTTTAGTTTTAATACAATTAATATTGTGTTTATCATCCATTTTAAAGTTACTAGTGATTTTTTTTAAAACTTTTTTAATATTTAAAACAAATTCAACGTCTACAGCCATTTCTTTTGCAATAAAATCAAGATTTTCTATATCAACATTAAATCCTTTCATTTCATTTTTAATTTTGTTTGCTTCATAATTCCAATCTTTAAATGTAGTTGTAAATGAATCAAATTGTAATTTTAATTGGATGAATAGATCATCCATTTTTAATATAAGATCATTTAATTTTGCATTTGTTGTTAATTCATCATTGACATATTGTGTAATTTTCTTTATATAAAAATCATTCTCATCATTATTCATTTTAAGACATACAGACTGTGTTGAAATAGGATTTTTTGTTTCATATGATTTTGTATTATACTGTCCATTTTTACTTATAGTTTTTGATGATACCTTAGTATTTGGGTTAATATTTATTTCATTAACTAAAGTAGTATTATGTTTTTCTGTTTTGGAAAAATGCTGAATATTTGATGTTGAATTTTGCTGTTGAAGAGATAAGGATGGATGTTTATTAAATGCACTTTTCTTCAGAAATTGACTGTTGGATATTAATAGCTTAGCATATGATAAGTCAGAAAATCCATTTTCTACATATTTGTCAAATAATCCAACGAATGTTATAAGTTTATCAGTGGTATATTTAATTGCATTTAAATTTGTTTCTGTCGTGTTTCCAATTACTTCAAAACTATATTTGTAATTTAATGCTGTAAAAATTAAAAAGCTACCTGCAAGATAAAAATAGTAGCTATTAGCCCTATATGTTGTGTAAGATACAAAAATTACTAAATTAACTAGTATATTTAAAAGTATAATTATGATCAATCCTAAAAAAATATTATTGTTAAATACATACGCTAGTAATTGTAAAATCAGCGCATTATCAACATTTACTTTTAATGGATCTCCTAATATAAAATAACGTATATATTCCAAAATATTCATAGATCAAAAATGCATTTTTGTTTTAAATTTGAAACGGGATACAATATGAAATAATTGATAATTTATATTTGGAAAATATCTCACCCTTGAAATGGAAAATGCATATGACTATTTATTTAAAATAATTCTAATAGGAGATAGTGGAGTCGGAAAGACATGTTTGTTGCAAAGATATACTGATAATGTGTATAATGAATCCTTTCAATCAACAATAGGCGTTGATTTTAAAATTAAAACAATTAAATTAAATGGGAAAAAAGTAAAACTTCAAATATGGGATACTGCAGGCCAAGAACGATTTAAGTCCATTGTATCAAATTATTACCGTGGTGCCAATGGAGTATTTCTTGTATTTGATATGCTTAAAAAAGAGTCATTCAACAATATATCTCTGTGGCTTAAGGAATTTAGGAATAAAAACCCAGAATATGAAACCGAGATATTTTTACTTGGGAATAAGGTTGATGAGGTTAATAGAATTTGTATTTCTTCTGACGAGATTAAAATTTTTTGCCAGAATAATAACATCAATTTTAATAATTTTTTAGAAGTTAGTGCAAAAACCGATTTTAAAGTTGAAAATGCATTTTTACAATTAACAGAAAACATGATTAAAAAGTTTGGAAATAAAATGATTAGTAGAATGCATAAAAATAAACTTAATATTACGCAGACACAAGGTAGATGTTGCTAATCATTTATTTTAACCCTTCAGCTTAATATCTATTGCTATTTAAGCCCTTAAATATGCAACTAACTGAAATAGAAACAAAGATTATTAATTTATTAAAAGATTATTCACAAACTCTAATTCCTCCTGTAATTCCAAGAATTGCAGGAGGATGGGTAAGAGATAAACTTTTAGGAAAAAAAACAAATGATATGGATATTGCATTAGATAATATTACTGGAATACAGTTTGCAACAGGACTAATTAGCTATATAGGTATTGATATTCATGTTGGAAAGATTATGGCTAATCCAAAAAAATCAAAACACTTAGAAACAGCAGTTATACATTTATTTGGATTGACTATTGATTTTGTTCATTTGAGAACTGAATCATATACAACTTCACGAATTCCTCAAATAACTCATGGGACCCCTCAAGAGGATGCTTATAGGCGGGATATTACAATCAATGCATTATTTTATAATTTGGTCACAAATGAAATCGAAGATTTTACAGGAAAAGGACTGTATGACTTACAGAATAAAATAATTGATACTCCACTCAATCCAAATGTAACACTACTCGATGATCCTTTGAGAATTCTCCGAATATTTAGGTTTAGTGTAAAATTTAATTTTAAAATTAATGATCGAATTTATAATGCAATCAAGCATGATAATATTCGAATCGCATTTAGTACTAAAATATCCTGTGAGCGTATATGGAATGAATTATTTACAATGCTTAACTATGAAAATGGTTATTTAGGATTGGCTAAGTTATCTGAAGGGGACTTTATCGAACCAATTTTAAAACATAAAAATTCAATAGAGAAAGTTATGTCATTCATAAATAAAATACAAAAATGGTTAATACCACCAAAAATAAATAATGAGCAAGTTCTATTACGCCTATATATTATATTGCATGAATTCTCAGGAATATATTGTGGAAAAGACTTTTTAGCCTGTATATTAATTGCTAAAGCGTTAAAACCTCCAAAATATTTTATTAGAAACATTAATGCAATAGAATTAAATCTATTATATTTAAAAACAATAAATTTGAATGATTTTGACTTGTTAAGAATTCAAATAGAATTAATTATCGAGTGTGGAAAACTATGGAAAGAATCATTAATTGTATTTTATGGAATGGGTAATACTATTAATATAGAAAATATTTTTCTTAATATATTTAATAATCATCTTGAAGAAAGATGTTTTAGAAAACAATTCAAAATTAATGGAAATGACATTAAGAATATTATACCAGAGAAGCATGTTTCTAAAATTTCTAATTATTTGAAAAAGTGTAAAGTATTAGAAATTGAAAACGAATGCCTTAGTAAAAAAGAAATATTAGATATTGTATTCAATAATTATATTTAATAAATACTTTTTTGTAAATAATTATGATTTGCATACTTATTGTTTTCCATAGCAAAATTTCGTCCAAATTAATATATTTGAAACAATAATACTCTATTAAAATGTATGAACATTGAAGAAAAAAGTATAATATAACAAGTATTTTTTGTGTATTCCATAACGATATTGCCATGACTTCTACTATATTATTTGAATTACTTTGCATTTCATATAGTTTTTTTAAAAATAATAAAGCGCGAATATTATTAGAGGTAATTACAACTCCTGGTGATTTAGATACATAAAATAAATGTTTTAATATTATGTTACAAGTTATTGGAAAAATAAATATAAAACATTTAAAAATTGAATAATCAAAATCTATCATTTCTTTTATCGAAAAACAAAATGATACAAGTATAAATAAAATTGAAATAGTAATATTTCTTTCTAATGCTTTCCTTTTTCCAATTAAGATTTCAGATAACATTAGTAATTGAATAACAGTTAAACTTTCAAAAAACTTGATAACGATAGGAACAAGCTCCTTATATGCAGTTTCAGAAGTAATAAAATATTCTAATAAACCTAAAACAATTCCGCTTAAAAATTCATTTAATGTGTATCTATTACATTTAATACAAAATAAAATGATAAAAAAAATCATACAGGTGAGTGGTGCATAAAAGTTTTCAATGTCAATTATTCCTAATCTTTTGTTAAGTGTATCACCAATGGCAATATATTTTATTATATCTAATAATGCATATGCAGATAGAAATAATGATTTGCATAAATATTTTTTGTTAAAAGTAATCATATAAAGGGAAAAACATCTTTTTATTTATCTAAATAAAAATTTCCATAAGCTTAATTTAAATTATAGTTATATTACTGAGTAATACATTATCTCCTTAATTAAATACTATAATTTAACATATTCATTTAATATGATTAAAATGATGCCAGATCCAAAAATTTTCTATTCCTTAAAATTTATAACAACATTGAAACACGACTTTCCCTCCCTTTTTATATTTAAAAATAAAAAATATTAAACCCCCTACATGGCAAGGACTAAACAATCAGCAAGAAAAACTCCTGGATTAACAGGTGGTAAGGCACCTAAGAAGCAACTTGCAACTAAAGCTGCAAGAAAGACAGCTGGTGTTCCAGGAACATCTATAGCAAACAAAACACACAAAAGACATGGTAAAATAGCATTAAGAGAAATTCGAAGATATCAAAAATCAACAGATAGACTTATTCGAAAATTGCCATTTCAAAGATTGTGTCGAAGTATTTGTCGGGAGCAAGCAAATGCAGCAGATATTCGATTTCAAGGACCAGCTTTATTAGCCTTACAGGAAGCAACAGAAATGCATATTGTAGGAATGTTTGAAGATGCCTTATTGTGTGCTCAACATGCAAAGCGAATCACTGTATTTGGAAAGGATTTTTTATTGGTTCAGAGAATTCGATCTAGATTCTTAAGAAATGCAGCGCCAACTGAATAAATATTATGTTCGTTATTATTTAATTTTTATTTGTCCCTTATATTATGGAAGTTTCAAATATTTATATTCTACCTTTTTATATTAAATCTTCTGTTTACGAGAATGGAATCACAAAAAAGTTAAATGCAAAGATTTCTAGCAGAAAAATTATTCTACGAGGTGAACCATTTGAAATTATTAAAATCAATGACTCTGTTGAATTTTATAGGAACGGAAAAAAGCTTCAAGGAAATTTTTTTTATTTGAAATCGCCTATCAAAAACAGTAACTTTTTCAAAGGAGTTGAGATGACAAAAAAAGGTATAGATATAATATTGAAATAATTATTTATTCAATGTATCAAGAAAAATTTCAATTTCATGTAATTCAGTGAATACATATTTACTTGCAGCGTATTCAGCATCTTCAGAAATTTCTTGCTCTGTTAGTATAAATTTGTTTTCAGTGATCATTAGAAGAAAAGGGCTTGTGATTTAATATTAAAATATTTTTAATATCTATTTATTTTTACCCTAACAATAAATATAAATGCTATTTTCTTTTATTAATGCATTAACTTTTACTGAAAGAGAACAATATATAGAAAATTTGAATATAATAACATATGCTACGCTTGCTAAATTAGCAGCTACGGATCATTATTCGAAAATTAAAATGAATTTTTATGATAAATGCCCTAAACCAACAGAGAACTGTGGATTAGAATGTCAAGTTCCAATTATAAAGTATAAAAATCAACATGGAATTATTGATTTATTAAATGTGCCTGAATCGTATTCTCCACAGATTAAAAATTCAGCAAATGTTTGGAGTGATATATATAATATTAATGATGATATAACATTTAAAAATATAGTTAGTGGATTACAGTTTTCGGTCACAACTCATATTTGTAGTTTCCATACATATTTTTTTAATATACCTATACCTAATCCATTGAAATTTAAAATTCGTTATAGAGAAGAATATAAAAATCAATTTTTTGATTTATGGTTACTCATTAGATCGGCAGTTGGTAATATAAAAAATATTCATCCTGATATCGATTCAGAAACCATGGCTCTTTCTGAATTAATTACAGATGATATTGTCCCAGTAAATTTAGAAATCATTTCTGATTTAAAGAAAACTATAGAATTTATTGCATGCTTAAATTGTCAAAAATGTATTCTCTGGGGAACTATACAAACAAAGGGGTTGATATCTGCTGTGAAAATACTTAATAATATTAAAGTAGAGAAAAATGAACTAATTTTCCTTATAAATTTGTTCAGAAGATTAAGTGAAACAATTAAACAATCACGCAGATTATATGATATAAAATATCCAGAATTATATTTAATATTAGTTTATTATAAAAGATTATTACCAACTGTAGCAATTATATTATTAACCATATATATACTTATTAGAAGAAGAATTGTAATAGTTGTAAAAAATTAAAAGTAGAATTAAAATTTCCAATTATGGCCACAATTTGTACATCTAACAAATGTTGTCATTGGTTCATCACAAGATCGCGTTTGTAATTGATAATATGTACATTGGTTTTTTTGACATCGACCACATTTAAACATAGTTGTTTCTGCTTCAGTGCATGCTTGACGTGAAGAATTGATAGAATCTTCAACAATTTTCTTTTCTTCTAATTTTTGTTTTTCAGATTTCATGTCCTCACTACTTAATTCTACAAATTCTTTTATAGTAATTTCACCATTATATATTTTATTACAGATATCCTTACTAATTTTAAGATGTTGTGCTTTTTCACGTACTATTTTGGGAAATGTTTTTTGATAATATTTATTTATGAACTGAGCTAAGTCTAGAGATAGCTGTTTTACTATTTTCAAATCAATATCAGAAATATTTTTTATAAAATTATTATAAAATATTTTTTCTGCTTTCTGAATCTTGGTATCAGATGTTGTTGTAGTAAAAACTGTCTTGTTTTCTACATATATTTTATCGTCTCCACATAATTTTTTATTCATGAAAGCAAAAAAGGGCTTAAAAATAGAGTTGATTAATTTATTTATTTTATGGTGTAATAGATATTGAATTAAATAAAAAGTGATCTTTCTTATTTTCAAATAATTTTTTAAATTCAATATTAATTTTTTTATTTAACTCAGCTTTCTTTGTTAAATTCTTGATATCGATGGTCAGGTCAATAGAATTAGTATCTATTTCGACTATATATCTTGTTGCTATAGTATGCTTAATATTAATTTTTTTAATAAATGTTAGAAATTTTTCAGCCTTCATTTTTTCTTGAATAGACATATATTCCTCAATCTTATATGGTAATCTATTTATTCCACCAACTAAAATATACTCTTCATCTATTATTTTCAATACAACTGCCTTTTTTCCAGCTAAACGGCCATGAGTTAAAACTACAACCATATTCTTTTTGAAAATCATTGGGGCAGAAATTGCAAAAAAATTTATTAGAAAAATAAAACCTACTAAAATTAATTGCATGCATTTACAATTTAATTATATATATGTTATTGTTAGCAACATTTATTTTTTTTTTTTTAATGAATAAGTTAATGCATTCGAAATGTTATGTAAAAAATGATTCCATATAGAATTTCTACAAAAAATGATTTCACTAAAAAAAGTATAATGAACTCGTTTAACTTTATATTTTTCATCAAATTGCATGATATGATATAATTTGCCAGGATGATATAGCTTAGGATATTTACTATTGTGTTTAATGTCTTCCCGTATTTTATCAATCTTTATAGACAAATCATTTGGGGTGTTAATCAAATAAACAGATGAACTGATTGAAATACATAGATACTTTAAATCTAGTATTGATCCATAAGACAGCCTGGCTAACATATCATTTTCAAAAATATAAATTGAAATATCTAAACATTTTTCATTAATTTTTTTTACAATATTCTCAGAAAATGTTGGTGGTGCTGCAAATGCAACTGTTTTCACTTTGAATTGATATTTTTCTATAATTCTAAGATGTATCAATGTTGAAAGTGCTCCTCCAAGTGAAAATCCTGTAAGAAAAAATGTATCAACATTATTTTCTTTCGCTATCGAAGCTATCCGAGGAAAAACACTATCTATAAACATATCTGCTAACTTTTTAATTCCTTGATGAGTGTATCCATTCTGGAATTCAATATAATAAGCATCTAAATCGCTAATAGTATCATATGCATTTACTGTTCCACGTAAACTTATTGATATTTGCTTGATATTTTTTTCTGTTTTATAATAAATTAAATATCCAATCATATCTACATCTCCAGGATAATACTCGATTATGTCAGTCTCATTAATTTCTAATCTTTCTAAAATAGCTCTATAAATCGAATTAAATACATCATGTTTCTCTCTAACAATTGGAAGATGTTTAACAAAAAAATGATTTCCAAATGAGGCAGCTGCTTCTTGCATATATGAATATATTTTTTGAGCATCTTCATATTCAAAATATTGATATTTATCTTCACACAATTCTTTTATGCATAAATTTCCCTGGCAGTTATATTTATCACATTTTCCATATAGAAAACTATAATAAAAATCAGCAGGTAATACACCAACAATTGTCCAAAAGTTACATAATAAACTTCCCTCTTTAATATTTGAAAATAGTCCAAAAAGATTAATAAAAGTTTCTGTTTTACTAGGAGTAAAGCATAAATTAAACACCCTTTTGACAAACGAATTTTCTGTCCATGATTCATCTATTGGTTTTTCATTTAATATACTTGTGCTATCATATATAGTAAATTTGAAAGTAATTTTTCCCACAATATTCCATTGTGCATTGTAATAATTTGCCTTTTCTATTTCCTTTACAGTTAATATGTCAAACGTTTGTAAAAACAAAGTTTTATTATCGTAGCAATCTTTAAAATAAATTTTACTAGCACCCAAGTTCATACGTTTACCTTTATTTATGGTATAAAATTGTATTTTTACATGACCAAATAAAACTTCATGCAAAAGGTGTATATCAATTTTAAACGATTGTAAAAATTCTTGTGAGCTGTTTTTGTATAAATATTCAGTATAAATATCAGACATATATGGAAGATCTATTTTCTGAATTTCCAATACAATCGTTTTTCTATAAAGTTTATCCATATTAAATTAAGGGGATTAATATTATTTTGTCTTTCCATGGGAATAATAATAAAATTCAATAAATTATAAAATATAAAGATGAAATAGAAAGTTTAAAATTTGAGCCCTTATGAATGTAATAAATGAAAAACTTCAGTTAATTATCGAACGACTTAACGATGATTTACCAGACATTAGAAAAAATGCACTAGAAATGTTATTTGATGAAGTTCGTTCGACTTTAGGGACAATTACTTCTGAACAACAGGATTTGTTTGATGTTATCAATATATTAAAAAAGTATTTATCTAATTTTAAGGATAGTGAACGACAGATGCTAAATGACATTATTTCTGTATTAAGCATGGGAAGTGATAATGAATCAATAAGAAATGTTATATTGTATAGATTAGATGGAAATATAACTAGTTTAGAATATTGGGGTCATCAGTATGTTAGGCAACTTATATTTGGTTGTTGTATGATTCGAAATAAAGAAATAGAAGCAATTAATTATCTTTCATTGATTGATCCAATTGTAGCATATTTATTTAAATATAATTGTGAAATAGAAGCAATTGATTTTCTTTTAGAAATTAGTGGATTACCTGAAGAAGAAATTTGTGGTAATAATATTCGACCTGAAAATGGAACAAATATAAATAAATTAGAATTAATTGCACAATATACAGACAATAATAATTTTTATAGAATTTATTTATATTTACTTGAGCTTAATAAATTTTTTGACCTTTACAAATTGATCCTAAAAATCACAAAACAATTTCCATCAATGTATTTAGTTCATCTAATTGAATTTGAATTATATCAAGAAGCTATTGATTATGTCAAAACCCAAACAGGTACTGTTCGTTTACAACTATTATATATTTTAGCTAAATGTAATATTTATTTCCAAACAGGTATTTCAGAAGAAGAAGCAATTCTTACTAACAGTCATATTTCCAATTATTTTGTGGAAACAGCAACAAATTTAGAAATTCTTCAGTCAAAAAAAATAGATTATATTTTAAAGGGATTGAATATTGACAAAGTAGACATAGCAGTTATTGCAAATGGATTAATCCATTACGGATTTATGAGAGATCCAATATTTAGACGATTTTCGGGAGATTATAATATCAAGTCTGAATATTTAAATCTTTTAAATCAATGCAATAACAAAATTGTAACTATTAATGCATCTATGGGATGTATTTATGGGTTTAACCATGTTGAATTAGCTAAACTATTCACCAATGATTTGTTTGAGGTAAAAGATTTGGGAACAATTTTAGGATATGCTATTGCAGGTGCCAAAAGTCATGATAGAGATGGTAGCATGTTTCAGCTGTTATCTGGATTTTTAAATTCAACTAATACGAAAGATATACTAATTGCCCTTACTGGAATATCAATTGTATATTCTGGATCTATAGGATGCGAATTTAGTATAATTTATGAAAAAATATTTCCTCTCTTAAACCATAGTAATACTGATGTTATTTGTATGAGCATATATACATTGTGTTCTGTATTTTATGGAACAGCTAGAAATGATTTAGTTTCTATATGTACAGAAATCTTTGTAGAACAAGATTTAAGTACATCTGAATTTTATACATTTGCAATATTAGGCATTGGATTATTATTTTATAAAAGAGTAGATTTATTCGATTCTGAAGAGTATACAGGATTACCAAAACCAATTCAAATATTAGCATTAGGATTAATGCATCTTGGAAGTGGATCTCCAGATATAGTAGAAGAAATTATGAATAATTGTTTTGCAGGTGAAACCGAGCCTTTGAATGAATCTCTTGGATTAATTTCTATTGTTTTAGTTGGCATTGGCGATTCATTAGCATCACAAATGATTGAAAATCAATTAATGAGTTCATTATTACTTAATAATATACATATAAAAAATGTTGTTCCTAGTTGTTTAGCATTATTATATGCATCTTCAATGAAGTCTGATATAATCGATTTTTTAGAAAGAAGTATGAGTACAAGAGAAACAAATGTTACATCCATTGTTGCATTAGGAATATTAGCTGCTGGGACTAATAATTCACGAGCAATGAGTATTCTTAGCTCTAATTTCAACGCTATGTATAAAGACATAAAAGCTTCTAATGCATTAATCTTTTCACAAGGATTAATCAACTTAGGAAAGGGAATGCTTACTTTATCTCCATTAGTGTATGATAAACGAATTATTATTGACAAATCTATTATAGGACTTCTTGGATTAATTACAATGTTACTTAATGAAAATTATAGTTGTTTTAAGGAAAATTCATTTATTCTGTACATTATAACTCAGGCTATTTTGCCAAAATATGTAACAGGAATTACTGGTGAGATTAAAGTTGGAAAACCAGTTGAGACAGTTGGACTGACTGGAAATCCAAATCGAATTAGTGGAAAAATAGTTTACACGTCCCCAATTATATTAAATTATGATCAGAAAGCAGAAACGGATGTAGAAGTTGAAACAGTGTTTATTGAGGATATTTTAATAAAAAAATGATTAGTAATTATTTTTAATTTTTAAATAAAAATTTATTGTATTTATGAATTATAATTCAATTAATAAAAAAATTTTGAAATTATTACAAAATAATTATTCTATGTATTTACAACTTCTTAAAGAAAAAAAATATGAAAAATTACTCAATAAATTATAGGATGCAAAATATAATTATTTACTTAAAAATATACCATTTATGAATTATAATAACTGGGATTATATCCTTCCATATGACAATAGTGTAGCCGAGAAATATCAATTAAACTTTATTAATGCTAGTTTTGTTGATAGATTTATATGTTGTCAGGAACCTAAAAAGCAATATGTTAATCATTTTTATGATTTTCTAAATAAATCTGATATCAAGCTAATCATTTCATTAAAAAATAATTTAACATTTTTCAATGATAAAACAGCTTCTAAAGTTACATTGGATGATTATTTTTTCACAGTAAAGGAATATGACATCAACGGAAAAGTATATACCCAAATAAACTGTCATTCTTGGCCGGATAGGGGAATTTTACCTGCAGAGCAATTAGAGGAGTTACACTTGTATATTAAAACAAATTATCCTACTGAATTTTACGGGCAATTTAATATTTTAGTGCATTGTTGGGCGGTGTTGGAAGAACAGGTACTTTCATTATGTATTCTCTTTTAAAAGAAATGAATAGGAAAATTACACCCGAGATATTTTTGGATACATTACTATGGTTACGATCGCAAAGACACTTACTAGTCGAATCACCCGTTCAATTAAAATTTTTAGCTGAACAGTTTATTAAATAGATATTAGGTAATATTGGTGAGATAGTGTTATGGTGGACAGAATGAACAAAAATTTATTACATTATGAAGTTTGGCATCACAATTATAACAAAATGGAGATAAACATTTGTTACAAACACTAATAACTTCTAATTTTTCACATCCATAACATTTATTTTTTTCAAGACTAGAAAGTTCTTTAGATGTAACAATTTTGACAAATGATTCAAGATAATAGTTGTAACAAATTAAATCACGTATATCTGTTGACGAAATCAATTCAAGTTTGCAAATAGGACATGTGAATGGAATTGTACACACAAAGCCTTTGCATTTTGGACATTCAAACAAAACTGAACACAAATTTAAATGACACGCACATAAATGTTGTGTATAGATGTTTTTCGGAAACTCTACTTCAATCAGAGAACATTTTAAATCTAATGATTCTAGAAGATATAAAAAATCACTCAATATAGACTTAAATTGAAAACTATTTATAAGCACGACATATTTTCCTCCGGTAGAAATACAGACCTTTTTGAGAAGTGTAACTTCACCACATATTGAAATAATATTAATTTTTATTTTTTTTTGTATTATTTTTTTCATTACCTTATTAAAATTTATTTCCCCATTGATCACTCCAACACTAGAGATAATTAATAAACATTTTTTCAATGTGATTTTTGTATCTATAATTCCATTTATACATGCTAAACATTATATAAATGAAAATGATTTATTCATACAACGAAAATAAATCTTTGTTAAATTTAAAAATAATATTTAACATTCTACAAGTATAGAATTATATAAACAAATTTATTAGTACATAAAAATCATTAATTTCATAATTACTTCTTAGTTATATACTAATTAATATTATTAATTAATCTCATTAACTGTTTGAAATGTATCTAATAATTTATTGTCTATGTGGTTATCACTTAATAATATTATATACAATCGTTTTATTTTGTTTATGTCATCATTAATTCCCTTTATTAATTGTTTGAATCCATTATTAATTTTATCTACTAATTCAACACCATTTGTTAATTGTTGAAATTTTTTATCTATTGTATTTACCAAATTGGCATTGTTTATAAGTTGCATAATTCCTTCATTTACTTTATTTACATCTTCATTAATTTCATTAGTTAATTGATTAAACCTTTCATTTAATTTATTTATCAATTCAACAATAATTAAATTATCTGGATTATTTTCTGGTTTTAATGATTCAACATCTATATTTTGATTGTATTTTGTATTAATATCAATATTTAAATTGCTATTGGTTTGTGTCAAATTACTTTTATCTATTGTTTGTTCAAATTTGTCATTGTCTATCAATTCAACATTAATTGTATTATTTGGATTTGGAATATTATTTTTTGTACAATAGTAAATAGAAAACAAAGAGAACAAAGTACAGTACATGTTTCAAAACAGGGTCAACACTAATTTTTTTATAAATAAATAAAATGATAATAATGATTATTTCTAATATTAATAAAAACAAGATTAAATCACATTGTTAAATTAAATATAAATTTTCAATCATAATTAATTTTGATGTTTTAATAATATATAAACAAATATTTTTATACATCTAATTATTAATTTGAATTCATGTTGAACAACACATAAATGCAATAAATGACAAAATAAAAATCACTAATAAGATTACAATTAATATTATCATAATTCTTACTAAACTGTCAATAGGATTGATTTCTTCATTTGAGTTTTTAATATTTTTAATTAATTCCTTTTCATATTTTTTTGAAGTAATGGAATTATTTTTATTTTCTACTACTGGTTTTGGATTACTTGTATAAATATTGTGTGGTTTTTTATACGATGTGTTTTCAATTTTTATTGATGATTCACCGGTGTTTAAAGATGAATTCATCTTTTTAAAAAGCTGTTTTGGATTTACTTCGGCATTTAACCTTTCTTTCTCAGTTAATTTATCTATAATATTATCATTTTTCCAAGAGTTAATAAATGATTTAGCAGAAAATGTAAAATTAAGAAAAAGTACGAAATATATTGCAATTATCATCATTTATGAGGTATTAGTTTACTATTTTTACATAAATAAATAAACAAAAATCGAGTTTAAAATTAAAAGGTTAATGTAATGCACTACTATAACTTTAACCCTTTATATGAAATTAAAATTTTTAAATGGGGATCAACAATGGAAATGGTGTTGCTTTATAATATTATACATATTTGTCTTTGCTATAATTTTTCTTTATCAATGTGTGTACATTAAAAAATCTGAAATTCAAGAATTACAAAAAAAATTAGATAAAGAATTACAAGAATTAAAAAAGATTTTTGAACAGCAACAACAAAAATCTAATGAATATAAAATAAAAGCTACTAAAGCTTTAAATGAATTATTTGATCAAGAATATACAAATCAAAATAAAAAAGACAATCAAATGTACTAAAAGTTTAAATTTTTAATTATAAACGAAAAGTAAATAATATGTAAAATTGTCGTTTCCACTTATGCAATGGTATTAAATTGATTATATTTTAATTCCATTAATTTTTTATAAATTTCACAAATTTTTGTACAAAGGGAAATAGTATGTTTTTTTCCGTAAACAATTGATTCCATTTCCTCTTTTTTAGTTACTGTAGTTTTAAAATTGTTTATAATATGAGAATAATCTTCAAGAATAGCATCCAAAAGTTCATTTATTTCTTTTTTAATATTATCATAGTACATTAGGGCTAAAATTATTCATTATTACTGTAACATTTCATTATAATACGTCAAAACATCAGGTACCTTTTTAAATTGATCATCATATAGGTGTCTTCTAAATATTTCACTATATACACTATGTTCTCTATCATGAACTAGTATATATCGTGGCTTTACTTTTTTGTTAGTAGTAAAGTAAATTATTAATTCATTTACAAACTTTCCAAATTTATTATCTTTAATATTTTCAAACAATGTTATTCCACTAAGGGAAATGTTATTATCAATATCGAAAATTTCATGGTATGTTTCATTTGTTTGTCCTATAAAAACAAATAAATTAATTCCACAATCCATTATATATATTTGATCATCAATTATTGAATTTAGACTAAGGTTGAGACATTCTACAGTATTATTAATATTAATAAGCACAGGATATGATATACGATCTAATATCTCTATGGTTGAATTCATGAATAAATACACATAAAATGCTCGAAAGTCAATTGCTATTGTATTATCCATAAGTAAATTTTTCTTAAATGCTGAAAAATAACTTGCCAGGATATTCCGGTTATGAATGATTATTGAATCTTCTGATTTAAACATAGATTTAAGGGTAGTGTCCAAATAGATAGATCCAGCACCTTCTTTGTCAATTTCTTTTTCAATTGCATTATGAAACAATCCGAGTGCTAATTCTTGTATGCCTGTTTCATTTATATTTTTACTATTGAGTGTACATACTCTTATTGTTTTAAGACCATTTTTTCTAGTACGAATCATTGCCAGTTGAACTATATATTGATTTTCAGATGATAATTCAAGTGATATAGAATGTGCAGAAACATAGTTTGGAAAAGCAAAGAGATCGTTTCCTCTTGATATACAATTTCCACGAACTTTTCTAATAGTTACACCTTCAGATACTCGTACACGCATTAATGCATTATAATAAATTTCTCCATTTATTGCATTATTCAAATCAGTAAATAATTTTTCAGTGTAAATAGGATCATCTCCATCATAGTTAGTATAATGAAATACTTGTCCTCCACCTTGGGTAAGAATTCGCATTTGACCATATTCTAATGTTGTTTTAGTACACATAAATAGATTTAACGCAATGGATTTAGTTTGTAATGATTCTAGAGTTTGTTTGTATTCATTATTAATAGCAAAACGGTTATTTGGTTTTAATGAACTAATTCCAGAATTTGGCATAGATGAAATAAATGAATAAATGCTTCCTGATCTAAAGCACATAGAAATTAATTTTATTGCAGCTAAAATGTTATTGTTATTATTTGCCTCCATTTTGTTAAAATATTCTATAACCTTTTCTATCAATTCATCTAATATTTCAAAATCCTCTGTAAATAATCCAAAACTAACCTCATGATTTAATAAAAGAGGCATTGCACCTGTTATTACACTGATAGTACCAGTCTTATTTAATATATATACATGTTCAGAAAAAAAGAACAATGCAACTCGTGTTCTGCAGTCATATGCATTTTCCAAAATAGCTTCTTTTAAGCATTCCTTAATTGCATGTATTACACTATCTAATAATTTGTTTTTTCTTCCTTCTAGTGATATATCAATAAGAAAACATATTATAGGAGGATCAGTTGTTTTAACATTAAAGTTTTCAGGAGCCTCAAGTTCATATAATTCTGATATTAGTGGAATATTTTCAAAAGTTTGTCTGTTATAGATTTCAATCTGTTCAGTTTGTCTGTATGAATTATTTTTATATATAAATGGTTGTTCAACTTGATTGATCATATTACACAAAGAACATTTCCACTGCATTCCAGGATTTATTACTTCACAGTAGCAATTCAAGTAGCTTTGACAAGATAAGCATCGTATTAATGGCATAGTATTAATATTAATATCCATAGCATCAAATTCAACAGTTATAAAGGTTAGGTTGTTATATTTACAACTTACCCGATACCATTCATAAAAATTTTCTGGTTCATTTGGCACATGCTCCATATGATTTGGATTCTCCTGTTGATCCATATTTGTGGGTATAAAATTAAATATATTTTTATTTTTGTTTTTGTAAACTTTTTAATTTTAAATAACTTTGGCATTAATATTAATTTTGTAGTAGGATTATTTTAGTATTTCGCATGCCCTTAAAATATTCTTATGGTACAGAATTATACATTGTTTGTTCAGTTTGCAAATATACTACAAGAAATTGAAAACACAAATAAAAGATTGAAGATTCAGGAAATTCTTAGCAATTATTATTCACATATAATAAATACTGATATTGGATTGTTGCATATGATACTTCACATGTCAACAGCAACTGTATATCCTTCTTTTGTAAATCTTGAAATGAATATAGGGGAGGGGGCGTTACAGCCAATAATAGCTGAATGTACAGGGATGGATATTAAGAAAATCAAGCTATTATTTAAAGATATTGGAGATTATGGAGAAATCATTAAGACATATAAAATTCAAAAAACATTTAACTTTCTTTCGGCAGCTAATATTACTCAGCTTACAATTCATGATGTTTATAATCTTTGCACAAAATTATGTGTCATTACAGGGACAAAATCAAACGTAGAAAAACGAAAAATTATTTTAGAAGTTATTAGAGATGCTTCTCCTCTTGAAAGTAAATATATAATTCGTTTGTTAGAAGGTAAATTAAAAATTGGATTAGCTCTACAAACAATATTAATTAGTCTTTCAATGGCTCTTGGATATTCTGACTACAATATAATTAAAGAAGCATACAATAAACGGCCTGACTTCAAATATTTAGTTGAAAAAATTATTGTTGCAAAAAGCATTGGAAAGACATTAAAGAATTTAATTATTAAAATTGAGCCGGGAATTCCATTGAAGCCAATGTTGGCAACACCGATCACTAATTTAGATAGTGCTATATCCAAATTTAATAATGTTCTTGCTGAGTATAAATATGATGGAGAACGAGCGCAAATTCATTCATATTTATTTAAAAGACAACTGTCATTTAAAAATTTGACTGGATCTACCACAACTATTGAACGATTTTTTGAACAAAAAACCAATGATGGAATAGTAAATGTAAATCAAAATACATCTGTAACTGGCAAGGTTTGTTACAATAAAATTTTTAGTAGAAATAATGAAGATATTTCTGAAAAATACAACGATTTAGTACAAATTAAGTTTAATAAAAATAACAAGAGCTATATTTTAGATGGTGAGATTGTTGCTTATGAAAATGGAAATATAATGCCTTTTCAAACTCTTAGTACAAGAAAGAGAAAATATAATGAAAAAATAGAAGTTCCTGTGTGCTATTTTGCATTTGATTTAATTTATTTTGATGGGAAGGAATTAATTGACATGCCTCTTGAAGATCGTCGAAAATTATTGCATGCTAATTTTACTACTATTCCAAATGTATTTGAATTTGCTATTGGAAAAATTATAGATAATAATAAGGATATGAATGAGTTTTTTGAGGCTGCATCAACCAATCGGTGCGAAGGATTAATGATAAAGGATTTAGGCGCATTATATCAACCATCACATCGTTCAAACCAATGGCTAAAATTAAAAAAAGATTATTTAGATAATTTAGGTGATTCATTTGATTTAGTTGTTATTGGTGCTTATTTTGGAAAAGGAAAACGAACAGGCATGTACGGTGGTTTTTTACTTGGAGTATATAATGATGAAACAAATACATTTGAAGCGTGCTGTAAAATCGGTACTGGATTCAGTGATGATTATCTTATGAAAATTTATAAAGAATTGGAGCCTATACAACAGCATACTAATATATTAGTAAAAGATAGGCCAGACATTTTTGTTATTCCCCGATTTGTTTGGGAAATTAAAGCAGCTTCTTTAACATTATCTCCTATATATATAGCTGGAAGTACAGATACGATATCAAATAAAGGTATTTCACTACGATTTCCACGATTTATTCGTGAACGACTAGATAAATCACCAGAAAATGCTACAACATCAAAAGAACTATTTACTATGTTTATGGAACATACAAAAAACTGCAAATAAATTGCATATTAATGGATAAATAATTCTCAAATTTATTAAAAATATGTTATAATTAAAATTAATATACATATTTTTAATTAAAATCCTTTTATATGATTAGTACAATTCAAAAAAATATTAATGAATTTTTACATCAAAGTAAAAAACCACTTTTAATTTTAATTCAGGGATGTACATGTTCTGGAAAAACAACATTTGCTAATTATTTACATGCACATTTAGAAAAAGTTATAACAACAACTATGATTTCACTAGATAATTATTATAAACAAGTCAATCATGATTATTTCAACTCTGAAATTACAAGCTATGATTTTGATAATCCAAAAGCATTTGAATGGAATAATCTAAGTAAAACATTGAAAGGATATATAAATAAAGATGATATTATATTTAAATATAATTATGATTTCAACAAATCACTTTCTACAGAATATAAAGTAAAAAACACTTATCCTAAAATAATTATACTTGAAGGACTATATTCATTTAATTTGTTTAGTGATAATAAGTTTGATTTAACTAAATTAAATCCATATAAATCAGCATTAGAATATGATGATGTTCCATTAGAATTAAATGATACATCTTTGAGGAATCAATTTATTACATTAAAAATTATGTTAACAATGAATAAAGATGATATAAAAAACATTTTATTATATAGAAATGCAAATTATAGAAATTCCGATGATGTTACTCATAACTTTAATACGATTTTAATAAACAAATTTAACCATTATATCTGGCCAGCTACAGAAAAATGGGGTTTACTAGGTAAAAAAGAAGCTGATGTTATAATTGTTGGTGGAACACGTAATAAAAAAGATATAAAAATAGTTACTAATGCAATATTGACGCCATTTAATTACAAAGATCAATTAATTACTTTTGAAAAATTTATTGACCATTAATATTAATAAATATTAACTTAATATGATTATTATTTTACTTCCTACCCTTAAAATGAACTATAATATAATTAAAAATATTATATCTCAAAAATTGATTAAAAATAATAAAATTGATATTATCTTAATTCAAGGTGCATCCTGCGCTGGAAAATCAGTGTTTTGTAATTATTTGTATAAACACTTGAAACAAGTTAAACGGATCATTAAAATTAGTACAGATAATTATTATCGTAGTCCAGGATTTGATTATAAAGATCCTAAAGTAAAGTTATATAATTTTGATAATCCTTGTGCTATTGATTGGGATAGTCTTTCAAGAACATTAGAAGGATATGCTAATAGAGAACCACAAATTATTGGAAGTACATATGATTTTTTTACCAAAATACGTAAAGATATTATTATTCCTAATATTTATCCAGATATAATATTAATAGATGGAATTTATGCCTTTAATTTATTTAACAAAAATATGTTTAATATTACGCGTTTAGATCCATATAAAGATATTGAAAGTCATGATACAAATGTATATACGGATAATGACCATAATTTTCTTACAAAATTTAATGTTTTAAAAATTCAACTTAAAATTTCTAAAGAATATGCAAAAAAGATTCGCATCGAAAGAGATATTAAAAAAAGATTTAAAAATTGTTCTCCTGAATTTATTGATATGCTGGAGTATAAATTGAAAAAACAATGGCCAGTAC

>Scaffold_1888
[truncated: 115,817 more chars]
