# Supplementary material for: Patterns of Genome Evolution among the Microsporidian Parasites Encephalitozoon cuniculi, Antonospora locustae and Enterocytozoon bieneusi
Source: PLoS One. 2007 Dec 5;2(12):e1277. doi: 10.1371/journal.pone.0001277 (PMC2099475; doi:10.1371/journal.pone.0001277)
Supplement: Table S1 — Description of the ORFs, their position along the scaffolds, and the accession number of their best match against the E. cuniculi genome. (0.37 MB DOC) [file pone.0001277.s003.doc]

**Table S1**: List of putative Open Reading Frames identified along the three *E. bieneusi* genomic fragments analysed in this study.

| ***E. bieneusi* ORFs best match against *E. cuniculi* genome and ORFs description** | | **Accession number** | **1st nt position** | **Last nt position** | **Sense-Antisense** |
| --- | --- | --- | --- | --- | --- |
| **Scaffold SC_2384** |  |  |  |  |  |
| **ECU03_0170** | **Hypothetical Protein** | **NM_001040892** | **98** | **1540** | **AS** |
| **ECU03_0160** | **Hypothetical Protein** | **NM_001040891** | **1561** | **2451** | **AS** |
| **ECU03_0250** | **similarity to glucose/Na cotransporter** | **NM_001040900** | **2501** | **3364** | **S** |
| **ECU09_0990** | **Hypothetical Protein** | **XM_950560** | **3374** | **4096** | **AS** |
| **ECU03_0290** | **DNA-Directed RNA polymerase II** | **NM_001040904** | **4224** | **8849** | **S** |
| **ECU03_0300** | **Hypothetical Protein** | **NM_001040905** | **10282** | **10659** | **AS** |
| **ECU03_0310** | **40S Ribosomal Protein S16** | **NM_001040907** | **10933** | **11364** | **AS** |
| **ECU03_0320** | **60S Ribosomal Protein L13** | **NM_001040908** | **11438** | **11923** | **S** |
| **ECU10_0590** | **Phosphatydil Inositol-3-kinase** | **NM_001042007** | **12949** | **13791** | **AS** |
| **ECU03_0420** | **similarity to yeast CDC68** | **NM_001040917** | **13918** | **16419** | **S** |
| **ECU03_0430** | **WD repeats-containing protein** | **NM_001040918** | **16473** | **17669** | **AS** |
| **ECU10_0970** | **Hypothetical Protein** | **NM_001042045** | **17700** | **18845** | **AS** |
| **ECU03_0440** | **DNA-Directed RNA polymerase I** | **NM_001040919** | **19029** | **22229** | **AS** |
| **ECU09_1100** | **Hypothetical Protein** | **XM_950571** | **22292** | **22882** | **S** |
| **ECU08_1630** | **Hypothetical Protein** | **NM_001041900** | **23000** | **23716** | **AS** |
| **ECU09_1170** | **Hypothetical Protein** | **XM_950575** | **24199** | **25140** | **AS** |
| **ECU07_0090** | **DNA-Directed RNA polymerase III** | **NM_001041559** | **25196** | **28720** | **AS** |
| **ECU03_0240** | **ABC Transporter** | **NM_001040899** | **28785** | **29483** | **AS** |
| **ECU10_0690** | **ATP-Dependent RNA Helicase** | **NM_001042017** | **30726** | **32057** | **AS** |
| **ECU06_0940** | **similarity to 3-Ketoacyl CoA Thiolase** | **NM_001041472** | **32637** | **33494** | **S** |
| **ECU06_0190** | **Vacuolar ATP Synthase 16kDa** | **NM_001041397** | **33717** | **34193** | **S** |
| **ECU04_0740** | **60S Ribosomal Protein L22** | **NM_001041107** | **34244** | **34582** | **S** |
| **ECU09_1680** | **asparagine--tRNA ligase** | **XM_950628** | **34624** | **36000** | **AS** |
| **ECU03_0520** | **HSP 70kDa PROTEIN** | **NM_001040927** | **36093** | **38237** | **AS** |
| **ECU03_0530** | **Hypothetical Protein** | **NM_001040928** | **38422** | **38967** | **S** |
| **ECU01_1095** | **Hypothetical Protein** | **XM_960853** | **39019** | **39315** | **AS** |
| **ECU03_0590** | **Integral Membrane Protein YIPA** | **NM_001040934** | **41757** | **42350** | **AS** |
| **ECU05_0260** | **Phosphomannomutase** | **NM_001041231** | **42394** | **43050** | **AS** |
| **ECU01_1390** | **Chitin synthase I** | **XM_960884** | **43767** | **46271** | **S** |
| **ECU07_0410** | **Ubiquitin Carboxil Terminal Hydrolase** | **NM_001041591** | **46345** | **48948** | **AS** |
| **ECU02_0930** | **Suppressor of Forked Protein** | **NM_001040807** | **49225** | **50631** | **S** |
| **ECU03_0310** | **40SRibosomal Protein S16** | **NM_001040907** | **50687** | **51079** | **S** |
| **ECU02_0900** | **60S Ribosomal protein L35A** | **NM_001040804** | **51087** | **51416** | **S** |
| **ECU10_0450** | **Hypothetical Protein** | **NM_001041993** | **51433** | **51825** | **AS** |
| **ECU10_1360** | **Hypothetical Protein** | **NM_001042084** | **52629** | **53216** | **AS** |
| **ECU07_0590** | **Hypothetical ProteinYMJ5-CAEEL** | **NM_001041609** | **54254** | **54370** | **S** |
| **ECU09_1440** | **phospholipid-transporting ATPase** | **XM_950604** | **55644** | **56609** | **S** |
| **ECU08_0280** | **Proteasome Beta-Type Component C7-1** | **NM_001041766** | **56621** | **57202** | **AS** |
| **ECU07_0960** | **DNA-Directed RNA Polymerase I** | **NM_001041646** | **57953** | **58561** | **S** |
| **ECU03_1260** | **Endoplasmic reticulum membrane protein** | **NM_001040998** | **58629** | **59276** | **S** |
| **ECU07_1010** | **Structure-Specific Recognition Protein** | **NM_001041652** | **59559** | **59852** | **S** |
| **ECU10_0640** | **Dynein Heavy Chain** | **NM_001042012** | **59889** | **60479** | **S** |
| **ECU01_0750** | **Hypothetical Protein** | **XM_960817** | **60810** | **61787** | **S** |
| **ECU02_0880** | **Hypothetical Protein** | **NM_001040802** | **62104** | **62616** | **AS** |
| **ECU09_1640** | **ATP-dependent RNA helicase** | **XM_950624** | **62704** | **63816** | **AS** |
| **ECU02_0850** | **Protein Disulfide Isomerase** | **NM_001040799** | **65140** | **66630** | **AS** |
| **ECU04_1290** | **ATP-Dependent RNA Helicase (SKI2 FAMILY)** | **NM_001041163** | **66693** | **67562** | **AS** |
| **ECU01_0400** | **Hypothetical Protein** | **XM_960781** | **67803** | **68099** | **AS** |
| **ECU02_0700** | **Hypothetical Protein** | **NM_001040785** | **68211** | **68654** | **AS** |
| **ECU02_0840** | **Hypothetical Protein** | **NM_001040798** | **68830** | **69933** | **S** |
| **ECU11_1460** | **Translation Elongation Factor 2** | **NM_001042285** | **70172** | **72733** | **AS** |
| **ECU11_1450** | **Protein Transport Protein SEC13 Homolog** | **NM_001042284** | **72888** | **73712** | **S** |
| **ECU11_1430** | **Diphtin Synthase** | **NM_001042282** | **77644** | **78402** | **S** |
| **ECU08_0290** | **DNA Replication Licensing factor MCM3** | **NM_001041767** | **78470** | **80515** | **S** |
| **ECU05_1410** | **Hypothetical Protein** | **NM_001041350** | **80542** | **81102** | **S** |
| **ECU11_1400** | **DNA-Directed RNA Polymerase II** | **NM_001042279** | **81143** | **81469** | **AS** |
| **ECU11_1380i** | **60S Ribosomal Protein L15** | **NM_001042277** | **81579** | **82193** | **S** |
| **ECU11_1390** | **Hypothetical Protein** | **NM_001042278** | **82218** | **82763** | **AS** |
| **ECU11_1320** | **similarity to Plasmodium Membrane Protein** | **NM_001042271** | **82789** | **83223** | **S** |
| **ECU11_1330** | **Transcription Factor** | **NM_001042272** | **83296** | **84009** | **AS** |
| **ECU02_1150** | **DNA Replication Licensing Factor MCM4** | **NM_001040829** | **84219** | **86372** | **AS** |
| **ECU11_1080** | **Manganese Superoxyde Dismutase** | **NM_001042247** | **86450** | **87232** | **AS** |
| **ECU11_1350** | **Translation initiation factor 4E** | **NM_001042274** | **87269** | **87808** | **AS** |
| **ECU10_1460** | **Glutaminyl tRNA Synthetase** | **NM_001042094** | **87891** | **89966** | **AS** |
| **ECU10_0390** | **Hypothetical Protein** | **NM_001041987** | **90168** | **91763** | **S** |
| **ECU10_1440** | **Hypothetical Iintegral Membrane Protein YSX3 CAEEL** | **NM_001042092** | **91792** | **92307** | **S** |
| **ECU11_1030** | **Proteasome Regulatory Subunit YTA6** | **NM_001042242** | **92360** | **93619** | **S** |
| **ECU10_0250** | **DNA-Directed RNA Polymerase II** | **NM_001041973** | **93634** | **97050** | **AS** |
| **ECU11_0980** | **Synaptobrevin-Related Protein** | **NM_001042237** | **97655** | **98332** | **AS** |
| **ECU10_0920** | **Ribonucleoside Diphosphate Reductase** | **NM_001042040** | **98419** | **98760** | **AS** |
| **ECU10_1370** | **Inositol Polyphosphate-5-Phosphatase** | **NM_001042085** | **98884** | **99987** | **S** |
| **ECU11_0910** | **Hypothetical Protein** | **NM_001042230** | **100903** | **101589** | **S** |
| **ECU08_1140** | **Hypothetical Protein** | **NM_001041853** | **101854** | **103182** | **S** |
| **ECU11_0680** | **Hypothetical Protein** | **NM_001042207** | **103188** | **104180** | **AS** |
| **ECU02_1490** | **Alanyl tRNA Synthetase** | **NM_001040863** | **104235** | **106817** | **AS** |
| **ECU08_2070** | **Hypothetical Protein** | **NM_001041942** | **107163** | **109037** | **AS** |
| **ECU08_1100** | **Coatomer Complex Beta Subunit** | **NM_001041849** | **109097** | **109564** | **S** |
| **ECU02_1210** | **Glutamyl tRNA Synthetase** | **NM_001040835** | **109571** | **111466** | **AS** |
| **ECU11_0520** | **Hypothetical Protein** | **NM_001042191** | **111486** | **112151** | **AS** |
| **ECU04_0250** | **ATP-Dependent RNA Helicase (DEAD box family)** | **NM_001041058** | **112262** | **112417** | **S** |
| **ECU09_1750** | **Hypothetical Protein** | **XM_950635** | **114357** | **115289** | **AS** |
| **ECU11_0660** | **SER/THR Protein Phosphatase PPI-1** | **NM_001042205** | **115401** | **116348** | **AS** |
| **ECU11_0670** | **similarity to Protein YG22_yeast** | **NM_001042206** | **116431** | **117885** | **AS** |
| **ECU04_0890** | **Pseudouridylate Syntase 2** | **NM_001041122** | **118011** | **118898** | **S** |
| **ECU04_0410** | **Hypothetical Protein** | **NM_001041074** | **118942** | **119532** | **S** |
| **ECU03_1590** | **Ankyrin Repeat-Containing Protein** | **NM_001041031** | **119540** | **120856** | **AS** |
| **ECU11_0820** | **DNA Repair Protein RAD51 Homolog** | **NM_001042221** | **121744** | **122757** | **AS** |
| **ECU11_0830** | **Transcription initiation factor TFIID** | **NM_001042222** | **122832** | **123194** | **AS** |
| **ECU11_0840** | **Proteasome Regulatory Subnit YTA6** | **NM_001042223** | **124412** | **125560** | **AS** |
| **Scaffold SC_2496** |  |  |  |  |  |
| **ECU09_1890** | **Transcriptional activator** | **XM_950650** | **1** | **2472** | **AS** |
| **ECU09_1880** | **Hypothetical Protein** | **XM_950649** | **2575** | **3786** | **S** |
| **ECU09_1870** | **Undecaprenyl Pyrophosphatase Synthetase** | **XM_950648** | **3795** | **4643** | **AS** |
| **ECU06_1310** | **Hypothetical Protein** | **NM_001041509** | **4663** | **5262** | **AS** |
| **ECU10_1090** | **DNA-binding domain** | **NM_001042057** | **5449** | **5988** | **AS** |
| **ECU09_1840** | **26S proteasome regulatory subunit 8** | **XM_950645** | **6031** | **7191** | **S** |
| **ECU07_1780** | **hypothetical protein v26.11** | **NM_001041727** | **7205** | **8041** | **AS** |
| **ECU09_1820** | **Hypothetical Protein** | **XM_950643** | **8737** | **9495** | **AS** |
| **ECU09_1800** | **DNA helicase** | **XM_950640** | **9689** | **12448** | **S** |
| **ECU09_1790** | **ATP synthase** | **XM_950639** | **12458** | **14614** | **AS** |
| **ECU09_1805** | **U6 snRNA-associated Ribonucleoprotein** | **XM_950641** | **14661** | **14975** | **AS** |
| **ECU09_1770** | **Guanine Nucleotide-Binding Protein Subunit Beta** | **XM_950637** | **14998** | **16050** | **S** |
| **ECU09_1760** | **Hypothetical Protein** | **XM_950636** | **16111** | **17526** | **S** |
| **ECU09_1740** | **Phospholipase A2 activating Protein** | **XM_950634** | **17542** | **19188** | **S** |
| **ECU07_0100** | **similarity to NGG1-Interacting Factor 3** | **NM_001041560** | **19309** | **19743** | **S** |
| **ECU08_1220** | **Hypothetical Protein** | **NM_001041861** | **19764** | **20081** | **AS** |
| **ECU08_1210** | **Hypothetical Protein** | **NM_001041860** | **20141** | **21310** | **AS** |
| **ECU09_1210** | **GPI-Anchor Biosynthesis Protein** | **XM_950579** | **21353** | **22117** | **S** |
| **ECU08_1100** | **Coatomer Complex Beta Subunit** | **NM_001041849** | **22136** | **24505** | **AS** |
| **ECU08_1110** | **Guanine Nucleotide Binding Protein Beta Subunit** | **NM_001041850** | **24674** | **25642** | **S** |
| **ECU08_1130** | **Hypothetical Protein** | **NM_001041852** | **27542** | **28153** | **AS** |
| **ECU11_1770** | **NIFS-LIKE Protein** | **NM_001042316** | **28183** | **29427** | **AS** |
| **ECU10_1710** | **Hypothetical Protein** | **NM_001042121** | **29473** | **30243** | **S** |
| **ECU07_0580** | **Hypothetical Protein** | **NM_001041608** | **30256** | **31206** | **AS** |
| **ECU08_1160i** | **Ribosomal Protein L23** | **NM_001041855** | **31539** | **31985** | **AS** |
| **ECU06_1190** | **putative GTP-Binding Protein** | **NM_001041497** | **32032** | **33147** | **AS** |
| **ECU08_1180** | **Hypothetical Protein** | **NM_001041857** | **33869** | **35974** | **S** |
| **ECU09_1270** | **Hypothetical Protein** | **XM_950585** | **36176** | **36907** | **AS** |
| **ECU10_1160** | **Hypothetical Protein** | **NM_001042064** | **36943** | **38985** | **AS** |
| **ECU07_0350** | **Required for Nuclear Division CUT1** | **NM_001041585** | **39013** | **41118** | **S** |
| **ECU05_0840** | **Sorting Nexin GRD19 Homolog** | **NM_001041292** | **41186** | **41560** | **AS** |
| **ECU11_2060** | **60S Ribosomal Protein L35** | **NM_001042345** | **41711** | **42073** | **S** |
| **ECU09_1670** | **Hypothetical Protein** | **XM_950627** | **42144** | **42854** | **S** |
| **ECU04_1470** | **Na+/H+ Antiporter** | **NM_001041182** | **43393** | **44616** | **S** |
| **ECU04_1280** | **Acylglycerol-3-Phosphate Acyltrasferase** | **NM_001041162** | **46028** | **46900** | **AS** |
| **ECU04_1590** | **Hypothetical Protein** | **NM_001041194** | **49156** | **49710** | **S** |
| **ECU04_1570** | **Hypothetical Protein** | **NM_001041192** | **49831** | **50235** | **AS** |
| **ECU04_1540** | **Hypothetical Protein** | **NM_001041189** | **50273** | **50581** | **S** |
| **ECU04_1560** | **GTP-Binding Nuclear Protein** | **NM_001041191** | **51349** | **52053** | **S** |
| **ECU04_1500** | **Regulator of Chromosome Condensation** | **NM_001041185** | **52198** | **53313** | **S** |
| **ECU08_1690** | **putative zinc finger protein** | **NM_001041906** | **53377** | **54027** | **AS** |
| **ECU07_0950** | **similarity to Replication factor A Protein 3** | **NM_001041645** | **54034** | **54318** | **AS** |
| **ECU01_0230** | **Hypothetical Protein** | **XM_960765** | **54344** | **55030** | **S** |
| **ECU02_1080** | **similarity to Monoubiquitin/Carboxy-extension protein fusion** | **NM_001040822** | **55104** | **55326** | **S** |
| **ECU09_0395** | **60S Ribosomal protein L39** | **XM_950499** | **55367** | **55531** | **AS** |
| **ECU05_0540** | **Hypothetical Protein of the PI3/PI4 Kinase** | **NM_001040822** | **55595** | **56689** | **AS** |
| **ECU01_0430** | **Hypothetical Protein** | **XM_960784** | **56723** | **57286** | **S** |
| **ECU06_0290** | **Hypothetical Protein** | **NM_001041407** | **57311** | **57991** | **S** |
| **ECU08_0360** | **similarity with Ornithine Decarboxylase DCOR_YEAST** | **NM_001041774** | **58043** | **58276** | **S** |
| **ECU08_0500** | **Hypothetical Protein** | **NM_001041788** | **58282** | **58671** | **AS** |
| **ECU02_0510** | **MPS1-like THR/TYR dual Specificity Protein kinase** | **NM_001040767** | **58724** | **59308** | **AS** |
| **ECU01_0380** | **Hypothetical Protein** | **XM_960779** | **59326** | **60285** | **AS** |
| **ECU11_1440** | **Hypothetical Protein** | **NM_001042283** | **62252** | **62965** | **AS** |
| **ECU06_1430** | **Cell Division Control Protein CDC23** | **NM_001041521** | **63130** | **64461** | **S** |
| **ECU06_1440** | **Translation Elongation Factor 1-ALPHA** | **NM_001041522** | **64534** | **65865** | **S** |
| **ECU05_0160** | **Putative aminoacid transporter** | **NM_001041222** | **69824** | **70570** | **AS** |
| **ECU05_1300** | **Hypothetical Protein** | **NM_001041339** | **70962** | **71972** | **S** |
| **ECU02_0340** | **Proteasome Theta Subunit** | **NM_001040750** | **73664** | **73909** | **AS** |
| **ECU07_0680** | **CUT3-Like Chromosome Segregation Protein** | **NM_001041618** | **74514** | **75362** | **S** |
| **ECU07_1420** | **20S Proteasome alpha-type subunit** | **NM_001041692** | **76018** | **76848** | **AS** |
| **ECU06_0620** | **Histydyl tRNA Synthetase** | **NM_001041440** | **76874** | **78220** | **AS** |
| **ECU09_0700** | **Hypothetical Protein** | **XM_950531** | **78323** | **80119** | **S** |
| **ECU07_1440** | **Hypothetical Protein C215** | **NM_001041694** | **80175** | **84788** | **S** |
| **ECU07_1460** | **Ribosomal Protein L37** | **NM_001041696** | **84837** | **85094** | **S** |
| **ECU07_1470** | **Hypothetical Protein** | **NM_001041697** | **85186** | **86199** | **S** |
| **ECU11_1310** | **NADPH Adrenodoxin Oxidoreductase** | **NM_001042270** | **86270** | **86956** | **AS** |
| **ECU04_1000** | **Myosin Heavy Chain** | **NM_001041133** | **87096** | **88529** | **AS** |
| **ECU07_1500** | **Cation-Transporting ATPase** | **NM_001041700** | **91359** | **94787** | **S** |
| **ECU04_1060** | **Dolichol-Phosphate Mannosyltransferase** | **NM_001041140** | **94796** | **98524** | **S** |
| **ECU01_0980** | **anti-telomeric silencing protein** | **XM_960841** | **98526** | **99191** | **AS** |
| **ECU01_0950** | **Hypothetical Protein** | **XM_960837** | **99213** | **100400** | **AS** |
| **ECU01_0930i** | **Thioredoxin** | **XM_960835** | **101216** | **101755** | **AS** |
| **ECU04_0280** | **Suppressor of Stem Loop Protein 1** | **NM_001041061** | **102355** | **102729** | **AS** |
| **ECU02_1250** | **Transcription Initiation Factor TFIID 28kDa Subunit** | **NM_001040839** | **102852** | **103205** | **S** |
| **ECU08_0580** | **Hypothetical Protein** | **NM_001041796** | **105079** | **105579** | **S** |
| **ECU01_0840** | **ribonucleoprotein** | **XM_960826** | **107474** | **107950** | **AS** |
| **ECU04_1510** | **Hypothetical Protein** | **NM_001041186** | **107980** | **108816** | **AS** |
| **ECU04_0780** | **Eukaryotic Translation Initiation Factor 6** | **NM_001041111** | **110764** | **111480** | **AS** |
| **ECU06_0230i** | **Nucleolar Protein** | **NM_001041401** | **117972** | **118688** | **AS** |
| **ECU06_0260** | **Trascription Factor of the E2F/DP Family** | **NM_001041404** | **122064** | **122660** | **AS** |
| **ECU07_1760** | **Glucosamine Phosphate N-Acetyltransferase** | **NM_001041725** | **122877** | **123446** | **S** |
| **ECU07_0540** | **Putative ATP Binding Protein** | **NM_001041604** | **123521** | **124279** | **S** |
| **ECU01_1370** | **Septin Homolog** | **XM_960882** | **124549** | **125454** | **S** |
| **ECU05_1400** | **26S Proteasome Zeta Chain** | **NM_001041349** | **125477** | **126166** | **AS** |
| **ECU05_1370** | **Zinc Metalloprotease** | **NM_001041346** | **126193** | **127428** | **AS** |
| **ECU05_1380** | **Hypothetical Protein YD98** | **NM_001041347** | **127492** | **128025** | **AS** |
| **ECU05_1350** | **Pseudouridine Synthase B** | **NM_001041344** | **128059** | **128559** | **AS** |
| **ECU05_1330** | **Hypothetical Protein** | **NM_001041342** | **129397** | **129933** | **S** |
| **ECU05_1320** | **U3 Small Nucleolar Ribonucleoprotein** | **NM_001041341** | **129997** | **130539** | **S** |
| **ECU05_1310** | **U6 snRNA-associated Small Ribonucleoprotein** | **NM_001041340** | **130574** | **130984** | **S** |
| **ECU05_1280** | **Double-Strand Break DNA Repair Protein** | **NM_001041337** | **130990** | **132336** | **AS** |
| **ECU03_0670** | **Belongs to the Ribonuclease HII Family** | **NM_001040942** | **132582** | **133340** | **AS** |
| **ECU05_1240** | **Gamma Glutamyl Transpeptidase** | **NM_001041333** | **133391** | **135130** | **AS** |
| **ECU05_1260** | **Hypothetical Protein** | **NM_001041335** | **135218** | **136225** | **AS** |
| **ECU05_1250** | **CDP-Diacylglycerol Synthase** | **NM_001041334** | **136250** | **137197** | **AS** |
| **ECU10_0560** | **Oxidoreductase of the Short Chain Dehydrogenase** | **NM_001042004** | **137400** | **138119** | **S** |
| **ECU05_1220** | **Hypothetical Protein** | **NM_001041331** | **138128** | **138946** | **AS** |
| **ECU09_0500** | **Hypothetical Protein** | **XM_950510** | **138994** | **139980** | **AS** |
| **ECU05_1210** | **DNA Polymerase alpha/Primase Large Subunit** | **NM_001041330** | **140011** | **141207** | **S** |
| **ECU09_0490** | **Hypothetical Protein** | **XM_950509** | **141215** | **142930** | **AS** |
| **ECU09_0480** | **T complex protein 1 subunit beta** | **XM_950508** | **143722** | **145305** | **AS** |
| **ECU09_0440** | **Histone H4** | **XM_950504** | **145970** | **146077** | **AS** |
| **ECU09_0430** | **DNA polymerase delta catalytic subunit** | **XM_950503** | **146255** | **149161** | **S** |
| **ECU09_0400** | **mRNA capping enzyme** | **XM_950500** | **149192** | **150313** | **AS** |
| **ECU08_0880** | **Endonuclease III** | **NM_001041827** | **150448** | **150705** | **S** |
| **ECU06_1250** | **ATP-Dependent RNA Helicase** | **NM_001041503** | **151159** | **155349** | **AS** |
| **ECU01_1060** | **DNA repair helicase** | **XM_960849** | **155373** | **157274** | **AS** |
| **ECU09_0980** | **serine/threonine protein phosphatase catalytic subunit** | **XM_950559** | **157286** | **158161** | **AS** |
| **ECU04_0680** | **RAS-Related GTP-Binding Protein RAB11** | **NM_001041101** | **158224** | **158901** | **S** |
| **ECU06_1230** | **Hypothetical Protein YO7T** | **NM_001041501** | **158898** | **160118** | **AS** |
| **ECU04_0660** | **Putative Lipase** | **NM_001041099** | **160158** | **160976** | **AS** |
| **ECU04_0630** | **Ubiquitin Conjugating Enzyme E2 18kDa Subunit** | **NM_001041096** | **161621** | **162133** | **S** |
| **ECU07_0330** | **Putative Protein with Zinc Finger Domain** | **NM_001041583** | **163066** | **163821** | **S** |
| **ECU07_1780** | **Hypothetical protein v26.11** | **NM_001041727** | **163840** | **164340** | **S** |
| **ECU03_1380** | **similarity to Pelota Protein** | **NM_001041010** | **165697** | **166728** | **AS** |
| **ECU03_1420** | **Hypothetical Protein** | **NM_001041014** | **166817** | **168679** | **AS** |
| **ECU03_1430** | **RAS-Like GTP-binding Protein YPT1** | **NM_001041015** | **168771** | **169373** | **S** |
| **ECU03_0850** | **Hypothetical Protein** | **NM_001040958** | **170727** | **171542** | **AS** |
| **ECU03_1460** | **Histone H3** | **NM_001041018** | **171790** | **172239** | **S** |
| **ECU03_1110** | **Hypothetical Protein** | **NM_001040984** | **172284** | **172562** | **S** |
| **ECU04_0170** | **Hypothetical Protein** | **NM_001041051** | **172768** | **173682** | **S** |
| **ECU04_0180** | **Transcription Elongation Factor SII** | **NM_001041052** | **173691** | **174233** | **AS** |
| **ECU04_0330** | **60S Ribosomal Protein L27** | **NM_001041066** | **174280** | **174726** | **AS** |
| **ECU04_0320** | **Hypothetical Protein** | **NM_001041065** | **174796** | **176370** | **AS** |
| **ECU04_0310** | **26S Proteasome Regulatory Subunit 4** | **NM_001041064** | **176480** | **178885** | **S** |
| **ECU04_0300** | **Protein Tyrosine Phosphatase** | **NM_001041063** | **179082** | **179549** | **S** |
| **ECU11_1560** | **Protein of the Synthaxin/Epidomorphin FamilyY** | **NM_001042295** | **180545** | **181102** | **AS** |
| **ECU04_0600** | **Aminopeptidase P-Like Protein** | **NM_001041093** | **181293** | **181733** | **AS** |
| **ECU10_1110** | **Polyadenylate-Binding Protein 2** | **NM_001042059** | **181837** | **182127** | **S** |
| **ECU02_1230** | **similarity to SEC24-related protein** | **NM_001040837** | **182433** | **184427** | **AS** |
| **ECU02_1220** | **DNA Ligase** | **NM_001040836** | **184560** | **186446** | **S** |
| **ECU08_0070** | **Glutamyl Aminopeptidase** | **NM_001041745** | **186539** | **187360** | **S** |
| **Scaffold SC_1888** |  |  |  |  |  |
| **ECU02_1190** | **Hypothetical Protein** | **NM_001040833** | **1606** | **2109** | **S** |
| **ECU01_1240** | **Hypothetical Protein** | **XM_960869** | **2394** | **2678** | **AS** |
| **ECU01_1190** | **Hypothetical Protein** | **XM_960864** | **2709** | **3917** | **S** |
| **ECU07_0900** | **similarity to Hypothetical Protein YM60_yeast** | **NM_001041640** | **4132** | **5313** | **S** |
| **ECU02_1140** | **Signal Peptidase 21kDa Subunit** | **NM_001040828** | **7047** | **7598** | **AS** |
| **ECU07_0830** | **HFM1-Like ATP-Dependent RNA Helicase** | **NM_001041633** | **7673** | **10558** | **S** |
| **ECU10_0470** | **Hypothetical Protein** | **NM_001041995** | **10573** | **11748** | **AS** |
| **ECU04_0210** | **Glucose Transporter Type 3 [** | **NM_001041055** | **12023** | **13129** | **AS** |
| **ECU02_1130** | **Protein Kinase of the PI3/PI4 Family** | **NM_001040827** | **13194** | **14753** | **AS** |
| **ECU04_0610** | **Hypothetical Protein** | **NM_001041094** | **18524** | **19288** | **AS** |
| **ECU01_1050** | **Hypothetical Protein** | **NC_003242** | **19438** | **21162** | **AS** |
| **ECU02_1100** | **HSP90 Homolog** | **NM_001040824** | **22840** | **24855** | **S** |
| **ECU02_1090** | **ATP-Dependent DNA-Binding Helicase (RAD3/XPD subfamily)** | **NM_001040823** | **24898** | **26910** | **AS** |
| **ECU02_1080** | **Monoubiquitin** | **NM_001040822** | **26966** | **27415** | **AS** |
| **ECU02_0640** | **Transcriptional Repressor for RNA Polymerase II** | **NM_001040779** | **27643** | **27987** | **AS** |
| **ECU10_1780** | **Hypothetical Protein** | **NM_001042128** | **28005** | **29255** | **S** |
| **ECU08_1300** | **ADP/ATP Carrier Protein 1** | **NM_001041869** | **31471** | **33111** | **S** |
| **ECU10_1720** | **3-Hydroxy-3-MethylGlucaryl CoA Reductase** | **NM_001042122** | **33129** | **33350** | **AS** |
| **ECU11_1260** | **DNA Mismatch Repair Protein of the MUTL/HEXB Family** | **NM_001042265** | **33371** | **33679** | **S** |
| **ECU08_1370** | **Hypothetical Protein** | **NM_001041874** | **34131** | **34904** | **S** |
| **ECU08_1380** | **Glutaredoxin** | **NM_001041875** | **34907** | **35197** | **AS** |
| **ECU08_1410** | **Hypothetical Protein** | **NM_001041878** | **35289** | **36205** | **AS** |
| **ECU08_1420** | **similarity with phosphatidylinositol phosphate phosphatase** | **NM_001041879** | **36370** | **37914** | **S** |
| **ECU08_1430** | **Hypothetical Protein** | **NM_001041880** | **38269** | **39120** | **AS** |
| **ECU04_0960** | **Hypothetical Protein** | **NM_001041129** | **39188** | **39433** | **S** |
| **ECU04_0900** | **Phenylalanyl tRNA Synthetase alpha chain** | **NM_001041123** | **39452** | **41152** | **S** |
| **ECU08_1440** | **Hypothetical Protein** | **NM_001041881** | **41160** | **43082** | **AS** |
| **ECU08_1290** | **Farnesyl Transferase beta subunit** | **NM_001041868** | **44367** | **45341** | **S** |
| **ECU04_0580** | **Lysyl tRNA Synthetase** | **NM_001041092** | **47292** | **48848** | **AS** |
| **ECU04_1190** | **Putative Aminoacid Transporter YEU9** | **NM_001041153** | **48986** | **50215** | **AS** |
| **ECU08_1520** | **Vacuolar ATP Synthase Sununit AC39** | **NM_001041889** | **50396** | **51259** | **S** |
| **ECU08_0630** | **DNA Polymerase Alpha Complex** | **NM_001041801** | **51860** | **52687** | **S** |
| **ECU07_0130** | **Anti-Silencing Protein 1** | **NM_001041563** | **52734** | **53585** | **S** |
| **ECU07_0120** | **Hypothetical Protein** | **NM_001041562** | **54162** | **55226** | **AS** |
| **ECU07_0110** | **60S Acidic Ribosomal Protein P2** | **NM_001041561** | **55265** | **55567** | **AS** |
| **ECU07_0080** | **Hypothetical Protein** | **NM_001041558** | **55600** | **56370** | **S** |
| **ECU09_0210** | **Hypothetical Protein** | **XM_950480** | **56411** | **56743** | **S** |
| **ECU09_0490** | **Hypothetical Protein** | **XM_950509** | **56783** | **57490** | **S** |
| **ECU10_1790** | **Glycyl-tRNA Synthetase** | **NM_001042129** | **57555** | **59432** | **S** |
| **ECU07_0180** | **Acid Phosphatase Precursor** | **NM_001041568** | **59686** | **61455** | **S** |
| **ECU06_1380** | **Soma Ferritin** | **NM_001041516** | **61470** | **61970** | **AS** |
| **ECU07_0190** | **26S Proteasome Regulatory Subunit 10** | **NM_001041569** | **62044** | **63210** | **AS** |
| **ECU07_0200** | **Hypothetical Protein** | **NM_001041570** | **63310** | **64938** | **S** |
| **ECU07_0210** | **Hypothetical Protein** | **NM_001041571** | **65024** | **65455** | **S** |
| **ECU07_0290** | **Hypothetical Protein** | **NM_001041579** | **65605** | **66450** | **S** |
| **ECU07_0360** | **SER/THR/TYR Protein Kinase** | **NM_001041586** | **67337** | **68137** | **AS** |
| **ECU07_0370** | **Hypothetical Protein** | **NM_001041587** | **68197** | **68889** | **AS** |
| **ECU01_0140** | **glutamyl-aminopeptidase** | **XM_960758** | **70024** | **72531** | **AS** |
| **ECU07_0270** | **Hypothetical Protein** | **NM_001041577** | **72578** | **73420** | **AS** |
| **ECU07_0390** | **RAD18-Like Recombination and DNA Repair Protein** | **NM_001041589** | **73425** | **76247** | **AS** |
| **ECU07_0410** | **Ubiquitin Carboxil Terminal Hydroxyl** | **NM_001041591** | **77083** | **77940** | **S** |
| **ECU05_0110** | **Hypothetical Protein** | **NM_001041217** | **78004** | **78876** | **AS** |
| **ECU11_0620** | **DiacylGlycerol Ethanolamine Phosphotransferase** | **NM_001042201** | **81027** | **82037** | **AS** |
| **ECU05_1120i** | **Tyrosyl tRNA Synthetase** | **NM_001041321** | **83689** | **84522** | **S** |
| **ECU07_0450** | **Integral Membrane Protein YAB9** | **NM_001041595** | **84536** | **87445** | **AS** |
| **ECU07_0460** | **Nuclear Protein of the NHP2/RS6 Family** | **NM_001041596** | **87543** | **87887** | **AS** |
| **ECU07_0470** | **Hypothetical Protein** | **NM_001041597** | **87932** | **88300** | **AS** |
| **ECU07_0490** | **DNA Replication Licensing Factor MCM7** | **NM_001041599** | **89453** | **91090** | **AS** |
| **ECU06_0930** | **Phospholippid Transportng ATPase** | **NM_001041471** | **91181** | **91741** | **S** |
| **ECU06_0440** | **Hypothetical Protein YATA** | **NM_001041422** | **91759** | **93354** | **S** |
| **ECU06_0540** | **putative SKT5-like protein** | **NM_001041432** | **93398** | **94573** | **AS** |
| **ECU06_1610** | **Hypothetical Protein** | **NM_001041540** | **94682** | **94885** | **S** |
| **ECU10_1120** | **TFIID 70kDa Subunit** | **NM_001042060** | **95476** | **96363** | **S** |
| **ECU02_1380** | **CAAX Prenyl Protease 1** | **NM_001040852** | **96474** | **97742** | **AS** |
| **ECU07_0580** | **Hypothetical Protein** | **NM_001042180** | **97780** | **98748** | **AS** |
| **ECU02_1030** | **Hypothetical Protein** | **NM_001040817** | **99215** | **100882** | **AS** |
| **ECU02_1400** | **similarity to Hypothetical WD-repeat Protein YN57** | **NM_001040854** | **101120** | **102289** | **S** |
| **ECU02_1410** | **Secretory Pathway GDP Dissociation Inhibitor alpha** | **NM_001040855** | **102358** | **103644** | **S** |
| **ECU02_1430** | **similarity to Hypothetical ATP-binding Protein YJ42** | **NM_001040857** | **103664** | **104419** | **S** |
| **ECU02_1340** | **similarity to Hypothetical Protein YA7C** | **NM_001040848** | **106173** | **107435** | **AS** |
| **ECU07_0720** | **Hypothetical Protein** | **NM_001041622** | **108235** | **108723** | **S** |
| **ECU02_1360** | **Prolyl tRNA Synthetase** | **NM_001040850** | **108747** | **110252** | **S** |
| **ECU02_1330** | **Hypothetical Protein** | **NM_001040847** | **110390** | **110698** | **S** |
| **ECU02_1320** | **similarity to RAD4 protein** | **NM_001040846** | **110755** | **112458** | **S** |
| **ECU02_1240** | **13S Condensin Subunit** | **NM_001040838** | **112466** | **115024** | **AS** |
